# Improving the Reliability of Scale-Free Image Morphometrics in Applications with Minimally Restrained Livestock Using Projective Geometry and Unsupervised Machine Learning

**DOI:** 10.3390/s22218347

**Published:** 2022-10-31

**Authors:** Catherine McVey, Daniel Egger, Pablo Pinedo

**Affiliations:** 1 Department of Animal Sciences, Colorado State University, Fort Collins, CO 80523, USA; 2 Pratt School of Engineering, Duke University, Durham, NC 27708, USA

**Keywords:** scale free morphometrics, Euclidean distance matrix analysis, projective biometrics, facial morphology, facial expression, unsupervised machine learning, precision livestock farming, dairy welfare

## Abstract

Advances in neural networks have garnered growing interest in applications of machine vision in livestock management, but simpler landmark-based approaches suitable for small, early stage exploratory studies still represent a critical stepping stone towards these more sophisticated analyses. While such approaches are well-validated for calibrated images, the practical limitations of such imaging systems restrict their applicability in working farm environments. The aim of this study was to validate novel algorithmic approaches to improving the reliability of scale-free image biometrics acquired from uncalibrated images of minimally restrained livestock. Using a database of 551 facial images acquired from 108 dairy cows, we demonstrate that, using a simple geometric projection-based approach to metric extraction, a priori knowledge may be leveraged to produce more intuitive and reliable morphometric measurements than conventional informationally complete Euclidean distance matrix analysis. Where uncontrolled variations in image annotation, camera position, and animal pose could not be fully controlled through the design of morphometrics, we further demonstrate how modern unsupervised machine learning tools may be used to leverage the systematic error structures created by such lurking variables in order to generate bias correction terms that may subsequently be used to improve the reliability of downstream statistical analyses and dimension reduction.

## 1. Introduction

Methodological advances in machine vision, namely the rapid evolution of neural network-based approaches to image segmentation and analysis, have garnered growing interest in the utility of imaging systems in livestock management [1]. For some applications, existing databases may be suitable with respect to both size and structure to directly implement these cutting-edge analytical techniques. For example, algorithmic approaches to the evaluation of livestock confirmation are not only able to train on large image databases curated by breed improvement programs, but can also leverage the depth of literature on linear type traits to evaluate the efficacy of the resulting model [2]. Other applications of machine vision, however, may seek to extract information from livestock systems that has not previously been quantified due to issues with either accuracy or practicality. Applications related to livestock behavior and welfare may be particularly apt to fall into this latter category [3,4]. For use cases where an image database must be purpose-built for the task, perhaps with little prior literature to inform optimal sampling strategies or even how response features should be encoded, the scale and complexity of neural network-based analyses may constitute a considerable barrier to entry. Where such gaps in the literature base arise, preliminary analyses that employ less data-hungry approaches to image analysis may still serve as critical stepping stones towards these more sophisticated models, provided they return reliable results.

Landmark-based approaches to image analysis are a natural choice for such exploratory analyses. While not informationally complete, they can be easily implemented with manual annotation protocols in a range of programs without the extensive image preprocessing required by embedding techniques [5,6]. How the information contained in coordinate vectors is then compressed into 1D biometrics suitable for statistical analysis varies widely with the use case. As much of the existing literature base on image analysis in livestock has been geared towards estimation of body weight, many well-validated methods emphasize the absolute size of target features. Such measurements, however, must be derived from extrinsically calibrated imaging systems that require animals to be restrained with little freedom of movement so that either focal distance can be held constant, as with calibrated 2D systems [7,8,9], or to facilitate the limited focal range of 3D depth cameras [10,11,12,13]. Such restraint is, however, not conducive to many applications, namely those attempting to quantify behavioral responses [3,4]. Where instead the relative position (shape) of target features may suffice, such information may be gleaned from uncalibrated 2D images [14], but there is comparatively far less literature available on the reliability and resilience of such scale-free biometric measurements of livestock under minimal restraint.

One readily generalizable strategy that may be used to extract information about the shape of target features from coordinate vectors is to simply employ pre-defined geometric transforms that are intrinsically scale-free. Angle measures have been used extensively in definitions of subjective conformation scoring scales, and have subsequently been extended to analysis of livestock images. Previous work exploring the use of angle-based measures of leg conformation in breeding sows [15,16] and several horse breeds [17,18] have generally reported good within-photo repeatability (>0.5) over multiple annotations, but a wide range of between-photo repeatability estimates have been reported with replicated imaging, from as high as 0.96 to as low as 0.34. Curvature measures have been employed more sparingly, but have previously been validated against subjective lameness scores to quantify back arching in cattle [19]. While geometrically defined metrics allow for targeted extraction of readily interpretable shape measures, this approach may not suffice to capture more complex geometric features.

A more informationally complete strategy employed to produce scale-free biometrics is to align coordinate vectors across images via an affine transformation and then extract shape features using an unsupervised dimension reduction technique. Geometric morphometric (GM) analysis is a well-established method in anthropological studies that combines Procrustes alignment with gPCA feature extraction [14]. In work by Druml and colleagues, GM analyses successfully recovered holistic distinctions in overall body type amongst Lipizzaner breeding stock, but was significantly impacted by changes in animal stance and pose [20,21,22]. In a follow up study, high within-photo repeatability (>0.9) and good between-digitizer reproducibility (>0.7) were reported, but between-photo repeatability estimates were not provided [18]. In Alhajeri et al. (2019) GM analyses also recovered significant differences in hump morphology between the two major classes of camel breeds in the Arabian peninsula [23]. PCA and kernel PCA have also been used after affine point alignments to relate variations in topline morphology to body condition scores in cattle [24,25]. In order to fully circumvent with camera position, GM analyses have recently been extended to 3D images using advanced photogrammetric techniques [26]. Unfortunately, these preliminary studies failed to recover significant differences in facial grimace in piglets following castration, which the studies speculated were due to difficulties with image alignment and the invasiveness of the imaging system. While such approaches provide more holistic descriptions of shape, the intensive image acquisition, annotation, and alignment protocols required makes these techniques more challenging to implement, and the subsequent dimension reduction may produce metrics that are more challenging to interpret or extrapolate to new data. 

The purpose of this study was to characterize the efficacy and reliability of two additional strategies to producing scale-free biometrics from uncalibrated 2D images of minimally restrained livestock that may offer an intermediate approach between these two existing analytical paradigms. The first was globally normalized Euclidean distance measures, here referred to as *normalized length biometrics*, an informationally complete approach to extracting coordinate-free descriptors of shape without preliminary affine alignment [14]. The second method sought to employ a wider range of readily generalizable vector projection-based techniques, hereafter referred to as *projective biometrics*, to produce locally normalized biometric values that could accommodate a wider range of complex morphological features than simple angle measures [27]. Detailed measurement systems analyses were carried out to compare not only the precision of these two morphometrics systems, but also their susceptibility to bias in the face of lurking variables that could be readily controlled in working farm environments, as well as their amenability to bias correction strategies using novel unsupervised machine learning techniques.

## 2. Materials and Methods

In this study, structural variations in the osseous and cartilaginous features of the bovine face were utilized as a model to contrast the performance of the normalized length and projective biometric approaches to scale-free measurement. The face was chosen as a model over more conventional type (conformation) traits for several reasons. From a theoretical standpoint, the face offered a diverse range of geometrically complex morphological features arranged densely over a compact anatomical region, which provided a comprehensive sampling of prospective geometric relationships from a manageable number of annotated points. Further, skull morphology could be subdivided into localized regions that facilitated functional comparisons between measurement systems, with some subregions featuring only fixed boney traits, and thus subject only to annotation and projection errors, and fleshier subregions, wherein annotation of underlying osseous and cartilaginous landmarks might also be influenced by changes in facial expressions (i.e., -pose). From a more pragmatic standpoint, the head is highly mobile with respect to all three axes of movement (pitch, roll, and yaw) even while the cow is otherwise stationary in a headlock, which facilitated location of a large number of individual animals in a working farm environment for repeated sampling. Finally, while comparisons between measurement systems are intended to be readily generalizable to a broader scope of prospective use cases, there are emerging utilitarian interests in assessment of livestock faces to extract measures of facial expression [26,28,29,30,31,32,33,34] and morphology [27,34]. The authors hope that this work may provide more direct methodological insights for the inevitable progression of this growing body of research towards objective image-based measurements.

### 2.1. Image Acquisition and Annotation

Animal handling and imaging protocols implemented in this study were approved by the Colorado State University Institution of Animal Care and Use Committee (IACUC #16-6816A). This study followed all guidelines for the use and care of agricultural animals used in research outlined in the Colorado State University IACUC Composition and Responsibilities Policies and the Colorado State University Policy on the Use of Live Vertebrate Animals.

Facial images of 108 mature Holstein dairy cows were collected over a two-week period on a commercial dairy in July 2017 using an Olympus TG-2 iHS 12MP Waterproof Camera. Cows were photographed while locked in the feed bunk of their home pen while standard herd checks were being performed (not longer than 90 min). The distance between the cow and camera person was allowed to vary from 1 to 3 standard bunk spaces, as determined by the unmanipulated arrangement of animals in the headlocks at roughly 50% stocking capacity. To better emulate a feasible experimental protocol, gross variations in out-of-plane facial angle were controlled by attempting to align as closely as possible the outlines of the proximal and distal eye orbitals.

The procedural target was to photograph each cow from both side profiles on three separate days. Of the 108 cows recorded, complete image profiles were generated for 74 animals. The remaining cows were either not located for a third day of photographing or had an image discarded farther down the analysis pipeline due issues with image quality not identified on farm: poor image resolution, shadows, or feed and dirt obscuring facial landmarks. Missing values appeared randomly distributed across the herd, however, and not driven by any readily identifiable form of sampling bias. In total, 551 images were deemed suitable for analysis. Photos were analyzed using the image analysis toolbox in MatLab R2016a version 9.0.0.341360 (The Mathworks Inc., Natick, MA, USA). A total of 60 unique anatomical landmarks were defined across four distinct anatomical regions: eye, muzzle, topline, and forehead/jaw. Full details on the protocol used to identify these anatomical landmarks, and how they were partitioned between anatomical regions of the face, are provided in supplemental materials. Coordinate locations of these landmarks were extracted manually by a single digitizer (CM) in two annotation replicates, with all animal being annotated within an anatomical subregion before coordinate extractions were repeated.

### 2.2. Morphometric Algorithm Specification

The first strategy employed here to extract scale free biometrics from these annotations was based on Euclidean Distance Matrix Analysis [14]. In this approach, Euclidean distances are calculated between all pairwise combinations of landmark points to produce an informationally complete transformation from 2D coordinates to 1D distances values without preliminary image alignment. To convert the resulting pixel distance values into a scale-free measurement independent of extrinsic factors (cow size, focal distance, etc) and intrinsic parameters (camera resolution, zoom, etc.) influencing image scale, each distance matrix is normalized by the sum of all distance values in order to divide out the scaling factor unique to that image [35,36]. Here, to facilitate independent comparisons, this normalizing term was calculated within each anatomical subregion and applied to the corresponding distance matrix to produced normalized length biometrics.

While Euclidean distances matrices are simple to compute, the number of normalized length biometrics produced grows polynomially O(n^2^) with the number of anatomical landmarks selected, with many of the resulting distance measures being geometrically redundant. Dimension reduction techniques may be applied to reduce the overall problem size, but the resulting aggregate biometrics can be difficult to interpret [14,35,36]. 

In contrast, the second measurement technique used to produce scale free biometrics in this study sought to identify a priori facial features that were visually distinct across the population and break them down into their most basic and interpretable geometric components. In this readily generalizable approach, vector projection techniques were used to impute one or more auxiliary coordinate points at the intersection of anatomical reference lines between landmark features. These auxiliary coordinates were then used to generate distance measures that more narrowly defined the relative geometric arrangement of landmark coordinates. Such distance measures were then re-expressed as ratios in order to divide out the image scaling factor, and are collectively referred to here as projective biometrics.

**Figure 1:**
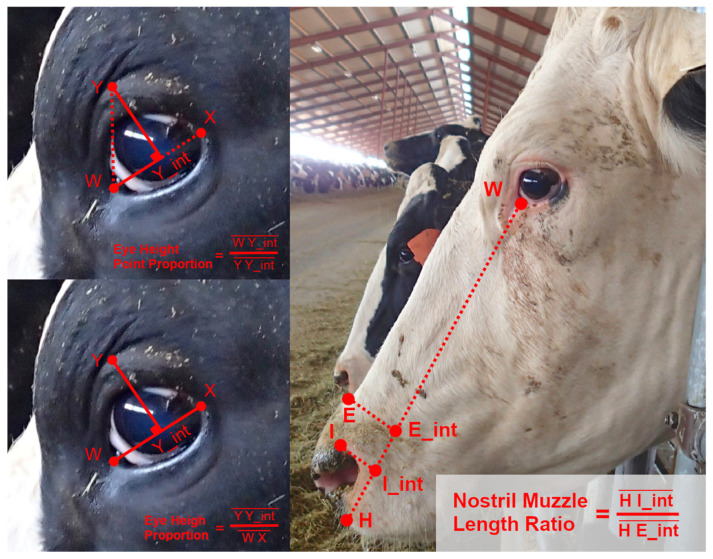
Examples of Projective Biometrics. Eye Height Point Proportion (**upper left**) is an example of an orthogonal projection that is geometrically equivalent to an angle measure via a trigonometric transform. Eye Height Proportion (**bottom left**) provides a more geometrically intuitive definition of eye height using only a single locally defined normalization term. Nostril-Muzzle Length Ratio (**right**) is an example of how projections onto a reference line can provide more anatomically intuitive distance measures.

Using observations from the image database, and considering some conventions from work on equine facial morphology, projective biometrics were derived to measure a total of 92 unique morphological features across all four anatomical subregions that demonstrated visually observable levels of variation across the sample population [37]. For some of these unique features (Figure 1: upper left), orthogonal projections onto anatomical reference lines produced distance ratios that were trigonometrically equivalent to angle measures (and in several cases were converted to angle measures to align with conventions in the lay literature). For other metrics, these anatomical reference lines were used as a more geometrically (Figure 1: bottom left) or anatomically (Figure 1: right) intuitive means of contrasting distances using a single locally defined normalization term. For a subset of these biometrics, multiple versions were computed to explore the most appropriate choice of anatomical reference line. Full details on the derivations of all projective biometrics can be found in supplemental materials. 

### 2.3. Algorithm Validation

All statistical analyses were carried out in R version 3.5.1 [38]. Comparative performance of these two measurement systems was assessed with respect to five characteristics: (1) within- and between-photo repeatability, (2) bias in biometrics attributed to measures of image scale and quality, (3) degree of correlation amongst residual error terms, (4) reliability of composite features produced by dimension reduction techniques, and (5) reliability of metrics following bias correction using unsupervised machine learning techniques. 

#### 2.3.1. Metric Repeatability

Repeatability was assessed for each individual biometric from both measurement systems via an independent mixed effect model using the *lme4* package [39], with nested random effects for cow, side of the face (to allow for structural asymmetry), and photo. Models were optimized using REML criterion, and the resulting variance components used to estimate two forms of repeatability: (1) within-photo repeatability (Equation (1)), a reflection of resilience to errors in landmark point annotation; and (2) overall repeatability (Equation (2)), an estimation of the proportion of variability in a single metric extraction from a sigle photo attributable to the underlying anatomical signal. It should be noted that sample mean and not the true value of the morphological feature was here used to calculate variance terms, and so both these repeatability estimates reflect the precision and not necessarily the accuracy of candidate biometrics.

(1) $$Rep_{WP}=\frac{\sigma^{2}{}_{cow}+\sigma^{2}{}_{side}+\sigma^{2}{}_{photo}}{\sigma^{2}{}_{cow}+\sigma^{2}{}_{side}+\sigma^{2}{}_{photo}+\sigma^{2}{}_{error}}$$

(2) $$Rep_{OA}=\frac{\sigma^{2}{}_{cow}+\sigma^{2}{}_{side}}{\sigma^{2}{}_{cow}+\sigma^{2}{}_{side}+\sigma^{2}{}_{photo}+\sigma^{2}{}_{error}}$$

Provided that there was neither compelling theoretical nor strong empirical evidence to support an assumption that the repeatability estimates of individual biometrics should be independently and identically distributed (see Section 2 of results), formal statistical comparisons could not be made across the two measurement systems. Qualitative comparisons were instead made using visualizations generated via the *ggplot2* package [40]. Histograms were generated for overall repeatability estimates within each anatomical subregion, and a density plot overlaid to compare these results against the within-photo repeatability estimates. For each individual biometric, however, bootstrapped 95% confidence intervals for both repeatability estimates are provided in supplemental materials [39]. 

#### 2.3.2. Resilience to Changes in Image Attributes

**Figure 2:**
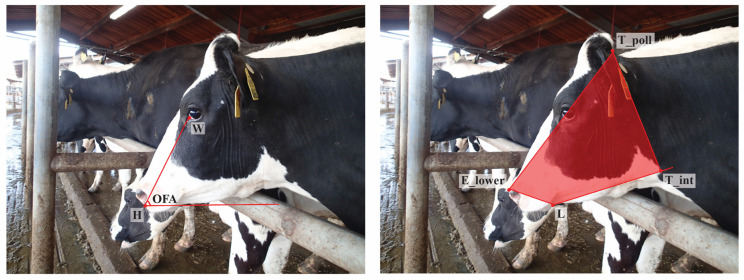
Measures of image quality: Overall Face Angle (**left**) and Frame:Face Ratio (**right**).

To validate the robustness of these measurements systems to bias attributable to variations in image attributes, two auxiliary metrics were extracted from each image. First, to confirm the tolerance of biometrics to in-plane variation in facial angles (pitch), Overall Facial Angle was calculated as the angle formed between the line passing between the rostral-most points of the eye and nose, and the horizontal plane of the image (Figure 2: left). Next, to explore the resilience of biometrics to changes in image scale, and thereby compare the effectiveness of the two length normalization schemes, the Face:Frame Ratio of each image was calculated as the total number of pixels occupied by the cows head, approximated using the area of the polygon formed by anatomical coordinates, divided by the total pixels in the image (Figure 2: right). 

In order to identify systematic correlations with changes in image quality, image attribute estimates were added as fixed effects as in Equation (3) to the mixed models used to estimate repeatability. The total proportion of variability in each observed biometric attributed to this fixed effect model was then estimated via the marginal R^2^ calculated using the *piecewiseSEM* package [18,41]. The two measurement systems were then contrasted via scaled density plots for each anatomical subregion [40]. For each individual biometric, the full model with fixed effects and a reduced model with only a fixed intercept were refit using ML criterion, and a nested model ANOVA test was run to assess the statistical significance of image attribute variables. The resulting chi-squared and *p*-value estimates are provided in supplemental materials, as are the coefficient values estimated for theses scaled image attribute values.

(3) $$fixed\;effect=Frame:Face\;Ratio+{\left(Frame:Face\;Ratio\right)}^{2}+Face\;Angle\;+{\left(Face\;Angle\right)}^{2}+Frame:Face\;Ratio\times Face\;Angle$$

#### 2.3.3. Error Structure Analysis

To explore correlation structures present amongst error terms, mixed models were again independently fit to each biometric as in the repeatability analysis and residuals calculated at the level of side within cow [39]. The *statsby* utility in the *psych* package was then used to decompose overall correlation between residual estimates into within- and between-photo correlations across each pairwise combination of biometrics within a given anatomical subregion of the face [42]. The additive components to overall correlation were then computed using Equation (4) in order to compare the relative impact of these two potential sources of systematic error [42]. To avoid trivial correlations amongst alternative derivations of projective biometrics, only the best version of each metric with respect to repeatability estimates were used to create pairwise combinations. To contrast the performance of the two measurement systems, overall error correlation estimates are presented via histogram with density lines overlaid (with equivalent scaling) representing the additive components attributed to within- and between-photo correlation. *p*-values assessing the significance of the within- and between-group correlation values for each individual biometric are provided in Supplementary Materials.

(4) $$r_{x,y}=eta_{x}{}_{WG}\times eta_{y}{}_{WG}\times r_{x,y}{}_{WG}+eta_{x}{}_{BG}\times eta_{y}{}_{BG}\times r_{x,y}{}_{BG}$$

#### 2.3.4. Reliability of Dimension Reduction

Dimension reduction techniques are frequently employed to concentrate information contained in scale-free biometrics down to a more tractable problem size for statistical analysis. If correlations between biometric measurements are not attributed exclusively to anatomical signal, however, dimension reduction tools may also consolidate information from extraneous within- and between-photo factors that impose systematic error structures between biometrics [14,43]. To assess the susceptibility of these two measurement systems to this risk, dimension reduction was carried out on biometric measurements within each anatomical subregion. Due to the non-independence inherent to repeated measurements, standard PCA could not be applied across observational units in this dataset [43]. To better accommodate the nested structure of this dataset, dimensional reduction was here carried out via Hierarchical Multiple Factor Analysis (HMFA) using the *FactoMineR* package [44,45,46]. The number of basis (loading) vectors generated was set to the total number of biometric features assessed so as to approximate a PCA result. For anatomical subregions where the number of candidate metrics was larger than the number of observational units (a wide embedding problem), an upper limit of 107 dimensions was imposed by the sample size of cows [5].

To evaluate the repeatability of metrics produced by HMFA analysis, partial scores were extracted independently for each cow for the left and right side of the face, aggregating information across nested photo and annotation replicates. Random effects models were then fit independently to these partial scores for each factor dimension, with cow fit here as the only random effect. As the previous repeatability analyses revealed the proportion of variance attributed to side effect (fluctuating asymmetry) to be minimal (generally < 0.1), repeatability was then calculated at the cow level to approximate the overall repeatability of each HMFA score. For each anatomical subregion, scatter plots were then generated comparing these repeatability estimates to the total proportion of variance assigned to each corresponding basis (loading) dimension [40]. 

To more directly evaluate the influence of systematic error structures on the results of the dimension reduction on the observed metric, HMFA analysis was also carried out on the residual values generated to evaluate error structure. Any dimensions that explained at least 5% of the residual variability were retained for the error basis. If no bases met this criterion, the error basis consisted of only the first principal axis generated by the HMFA analyses. Canonical correlation analyses were then conducted to compare the resulting error basis space to each of the individual basis dimensions estimated from the observed data [5,47]. The resulting correlation between axes for the nominal and residual measurement estimates, which equate to the cosine of the angle between the error subspace and observed basis vector, were then applied as a continuous color scale to the scatter plots generated from the repeatability estimates. 

#### 2.3.5. Reliability of Bias Corrected Biometrics

Systematic error structures created between biometrics by lurking variables have the potential to lead downstream statistical analyses astray. Where repeated measures are available to provide, through repetition, a reasonable estimate of the true value of a morphometric feature, we hypothesized that these same residual correlation structures between biometrics might be leveraged by unsupervised machine learning tools to empirically recover an approximation of the link function between lurking variables and observed measurements [48]. This might then provide reasonable approximations of measurement bias in each observation, even when the causative factors cannot be directly inferred or quantified, which could in turn serve as bias correction terms to improve the accuracy of variance and BLUP estimates for individual biometrics.

To create encodings of the systematic error structures found in both morphometric system, we here utilized clustering utilities that we have introduced in previously work that are available in the Livestock Informatics Toolkit (*LIT* package) in R [49]. First, fully nested mixed effect models with no fixed effects were fit to each individual biometric, and the residual estimates extracted as the difference between the observed measurement and BLUP estimates at the level of the image replicate in order to isolate error structures attributable to image annotation. The residuals for all biometrics within a given subregion of the face were then compiled into a single matrix, wherein residuals for each individual biometric were centered and scaled to uniform variance. Data Mechanics, an iterative hierarchical clustering algorithm that can be used to reweight and subsequently share structural information between the row and column axes of a data matrix, was here used to simultaneously cluster together images whose latent attributes that impacted the accuracy of annotation created similar patterns in residual estimates across biometrics, and also biometrics with similar bias responses across images [50,51]. Clustering was here performed on a grid of metaparameter values for cluster granularity from 5 to 8 for row clusters and 2 to 5 for column clusters [52]. Heatmap visualizations of the clustering results were then visually inspected to select to simplest (coarsest) encoding that captured all appreciable systematic fluctuations in residual estimates [50]. From these results the dendrogram fitted to the row (image) index of the residual matrix was pruned to create a discrete variable to serve as a bias correction term for errors in annotation. 

To create an optimal encoding of systematic error attributed to image level attributes (camera position, pose, etc.), the hierarchical nature of error structures within this nested image data set was leveraged by recursively applying the proposed algorithmic pipeline for creating an bias correctio term for errors in image annotation. This was conducted by recalculating residual estimates between observed biometrics measurements and BLUP estimates calculated at the level of side of the face nested within cow, here using a fully nested fixed effects model that also contained the bias correction term for errors in image attributes. Data Mechanics clustering was again applied to the resulting scaled residual matrix, with the coarsest encoding capable of capturing all heterogeneity in error values again used to create a discretely encoded variable that would serve as a bias correction term for image attributes.

Fully nested mixed effects models were then refit to each individual biometric using both bias correction terms. From these regression results, the overall repeatability of each biometric was recalculated, as in the previous methods section. Additionally, the *piecewiseSEM* package was again used to calculate the marginal R^2^, in order to estimate the total proportion of variance in each observed biometric that was attributed to bias correction terms and subsequently and thus excluded from the final BLUP estimates [41]. Results are provided for all biometrics from either measurement system, with bootstrapped confidence intervals, in Supplemental Materials. To compere measurement systems, the *ggplot2* package was used to plot the overall repeatability values for each biometrics before and after bias correction for each anatomical subregion of the face, wherein the color of each data point was used to reflect the proportion of total variance attributed to systematic bias in the observed estimate [40]. 

## 3. Results and Discussion

### 3.1. Metric Repeatability

**Figure 3:**
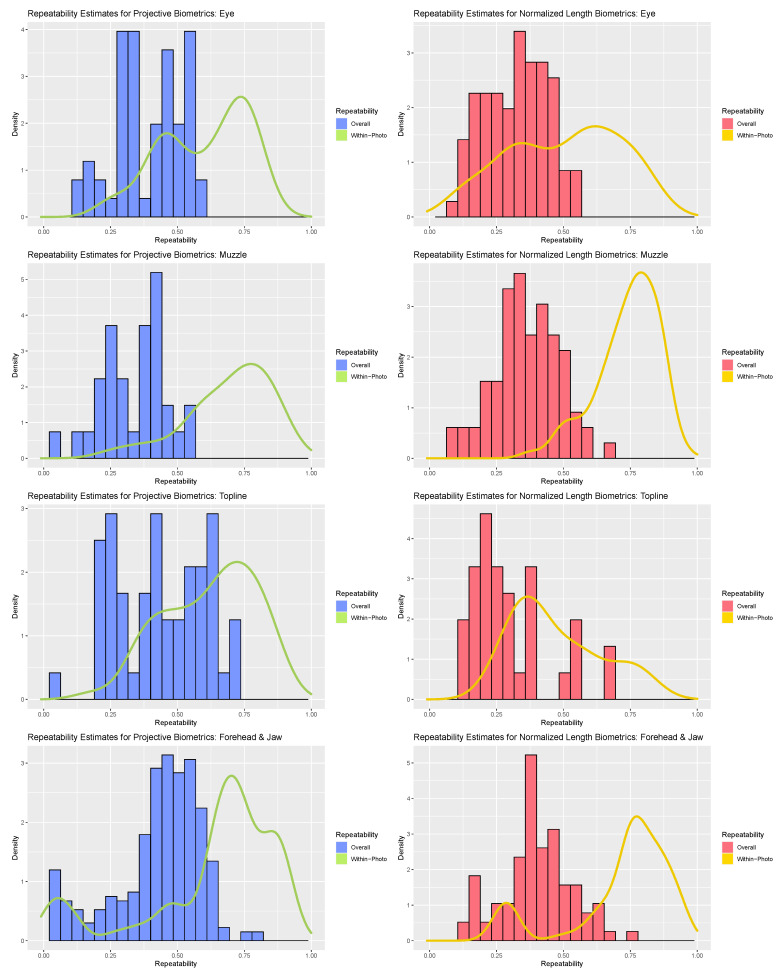
Comparison of within-photo repeatability (density plot) and overall repeatability (histogram) for normalized length biometrics (red) and projective biometrics (blue) for the four anatomical subregions (from top: eye, muzzle, topline, forehead).

Perhaps the most striking result of the repeatability analyses was the range of values that individual biometrics assumed across both measurement systems (see Figure 3). The majority of within-photo repeatability estimates fell in the good (>0.75) to moderate (>0.5) ranges, but some biometrics did demonstrate poor within-photo repeatability (<0.5). Given the diminutive scale of the finer facial features relative to the resolution of the camera used, it is not surprising that uncertainty in landmark annotations would represent a non-negligible source of error for this use case, but individual metrics with poor within-photo repeatability warranted further scrutiny. For the forehead and jaw subregion, poor within-photo repeatability values appeared to be linked to a subset of landmark coordinates around the poll (‘T_Slope’) and eye orbital (‘CAN’) that may have been partially obscured by hair on animals with thicker coats. For both the eye and topline subregions, poor within-photo repeatability largely corresponded to landmark points that were not defined at the intersection of two anatomical edges (e.g., where upper and lower eyelids meet) but were instead defined as the point along a smooth anatomical edge that achieved maximal displacement from an anatomical reference line (e.g., highest point of upper eyelid). This was especially true for features where displacement of a feature from its baseline was not significant for all animals (e.g., cows with a straight/flat nose). Normalized length biometrics may have been slightly more impacted by such issues in point annotation, especially for topline traits, where target features were relatively flat and thus the position of the landmark relative the length of the reference line was more uncertain than the distance of displacement. While such issues may be avoided in less ambitious annotation schemes, or in use cases with larger and more distinct features, these results underscore the importance of preliminary validation work, particularly for measures that rely on landmarks defined by relative displacement.

Contrasting the distribution of overall repeatability estimates against the density curves for within-photo repeatability revealed clear and consistent loss in measurement precision with the inclusion of variables that could not be held constant between photos. As a result, “single shot” repeatability for biometrics produced from both measurement systems generally ranged from moderate to poor. The fleshier muzzle and eye subregions were perhaps the most severely impacted, with relatively few biometrics demonstrating overall repeatability estimates above 0.5 with either measurement strategy. The muzzle subregion proved particularly susceptible to the impact of between-photo error, given its comparably strong within-photo repeatability values. As muzzle biometrics largely measured fleshier cartilaginous features—nostrils, lips, chin—this result could indicate that these biometrics were influenced by between-day changes in facial expression (i.e., animal pose). Alternatively, as the muzzle is relatively far away from both the poll (the axis of movement of the skull) and the boney eye orbital (the anatomical reference used here to control for image angle) this result could also be attributed to out-of-plane changes in facial angle. The bonier forehead and topline subregions fared somewhat better, with the impact of between-day error somewhat attenuated in comparison and some biometrics even achieving good overall repeatability estimates exceeding 0.75. For these subregions, projective biometrics demonstrated a slight upward shift in density relative to normalized length metrics, especially amongst the topline subregion.

When biometrics suffering from readily identifiable shortcomings in annotation protocols were excluded from consideration, both measurement systems produced repeatability estimates comparable to previous work on scale free measurements in livestock. Kristjansson et al. (2013) and Gmel et al. (2018) reported within-photo repeatability ranging from roughly 0.5–0.98 in their image-based measurements of leg angles in horses [17,18]. While the upper bound for within-photo repeatability for facial biometrics was slightly lower for projective and normalized length biometrics, the lower bound was comparable for all anatomical subregions but the eye. This is not necessarily surprising given the difference in scale of the morphological traits considered [17,18]. Kristjansson et al. (2013) and Gmel et al. (2018) also reported a wider range of between-photo repeatability estimates from as high as 0.96 down to 0.3 [17,18]. Again, the upper bound for overall repeatability of facial biometrics was lower for both measurement systems, with the boney topline and forehead subregions achieving a similar lower bound, but the fleshier subregions trended even lower. Stock et al. (2017, 2018) reported a narrower range of between-photo repeatability values for leg angles in sows from 0.63 to 0.82, which might reflect the impact of the relative mobility of measurement targets on single pass repeatability, but may also have been influenced by the inclusion of farm and parity as fixed effects in the models used to estimate variance terms [15,16].

Overall, the results of the repeatability analysis reveal that both measurement systems were capable of producing morphometrics with adequate or even good repeatabilities from one annotation of a single photo, but that such biometrics were ultimately in the minority of candidate metrics underscore the importance of preliminary measurement validation. For metrics where variations in the underlying static morphology was partially obscured by fleshier features, multiple images would be necessary to produce reasonably precise measurements. In this use case featuring morphological traits that were quite small in scale, the repeatability of all measurements could have been improved through replication of the landmark annotations—a strategy that could also be employed to bolster measurement precision in use cases of non-replicable transient features, such as facial expression.

### 3.2. Resilience to Changes in Image Attributes

**Figure 4:**
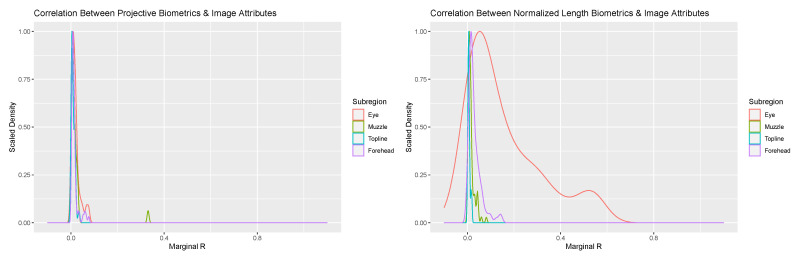
Scaled density plots representing the proportion of variability in normalized length (**right**) and projective biometrics (**left**) additively associated with Frame:Face Ratio and Face Angle for the four anatomical subregions of the face.

Addition of image attribute variables related to image scale and orientation to the mixed modeling equations provided additional insights into the potential sources of between-day error. Overall, the proportion of variance attributed to these image attribute values were negligible for the overwhelming majority of biometrics for both measurement systems (see Figure 4). Amongst projective biometrics, only a single metric produced a marginal R value of any note: Nostril Flare Point Proportion—Lower Front. Closer assessment revealed a strong association between this metric and facial angle, which in the images appears to be the result of tightening of the nostrils associated with snorting amongst a subset of cows that pulled back in the headlock in response to being photographed. That no projective biometrics showed strong correlation to Frame:Face Ratio suggests that the localized normalization technique these measures utilized to produce a scale-free metric was sufficient to adjust for between-photo changes in image scale.

For normalized length biometrics the majority of marginal R values were again trivial, with some notable exceptions. A subset of metrics in the forehead and jaw anatomical subregion produced marginal R values in excess of 0.1. Closer inspection revealed that annotations of the cranial-most point along the jaw (point ‘M’) were influenced by abduction of the head towards the chest, with contraction of musculature at attachments along the jaw altering how far back from the chin the jaw bone could be visualized, thus leading to a significant association with Overall Facial Angle. This issue appears to have been attenuated amongst projective biometrics, as the position and angle the line of the jaw itself, onto which interpolated points were projected, was not affected by these head movements. This result underscores that, where it can be anticipated a priori, the impacts of uncontrolled variations in animal pose can be at least partially avoided through careful design of projective biometrics.

Of perhaps greater concern, however, were the magnitude of correlations between image attributes and a notable proportion of normalized length metrics for the eye. Again, coefficient estimates revealed that Overall Facial Angle and not Frame:Face Ratio drove these association, and so the efficacy of the global scaling strategy employed by normalized length measures was not called into question. While it is possible that in-plane variations in facial angle (pitch) could be correlated to out-of-plane changes in head position (roll and yaw), it seems unlikely that the subsequent projection errors would have been seen only in the eye and for no other anatomical subregions. Closer inspection revealed the highest magnitudes of correlation were generally found between landmark annotations along the outer eyelid, and that annotations along the line of boney eye orbital generally produced the lowest marginal R estimates. This suggests that movement of the fleshy features of the eye between photos may account for these associations. It is possible that reactions to the photographer might have elicited synonymous changes in head position and facial expression. Changes in the eyelid could also have simply been compensatory, allowing the cow to maintain visual focus on the camera person with changes in head position. Visual inspection of the raw images did not ultimately provide clear evidence for one explanation over the other, underscoring the difficulty in interpreting normalized length metrics. That such changes were not also captured in the projective length biometrics is likely attributable to their informationally incomplete nature. For landmark points of the eye annotated between each pair of adjacent latitudinal and longitudinal corners, projective biometrics only included information on the displacement from this anatomical reference line, whereas informationally complete normalized length measures would have been affected by both displacement and the position of these points along the length of their corresponding reference lines. Overall, this result emphasizes that, while the normalization schemes employed by both scale-free morphometrics systems appear resilient to variation in distance between animal and camera, imaging protocols should still consider that the animals themselves may still be influenced by camera position.

### 3.3. Error Structure Analysis

**Figure 5:**
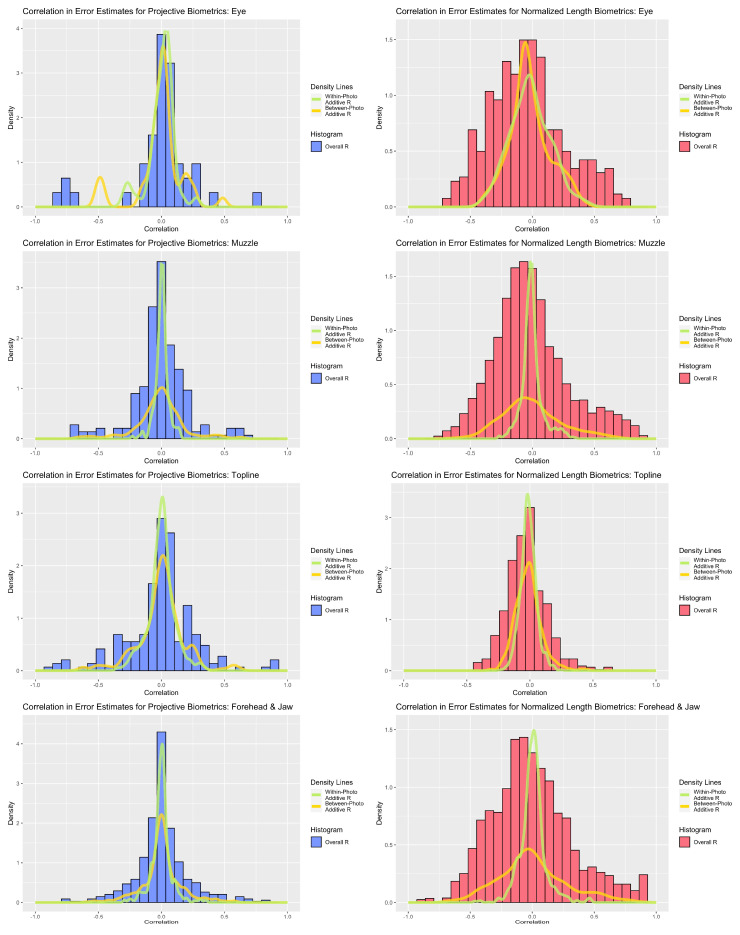
Comparison of correlations between overall, between-photo, and within-photo error estimates for normalized length (red) and projective biometrics (blue) for the four anatomical subregions (from top: eye, muzzle, topline, forehead).

Correlation analyses revealed strong associations amongst residual estimates for both measurement systems (see Figure 5). Between-photo error contributed greater additive correlation to the overall error correlation estimates for the forehead and muzzle subregions, a distinction that was strongest amongst normalized length metrics. For the eye and topline subregions, within- and between-photo error were more balanced in their contributions of additive error correlation. With respect to overall error correlation, projective biometrics tended to produce proportionally fewer correlations of high magnitude, as evidenced by thinner histogram tails, than normalized length biometrics. This disparity was the most apparent for muzzle and forehead biometrics. Amongst topline and eye biometrics, a small subset of projective biometrics produced error correlations of higher magnitude than with normalized length; however, on closer inspection, these results were largely attributed to linear redundancy in the derivations of length measures estimated along the same anatomical reference lines using concurrent landmarks, which could be readily avoided though structured subsetting of such metrics without loss of information. 

**Figure 6:**
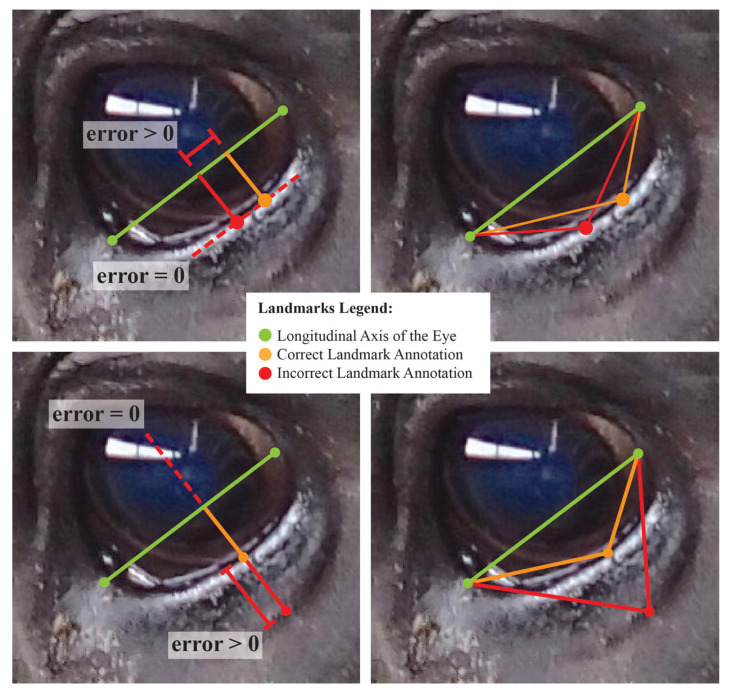
A visualization of changes in normalized length (**right**) and projective biometrics (**left**) with errors in point annotation along the curve of the lower eye. Top photos represent lateral uncertainty in annotation of the maximal point deviation of the curve from the baseline. Bottom photos represent uncertainty in the curve used to place landmark points. In this exaggerated example, these errors in point annotation are isolated to distinct orthogonal projections, but result in synergistic tradeoffs in the Euclidean distances used in calculating normalized length biometrics.

In comparing the performance of normalized length and projective biometrics with respect to within-photo error correlations, little advantage is seen for any anatomical subregion save for the eye, where normalized length measures again appear to generate proportionately higher magnitude correlations. This result is perhaps partially attributable to differences in how roundness, the most pervasive anatomical feature for the eye, is geometrically encoded by these two measurement systems. For projective biometrics, the displacement of a curve from a baseline (roundness proportion) and the relative position of that maxima point along that baseline (roundness point proportion) form the arms of a right triangle, and thus are geometrically orthogonal quantities, which in turn aids in orthogonalizing errors in landmark annotations. Normalized length metrics, on the other hand, can be visualized as the hypotenuses of the same triangles, the lengths of which are simultaneously influenced by errors in point annotation in both the x- and y-directions relative the base, resulting in more diffuse error correlation structures among within-photo errors (see Figure 6). That this effect is seen so strongly for the eye Is perhaps attributable to the “stacked” nature of eye annotations. While the latitudinal corners of the eye were determined by the intersection of the eyelids, the longitudinal corners were defined as the point of maximal deviation from this baseline. The intermediate eye points were then added at the point of maximal deviation from the anatomical reference lines defined between each set of adjacent corners. Thus, errors in annotating the highest and lowest point of the eye would have been propagated to the annotations of these intermediate eye points by biasing the reference line, creating structural dependencies in the annotation errors of the resulting biometrics.

Differences in the resilience to between-photo error correlation demonstrated by these two measurement systems were less consistent. Thicker tails are observed on the distribution of additive between-photo correlation for normalized length biometrics in the forehead and muzzle subregions, but projective biometrics demonstrate slightly thicker tails with eye and topline biometrics. Multiple sources of error may be contributing to such error structures. For anatomical subregions with fleshier traits, namely the eye and muzzle metrics, changes in facial expression between photos may partially account for correlations amongst error terms. As contractions of facial muscles cause some length measures to shorten, others must grow longer in compensation, creating systematic error structures that would be consistent across cows by virtue of their shared muscular anatomy [6]. That changes in the environment between photographs might elicit a response in facial musculature that would produce systematic changes in the annotations of anatomical landmarks in the fleshier regions of the face is not really a surprising result. Nor is the magnitude of these correlations, where they were uncovered, without precedence. In their analysis of equine conformation using geometric morphometrics, Druml et al. (2015) found that the 1st and 4th principal axes of their Lippizan shape space did not describe variations in confirmation, but were instead attributed to variation in neck and leg posture, respectively, accounting together for roughly 50% of total shape variability [21]. In a follow-up study, Gmel et al. (2018) confirmed that a number of subjective scores of horse posture, when added to mixed effect models of these shape space responses, produced marginal R values in excess of 0.1 [22]. Thus, these results only provide further evidence that uncontrolled variations in animal pose can serve as nontrivial sources of measurement bias for scale-free anatomical morphometrics. 

**Figure 7:**
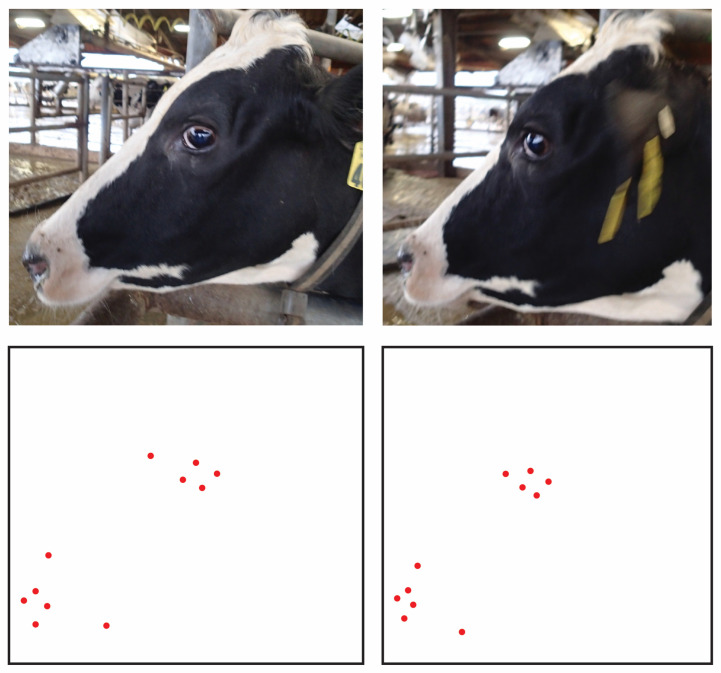
Demonstration of the impact out-of-plane variations in facial angle can have on the relative locations of anatomical landmark points. The change in angle between the 3D object and the 2D plane of the camera on which the image is being rendered can be described by homographic transforms. This geometric operation results in systematic changes in the relative position of landmark points, which would in turn produce correlations in between-photo error terms.

Nontrivial between-photo correlations were also found amongst topline metrics, however, which consisted exclusively of boney traits, and so changes in expression cannot be the only source of such error correlation. Changes in out-of-plane angles of the face (head posture) here become suspect. With any changes in roll and yaw of the head along the axis of the poll between images, projection of the anatomical points on the 3D dimensional surface of the skull onto the 2D plane of the image will be warped. Indeed, because these points all lie on the same rigid surface of the skull that is similar in overall configuration across animals, such movements will induce systematic transformations in their relative coordinate locations, and thus in turn the coordinate-free distance measures computed from them [43,53]. A visual representation of this effect is provided in Figure 7. While a priori knowledge of muscular anatomy might be used to derive projective biometrics resistant to error correlations due to changes in expression, provided that the shape of a feature is not simultaneously influenced by multiple muscular attachments contracting in different directions, neither measurement system could be expected to the accommodate the complex and nonlinear (nonaffine) changes in the projections of landmark coordinates onto the plane of the camera with changes in position of the skull. This results therefor confirms that, for use cases where out-of-plane deviations in camera angle cannot be controlled in the acquisition of images of unrestrained animals, camera position may still serve as a source of systematic bias for either approach to scale-free measurement.

Regardless of their ultimate cause, high magnitudes of correlations between biometric error terms are cause for statistical concern. When biometrics are added to the right-hand side of the model equation as predictor variables, high magnitude error correlations could enhance overall correlations between covariates, which could in turn cause variance inflation and model instability. Of perhaps greater concern, however, would be scenarios where multiple biometrics are added to the left-hand side of the equation as a multivariate response. This might occur if such biometrics were incorporated into genetic analyses or simply in multivariate analyses attempting to utilize correlations between biometrics to improve measurement precision and thus carry out more robust tests of model hypotheses. Here, the standard *iid* error assumption would not necessarily be met for all potential sets of biometrics [54]. Indeed, if the magnitude of additive correlation attributed to error is large relative to that of the underlying anatomical signal linking two biometrics, failure to incorporate an off-diagonal term to the error model would only serve to concentrate and magnify the effects of lurking (confounder) variables, potentially leading to misleading statistical inferences. The addition of non-zero error covariate terms to the error model, however, would greatly increase the number of parameters to be estimated in the resulting model and thus greatly increase the requisite sample size [54]. If the factors varying between images (camera position, pose, etc.) could be directly quantified, these sources of systematic heterogeneity in both measurement systems could be accounted for using far smaller sample sizes, but in applications with minimally restrained animals the imaging protocol would be made significantly more complicated in an effort to streamline statistical analyses.

### 3.4. Reliability of Dimension Reduction

**Figure 8:**
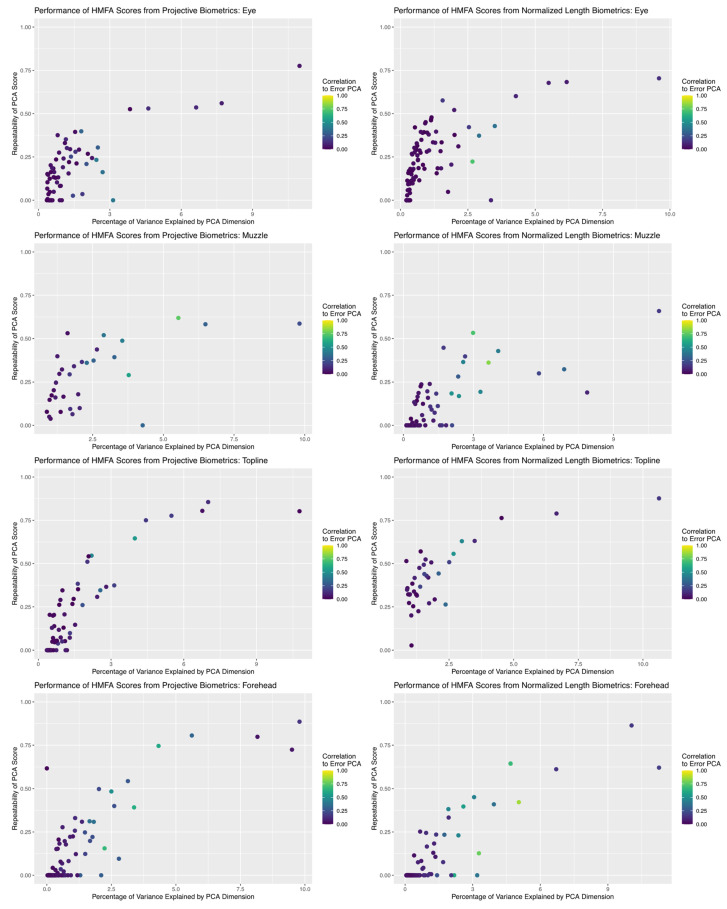
Comparison of the relationship between the proportion of total variance explained by each dimension returned by HMFA analysis and the repeatability estimate of the partial scores. For both measurement systems, basis dimensions with intermediate repeatability estimates align with the subspaces generated for systematic error structures, which may indicate they are concentrating variance attributable to lurking variables.

Results of dimension reduction analysis revealed that HMFA did succeed in producing aggregate scores of facial morphology with good repeatability estimates for both measurement systems (see Figure 8). In comparing the repeatability results across anatomical subregions, the maximum repeatability values achieved were fairly consistent between the two measurement systems, but for the muzzle and forehead subregions the repeatability values of projective biometrics decayed perhaps marginally slower than normalized length measures as the percentage of total variability explained by the corresponding basis dimension decreased. For both measurement systems, individual basis dimensions explained relatively small proportions of the total measurement variance. This result is not entirely unexpected, given the number of candidate biometric values with moderate to poor repeatability estimates and thus by extension little anatomical signal; however, it is also possible that relationships between the morphological features of the face are not well approximated by an inherently low-dimensional linear representation, either because they do not share strong biological associations or because their relationships are nonlinear in character.

One noteworthy pattern observed in both measurement systems across all anatomical subregions is that, while the first few basis dimensions produced by the HMFA analysis yielded scores with both good repeatability and little correlation to the subspace estimated for the error structure, there are many dimensions with intermediate repeatability values that are quite close to the error subspace. This may suggest that, while the first few bases are successfully isolating the underlying anatomical signal from the measurement noise, these intermediate values may also be concentrating systematic error structures alongside these more moderate anatomical inter-relationships. This raises methodological concerns, as most of these intermediate dimensions with acceptable repeatability estimates would be retained for further statistical analysis when basis dimension is determined by visual inspection of the corresponding scree plots (provided in supplemental materials).

The concentration of systematic error structures due to changes in facial expression or camera angle in such HMFA scores would only increase the risk that these lurking variables might lead to misleading inferences [43]. For example, if one of these scores concentrated error correlation structures due to nonaffine changes in facial angle across multiple observed metrics, and if variations in camera angle between photos were not entirely random—camera persons consistently favored slightly different camera angles, variations in chute layout between farms made some camera angles more accessible than others, etc.—this could enhance lurking variables related to farm and scorer effects in any subsequent statistical models. Alternatively, if, for example, fearful cows tend to assume a different head position than more even-tempered animals, and if such dimension reduction techniques concentrated correlated error structures from the resulting errors from projection of the 3D structures onto the plane of the camera, then genetic selection using image based measures of these morphometric features might carry with in unintended selection pressure for behavioral responses of cows to imaging. Given the difficulty of interpreting any dimension reduction on high dimensional systems, such issues with confounding variables might easily go unnoticed.

Thus, these results suggest that, while dimension reduction techniques are a valid approach for both measurement systems, future studies employing this step in analytical pipelines with such scale-free metrics should exercise additional caution in selecting the appropriate number of dimensionally reduced features, and should ideally utilize a repeated measures design on at least a subset of sampled animals in order to facilitate comparisons of aggregate scores from observed measures against aggregate scores from the error subspace. For use cases with transient features where true replication is not possible, such as measures of facial expression, it may be advisable to extract supplemental measures of static skull morphology on a subset of animals to carry out similar validation procedures to identify potential lurking variables. 

### 3.5. Reliability of Bias Corrected Biometrics

**Figure 9:**
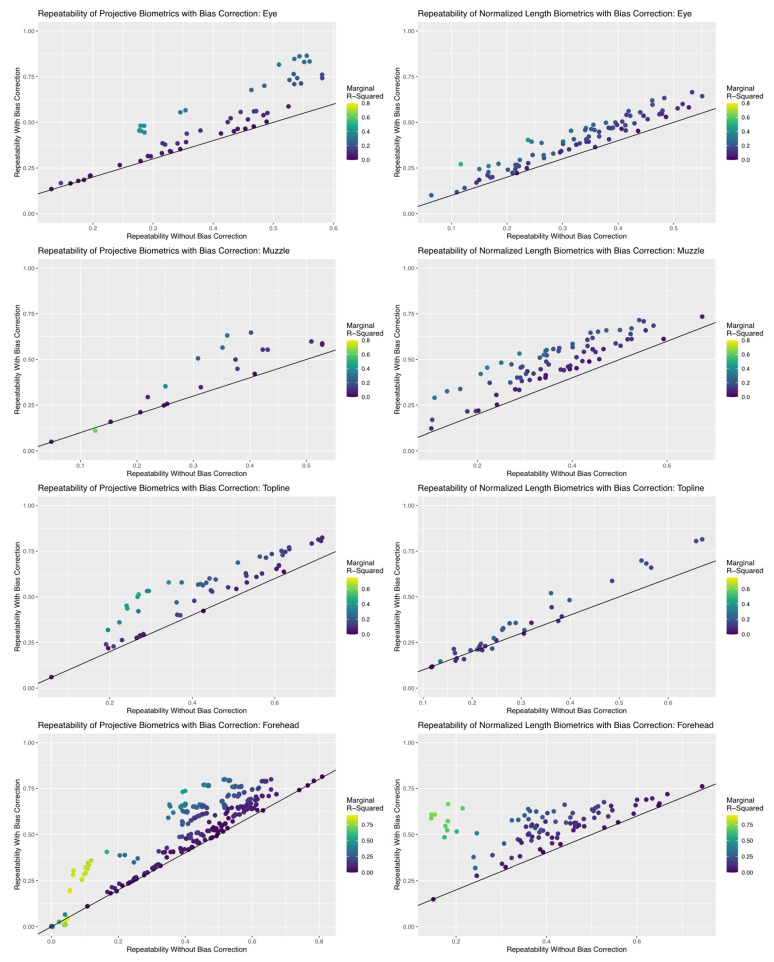
Comparison in the repeatability estimates for each biometric calculated with and without bias correction terms. The black line super-imposed on each scatterplot represents no change in repeatability estimates, such that all points that fall above this line represent an improvement in the reliability of the biometric. Each biometric point is colored by their corresponding marginal R^2^ estimate, which represents the total proportion of observed variance in the biometric attributed to the bias correction terms. An appreciable proportion of biometrics show improvements in repeatability with inclusion of these terms for both measurement systems for all anatomical subregions, but the performance of projective biometrics was more strongly impacted for the eye and forehead subregions.

Results of the repeatability analyses, summarized in Figure 9, revealed that the inclusion of bias correction terms created using simple UML clustering tools succeeded in improving the reliability of an appreciable proportion of biometrics in both measurement systems, but efficacy of bias correction did differ between anatomical subregions. The proportion of total (marginal) variance attributed to encodings of systematic differences in residual estimates terms was low to moderate (<0.50) for both measurement systems for the topline and eye subregions. As a result, repeatability estimates for biometrics in this subregion were improved between 0 and 20%, which increased the proportion of metrics with acceptable repeatability (>0.50), but few biometrics achieved good repeatability values (>0.75). This result is not necessarily surprising given the results of the previous analyses of error correlation structures, where overall residual correlation estimates were relatively lower for these two anatomical subregions, which suggests that the algorithmic pipeline proposed here for bias correction can be effective in applications where systematic correlation structures may be more are subtle.

Bias correction terms had a far greater impact on repeatability estimates for the eye and forehead subregions. The proportion of total (marginal) variance attributed to encodings of systematic error structures here occupied a much wider range of estimates, being as high as 0.85 for some biometrics. This result is not surprising, given that previous analyses of error structures recovered higher magnitude correlations for both these anatomical subregions, but it does confirm that these biometrics may be significantly biased by lurking variables related to annotation and imaging protocols. Addition of bias correction terms improved repeatability estimates of a notable proportion of biometrics within these anatomical subregions—some more than 20%—so that a greater proportion of biometrics in either measurement system could be said to exhibit acceptable (>0.50) or even good (>0.75) repeatability values. These improvements were not, however, as uniform as in the topline and muzzle subregions, which warranted further examination.

**Figure 10:**
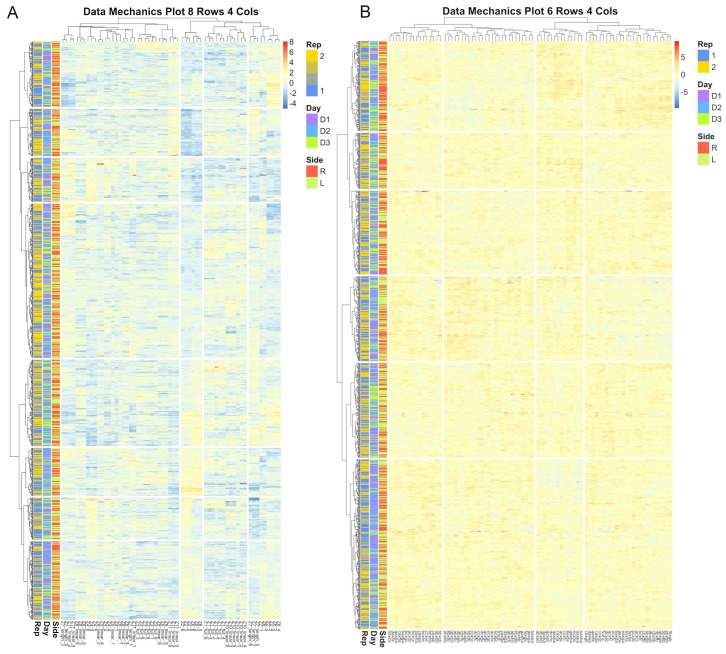
Heatmap visualizations of data mechanics clustering results for between image error for the eye anatomical subregion. Each row represents an image and each column represent a candidate biometric. Each cell is colored to represent the scaled residual estimate for a given image for a given biometric. Results for the projective biometrics (**A**) not only show greater variability in residual estimates than for normalized length biometrics (**B**), but there is considerably greater systematic heterogeneity captured by the clustering algorithm, which is visualized as distinctive color patters between clusters as visualized both across the rows and columns of the residual matrix.

Improvements in the repeatability estimates for eye biometrics were notably higher for projective biometrics than for normalized length metrics. Comparison of the encoding granularities used to create annotation and image error bias correction terms, which are presented in Table 1, reveal that data mechanics algorithms were for this anatomical subregion able to recover greater systematic heterogeneity in residual estimates for projective biometrics, which would readily explain the differences in the efficacy of bias correction terms. Heatmap visualizations comparing the clustering results for the image error encoding are provided in Figure 10. As normalized length biometrics were previously shown to be disproportionately impacted by within-image error, one interpretation of this result might then be that that systematic error in image annotation were here occluded by nested errors in annotation—errors that do not appear to have been fully captured by the encoding of residual annotation error, likely due to the nonlinear (trigonometric) relationships that have already been discussed for anatomical reference points that are defined as the maximal point of deviation from an anatomical reference line.

Responses to bias correction also differed between the two measurement systems for forehead biometrics. While coarser encodings were here recovered for projective biometrics by clustering analyses when compared with the results for normalized length (see Table 1), improvements in metric repeatability following inclusion of the bias correction term were arguably better for projective length, which saw repeatability estimates improved to acceptable (>0.50) or even good (0.75) for a notably larger proportion of metrics. Interestingly, the greatest improvements in normalized length metrics were seen amongst metrics for which the majority (>0.50) of the total variance in the observed measurement was attributed to bias correction terms, as determined by marginal R^2^ values. That the observed values of these biometrics are so heavily influenced by systematic error induced by lurking variables suggests that, for applications where imaging cannot be replicated, these metrics might even be directly employed as bias correction terms using a similar encoding pipeline to allow for nonlinearity in the latent link function. For example, in applications with transient facial expression, repeated images of the target features of analysis might not be easily obtained, but if multiple images are acquired from each animals, structural morphometrics sensitive to variation in camera position might be extracted and this UML pipeline applied to create bias correction terms for projection error, allowing researcher to apply minimally invasive imaging protocols while still minimizing the risk of measurement bias. 

While differences in the performance of bias correction terms between anatomical subregions here seems most easily explained by the efficacy of the UML pipeline to recover latent error structures, this information must also be passed to the mixed effect model through the fixed effects terms in order to influence the final estimation of the underlying morphometrics. By utilizing a clustering-based approach, nonlinearity in the latent link function connecting the observed measurements with the one or more sources of systematic error in this system could be accommodated without stating a specific model. Such coarsening of the data, however, may place practical limitations on the acuity of the bias correction model. In these analyses, clustering granularity was selected subjectively to capture all systematic heterogeneity that was visually distinguishable by heatmap. Finer encodings might be used to further minimize loss of information, with any over-fitting of the bias correction terms ultimately only translating to more conservative estimates of random effects. Future work, however, might also consider applications of nonlinear manifold embedding approaches in this bias correction pipeline to create continuous linearized estimates of latent lurking variables [5]. While such approaches have been used in a range of image analysis applications, it should be noted that these information compression techniques can be prone to imposing harmonic artifacts into embeddings of data sets that are not well sampled across the entire domain, and that such artifacts are not always easily distinguished from systematic structures in the data [5,52,55]. Subsequently, embedding-based approaches may be more appropriate where the image data set can be augmented with more systematic sampling of factors known to impose biases, such as camera position [5,55]. For data sets lacking this structured sampling, however, a clustering based approach may offer a better tradeoff between the performance and the reliability of the bias correction pipeline. For applications that require biometric measurements of the highest accuracy, the method and modality of image acquisition is still likely to provide greater payoffs in measurement performance than these downstream algorithmic remediation techniques, regardless of the UML techniques utilized in this bias correction pipeline.

## 4. Conclusions

The results of these analyses show that both normalized length and projective biometrics are resilient to fluctuations in image scale and are capable of extracting morphometric measurements from uncalibrated 2D images with moderate to good repeatability. For applications that require high accuracy measurements of finer scale features, replication of image annotations may be needed, particularly for landmarks identified by their relative displacement from anatomical reference lines. Both measurement systems, however, were susceptible to systematic bias induced by variations in lurking variables that cannot be held constant between images. We have shown that such systematic error can lead astray standard multivariate approaches to dimension reduction, which could in turn increase the risk that statistical inferences gleaned from such datasets could become confounded with lurking variables. To avoid risk of spurious inference, without increasing the complexity or invasiveness imaging protocol, additional care must be taken not only in the design of the biometrics but also the downstream analyses. We have demonstrated that simple and readily generalizable unsupervised learning tools are capable of working with, not against, the systematic error structures created in scale free biometrics to infer information about lurking variables. Encoded variables extracted from these clustering results can subsequently be incorporated into downstream analyses to control statistically for variables that cannot be controlled on farm, thereby helping to ensure that the insights and intuitions gleaned from these emerging PLF data streams can provide a reliable foundation for future work. While these new algorithmic techniques have been shown to improve the overall reliability of scale free morphometrics from unstructured data sets, future work should consider how structured systematic sampling techniques might be incorporated into image acquisition protocols to further improve the efficacy of these measurement systems. In particular, subsequent work might consider how this algorithmic pipeline might be expanded to incorporate targeted application of more costly and invasive 3D imaging techniques, in order to better overcome systematic biases from variations in camera position in applications with minimally restrained livestock that require less invasive 2D imaging tools. 

## Figures and Tables

**Table 1:** Summary of encodings granularities recovered using data mechanics clustering for projective biometrics and normalized length biometrics for both the annotation and image error bias correction factors. Complete results of data mechanics analyses are provided in Supplemental Materials.

	Projective Biometrics				Normalized Length Biometrics			
	Annotation Error Encoding		Image Error Encoding		Annotation Error Encoding		Image Error Encoding	
	Rows	Columns	Rows	Columns	Rows	Columns	Rows	Columns
Eye	8	5	8	4	7	3	6	4
Muzzle	7	4	7	4	8	4	8	5
Topline	7	4	8	4	7	4	7	4
Forehead	5	5	7	5	7	4	8	4

## Data Availability

The datasets generated for this study are available on request to the corresponding author.

## Supplementary Materials

The following supporting information can be downloaded at: https://www.mdpi.com/article/10.3390/s22218347/s1, Anatomical Reference Points; Landmarks Annotation Guide; Derivations of Projective Biometrics; Measurement System Analysis Code; Measurement System Analysis Results; Data Visualizations.
