# Supplementary material for: Improving the Reliability of Scale-Free Image Morphometrics in Applications with Minimally Restrained Livestock Using Projective Geometry and Unsupervised Machine Learning
Source: Sensors (Basel). 2022 Oct 31;22(21):8347. doi: 10.3390/s22218347 (PMC9653925; doi:10.3390/s22218347)
Supplement: Supplementary file 1 [file sensors-22-08347-s001.zip › SupplementalMaterials/Annotation Protocol.pdf]

Eye Annotation

Horizontal Plane of the Eye

1) Rostral-Most Point of the Eye (A)
   Select the rostral-most point where skin of the eyelid meets the eye. For some cows, the upper and lower eye will meet at a point. The annotation point should be selected in the crook of this cusp. For others, this area will be flatter. Attempt to visualize the triangle formed by the lacrimal caruncle, and annotate this point midway along its base (as shown below). If the mucus membrane is swollen – do not select this tissue, but follow the line of the eyelid

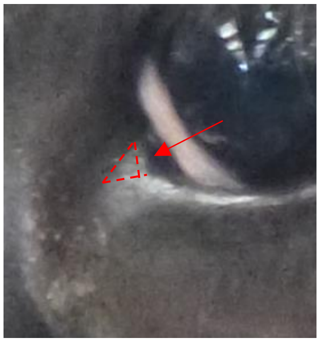

2) Caudal-Most Point of the Eye (B)
   Select the back-most point of the eye, where the eyelid meets the eye. For some cows, the eyelids will meet at a point, and the annotation should be placed in this crook. For others, the lateral angle of the eye will be flat. Attempt to visualize the triangle formed by the lids and place the annotation in the center of the base.

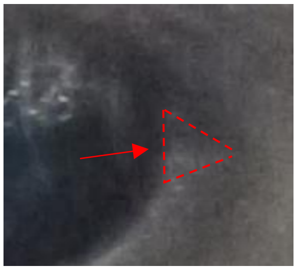

3) Canthus Point (CAN)
   Follow the indention of the canthus out until the skin again becomes level with the rest of the face. For some cows there will be a clear distinction between the smooth skin of the canthus and furred parts of the face, but in some cases it might be obscured by dried discharge form the eye, in which case just use your best judgment.

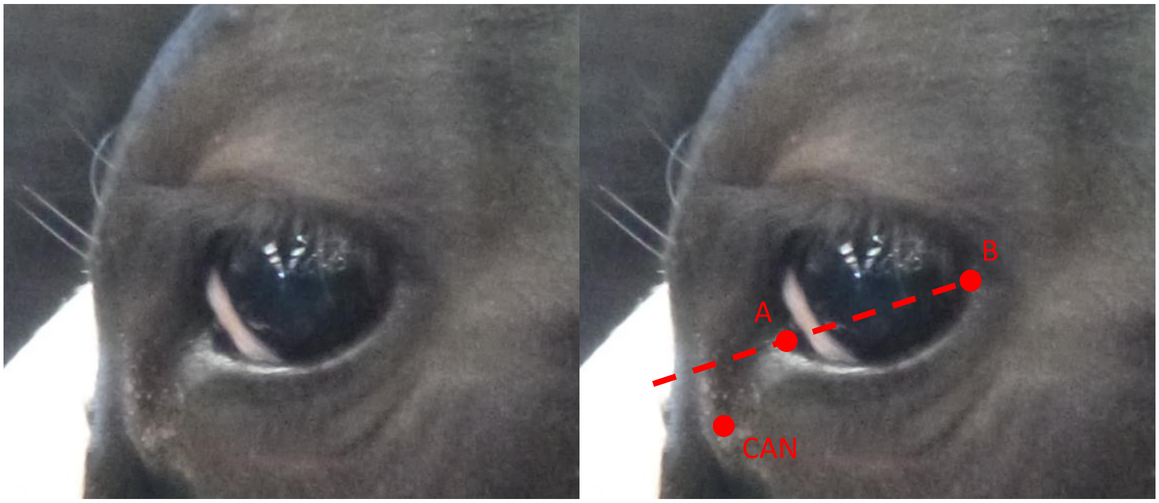

Inner Eye Lid Annotations

4) Highest Point of Inner Eyelid (C)
Select point along the upper eyelid, where it meets the eyeball, that attains maximal perpendicular distance from the horizontal line of the eye. If this point is obscured by the eyelashes, use your best judgement based on the shadows beneath the lashes.
5) Deepest Point of Inner Eyelid (D)
Select the point along the lower eye lid, where it meets the eyeball, that attains the maximal perpendicular distance from the horizontal line of the eye.
6) Eye Roundness Point – Upper Front (E)
Select the point of max perpendicular deviation of the inner eye lid, where it meets the eye ball, along the upper front line of the eye
7) Eye Roundness Point – Upper Back (F)
Select the point of max perpendicular deviation of the inner eye lid, where it meets the eye ball, along the upper back line of the eye
8) Eye Roundness Point – Lower Back (G)
Select the point of max perpendicular deviation of the inner eye lid, where it meets the eye ball, along the lower back line of the eye
9) Eye Roundness Point – Lower Front (H)
Select the point of max perpendicular deviation of the inner eye lid, where it meets the eye ball, along the lower front line of the eye. In some cows with particularly flat lacrimal caruncles, or with swollen mucus membranes (common in summer with flies), this point may sit practically on top of the rostral-most point of the eye. In this case, default to a point along the eyelid mid-way along lower front line of the eye.

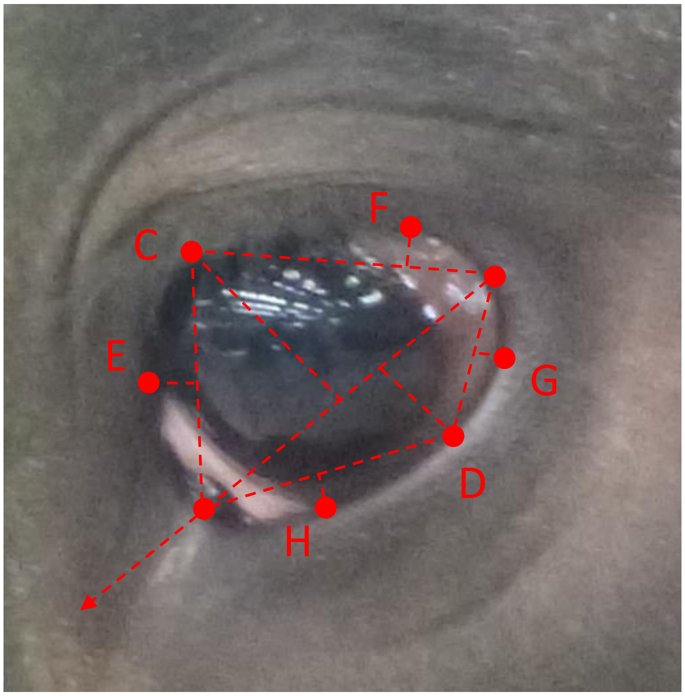

Outer Eye Lid Annotations

10) Highest Point of Inner Eyelid (C)
Select the point along the upper eyelid, where it meets the eyeball, that attains the maximal perpendicular distance from the horizontal line of the eye. If this point is obscured by the eyelashes, use your best judgement based on the shadows beneath the lashes.
11) Deepest Point of Inner Eyelid (D)
Select the point along the lower eye lid, where it meets the eyeball, that attains the maximal perpendicular distance from the horizontal line of the eye.
12) Eye Roundness Point – Upper Front (E)
Select the point of max perpendicular deviation of the inner eye lid, where it meets the eye ball, along the upper front line of the eye
13) Eye Roundness Point – Upper Back (F)
Select the point of max perpendicular deviation of the inner eye lid, where it meets the eye ball, along the upper back line of the eye
14) Eye Roundness Point – Lower Back (G)
Select the point of max perpendicular deviation of the inner eye lid, where it meets the eye ball, along the lower back line of the eye
15) Eye Roundness Point – Lower Front (H)
Select the point of max perpendicular deviation of the inner eye lid, where it meets the eye ball, along the lower front line of the eye. In some cows with particularly flat lacrimal caruncles, or with swollen mucus membranes (common in summer with flies), this point may sit practically on top of the rostral-most point of the eye. In this case, default to a point along the eyelid mid-way along lower front line of the eye.

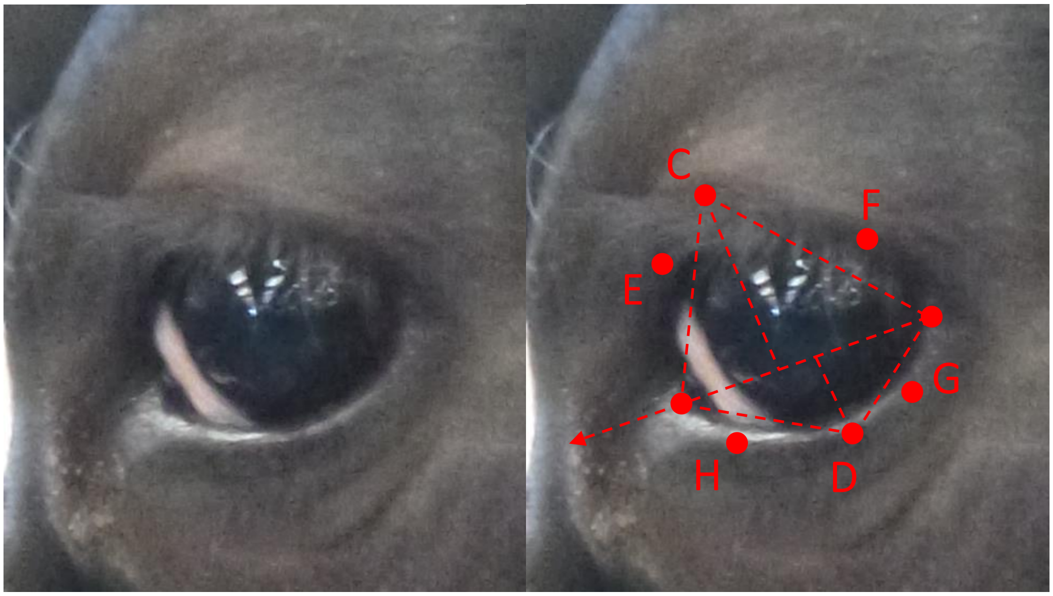

Eye Orbital Annotations

16) Highest Point of the Eye Orbital (C)
Follow the line formed by the upper eye lid crease, where the skin of the eyelid meets the ridge of the boney eye orbital creating a faint shadow. When the eye is held open, there should be a slight indent along this line from tension in the muscle, corresponding to the highest perpendicular point of the eye. Place the annotation at this indent.

17) Deepest Point of Inner Eyelid (D)
Starting at the back of the eye, follow the bottom line of the lower eye lid where it meets the bone of the eye orbital/check down to its deepest point. For some cows, there is a clear distinction between smooth fine skin of the eye lid and the hair of the face (particularly in the winter). When this is not the case, focus on following the shadow extending from the rostral-most point of the eye. Additional skin creases may be present below this line, but do not represent the line of the eye orbital and are not the target of annotation.

18) Eye Roundness Point – Upper Front (E)
Follow the line formed by the upper eye lid crease, select the point of max perpendicular deviation along the upper front line of the eye

19) Eye Roundness Point – Upper Back (F)
Follow the line formed by the upper eye lid crease, select the point of max perpendicular deviation along the upper back line of the eye

20) Eye Roundness Point – Lower Back (G)
Follow the line formed by the lower eye lid crease, select the point of max perpendicular deviation along the lower back line of the eye

21) Eye Roundness Point – Lower Front (H)
Follow the line formed by the lower eye lid crease, select the point of max perpendicular deviation along the lower front line of the eye

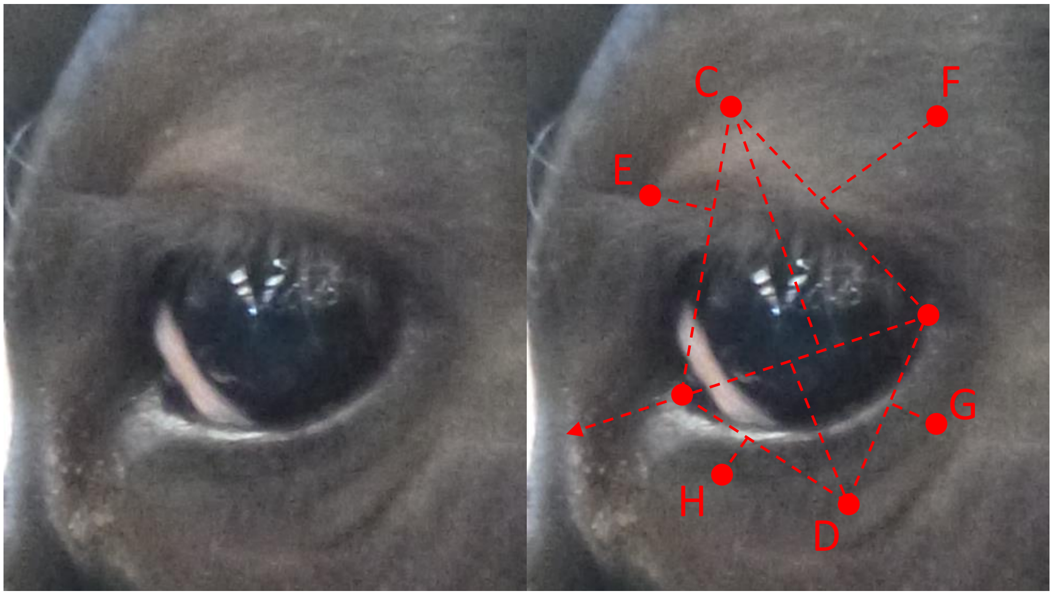

Forehead Annotation

<u>Eye Orbital Annotations</u>

1) Caudal-Most Point of Eye Orbital
   Select to point where the curve of the eye orbital flattens out to become the forehead. In some animals there will be a slight concavity where the eye orbital and flat portion of the frontal bone meet. A reference line has been added to approximate this location when image angle or hair obscures this point. There will often be an inflection in the curvature of the hair at this point, if image quality permits
2) Midpoint of Eye Orbital
   Select the point of greatest perpendicular displacement from the reference line of the eye orbital.

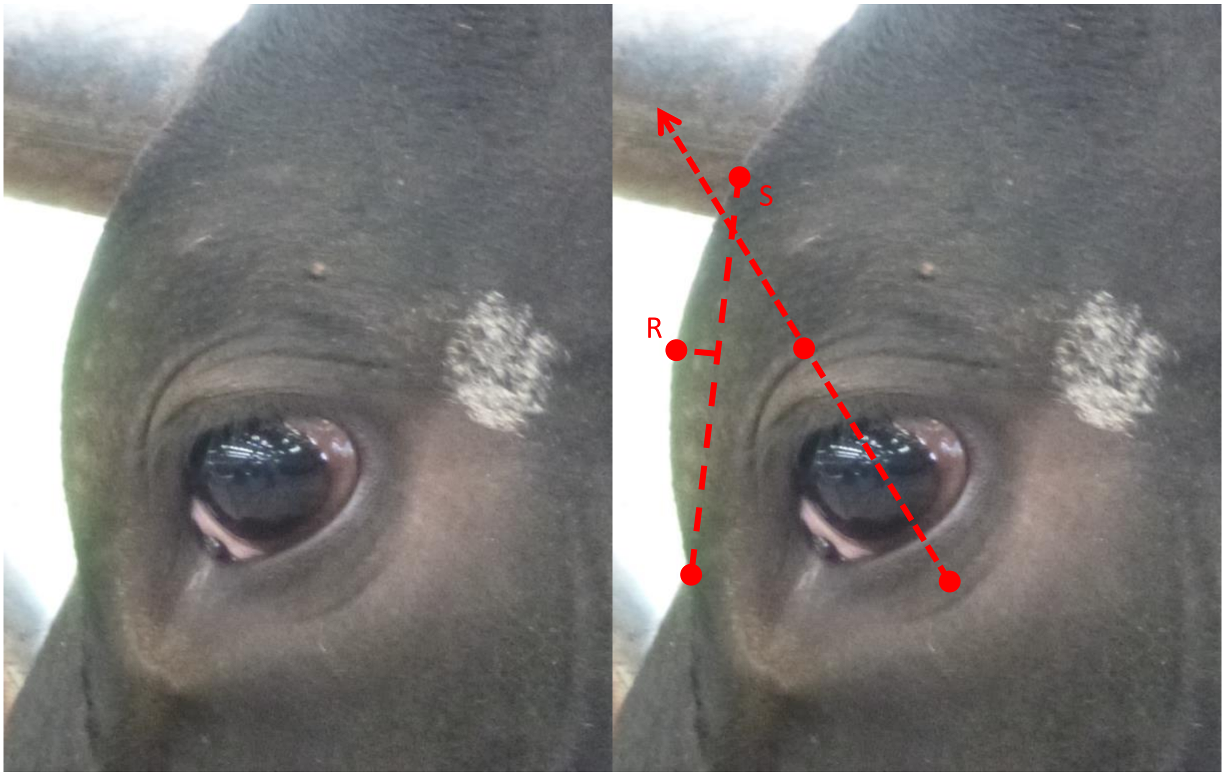

Forehead Annotation Points

3) Rostral-Most Base of Nuchal Crest (T_int)
   Select the point where the topline of the forehead intersects with the rostral most point of the base of the nuchal crease.
4) Lateral Base of the Nuchal Crest (T_slope)
   Select the point where the lateral edge of the frontal bone (extending from the eye orbital) intersects with the base of the nuchal crest. This will is typically easily visualized as the peak of an inverted corner in the hairline of the poll.
5) Caudal Point of the Nuchal Crest (T_poll)
   Select the point where the plateau created by the top of the nuchal crest inverts down towards the base of the neck. For some cows this will be quite square and present a clear inflection point. With others there may be a sloping indent – place the annotation point in the center of this indent
6) Cuadal-Most Base of Nuchal Crest (T_back)
   Select the point where the boney base of the nuchal crest appears to inserts into the back of the skull/muscle of the neck.
7) Apex of the Nuchal Crest (U)
   Select the point of maximal perpendicular deviation of the outline of the nuchal crest from the reference line of the forehead

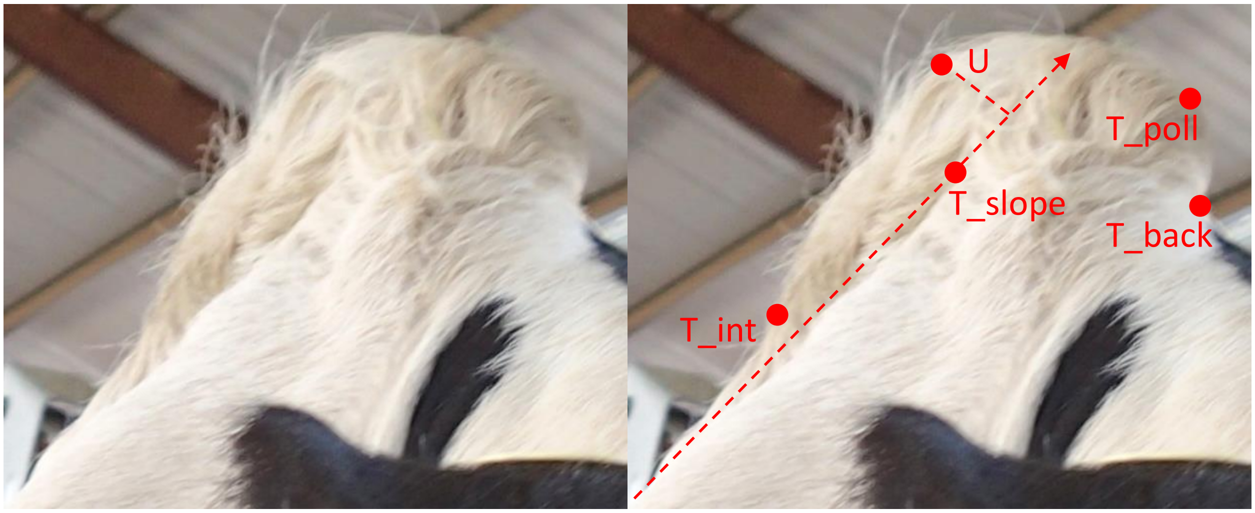

Jawline Annotation Points

8) Insertion Point of the Jaw (M)
Select the point where the jaw muscle inserts into back onto the jaw bone. This will appear on most cows as a clear inflection point along the jaw before the skin fleshes out into the neck. If a clear insertion point cannot be seen, follow the line of the jaw bone back as far as can be seen and select this point
9) Point of Maximal Jaw Thickness (P)
Select the point of maximal perpendicular deviation of the jaw line from the reference line of the jaw. Note that some cows will have excess skin under their face, but the annotation point should fall on the lower profile of the jaw line/muscle

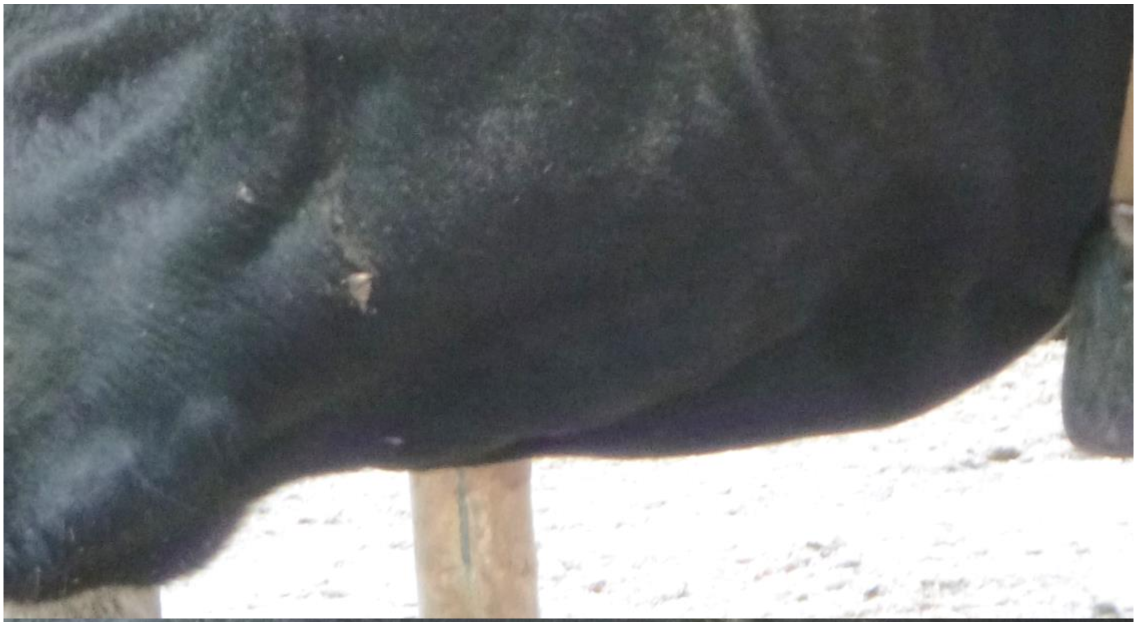

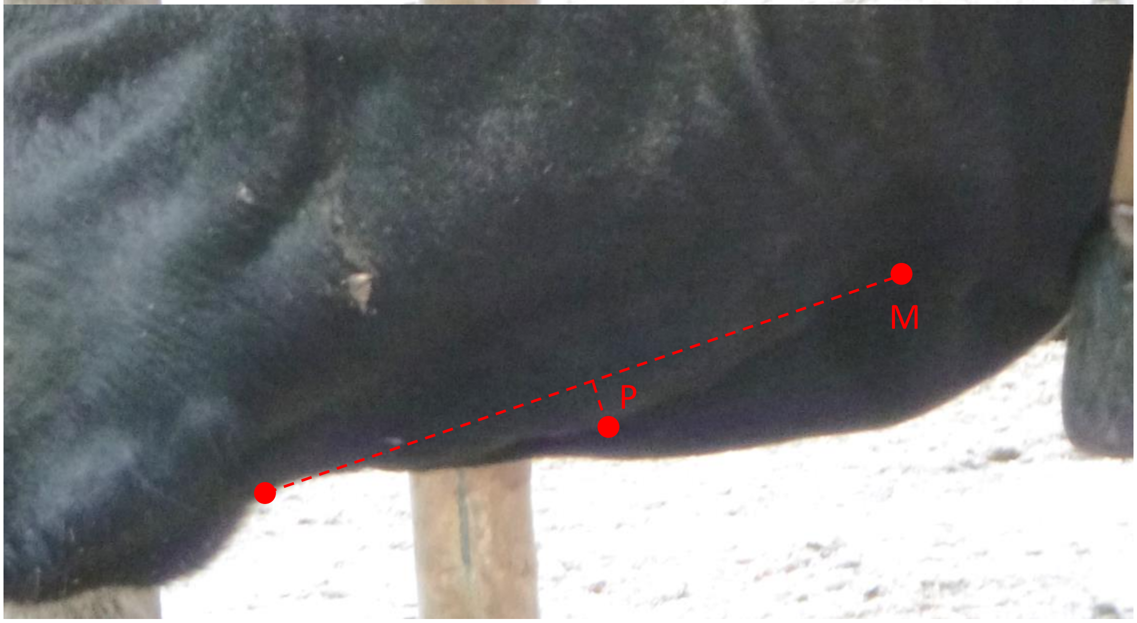

Muzzle Annotation

<u>Major Points of the Nares</u>

1) Tallest Point of the Nostril (I)
   Select the point where alar fold connects back to the meaty portion of the nares
2) Lowest Pint of the Nostril
   Select the point where the lowest point of the nostril connect to the philtrum (upper lip). This is typically identified as the point where the smooth and shiny skin of the inner nostril transitions back to the hair of the muzzle.
3) Caudal-Most Point of the Nostril
   Select the point of maximal perpendicular deviation in the direction of the skull from the vertical line of the nostril
4) Rostral-Most Point of the Nostril
   Select to point where the muscular inner flare of the inner nares connects with frontal fold of the nostril. For some cows, it will be possible to follow this line to where it terminates at the edge of the nostril. For other (typically darker) cows, this line can be visually projected from the inner nose outwards, and should be annotated where the outer nostril begins to curve back over the septum cartilage.

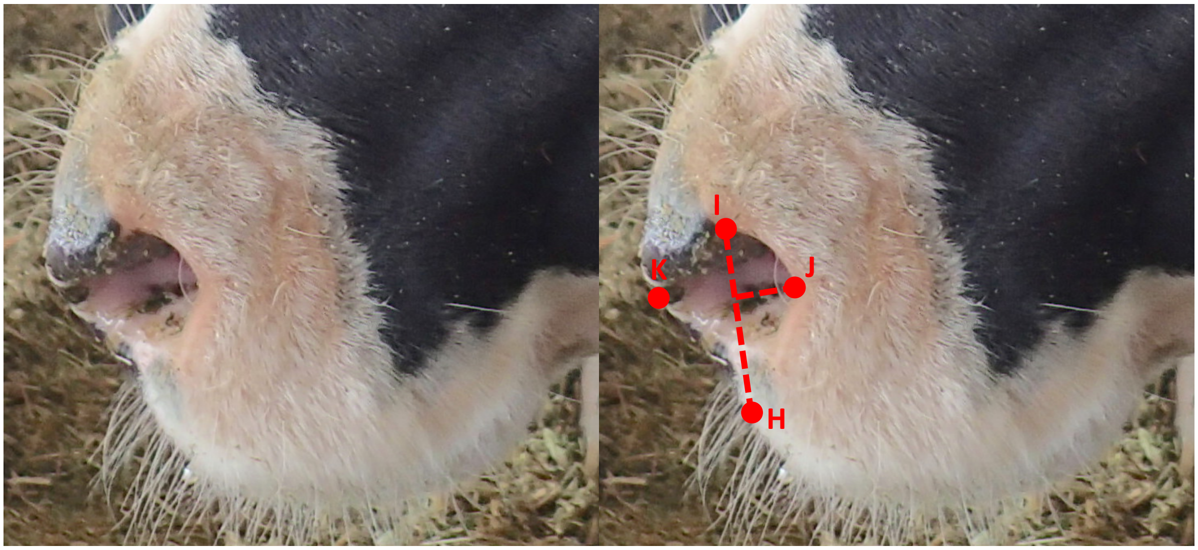

Inflection Points of the Nostril

5) Upper Front Inflection Point of Nostril (ki)
Select the caudal wing of the alar cartilage. This can be visualized by as the point where the upper nares (meaty inner flare) begins to curve back into the nose.
6) Upper Back Inflection Point of Nostril (ji)
Select the point along the line of the nostril demonstrating the greatest perpendicular deviation from the upper back line of the nostril.
7) Lower Back Inflection Point of Nostril (jh)
Select the point along the line of the nostril demonstrating the greatest perpendicular deviation from the upper back line of the nostril. This may occur on either side of the anatomical reference line. If there appears to be a tie – as much inward deviation towards the rostral side of the line as outward deviation towards the caudal side of the line – then select the point where the line of the nostril crosses the anatomical reference line.
8) Lower Front Inflection Point of Nostril (kh)
Select the point along the line of the nostril demonstrating the greatest perpendicular deviation from the lower front line of the nostril. If there is no deviation from this line, select a point on the reference line mid-way down its width.

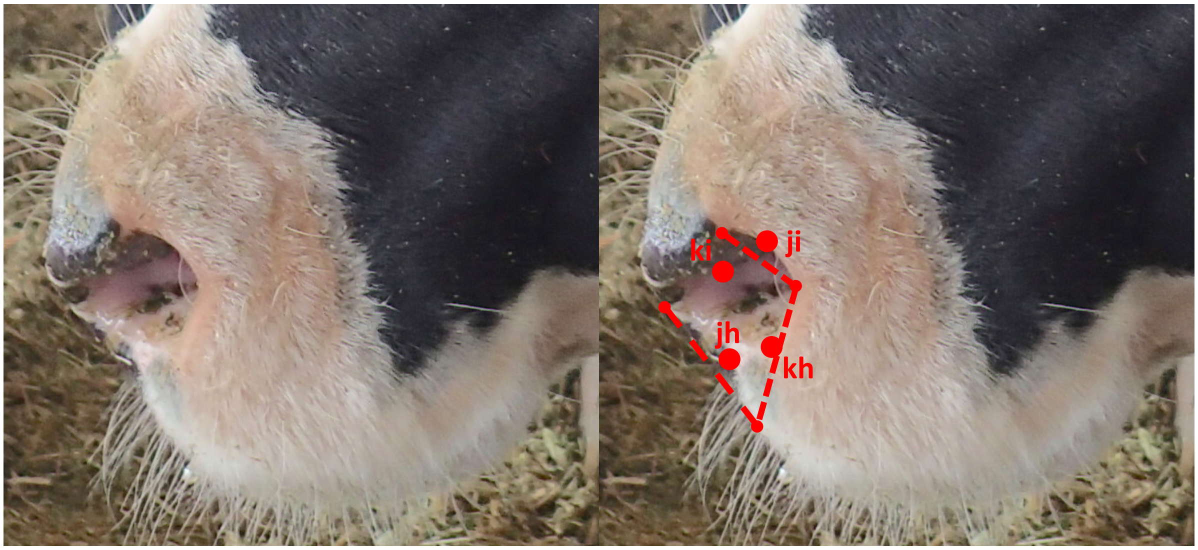

Annotation Points of the Muzzle

9) Lowest Point of the Philtrum (A)
Select the point where the reference leaves the upper jaw crossing the upper lip. If the point of the upper lip where it meets the lower lip can be clearly seen off the line, select by eye instead (may occur if the cow is slightly parrot jawed)

10) Point of Maximal Philtrum Deviance (B)
Select the point of maximal perpendicular deviation from the reference line of the upper lip. If the line of the philtrum does not deviate from the reference line, select a point on the reference line mid-way along its length.

11) Caudal-Most Point of Chin (L)
Select the point where the curvature of the back of the chin inverts to join the line of the jaw. If it joins the line of the jaw in a longer more gradual curve, select a point in the center of the crook formed

12) Deepest Point of Chin (Q)
Select the point of maximal perpendicular deviance along the profile of the chin.

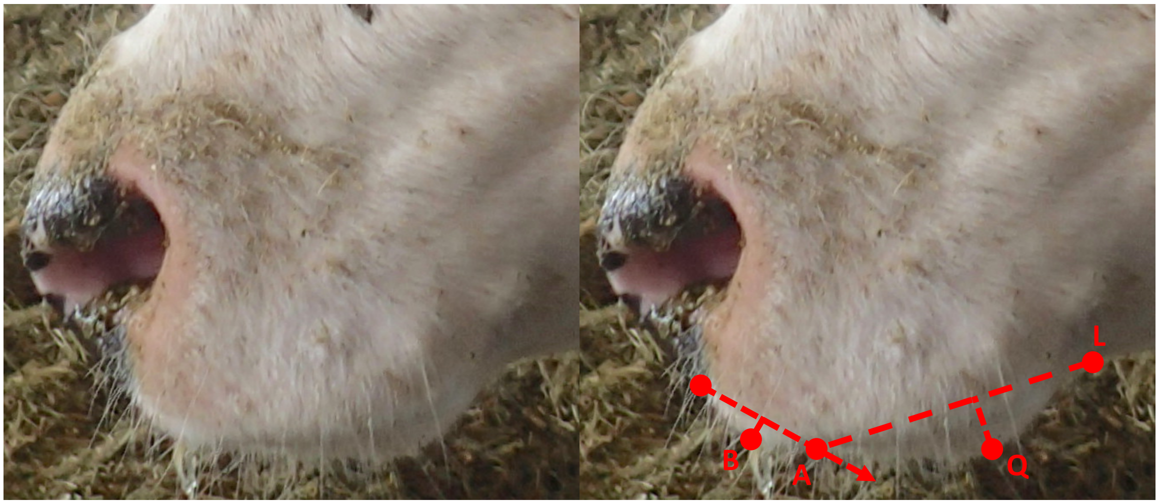

Topline Annotation

Muzzle Topline Annotation

1) Caudal-Most Point of Topline (E_upper)
   Select the point where the boney topline of the face attaches to the fleshy muzzle. In cows with heavier coats, the fur will often stick up at this point. Be sure to select the point adjacent to the bone. If there is some ambiguity where the boney anatomy ends and fleshy segment begins, select a point that splits the difference within the resulting inflection.
2) Rostral-Most Point of Muzzle (E_mid)
   Select the point where the nares creates an inflection point with the fleshy topline of the muzzle.
3) Midpoint of Muzzle (E_mid)
   Select the point along the topline of the muzzle that demonstrates the greatest perpendicular distance from the reference line of the muzzle

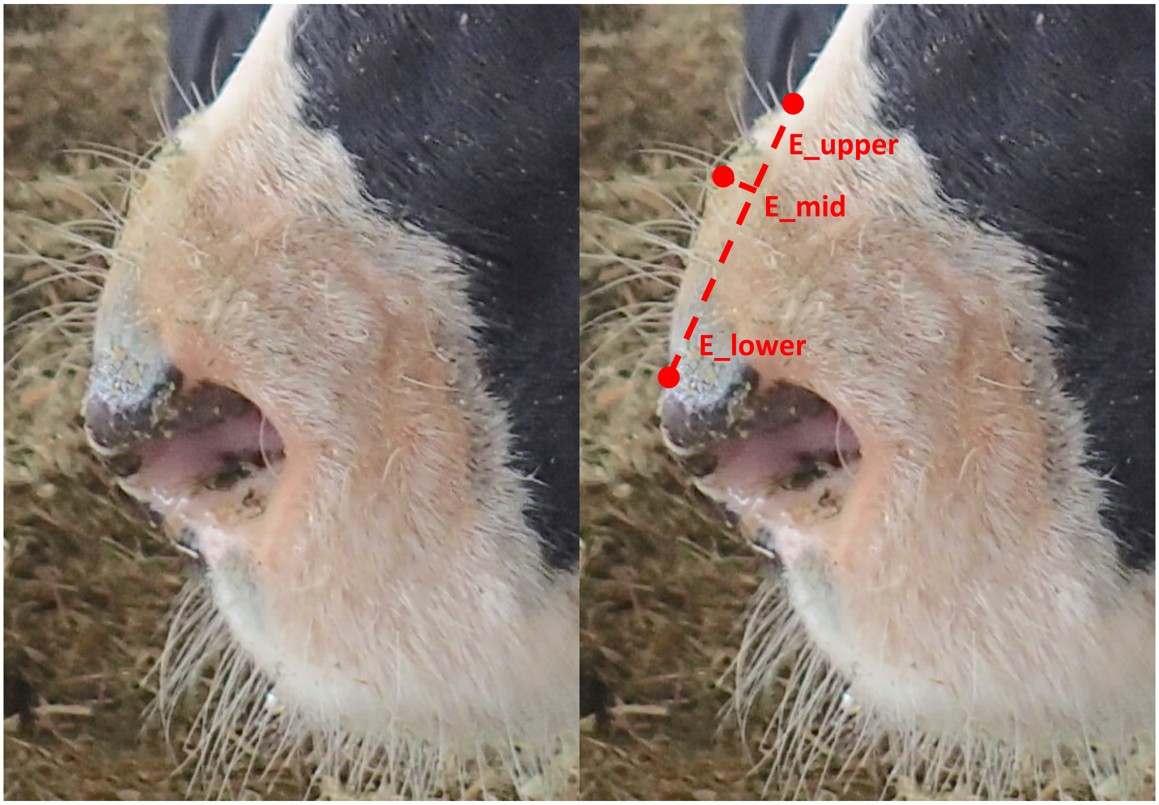

Main Topline Point Annotation

4) Caudal-Most Point of the Sinus (D)
   Select the point where the topline of the face meets the boney eye orbital
5) Rostral-Most Point of the Sinus (C)
   The section of the topline attributed to the conchofrontal sinus will present as a gradual downward curvature. At the rostral-most point of the sinus this curvature will either level off to a flat line or else serve as the point where there can be seen an inflection point to upward curvature. As the earlier effect can be subtle, a reference line has been provided to approximate the end of the sinus.
6) Caudal-Most Point of the Nose (F)
   Select the point roughly mid-way down the topline of the face where a point of downward curvature occurs roughly at the end of the nasal bone, often bounded by the upward inflections of the upper and lower topline. In some cows with particularly flat toplines this point may be difficult to distinguish, and so a rough reference line has been added to approximate the end of the nasal bone

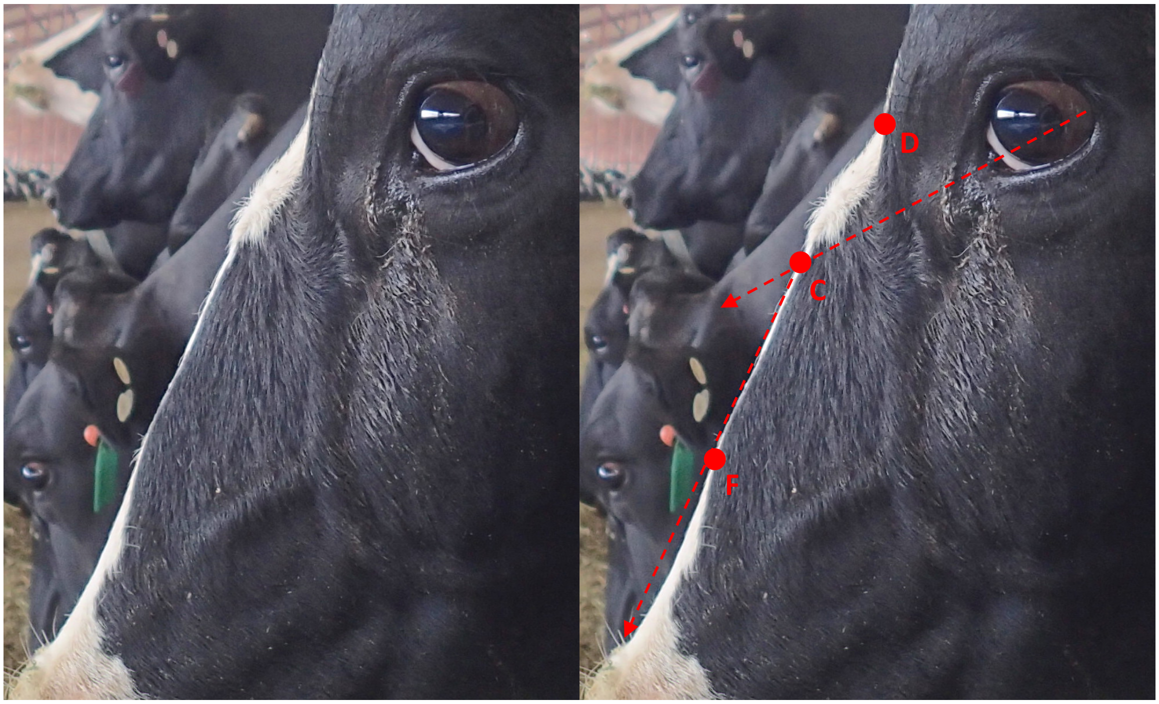

Topline Inflection Point Annotation

For GS, GU, and GL simply select the point of greatest perpendicular deviance from the provided reference line. If deviance occurs both above and below the reference line along its length, pick whichever deviance is greater. If deviance above and below the reference line appear roughly equivalent, select a point on the reference line mid-way along its length.
