# Supplementary material for: Improving the Reliability of Scale-Free Image Morphometrics in Applications with Minimally Restrained Livestock Using Projective Geometry and Unsupervised Machine Learning
Source: Sensors (Basel). 2022 Oct 31;22(21):8347. doi: 10.3390/s22218347 (PMC9653925; doi:10.3390/s22218347)
Supplement: Supplementary file 1 [file sensors-22-08347-s001.zip › SupplementalMaterials/Projective Biometric Derivations.pdf]

Eye Depth Proportion – Full Length

$$EDP = \frac{\|dy\|}{\|ab\|}$$

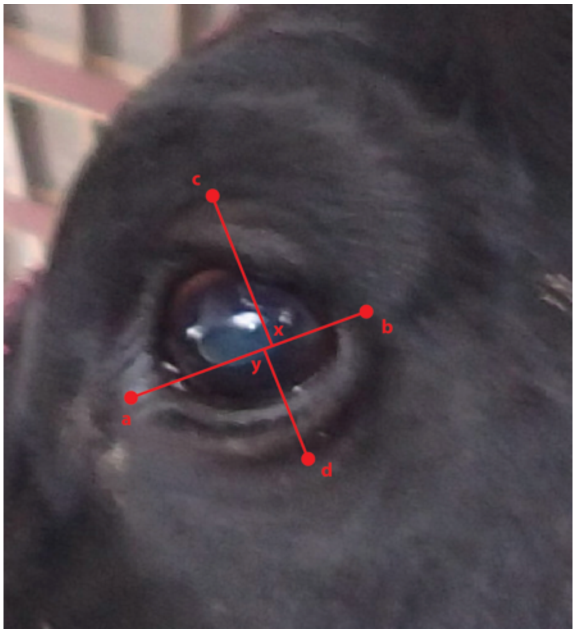

$V1 = \{aa, ba, ca, da, ea, fa, ga, ha\} \rightarrow \{a, b, c, d, e, f, g, h\}$
$V2 = \{ab, bb, cb, db, eb, fb, gb, hb\} \rightarrow \{a, b, c, d, e, f, g, h\}$
$V3 = \{ac, bc, cc, dc, ec, fc, gc, hc\} \rightarrow \{a, b, c, d, e, f, g, h\}$
$V4 = \{aa, ba, cb, db, eb, fb, gb, hb\} \rightarrow \{a, b, c, d, e, f, g, h\}$
$V5 = \{ab, bb, cc, dc, ec, fc, gc, hc\} \rightarrow \{a, b, c, d, e, f, g, h\}$

Eye Depth Point Proportion

$$EDPP = \frac{\|ay\|}{\|ab\|}$$

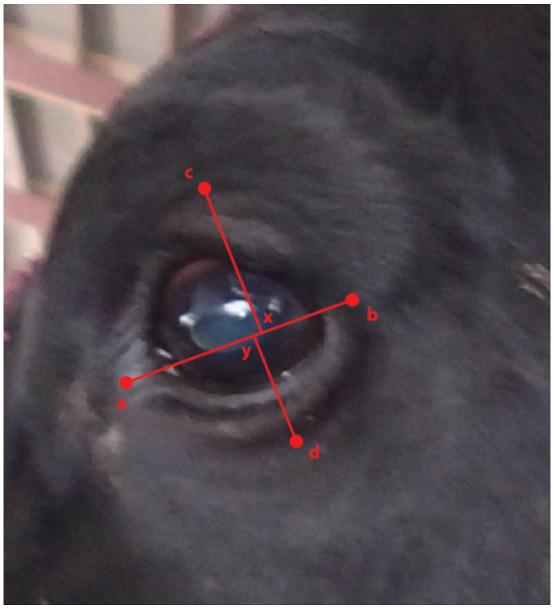

$V1 = \{aa, ba, ca, da, ea, fa, ga, ha\} \rightarrow \{a, b, c, d, e, f, g, h\}$
$V2 = \{ab, bb, cb, db, eb, fb, gb, hb\} \rightarrow \{a, b, c, d, e, f, g, h\}$
$V3 = \{ac, bc, cc, dc, ec, fc, gc, hc\} \rightarrow \{a, b, c, d, e, f, g, h\}$
$V4 = \{aa, ba, cb, db, eb, fb, gb, hb\} \rightarrow \{a, b, c, d, e, f, g, h\}$
$V5 = \{ab, bb, cc, dc, ec, fc, gc, hc\} \rightarrow \{a, b, c, d, e, f, g, h\}$

Eye Height Proportion – Full Length

$$EHP = \frac{\|cx\|}{\|ab\|}$$

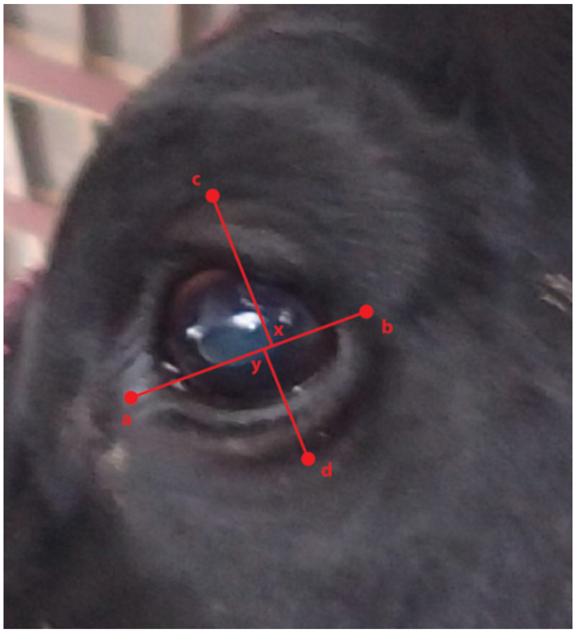

$V1 = \{aa, ba, ca, da, ea, fa, ga, ha\} \rightarrow \{a, b, c, d, e, f, g, h\}$
$V2 = \{ab, bb, cb, db, eb, fb, gb, hb\} \rightarrow \{a, b, c, d, e, f, g, h\}$
$V3 = \{ac, bc, cc, dc, ec, fc, gc, hc\} \rightarrow \{a, b, c, d, e, f, g, h\}$
$V4 = \{aa, ba, cb, db, eb, fb, gb, hb\} \rightarrow \{a, b, c, d, e, f, g, h\}$
$V5 = \{ab, bb, cc, dc, ec, fc, gc, hc\} \rightarrow \{a, b, c, d, e, f, g, h\}$

Eye Height Point Proportion

$$EHPP = \frac{\|ax\|}{\|ab\|}$$

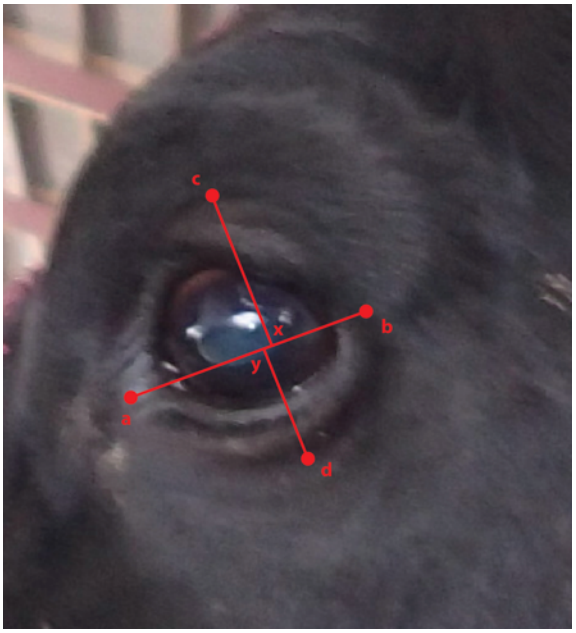

$V1 = \{aa, ba, ca, da, ea, fa, ga, ha\} \rightarrow \{a, b, c, d, e, f, g, h\}$
$V2 = \{ab, bb, cb, db, eb, fb, gb, hb\} \rightarrow \{a, b, c, d, e, f, g, h\}$
$V3 = \{ac, bc, cc, dc, ec, fc, gc, hc\} \rightarrow \{a, b, c, d, e, f, g, h\}$
$V4 = \{aa, ba, cb, db, eb, fb, gb, hb\} \rightarrow \{a, b, c, d, e, f, g, h\}$
$V5 = \{ab, bb, cc, dc, ec, fc, gc, hc\} \rightarrow \{a, b, c, d, e, f, g, h\}$

Eye Displacement Proportion

$$EDP = CF * \frac{\|xy\|}{\|ab\|}$$

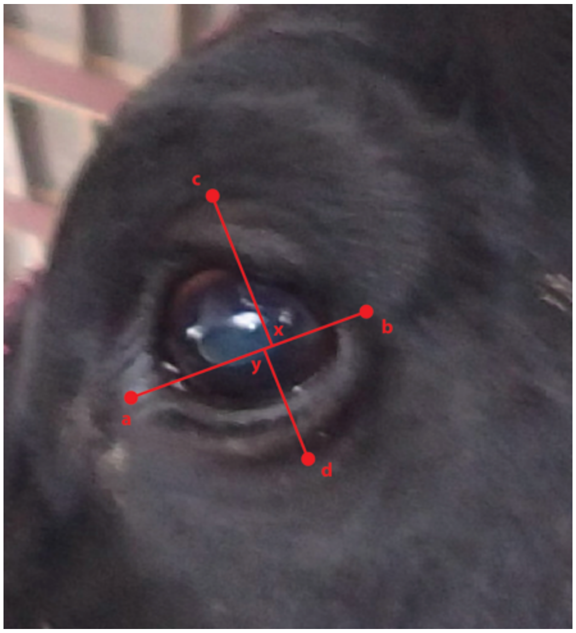

$$CF = \frac{\|ay\| - \|ax\|}{|\|ay\| - \|ax\||}$$

$V1 = \{aa, ba, ca, da, ea, fa, ga, ha\} \rightarrow \{a, b, c, d, e, f, g, h\}$
$V2 = \{ab, bb, cb, db, eb, fb, gb, hb\} \rightarrow \{a, b, c, d, e, f, g, h\}$
$V3 = \{ac, bc, cc, dc, ec, fc, gc, hc\} \rightarrow \{a, b, c, d, e, f, g, h\}$
$V4 = \{aa, ba, cb, db, eb, fb, gb, hb\} \rightarrow \{a, b, c, d, e, f, g, h\}$
$V5 = \{ab, bb, cc, dc, ec, fc, gc, hc\} \rightarrow \{a, b, c, d, e, f, g, h\}$

Eye Length Proportion - Length

$$ELP = \frac{\min\left(\|bx\| + \|by\|\right)}{\|ab\|}$$

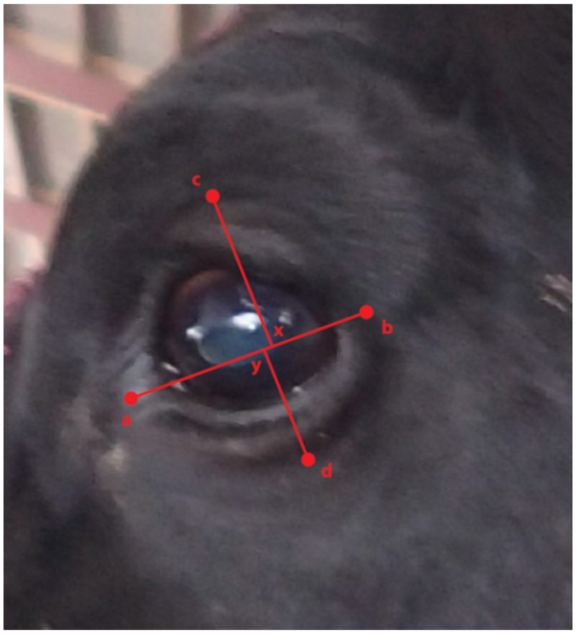

$V1 = \{aa, ba, ca, da, ea, fa, ga, ha\} \rightarrow \{a, b, c, d, e, f, g, h\}$
$V2 = \{ab, bb, cb, db, eb, fb, gb, hb\} \rightarrow \{a, b, c, d, e, f, g, h\}$
$V3 = \{ac, bc, cc, dc, ec, fc, gc, hc\} \rightarrow \{a, b, c, d, e, f, g, h\}$
$V4 = \{aa, ba, cb, db, eb, fb, gb, hb\} \rightarrow \{a, b, c, d, e, f, g, h\}$
$V5 = \{ab, bb, cc, dc, ec, fc, gc, hc\} \rightarrow \{a, b, c, d, e, f, g, h\}$

Eye Roundness Proportion – Upper Front

$$ERPUF = \frac{\|eq\|}{\|ac\|}$$

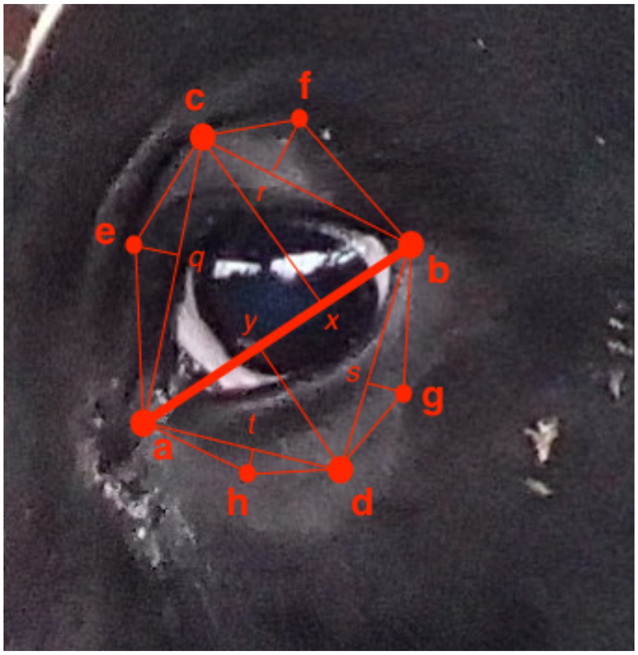

$V1 = \{aa, ba, ca, da, ea, fa, ga, ha\} \rightarrow \{a, b, c, d, e, f, g, h\}$
$V2 = \{ab, bb, cb, db, eb, fb, gb, hb\} \rightarrow \{a, b, c, d, e, f, g, h\}$
$V3 = \{ac, bc, cc, dc, ec, fc, gc, hc\} \rightarrow \{a, b, c, d, e, f, g, h\}$
$V4 = \{aa, ba, cb, db, eb, fb, gb, hb\} \rightarrow \{a, b, c, d, e, f, g, h\}$
$V5 = \{ab, bb, cc, dc, ec, fc, gc, hc\} \rightarrow \{a, b, c, d, e, f, g, h\}$

Eye Roundness Proportion – Upper Back

$$ERPUB = \frac{\|fr\|}{\|cb\|}$$

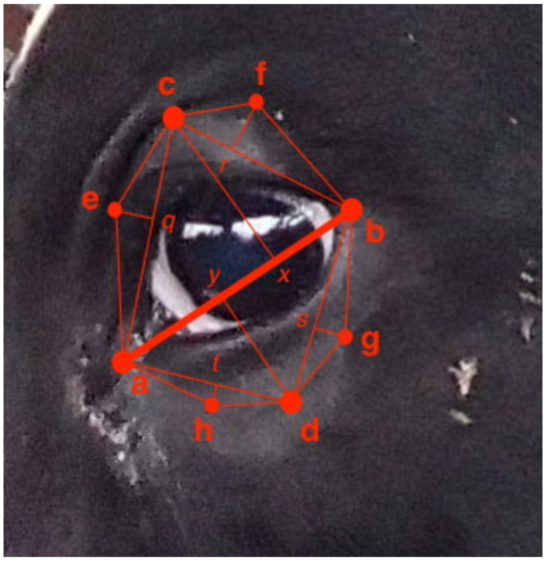

$V1 = \{aa, ba, ca, da, ea, fa, ga, ha\} \rightarrow \{a, b, c, d, e, f, g, h\}$
$V2 = \{ab, bb, cb, db, eb, fb, gb, hb\} \rightarrow \{a, b, c, d, e, f, g, h\}$
$V3 = \{ac, bc, cc, dc, ec, fc, gc, hc\} \rightarrow \{a, b, c, d, e, f, g, h\}$
$V4 = \{aa, ba, cb, db, eb, fb, gb, hb\} \rightarrow \{a, b, c, d, e, f, g, h\}$
$V5 = \{ab, bb, cc, dc, ec, fc, gc, hc\} \rightarrow \{a, b, c, d, e, f, g, h\}$

Eye Roundness Proportion – Lower Back

$$ERPLB = \frac{\|gs\|}{\|bd\|}$$

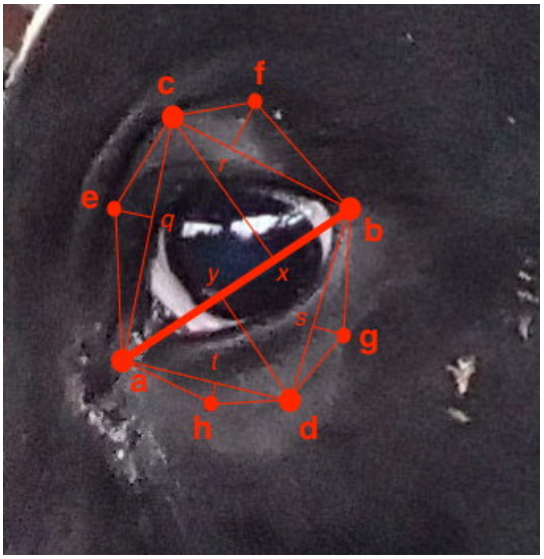

$V1 = \{aa, ba, ca, da, ea, fa, ga, ha\} \rightarrow \{a, b, c, d, e, f, g, h\}$
$V2 = \{ab, bb, cb, db, eb, fb, gb, hb\} \rightarrow \{a, b, c, d, e, f, g, h\}$
$V3 = \{ac, bc, cc, dc, ec, fc, gc, hc\} \rightarrow \{a, b, c, d, e, f, g, h\}$
$V4 = \{aa, ba, cb, db, eb, fb, gb, hb\} \rightarrow \{a, b, c, d, e, f, g, h\}$
$V5 = \{ab, bb, cc, dc, ec, fc, gc, hc\} \rightarrow \{a, b, c, d, e, f, g, h\}$

Eye Roundness Proportion – Lower Front

$$ERPUB = \frac{\|ht\|}{\|ad\|}$$

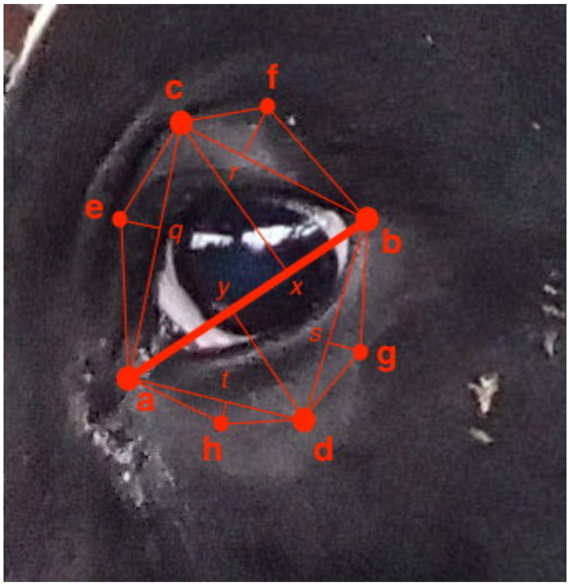

$V1 = \{aa, ba, ca, da, ea, fa, ga, ha\} \rightarrow \{a, b, c, d, e, f, g, h\}$
$V2 = \{ab, bb, cb, db, eb, fb, gb, hb\} \rightarrow \{a, b, c, d, e, f, g, h\}$
$V3 = \{ac, bc, cc, dc, ec, fc, gc, hc\} \rightarrow \{a, b, c, d, e, f, g, h\}$
$V4 = \{aa, ba, cb, db, eb, fb, gb, hb\} \rightarrow \{a, b, c, d, e, f, g, h\}$
$V5 = \{ab, bb, cc, dc, ec, fc, gc, hc\} \rightarrow \{a, b, c, d, e, f, g, h\}$

Canthus Depth Proportion - Depth

$$CDPD = \frac{\|CAN_{int}, CAN\|}{\|Z_{int}, Z\|}$$

$$V1 = \{aa, ba, da\} \rightarrow \{W, X, Z\}$$
$$V2 = \{aa, ba, db\} \rightarrow \{W, X, Z\}$$
$$V3 = \{aa, ba, dc\} \rightarrow \{W, X, Z\}$$
$$V4 = \{ab, bb, da\} \rightarrow \{W, X, Z\}$$
$$V5 = \{ab, bb, db\} \rightarrow \{W, X, Z\}$$
$$V6 = \{ab, bb, dc\} \rightarrow \{W, X, Z\}$$

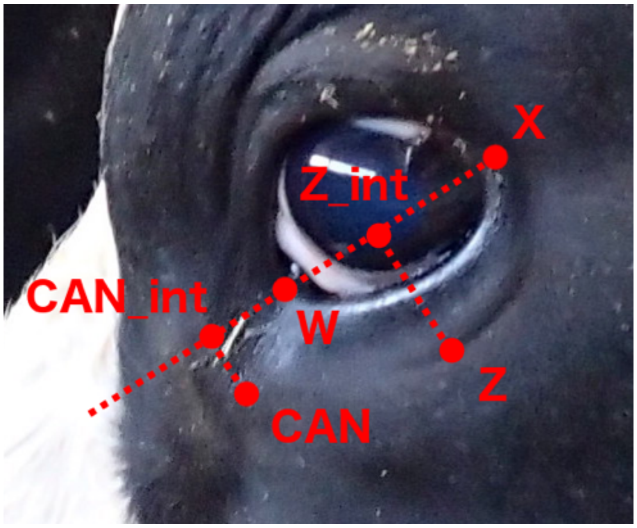

Canthus Depth Proportion - Length

$$CDPL = \frac{\|CAN_{int}, CAN\|}{\|W, X\|}$$

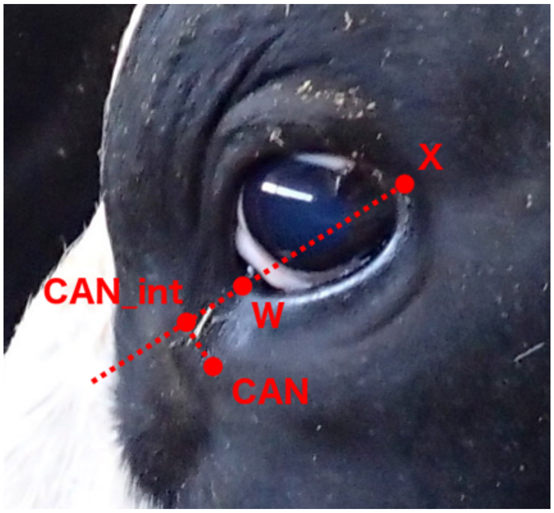

$V1 = \{aa, ba\} \rightarrow \{W, X\}$
$V2 = \{ab, bb\} \rightarrow \{W, X\}$
$V3 = \{ac, bc\} \rightarrow \{W, X\}$
$V4 = \{aa, bb\} \rightarrow \{W, X\}$
$V5 = \{ab, ba\} \rightarrow \{W, X\}$

Canthus Length Proportion

$$CLP = \frac{\|CAN_{int}, W\|}{\|W, X\|}$$

$V1 = \{aa, ba\} \rightarrow \{W, X\}$
$V2 = \{ab, bb\} \rightarrow \{W, X\}$
$V3 = \{ac, bc\} \rightarrow \{W, X\}$
$V4 = \{aa, bb\} \rightarrow \{W, X\}$
$V5 = \{ab, ba\} \rightarrow \{W, X\}$

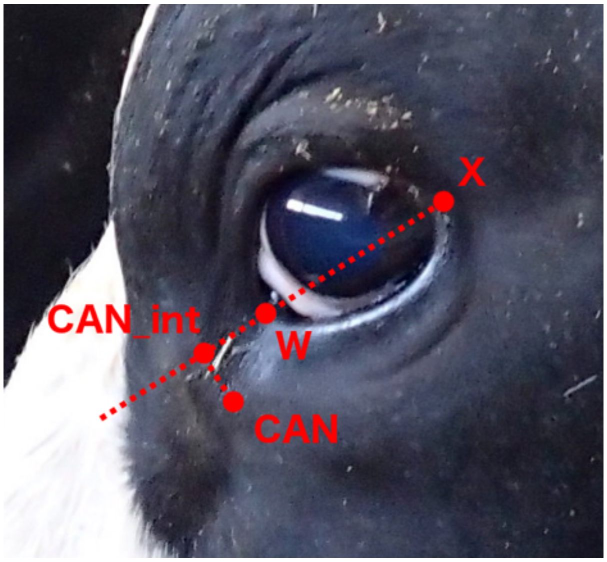

Canthus Width-to-Height Ratio

$$CWTHR = \frac{\|CAN, CAN_{int}\|}{\|CAN_{int}, W\|}$$

$V1 = \{aa, ba\} \rightarrow \{W, X\}$
$V2 = \{ab, bb\} \rightarrow \{W, X\}$
$V3 = \{ac, bc\} \rightarrow \{W, X\}$
$V4 = \{aa, bb\} \rightarrow \{W, X\}$
$V5 = \{ab, ba\} \rightarrow \{W, X\}$

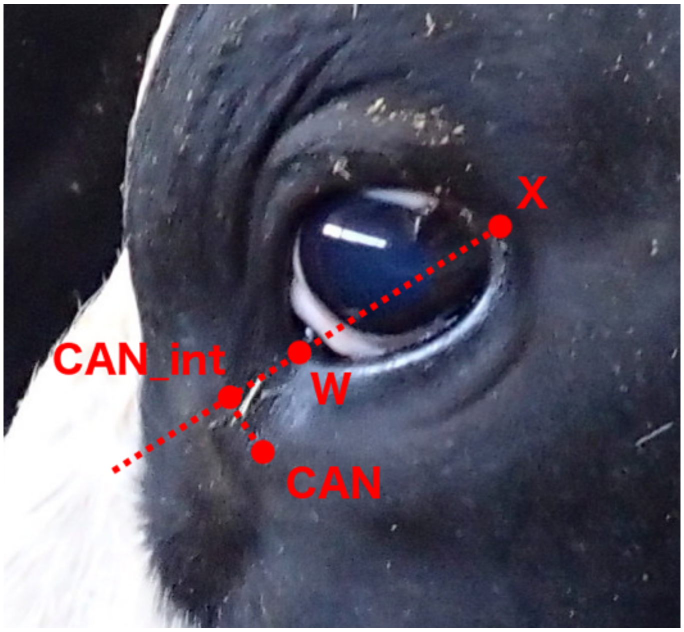

Chin Length Proportion

$$CLP = \frac{\|L, A_{int}\|}{\|L, U_{int}\|}$$

$$V1 = \{A_{extrap}, L\} \rightarrow \{A, L\}$$
$$V2 = \{A_{eye}, L\} \rightarrow \{A, L\}$$
$$V3 = \{B, L\} \rightarrow \{A, L\}$$

$$V4 = \{A_{extrap}, L_{full}\} \rightarrow \{A, L\}$$
$$V5 = \{A_{eye}, L_{full}\} \rightarrow \{A, L\}$$
$$V6 = \{B, L_{full}\} \rightarrow \{A, L\}$$

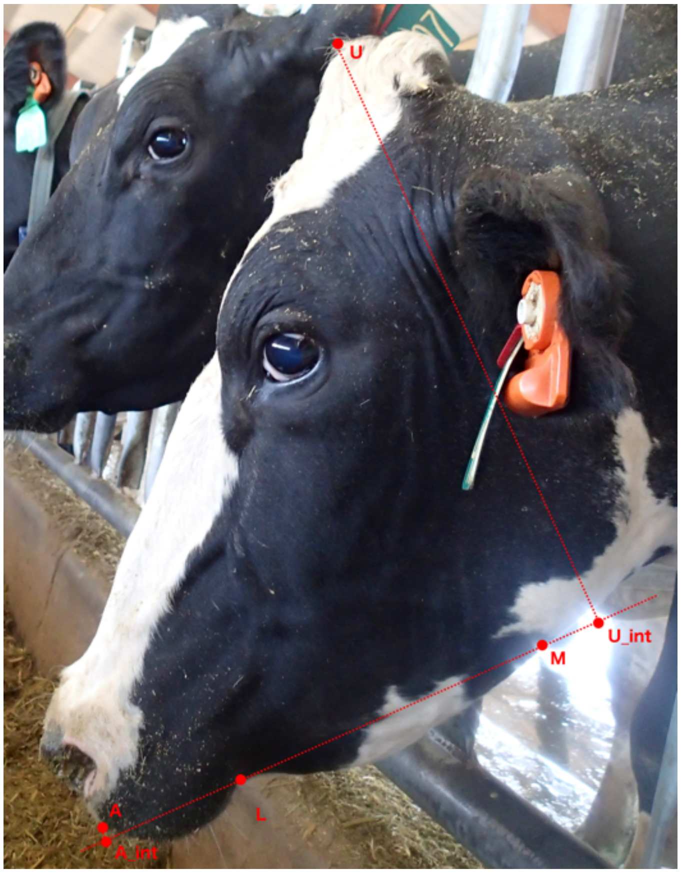

Cranio-Topline Length Ratio

$$CTLR = \frac{\|S, T_{int}\|}{\|W, H\|}$$

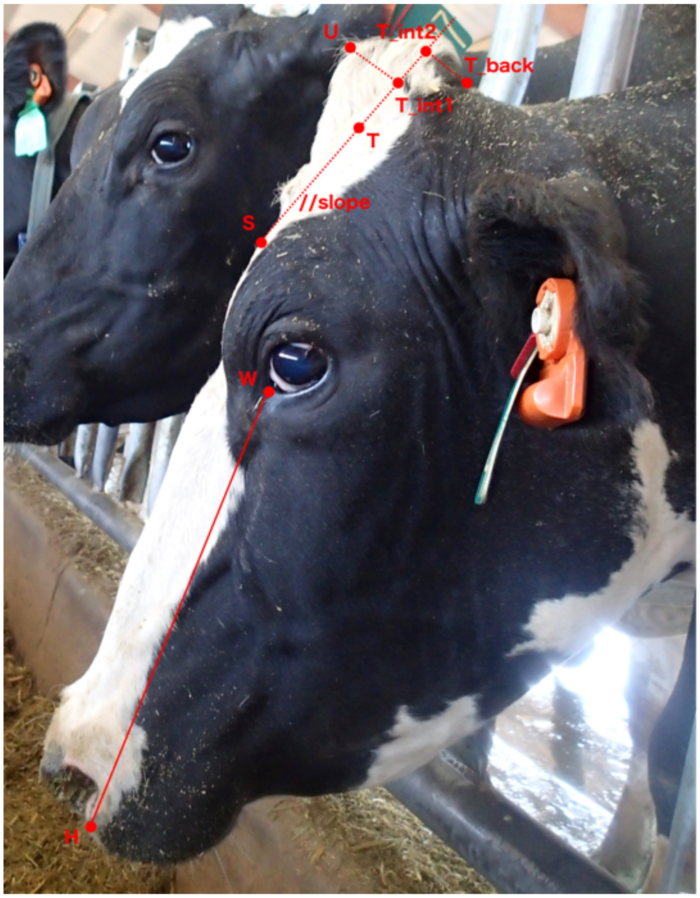

$V1 = \{S_{extrap}, T_{int1}, T_{slope}\} \rightarrow \{S, T_{int}, T\}$
$V2 = \{S_{extrap}, T_{int1}, T_{top}\} \rightarrow \{S, T_{int}, T\}$
$V3 = \{S_{extrap}, T_{int1}, T_{poll}\} \rightarrow \{S, T_{int}, T\}$

$V4 = \{S_{extrap}, T_{int2}, T_{slope}\} \rightarrow \{S, T_{int}, T\}$
$V5 = \{S_{extrap}, T_{int2}, T_{top}\} \rightarrow \{S, T_{int}, T\}$
$V6 = \{S_{extrap}, T_{int2}, T_{poll}\} \rightarrow \{S, T_{int}, T\}$

$V7 = \{S_{eye}, T_{int1}, T_{slope}\} \rightarrow \{S, T_{int}, T\}$
$V8 = \{S_{eye}, T_{int1}, T_{top}\} \rightarrow \{S, T_{int}, T\}$
$V9 = \{S_{eye}, T_{int1}, T_{poll}\} \rightarrow \{S, T_{int}, T\}$

$V10 = \{S_{eye}, T_{int2}, T_{slope}\} \rightarrow \{S, T_{int}, T\}$
$V11 = \{S_{eye}, T_{int2}, T_{top}\} \rightarrow \{S, T_{int}, T\}$
$V12 = \{S_{eye}, T_{int2}, T_{poll}\} \rightarrow \{S, T_{int}, T\}$

Eye Forehead Size Ratio_Linear

$$EFSR = \frac{\|W, X\|}{\|W, WX_{int}\|}$$

$$V1 = \{aa, ba\} \rightarrow \{W, X\}$$
$$V2 = \{ab, bb\} \rightarrow \{W, X\}$$
$$V3 = \{ac, bc\} \rightarrow \{W, X\}$$
$$V4 = \{aa, bb\} \rightarrow \{W, X\}$$
$$V5 = \{ab, ba\} \rightarrow \{W, X\}$$

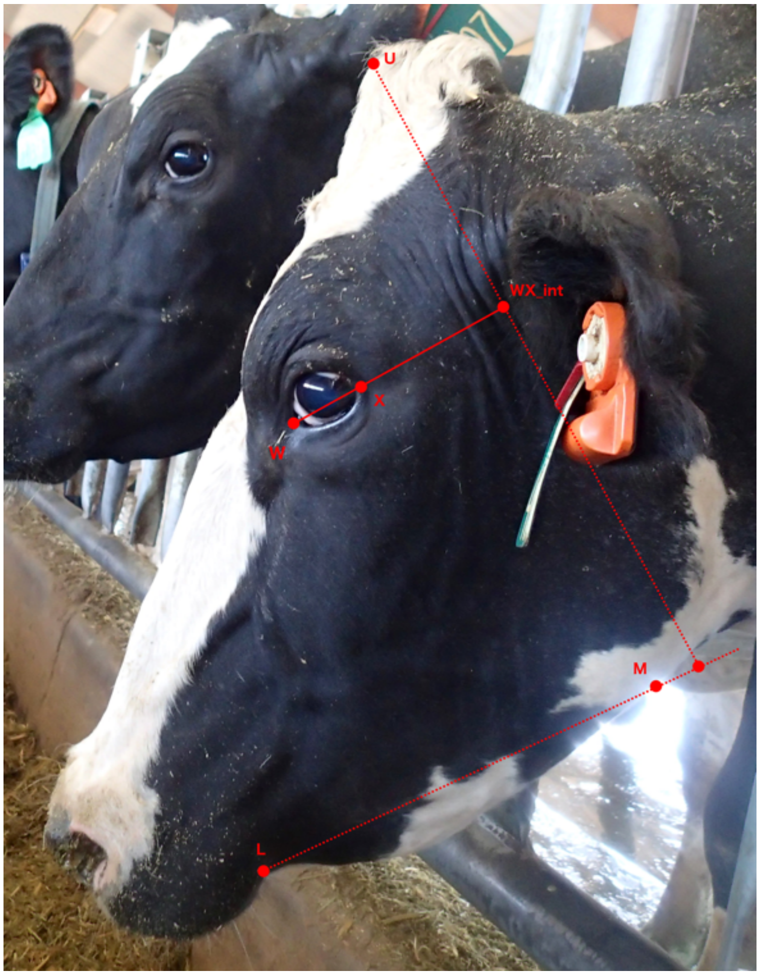

Eye Orbital Height-to-Length Ratio

$$EOHLR = \frac{\|S, S_{int}\|}{\|D, S_{int}\|}$$

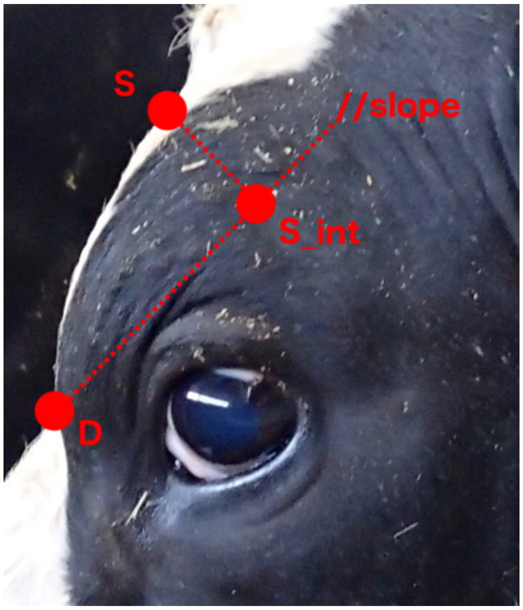

$V1 = \{S_{extrap}, \overline{HW}\} \rightarrow \{S, //slope\}$

$V2 = \{S_{extrap}, \overline{S_{eye}T_{slope}}\} \rightarrow \{S, //slope\}$
$V3 = \{S_{extrap}, \overline{S_{eye}T_{poll}}\} \rightarrow \{S, //slope\}$
$V4 = \{S_{extrap}, \overline{S_{eye}T_{top}}\} \rightarrow \{S, //slope\}$

$V5 = \{S_{extrap}, \overline{S_{extrap}T_{slope}}\} \rightarrow \{S, //slope\}$
$V6 = \{S_{extrap}, \overline{S_{extrap}T_{poll}}\} \rightarrow \{S, //slope\}$
$V7 = \{S_{extrap}, \overline{S_{extrap}T_{top}}\} \rightarrow \{S, //slope\}$

$V8 = \{S_{eye}, \overline{HW}\} \rightarrow \{S, //slope\}$

$V9 = \{S_{eye}, \overline{S_{eye}T_{slope}}\} \rightarrow \{S, //slope\}$
$V10 = \{S_{eye}, \overline{S_{eye}T_{poll}}\} \rightarrow \{S, //slope\}$
$V11 = \{S_{eye}, \overline{S_{eye}T_{top}}\} \rightarrow \{S, //slope\}$

$V12 = \{S_{eye}, \overline{S_{extrap}T_{slope}}\} \rightarrow \{S, //slope\}$
$V13 = \{S_{eye}, \overline{S_{extrap}T_{poll}}\} \rightarrow \{S, //slope\}$
$V14 = \{S_{eye}, \overline{S_{extrap}T_{top}}\} \rightarrow \{S, //slope\}$

Eye Orbital Projection Proportion

$$EOPP = \frac{\left\|Y, S_{extrap}\right\|}{\|Y, Z\|}$$

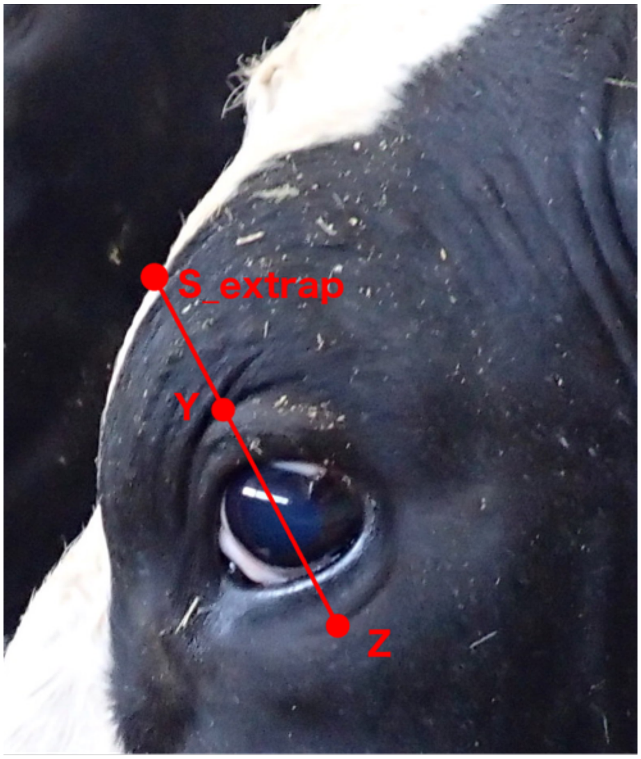

$V1 = \{ca, da\} \rightarrow \{Y, Z\}$
$V2 = \{ca, db\} \rightarrow \{Y, Z\}$
$V3 = \{ca, dc\} \rightarrow \{Y, Z\}$

$V4 = \{cb, da\} \rightarrow \{Y, Z\}$
$V5 = \{cb, db\} \rightarrow \{Y, Z\}$
$V6 = \{cb, dc\} \rightarrow \{Y, Z\}$

$V7 = \{cc, da\} \rightarrow \{Y, Z\}$
$V8 = \{cc, db\} \rightarrow \{Y, Z\}$
$V9 = \{cc, dc\} \rightarrow \{Y, Z\}$

Eye Orbital Roundness Point Proportion

$$EORPP = \frac{\|D, R_{int}\|}{\|D, S\|}$$

$$V1 = \{S_{extrap}\} \rightarrow \{S\}$$
$$V2 = \{S_{eye}\} \rightarrow \{S\}$$

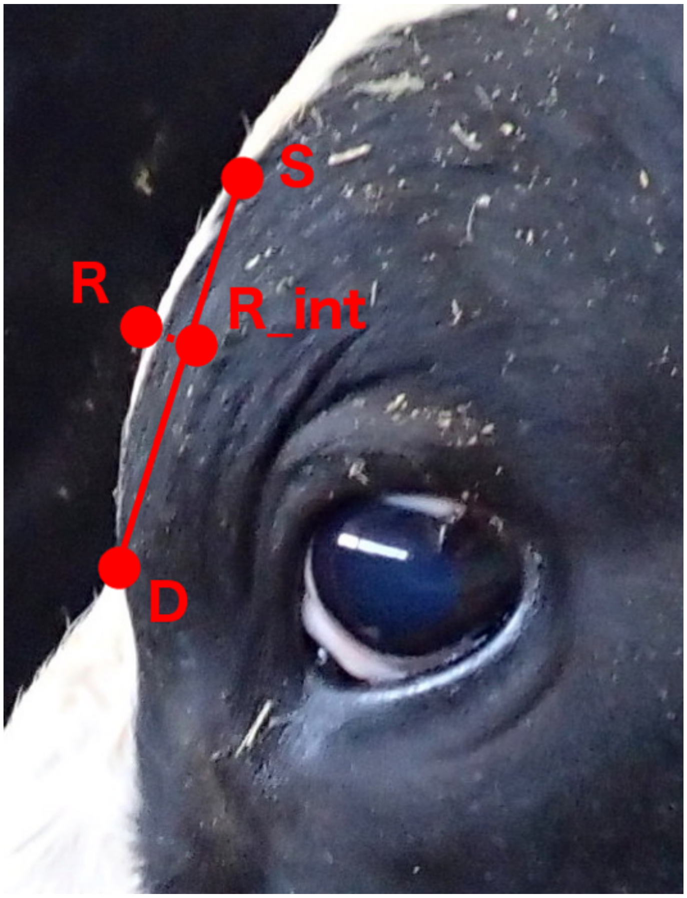

Eye Orbital Roundness Proportion

$$EORP = \frac{\|R, R_{int}\|}{\|G, S\|}$$

$V1 = \{S_{extrap}\} \rightarrow \{S\}$
$V2 = \{S_{eye}\} \rightarrow \{S\}$

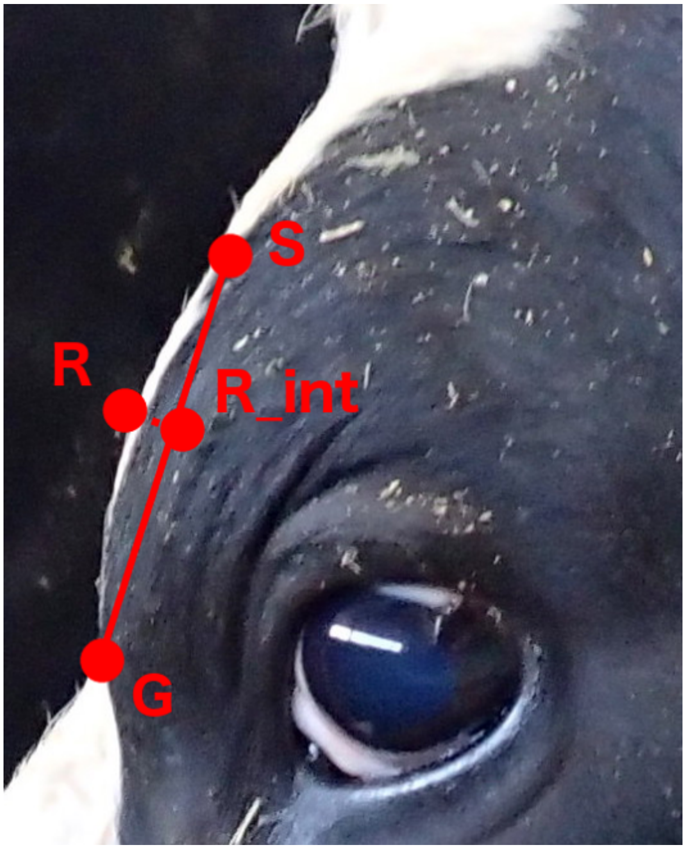

Eye Orbital-Eye Height Ratio

$$EOEHR = \frac{\|S, S_{int}\|}{\|Z, Z_{int}\|}$$

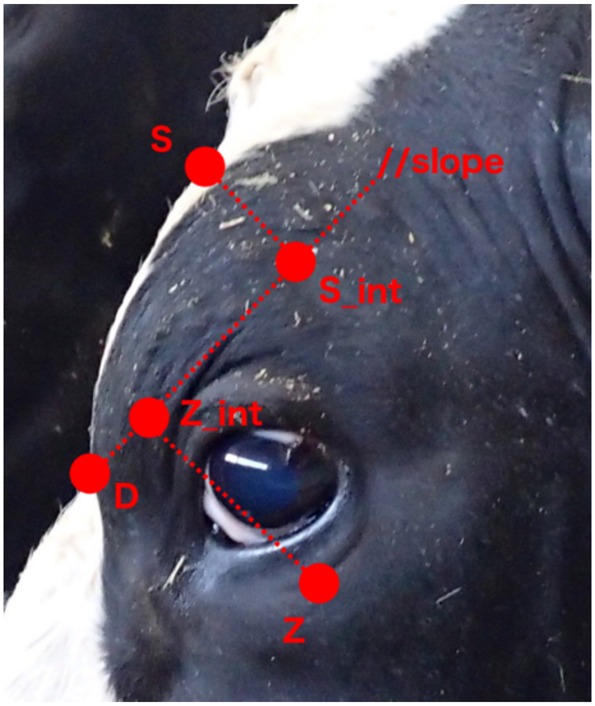

$V1 = \{da, S_{eye}, \overline{HW}\} \rightarrow \{Z, S, //slope\}$
$V2 = \{da, S_{eye}, \overline{S_{eye}T_{slope}}\} \rightarrow \{Z, S, //slope\}$
$V3 = \{da, S_{eye}, \overline{S_{eye}T_{poll}}\} \rightarrow \{Z, S, //slope\}$
$V4 = \{da, S_{eye}, \overline{S_{eye}T_{top}}\} \rightarrow \{Z, S, //slope\}$
$V5 = \{da, S_{eye}, \overline{S_{extrap}T_{slope}}\} \rightarrow \{Z, S, //slope\}$
$V6 = \{da, S_{eye}, \overline{S_{extrap}T_{poll}}\} \rightarrow \{Z, S, //slope\}$
$V7 = \{da, S_{eye}, \overline{S_{extrap}T_{top}}\} \rightarrow \{Z, S, //slope\}$

$V8 = \{da, S_{extrap}, \overline{HW}\} \rightarrow \{Z, S, //slope\}$
$V9 = \{da, S_{extrap}, \overline{S_{eye}T_{slope}}\} \rightarrow \{Z, S, //slope\}$
$V10 = \{da, S_{extrap}, \overline{S_{eye}T_{poll}}\} \rightarrow \{Z, S, //slope\}$
$V11 = \{da, S_{extrap}, \overline{S_{eye}T_{top}}\} \rightarrow \{Z, S, //slope\}$
$V12 = \{da, S_{extrap}, \overline{S_{extrap}T_{slope}}\} \rightarrow \{Z, S, //slope\}$
$V13 = \{da, S_{extrap}, \overline{S_{extrap}T_{poll}}\} \rightarrow \{Z, S, //slope\}$
$V14 = \{da, S_{extrap}, \overline{S_{extrap}T_{top}}\} \rightarrow \{Z, S, //slope\}$

$V15 = \{db, S_{eye}, \overline{HW}\} \rightarrow \{Z, S, //slope\}$
$V16 = \{db, S_{eye}, \overline{S_{eye}T_{slope}}\} \rightarrow \{Z, S, //slope\}$
$V17 = \{db, S_{eye}, \overline{S_{eye}T_{poll}}\} \rightarrow \{Z, S, //slope\}$
$V18 = \{db, S_{eye}, \overline{S_{eye}T_{top}}\} \rightarrow \{Z, S, //slope\}$
$V19 = \{db, S_{eye}, \overline{S_{extrap}T_{slope}}\} \rightarrow \{Z, S, //slope\}$
$V20 = \{db, S_{eye}, \overline{S_{extrap}T_{poll}}\} \rightarrow \{Z, S, //slope\}$
$V21 = \{db, S_{eye}, \overline{S_{extrap}T_{top}}\} \rightarrow \{Z, S, //slope\}$

$V22 = \{db, S_{extrap}, \overline{HW}\} \rightarrow \{Z, S, //slope\}$
$V23 = \{db, S_{extrap}, \overline{S_{eye}T_{slope}}\} \rightarrow \{Z, S, //slope\}$
$V24 = \{db, S_{extrap}, \overline{S_{eye}T_{poll}}\} \rightarrow \{Z, S, //slope\}$
$V25 = \{db, S_{extrap}, \overline{S_{eye}T_{top}}\} \rightarrow \{Z, S, //slope\}$
$V26 = \{db, S_{extrap}, \overline{S_{extrap}T_{slope}}\} \rightarrow \{Z, S, //slope\}$
$V27 = \{db, S_{extrap}, \overline{S_{extrap}T_{poll}}\} \rightarrow \{Z, S, //slope\}$
$V28 = \{db, S_{extrap}, \overline{S_{extrap}T_{top}}\} \rightarrow \{Z, S, //slope\}$

$V29 = \{dc, S_{eye}, \overline{HW}\} \rightarrow \{Z, S, //slope\}$
$V30 = \{dc, S_{eye}, \overline{S_{eye}T_{slope}}\} \rightarrow \{Z, S, //slope\}$
$V31 = \{dc, S_{eye}, \overline{S_{eye}T_{poll}}\} \rightarrow \{Z, S, //slope\}$
$V32 = \{dc, S_{eye}, \overline{S_{eye}T_{top}}\} \rightarrow \{Z, S, //slope\}$
$V33 = \{dc, S_{eye}, \overline{S_{extrap}T_{slope}}\} \rightarrow \{Z, S, //slope\}$
$V34 = \{dc, S_{eye}, \overline{S_{extrap}T_{poll}}\} \rightarrow \{Z, S, //slope\}$
$V35 = \{dc, S_{eye}, \overline{S_{extrap}T_{top}}\} \rightarrow \{Z, S, //slope\}$

$V36 = \{dc, S_{extrap}, \overline{HW}\} \rightarrow \{Z, S, //slope\}$
$V37 = \{dc, S_{extrap}, \overline{S_{eye}T_{slope}}\} \rightarrow \{Z, S, //slope\}$
$V38 = \{dc, S_{extrap}, \overline{S_{eye}T_{poll}}\} \rightarrow \{Z, S, //slope\}$
$V39 = \{dc, S_{extrap}, \overline{S_{eye}T_{top}}\} \rightarrow \{Z, S, //slope\}$
$V40 = \{dc, S_{extrap}, \overline{S_{extrap}T_{slope}}\} \rightarrow \{Z, S, //slope\}$
$V41 = \{dc, S_{extrap}, \overline{S_{extrap}T_{poll}}\} \rightarrow \{Z, S, //slope\}$
$V42 = \{dc, S_{extrap}, \overline{S_{extrap}T_{top}}\} \rightarrow \{Z, S, //slope\}$

Eye Topline Size Ratio - Linear

$$ETSRL = \frac{\|W,X\|}{\|W,H\|}$$

$$V1 = \{aa, ba\} \rightarrow \{W, X\}$$
$$V2 = \{ab, bb\} \rightarrow \{W, X\}$$
$$V3 = \{ac, bc\} \rightarrow \{W, X\}$$
$$V4 = \{aa, bb\} \rightarrow \{W, X\}$$
$$V5 = \{ab, ba\} \rightarrow \{W, X\}$$

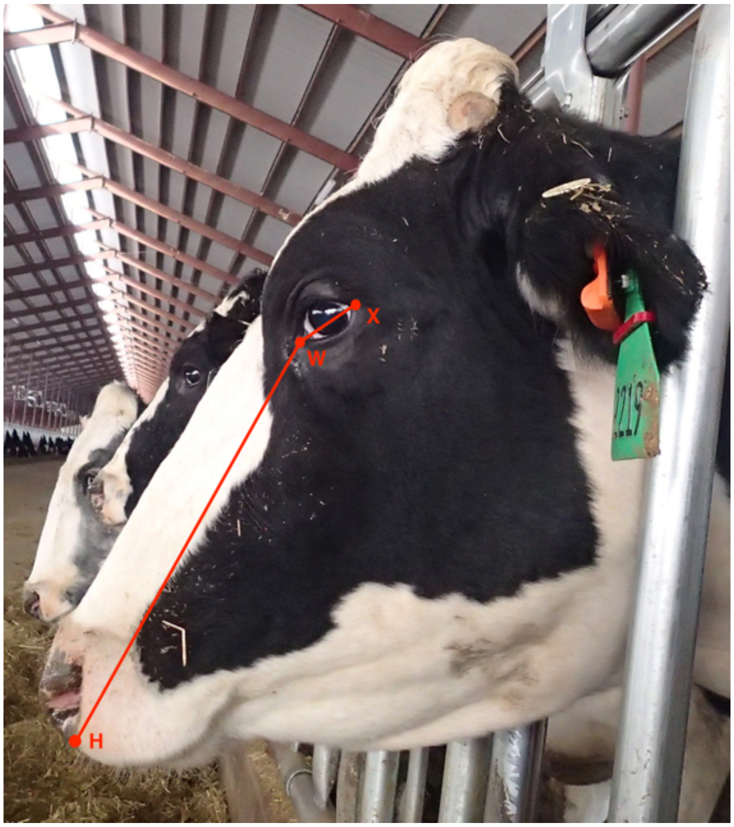

Eye-Sinus Size Ratio_Linear

$$ESSRL = \frac{\|W, X\|}{\|C_{int}, X\|}$$

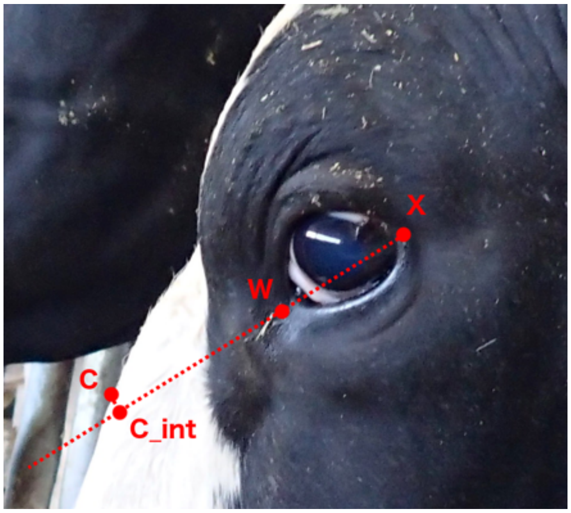

$$V1 = \{aa, ba, C_{extrap}\} \rightarrow \{W, X, C\}$$
$$V2 = \{ab, bb, C_{extrap}\} \rightarrow \{W, X, C\}$$
$$V3 = \{ac, bc, C_{extrap}\} \rightarrow \{W, X, C\}$$
$$V4 = \{aa, bb, C_{extrap}\} \rightarrow \{W, X, C\}$$
$$V5 = \{ab, ba, C_{extrap}\} \rightarrow \{W, X, C\}$$

$$V6 = \{aa, ba, C_{eye}\} \rightarrow \{W, X, C\}$$
$$V7 = \{ab, bb, C_{eye}\} \rightarrow \{W, X, C\}$$
$$V8 = \{ac, bc, C_{eye}\} \rightarrow \{W, X, C\}$$
$$V9 = \{aa, bb, C_{eye}\} \rightarrow \{W, X, C\}$$
$$V10 = \{ab, ba, C_{eye}\} \rightarrow \{W, X, C\}$$

Forehead-Eye Angle_Slope

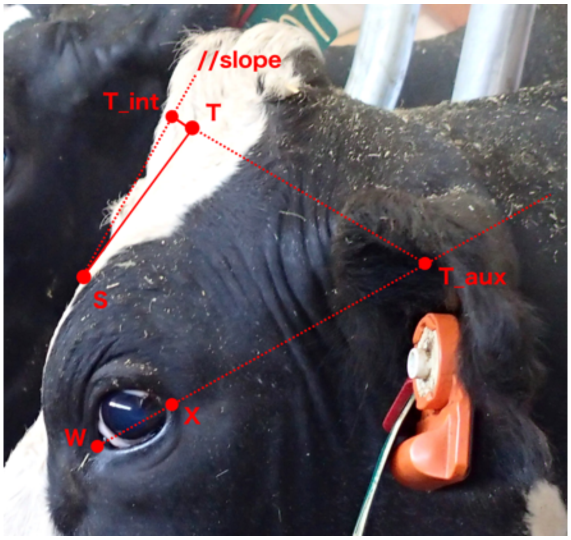

$$FEAS = CF * \frac{\|T, T_{int}\|}{\|S, T\|}$$

$$CF = \frac{\|T_{int}, T_{aux}\| - \|T, T_{aux}\|}{|\|T_{int}, T_{aux}\| - \|T, T_{aux}\||}$$

$V1 = \{\overline{aaba}, S_{extrap}, T_{slope}\} \to \{//slope, S, T\}$
$V2 = \{\overline{aaba}, S_{extrap}, T_{top}\} \to \{//slope, S, T\}$
$V3 = \{\overline{aaba}, S_{extrap}, T_{poll}\} \to \{//slope, S, T\}$
$V4 = \{\overline{aaba}, S_{eye}, T_{slope}\} \to \{//slope, S, T\}$
$V5 = \{\overline{aaba}, S_{eye}, T_{top}\} \to \{//slope, S, T\}$
$V6 = \{\overline{aaba}, S_{eye}, T_{poll}\} \to \{//slope, S, T\}$

$V7 = \{\overline{abbb}, S_{extrap}, T_{slope}\} \to \{//slope, S, T\}$
$V8 = \{\overline{abbb}, S_{extrap}, T_{top}\} \to \{//slope, S, T\}$
$V9 = \{\overline{abbb}, S_{extrap}, T_{poll}\} \to \{//slope, S, T\}$
$V10 = \{\overline{abbb}, S_{eye}, T_{slope}\} \to \{//slope, S, T\}$
$V11 = \{\overline{abbb}, S_{eye}, T_{top}\} \to \{//slope, S, T\}$
$V12 = \{\overline{abbb}, S_{eye}, T_{poll}\} \to \{//slope, S, T\}$

$V13 = \{\overline{aabb}, S_{extrap}, T_{slope}\} \to \{//slope, S, T\}$
$V14 = \{\overline{aabb}, S_{extrap}, T_{top}\} \to \{//slope, S, T\}$
$V15 = \{\overline{aabb}, S_{extrap}, T_{poll}\} \to \{//slope, S, T\}$
$V16 = \{\overline{aabb}, S_{eye}, T_{slope}\} \to \{//slope, S, T\}$
$V17 = \{\overline{aabb}, S_{eye}, T_{top}\} \to \{//slope, S, T\}$
$V18 = \{\overline{aabb}, S_{eye}, T_{poll}\} \to \{//slope, S, T\}$

$V19 = \{\overline{abba}, S_{extrap}, T_{slope}\} \to \{//slope, S, T\}$
$V20 = \{\overline{abba}, S_{extrap}, T_{top}\} \to \{//slope, S, T\}$
$V21 = \{\overline{abba}, S_{extrap}, T_{poll}\} \to \{//slope, S, T\}$
$V22 = \{\overline{abba}, S_{eye}, T_{slope}\} \to \{//slope, S, T\}$
$V23 = \{\overline{abba}, S_{eye}, T_{top}\} \to \{//slope, S, T\}$
$V24 = \{\overline{abba}, S_{eye}, T_{poll}\} \to \{//slope, S, T\}$

Forehead-Jaw Angle_Slope

$$FJAS = CF * \frac{\|T, T_{int}\|}{\|S, T\|}$$

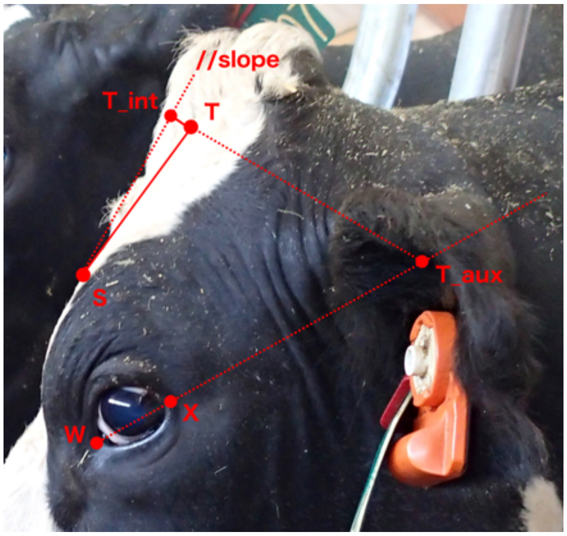

$$CF = \frac{\|T_{int}, T_{aux}\| - \|T, T_{aux}\|}{|\|T_{int}, T_{aux}\| - \|T, T_{aux}\||}$$

$V1 = \{\overline{LM}, S_{extrap}, T_{slope}\} \rightarrow \{//slope, S, T\}$
$V2 = \{\overline{LM}, S_{extrap}, T_{top}\} \rightarrow \{//slope, S, T\}$
$V3 = \{\overline{LM}, S_{extrap}, T_{poll}\} \rightarrow \{//slope, S, T\}$

$V4 = \{\overline{LM}, S_{eye}, T_{slope}\} \rightarrow \{//slope, S, T\}$
$V5 = \{LM, S_{eye}, T_{top}\} \rightarrow \{//slope, S, T\}$
$V6 = \{\overline{LM}, S_{eye}, T_{poll}\} \rightarrow \{//slope, S, T\}$

$V7 = \{\overline{L_{fill}M}, S_{extrap}, T_{slope}\} \rightarrow \{//slope, S, T\}$
$V8 = \{\overline{L_{full}M}, S_{extrap}, T_{top}\} \rightarrow \{//slope, S, T\}$
$V9 = \{\overline{L_{full}M}, S_{extrap}, T_{poll}\} \rightarrow \{//slope, S, T\}$

$V10 = \{\overline{L_{full}M}, S_{eye}, T_{slope}\} \rightarrow \{//slope, S, T\}$
$V11 = \{L_{full}M, S_{eye}, T_{top}\} \rightarrow \{//slope, S, T\}$
$V12 = \{\overline{L_{full}M}, S_{eye}, T_{poll}\} \rightarrow \{//slope, S, T\}$

Forehead Temple Ratio

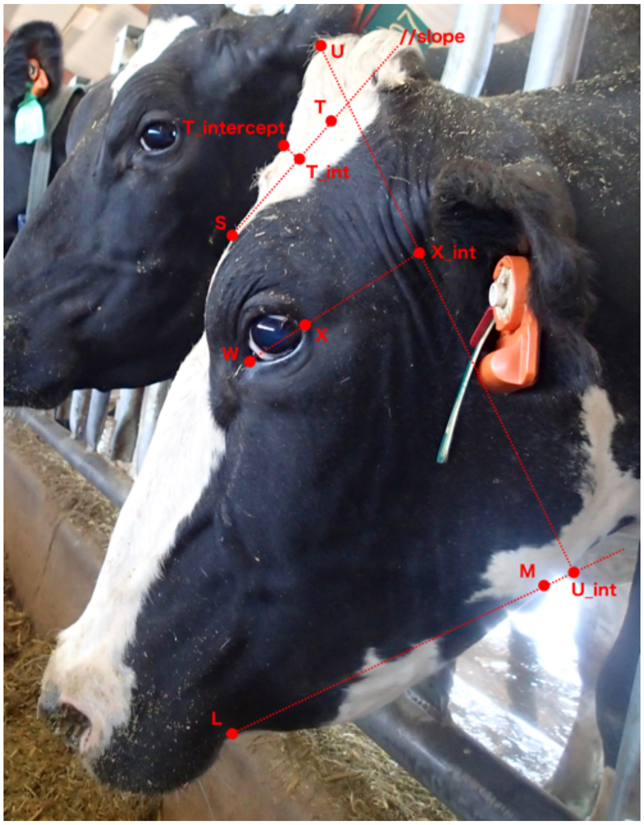

$$FTR = \frac{\|S, T_{int}\|}{\|X, X_{int}\|}$$

$$V1 = \{S_{extrap}, T_{poll}, aa, ba\} \rightarrow \{S, T, W, X\}$$
$$V2 = \{S_{extrap}, T_{poll}, ab, bb\} \rightarrow \{S, T, W, X\}$$
$$V3 = \{S_{extrap}, T_{poll}, ac, bc\} \rightarrow \{S, T, W, X\}$$
$$V4 = \{S_{extrap}, T_{poll}, aa, bb\} \rightarrow \{S, T, W, X\}$$
$$V5 = \{S_{extrap}, T_{poll}, ab, ba\} \rightarrow \{S, T, W, X\}$$

$$V6 = \{S_{extrap}, T_{slope}, aa, ba\} \rightarrow \{S, T, W, X\}$$
$$V7 = \{S_{extrap}, T_{slope}, ab, bb\} \rightarrow \{S, T, W, X\}$$
$$V8 = \{S_{extrap}, T_{slope}, ac, bc\} \rightarrow \{S, T, W, X\}$$
$$V9 = \{S_{extrap}, T_{slope}, aa, bb\} \rightarrow \{S, T, W, X\}$$
$$V10 = \{S_{extrap}, T_{slope}, ab, ba\} \rightarrow \{S, T, W, X\}$$

$$V11 = \{S_{extrap}, T_{top}, aa, ba\} \rightarrow \{S, T, W, X\}$$
$$V12 = \{S_{extrap}, T_{top}, ab, bb\} \rightarrow \{S, T, W, X\}$$
$$V13 = \{S_{extrap}, T_{top}, ac, bc\} \rightarrow \{S, T, W, X\}$$
$$V14 = \{S_{extrap}, T_{top}, aa, bb\} \rightarrow \{S, T, W, X\}$$
$$V15 = \{S_{extrap}, T_{top}, ab, ba\} \rightarrow \{S, T, W, X\}$$

$$V16 = \{S_{eye}, T_{poll}, aa, ba\} \rightarrow \{S, T, W, X\}$$
$$V17 = \{S_{eye}, T_{poll}, ab, bb\} \rightarrow \{S, T, W, X\}$$
$$V18 = \{S_{eye}, T_{poll}, ac, bc\} \rightarrow \{S, T, W, X\}$$
$$V19 = \{S_{eye}, T_{poll}, aa, bb\} \rightarrow \{S, T, W, X\}$$
$$V20 = \{S_{eye}, T_{poll}, ab, ba\} \rightarrow \{S, T, W, X\}$$

$$V21 = \{S_{eye}, T_{slope}, aa, ba\} \rightarrow \{S, T, W, X\}$$
$$V22 = \{S_{eye}, T_{slope}, ab, bb\} \rightarrow \{S, T, W, X\}$$
$$V23 = \{S_{eye}, T_{slope}, ac, bc\} \rightarrow \{S, T, W, X\}$$
$$V24 = \{S_{eye}, T_{slope}, aa, bb\} \rightarrow \{S, T, W, X\}$$
$$V25 = \{S_{eye}, T_{slope}, ab, ba\} \rightarrow \{S, T, W, X\}$$

$$V26 = \{S_{eye}, T_{top}, aa, ba\} \rightarrow \{S, T, W, X\}$$
$$V27 = \{S_{eye}, T_{top}, ab, bb\} \rightarrow \{S, T, W, X\}$$
$$V28 = \{S_{eye}, T_{top}, ac, bc\} \rightarrow \{S, T, W, X\}$$
$$V29 = \{S_{eye}, T_{top}, aa, bb\} \rightarrow \{S, T, W, X\}$$
$$V30 = \{S_{eye}, T_{top}, ab, ba\} \rightarrow \{S, T, W, X\}$$

Forehead-Topline Angle_Slope

$$FTAS = CF * \frac{\|T, T_{int}\|}{\|S, T\|}$$

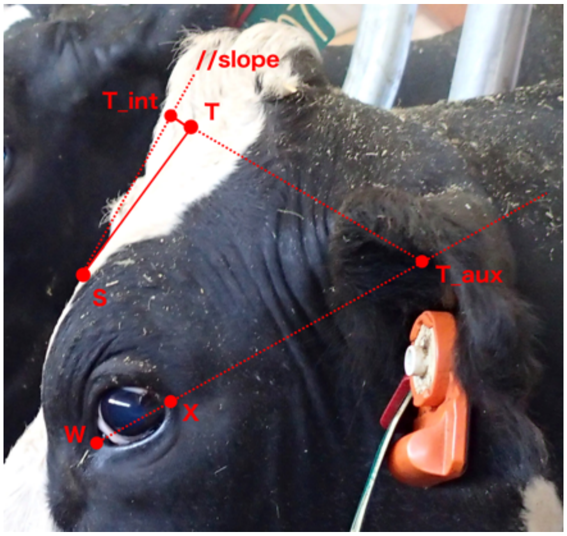

$$CF = \frac{\|T_{int}, T_{aux}\| - \|T, T_{aux}\|}{|\|T_{int}, T_{aux}\| - \|T, T_{aux}\||}$$

$V1 = \{\overline{WH}, S_{extrap}, T_{slope}\} \rightarrow \{//slope, S, T\}$
$V2 = \{\overline{WH}, S_{extrap}, T_{top}\} \rightarrow \{//slope, S, T\}$
$V3 = \{\overline{WH}, S_{extrap}, T_{poll}\} \rightarrow \{//slope, S, T\}$

$V4 = \{\overline{WH}, S_{eye}, T_{slope}\} \rightarrow \{//slope, S, T\}$
$V5 = \{\overline{WH}, S_{eye}, T_{top}\} \rightarrow \{//slope, S, T\}$
$V6 = \{\overline{WH}, S_{eye}, T_{poll}\} \rightarrow \{//slope, S, T\}$

Forehead Topline Length Ratio

$$FTLR = \frac{\|S, T_{int}\|}{\|W, H\|}$$

$V1 = \{S_{extrap}, T_{slope}\} \rightarrow \{S, T\}$
$V2 = \{S_{extrap}, T_{top}\} \rightarrow \{S, T\}$
$V3 = \{S_{extrap}, T_{poll}\} \rightarrow \{S, T\}$

$V4 = \{S_{eye}, T_{slope}\} \rightarrow \{S, T\}$
$V5 = \{S_{eye}, T_{top}\} \rightarrow \{S, T\}$
$V6 = \{S_{eye}, T_{poll}\} \rightarrow \{S, T\}$

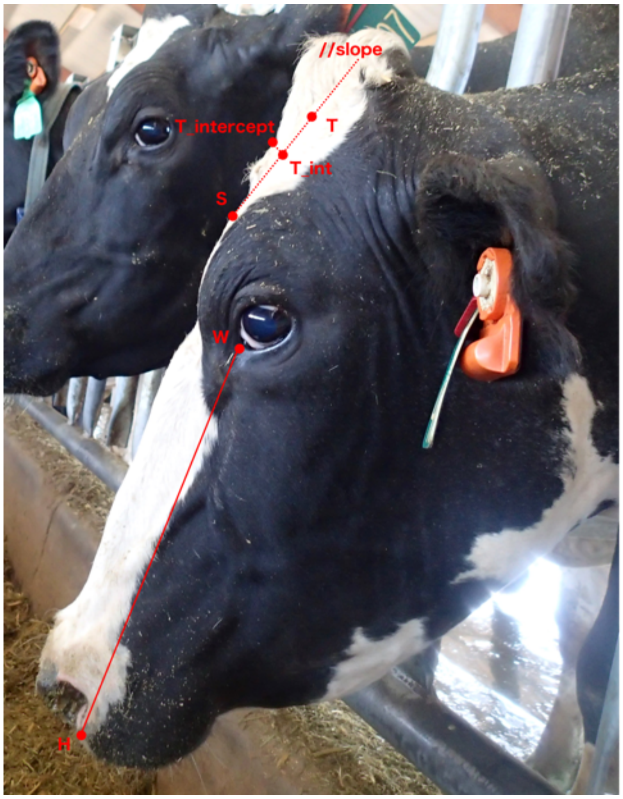

Forehead Width-to-Length Proportion

$$FWLP = \frac{\|X, U_{int}\|}{\|U_{int}, T_{int}\|}$$

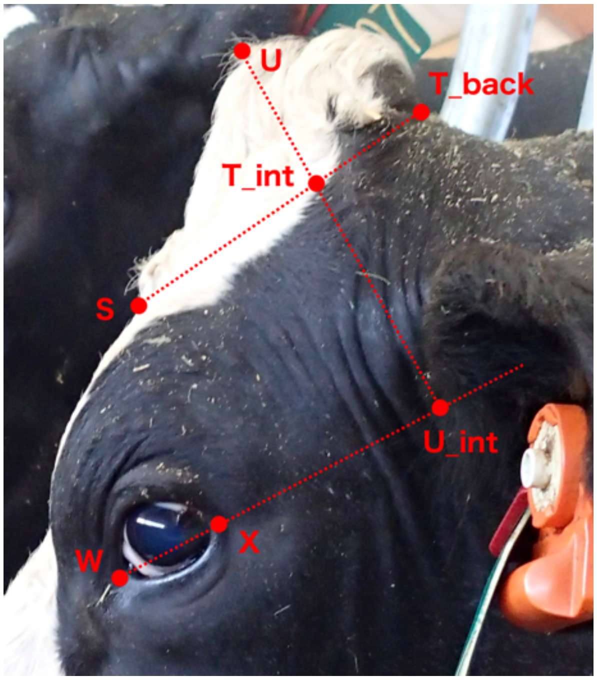

$V1 = \{S_{extrap}, T_{slope}, aa, ba\} \rightarrow \{S, T, W, X\}$
$V2 = \{S_{extrap}, T_{top}, aa, ba\} \rightarrow \{S, T, W, X\}$
$V3 = \{S_{extrap}, T_{poll}, aa, ba\} \rightarrow \{S, T, W, X\}$
$V4 = \{S_{eye}, T_{slope}, aa, ba\} \rightarrow \{S, T, W, X\}$
$V5 = \{S_{eye}, T_{top}, aa, ba\} \rightarrow \{S, T, W, X\}$
$V6 = \{S_{eye}, T_{poll}, aa, ba\} \rightarrow \{S, T, W, X\}$

$V7 = \{S_{extrap}, T_{slope}, ab, bb\} \rightarrow \{S, T, W, X\}$
$V8 = \{S_{extrap}, T_{top}, ab, bb\} \rightarrow \{S, T, W, X\}$
$V9 = \{S_{extrap}, T_{poll}, ab, bb\} \rightarrow \{S, T, W, X\}$
$V10 = \{S_{eye}, T_{slope}, ab, bb\} \rightarrow \{S, T, W, X\}$
$V11 = \{S_{eye}, T_{top}, ab, bb\} \rightarrow \{S, T, W, X\}$
$V12 = \{S_{eye}, T_{poll}, ab, bb\} \rightarrow \{S, T, W, X\}$

$V13 = \{S_{extrap}, T_{slope}, ac, bc\} \rightarrow \{S, T, W, X\}$
$V14 = \{S_{extrap}, T_{top}, ac, bc\} \rightarrow \{S, T, W, X\}$
$V15 = \{S_{extrap}, T_{poll}, ac, bc\} \rightarrow \{S, T, W, X\}$
$V16 = \{S_{eye}, T_{slope}, ac, bc\} \rightarrow \{S, T, W, X\}$
$V17 = \{S_{eye}, T_{top}, ac, bc\} \rightarrow \{S, T, W, X\}$
$V18 = \{S_{eye}, T_{poll}, ac, bc\} \rightarrow \{S, T, W, X\}$

Forehead Zygomatic Angle

$$FZA = CF * \frac{\|T_{top}, T_{int}\|}{\|S, T_{int}\|}$$

$$CF = \frac{\|T_{top}, T_{aux}\| - \|T_{int}, T_{aux}\|}{\left|\|T_{top}, T_{aux}\| - \|T_{int}, T_{aux}\|\right|}$$

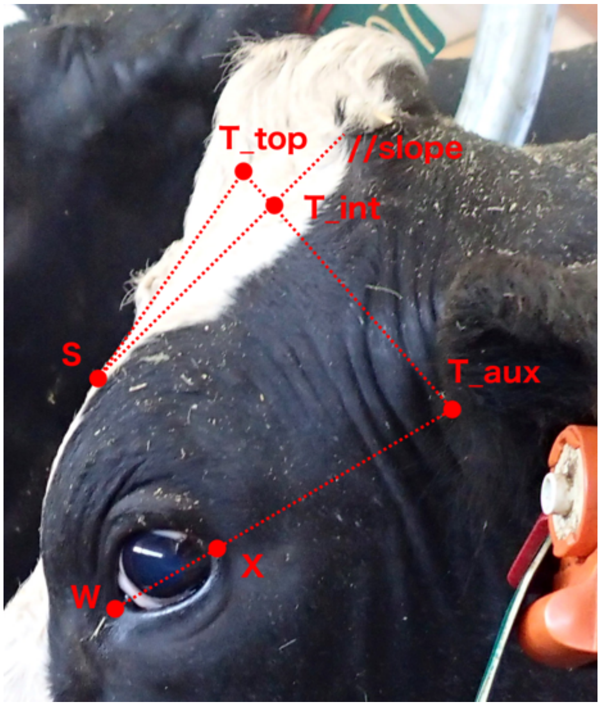

$V1 = \{S_{extrap} T_{top}, aa, ba\} \rightarrow \{//slope, W, X\}$
$V2 = \{S_{extrap}, T_{poll}, aa, ba\} \rightarrow \{//slope, W, X\}$
$V3 = \{S_{eye}, T_{slope}, aa, ba\} \rightarrow \{//slope, W, X\}$
$V4 = \{S_{eye}, T_{poll}, aa, ba\} \rightarrow \{//slope, W, X\}$

$V5 = \{S_{extrap}, T_{slope}, ab, bb\} \rightarrow \{//slope, W, X\}$
$V6 = \{S_{extrap}, T_{poll}, ab, bb\} \rightarrow \{//slope, W, X\}$
$V7 = \{S_{eye}, T_{slope}, ab, bb\} \rightarrow \{//slope, W, X\}$
$V8 = \{S_{eye}, T_{poll}, ab, bb\} \rightarrow \{//slope, W, X\}$

$V9 = \{S_{extrap}, T_{slope}, ac, bc\} \rightarrow \{//slope, W, X\}$
$V10 = \{S_{extrap}, T_{poll}, ac, bc\} \rightarrow \{//slope, W, X\}$
$V11 = \{S_{eye}, T_{slope}, ac, bc\} \rightarrow \{//slope, W, X\}$
$V12 = \{S_{eye}, T_{poll}, ac, bc\} \rightarrow \{//slope, W, X\}$

Forehead-Poll Length Ratio

$$FPLR = \frac{\|S, T_{mid}\|}{\|S, T_{int}\|}$$

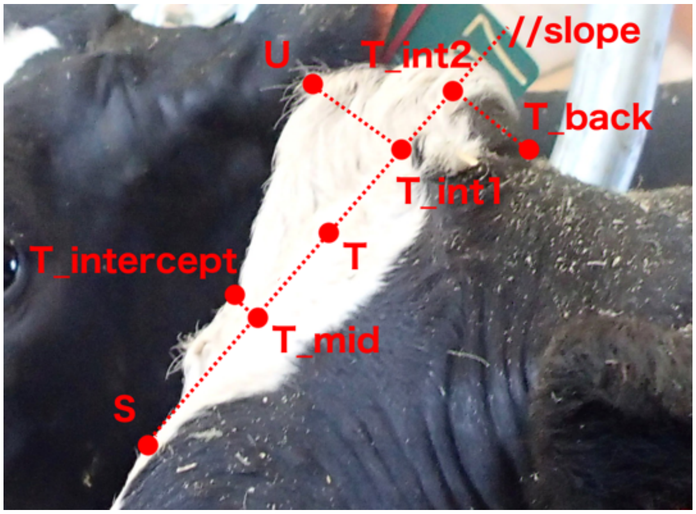

$V1 = \{S_{extrap}, T_{int1}, T_{slope}\} \rightarrow \{S, T_{int}, T\}$
$V2 = \{S_{extrap}, T_{int1}, T_{top}\} \rightarrow \{S, T_{int}, T\}$
$V3 = \{S_{extrap}, T_{int1}, T_{poll}\} \rightarrow \{S, T_{int}, T\}$

$V4 = \{S_{extrap}, T_{int2}, T_{slope}\} \rightarrow \{S, T_{int}, T\}$
$V5 = \{S_{extrap}, T_{int2}, T_{top}\} \rightarrow \{S, T_{int}, T\}$
$V6 = \{S_{extrap}, T_{int2}, T_{poll}\} \rightarrow \{S, T_{int}, T\}$

$V7 = \{S_{eye}, T_{int1}, T_{slope}\} \rightarrow \{S, T_{int}, T\}$
$V8 = \{S_{eye}, T_{int1}, T_{top}\} \rightarrow \{S, T_{int}, T\}$
$V9 = \{S_{eye}, T_{int1}, T_{poll}\} \rightarrow \{S, T_{int}, T\}$

$V10 = \{S_{eye}, T_{int2}, T_{slope}\} \rightarrow \{S, T_{int}, T\}$
$V11 = \{S_{eye}, T_{int2}, T_{top}\} \rightarrow \{S, T_{int}, T\}$
$V12 = \{S_{eye}, T_{int2}, T_{poll}\} \rightarrow \{S, T_{int}, T\}$

Jaw Angle_Slope

$$JAS = \frac{\|L, L_{int}\| - \|M, M_{int}\|}{\|L_{int}, M_{int}\|}$$

$V1 = \{aa, L\} \rightarrow \{W, L\}$
$V2 = \{ab, L\} \rightarrow \{W, L\}$
$V3 = \{ac, L\} \rightarrow \{W, L\}$

$V4 = \{aa, L_{full}\} \rightarrow \{W, L\}$
$V5 = \{ab, L_{full}\} \rightarrow \{W, L\}$
$V6 = \{ac, L_{full}\} \rightarrow \{W, L\}$

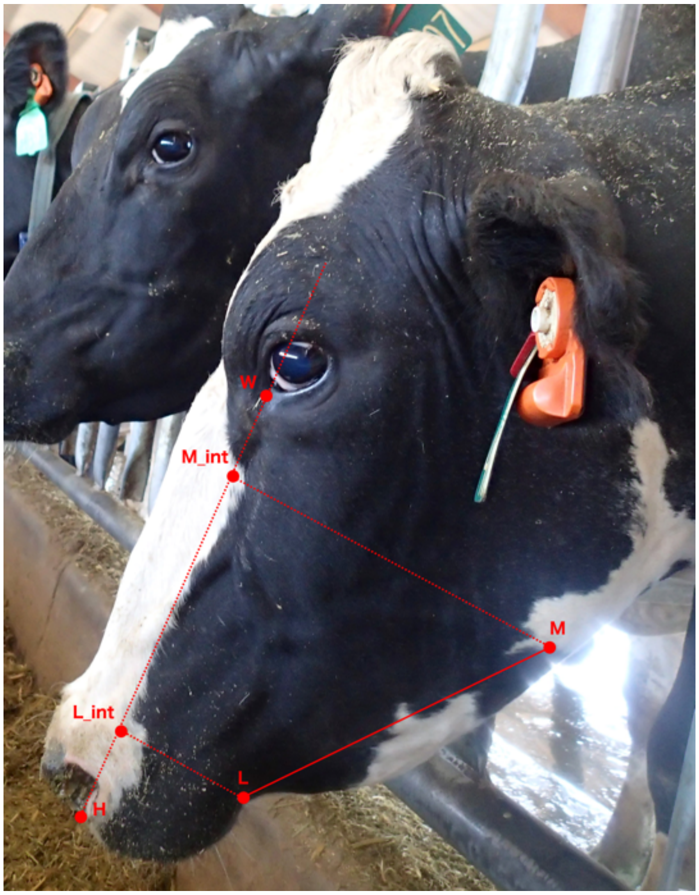

Jowl-Jaw Length Proportion

$$JJLP = \frac{\|C_{int}, U_{int}\|}{\|L, C_{int}\|}$$

$$V1 = \{C_{extrap}, U_{int1}, L\} \rightarrow \{C, U_{int}, L\}$$
$$V2 = \{C_{extrap}, U_{int2}, L\} \rightarrow \{C, U_{int}, L\}$$
$$V3 = \{C\_eye, U_{int1}, L\} \rightarrow \{C, U_{int}, L\}$$
$$V4 = \{C\_eye, U_{int2}, L\} \rightarrow \{C, U_{int}, L\}$$

$$V5 = \{C_{extrap}, U_{int1}, L_{full}\} \rightarrow \{C, U_{int}, L\}$$
$$V6 = \{C_{extrap}, U_{int2}, L_{full}\} \rightarrow \{C, U_{int}, L\}$$
$$V7 = \{C\_eye, U_{int1}, L_{full}\} \rightarrow \{C, U_{int}, L\}$$
$$V8 = \{C\_eye, U_{int2}, L_{full}\} \rightarrow \{C, U_{int}, L\}$$

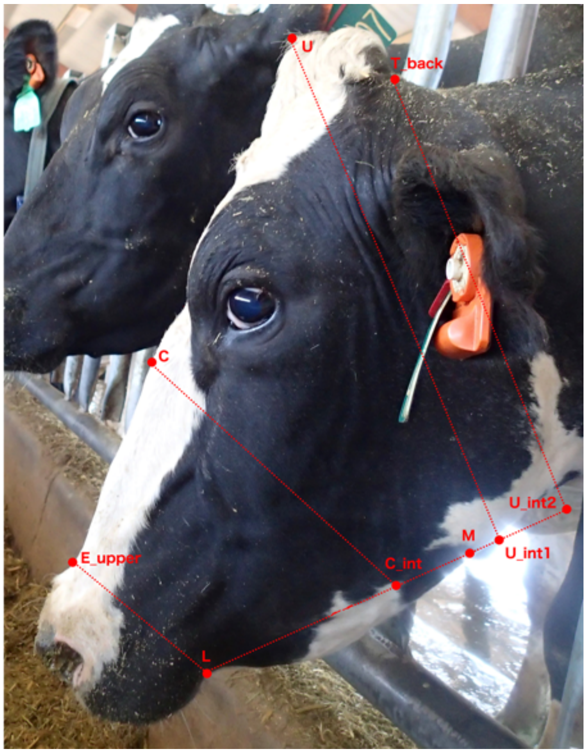

Jowl Length Proportion

$$JLP = \frac{\|D_{int}, U_{int}\|}{\|L, D_{int}\|}$$

$$V1 = \{U_{int1}, L\} \rightarrow \{U_{int}, L\}$$
$$V2 = \{U_{int2}, L\} \rightarrow \{U_{int}, L\}$$

$$V3 = \{U_{int1}, L_{full}\} \rightarrow \{U_{int}, L\}$$
$$V4 = \{U_{int2}, L_{full}\} \rightarrow \{U_{int}, L\}$$

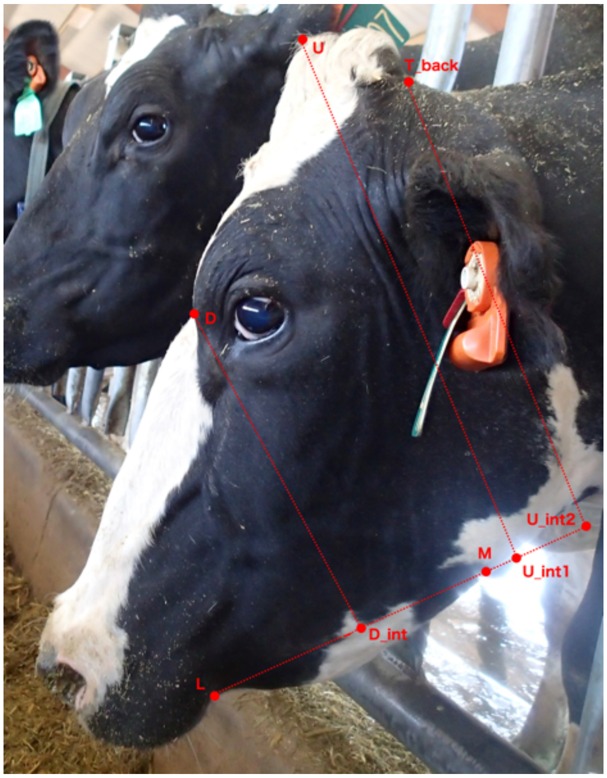

Midface Thickness Proportion

$$MTP = \frac{\|C, C_{int2}\|}{\|C_{int2}, E_{int5}\|}$$

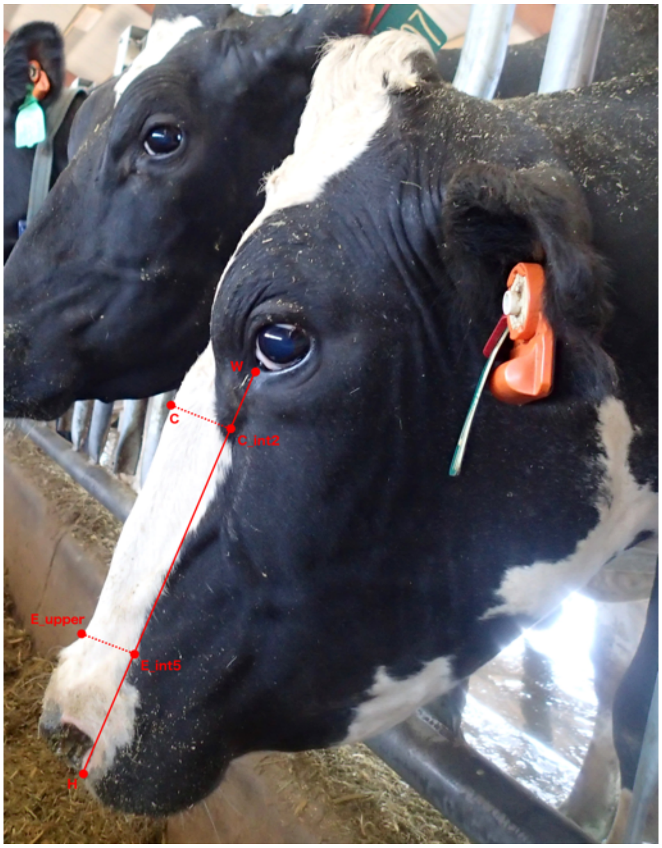

$$V1 = \{C_{extrap}\} \rightarrow \{C\}$$
$$V2 = \{C_{eye}\} \rightarrow \{C\}$$

Nasion Thickness Proportion

$$NsTP = \frac{\|G, G_{int}\|}{\|Y, X\|}$$

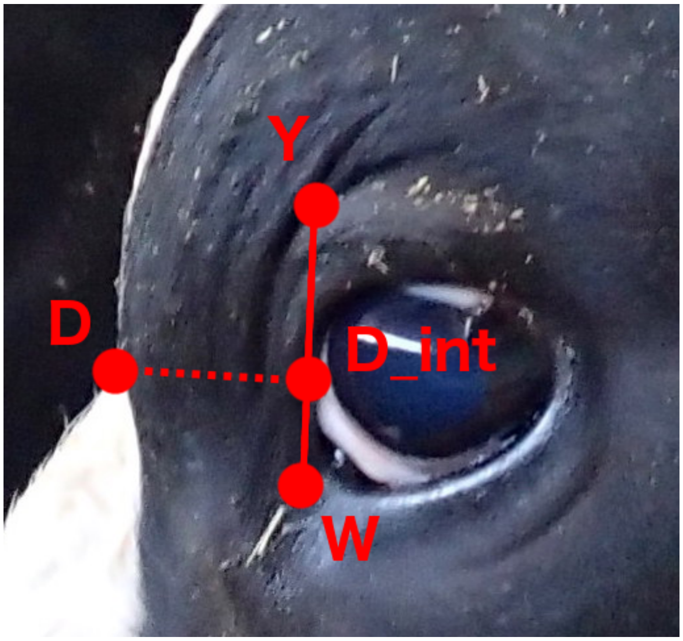

$$V1 = \{ca, aa\} \rightarrow \{Y, W\}$$
$$V2 = \{ca, ab\} \rightarrow \{Y, W\}$$
$$V3 = \{ca, ac\} \rightarrow \{Y, W\}$$

$$V4 = \{cb, aa\} \rightarrow \{Y, W\}$$
$$V5 = \{cb, ab\} \rightarrow \{Y, W\}$$
$$V6 = \{cb, ac\} \rightarrow \{Y, W\}$$

$$V7 = \{cc, aa\} \rightarrow \{Y, W\}$$
$$V8 = \{cc, ab\} \rightarrow \{Y, W\}$$
$$V9 = \{cc, ac\} \rightarrow \{Y, W\}$$

Overall Eye Angle_Slope

$$OEAS = \frac{\|W, W_{int}\|}{\|W, X\|}$$

$V1 = \{aa, ba\} \rightarrow \{W, X\}$
$V2 = \{ab, bb\} \rightarrow \{W, X\}$
$V3 = \{ac, bc\} \rightarrow \{W, X\}$
$V4 = \{aa, bb\} \rightarrow \{W, X\}$
$V5 = \{ab, ba\} \rightarrow \{W, X\}$

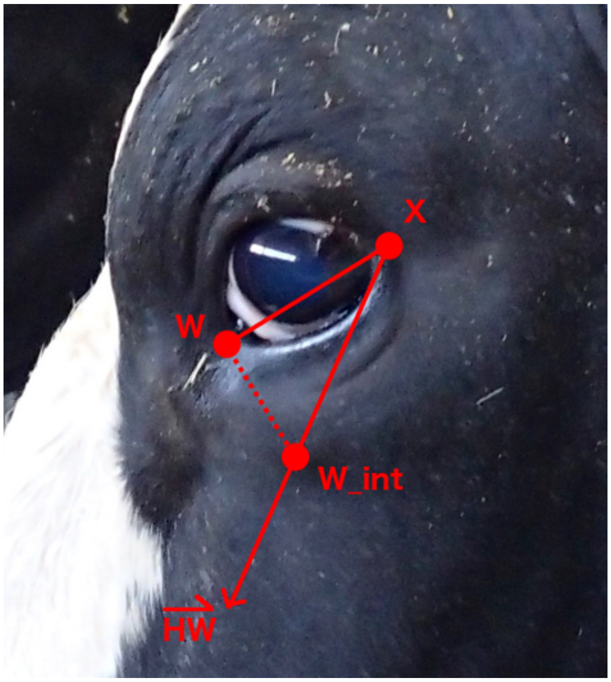

Poll Depth Proportion_Height

$$PDPH = CF * \frac{\|T_{back}, T_{int}\|}{\|U, T_{mid}\|}$$

$$CF = \frac{\|T_{int}, T_{aux}\| - \|T_{back}, T_{aux}\|}{|\|T_{int}, T_{aux}\| - \|T_{back}, T_{aux}\||}$$

$V1 = \{S_{extrap}, T_{slope}\} \rightarrow \{S, T\}$
$V2 = \{S_{extrap}, T_{top}\} \rightarrow \{S, T\}$
$V3 = \{S_{extrap}, T_{poll}\} \rightarrow \{S, T\}$

$V4 = \{S_{eye}, T_{slope}\} \rightarrow \{S, T\}$
$V5 = \{S_{eye}, T_{top}\} \rightarrow \{S, T\}$
$V6 = \{S_{eye}, T_{poll}\} \rightarrow \{S, T\}$

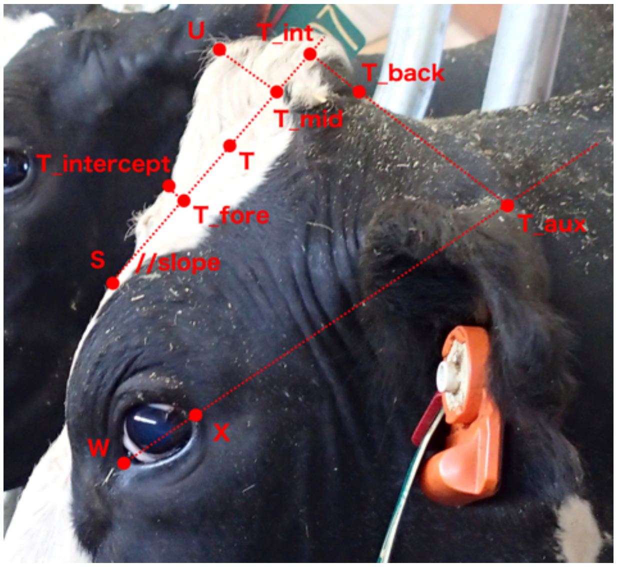

Poll Depth Proportion_Length

$$PDPL = CF * \frac{\|T_{back}, T_{int}\|}{\|T_{fore}, T_{int}\|}$$

$$CF = \frac{\|T_{int}, T_{aux}\| - \|T_{back}, T_{aux}\|}{|\|T_{int}, T_{aux}\| - \|T_{back}, T_{aux}\||}$$

$V1 = \{S_{extrap}, T_{slope}\} \rightarrow \{S, T\}$
$V2 = \{S_{extrap}, T_{top}\} \rightarrow \{S, T\}$
$V3 = \{S_{extrap}, T_{poll}\} \rightarrow \{S, T\}$

$V4 = \{S_{eye}, T_{slope}\} \rightarrow \{S, T\}$
$V5 = \{S_{eye}, T_{top}\} \rightarrow \{S, T\}$
$V6 = \{S_{eye}, T_{poll}\} \rightarrow \{S, T\}$

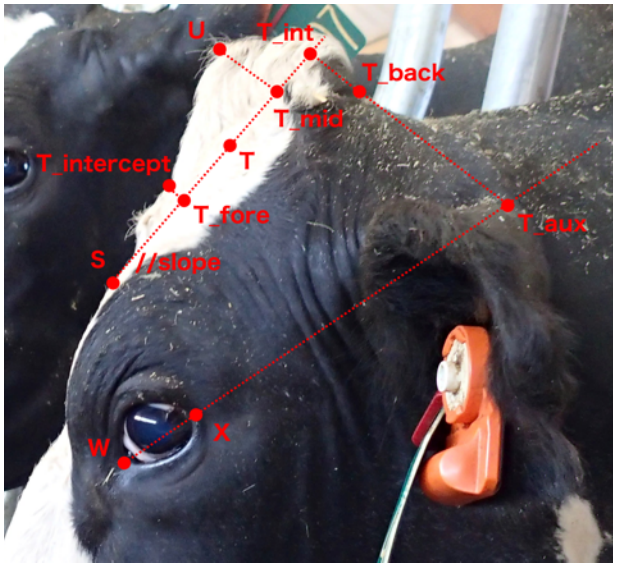

Poll Height Point Proportion

$$PHPP = \frac{\left\| T_{fore}, T_{mid} \right\|}{\left\| T_{fore}, T_{int} \right\|}$$

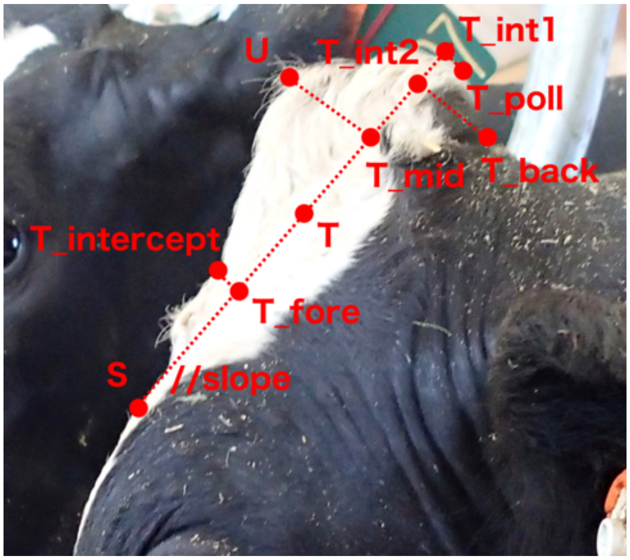

$V1 = \{S_{extrap}, T_{int1}, T_{slope}\} \rightarrow \{S, T_{int}, T\}$
$V2 = \{S_{extrap}, T_{int1}, T_{top}\} \rightarrow \{S, T_{int}, T\}$
$V3 = \{S_{extrap}, T_{int1}, T_{poll}\} \rightarrow \{S, T_{int}, T\}$

$V4 = \{S_{extrap}, T_{int2}, T_{slope}\} \rightarrow \{S, T_{int}, T\}$
$V5 = \{S_{extrap}, T_{int2}, T_{top}\} \rightarrow \{S, T_{int}, T\}$
$V6 = \{S_{extrap}, T_{int2}, T_{poll}\} \rightarrow \{S, T_{int}, T\}$

$V7 = \{S_{eye}, T_{int1}, T_{slope}\} \rightarrow \{S, T_{int}, T\}$
$V8 = \{S_{eye}, T_{int1}, T_{top}\} \rightarrow \{S, T_{int}, T\}$
$V9 = \{S_{eye}, T_{int1}, T_{poll}\} \rightarrow \{S, T_{int}, T\}$

$V10 = \{S_{eye}, T_{int2}, T_{slope}\} \rightarrow \{S, T_{int}, T\}$
$V11 = \{S_{eye}, T_{int2}, T_{top}\} \rightarrow \{S, T_{int}, T\}$
$V12 = \{S_{eye}, T_{int2}, T_{poll}\} \rightarrow \{S, T_{int}, T\}$

Poll Height Proportion

$$PHP = \frac{\|U, T_{mid}\|}{\|T_{fore}, T_{int}\|}$$

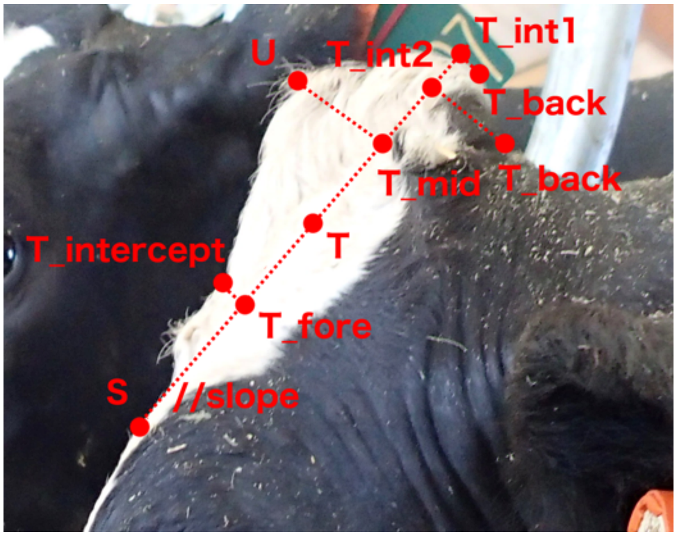

$V1 = \{S_{extrap}, T_{int1}, T_{slope}\} \rightarrow \{S, T_{int}, T\}$
$V2 = \{S_{extrap}, T_{int1}, T_{top}\} \rightarrow \{S, T_{int}, T\}$
$V3 = \{S_{extrap}, T_{int1}, T_{poll}\} \rightarrow \{S, T_{int}, T\}$

$V4 = \{S_{extrap}, T_{int2}, T_{slope}\} \rightarrow \{S, T_{int}, T\}$
$V5 = \{S_{extrap}, T_{int2}, T_{top}\} \rightarrow \{S, T_{int}, T\}$
$V6 = \{S_{extrap}, T_{int2}, T_{poll}\} \rightarrow \{S, T_{int}, T\}$

$V7 = \{S_{eye}, T_{int1}, T_{slope}\} \rightarrow \{S, T_{int}, T\}$
$V8 = \{S_{eye}, T_{int1}, T_{top}\} \rightarrow \{S, T_{int}, T\}$
$V9 = \{S_{eye}, T_{int1}, T_{poll}\} \rightarrow \{S, T_{int}, T\}$

$V10 = \{S_{eye}, T_{int2}, T_{slope}\} \rightarrow \{S, T_{int}, T\}$
$V11 = \{S_{eye}, T_{int2}, T_{top}\} \rightarrow \{S, T_{int}, T\}$
$V12 = \{S_{eye}, T_{int2}, T_{poll}\} \rightarrow \{S, T_{int}, T\}$

Chin Thickness Point Proportion

$$CTPP = \frac{\|A, Q_{aux1}\|}{\|A, L\|}$$

$$V1 = \{A_{extrap}, L\} \rightarrow \{A, L\}$$
$$V2 = \{A_{extrap}, L_{full}\} \rightarrow \{A, L\}$$
$$V3 = \{A_{eye}, L\} \rightarrow \{A, L\}$$
$$V4 = \{A_{eye}, L_{full}\} \rightarrow \{A, L\}$$

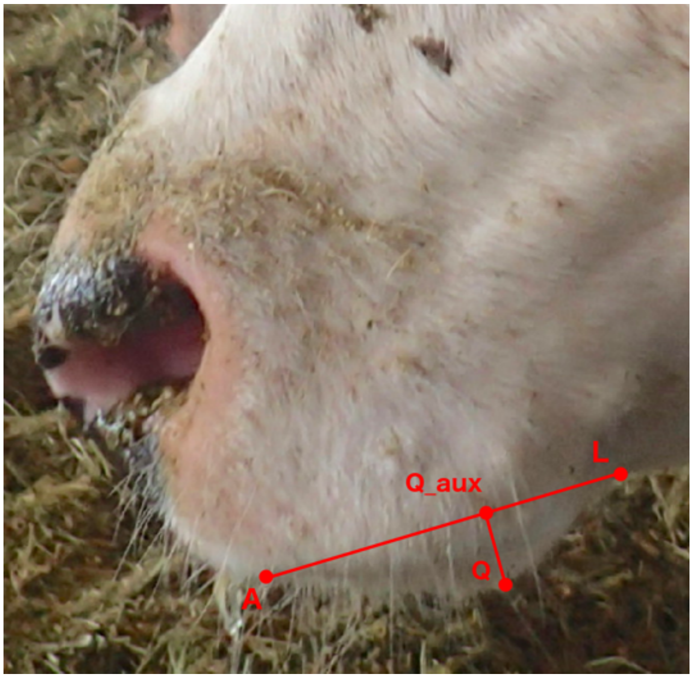

Chin Thickness Proportion

$$CTP = \frac{\|Q, Q_{aux1}\|}{\|A, L\|}$$

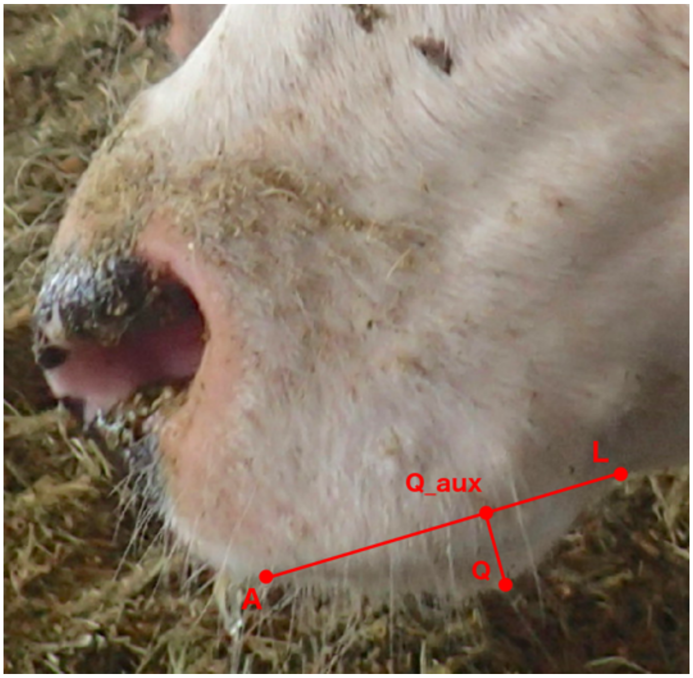

$$V1 = \{A_{extrap}, L\} \rightarrow \{A, L\}$$
$$V2 = \{A_{extrap}, L_{full}\} \rightarrow \{A, L\}$$
$$V3 = \{A_{eye}, L\} \rightarrow \{A, L\}$$
$$V4 = \{A_{eye}, L_{full}\} \rightarrow \{A, L\}$$

Chin-to-Lip Thickness Ratio

$$CLTR = \frac{\|Q, Q_{aux3}\|}{\|Q, H_{aux4}\|}$$

$$V1 = \{A_{extrap}, L\} \rightarrow \{A, L\}$$
$$V2 = \{A_{eye}, L_{full}\} \rightarrow \{A, L\}$$

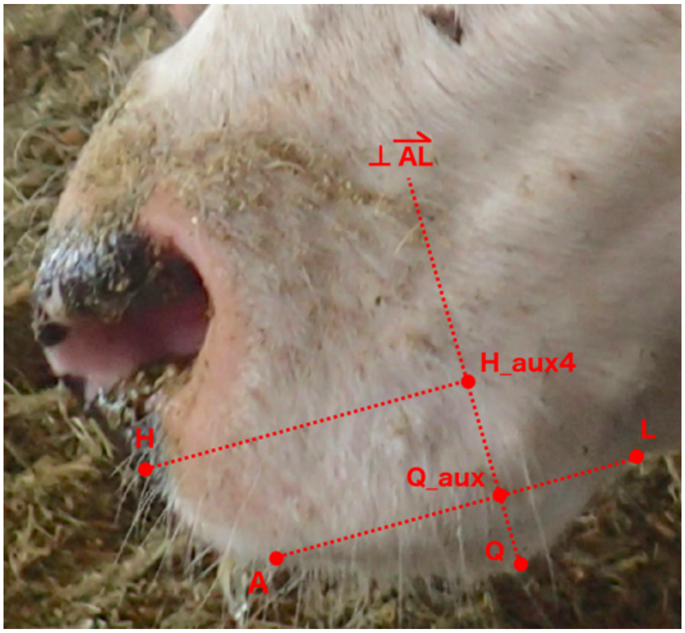

Mouth Eye-to-Extrap Offset _Height

$$MEEOH = CF * \frac{\|Ax_{aux}, Ay_{aux}\|}{\|H_{aux}, L_{aux}\|}$$

$$CF = \frac{\|H_{aux}, Ay_{aux}\| - \|H_{aux}, Ax_{aux}\|}{|\|H_{aux}, Ay_{aux}\| - \|H_{aux}, Ax_{aux}\||}$$

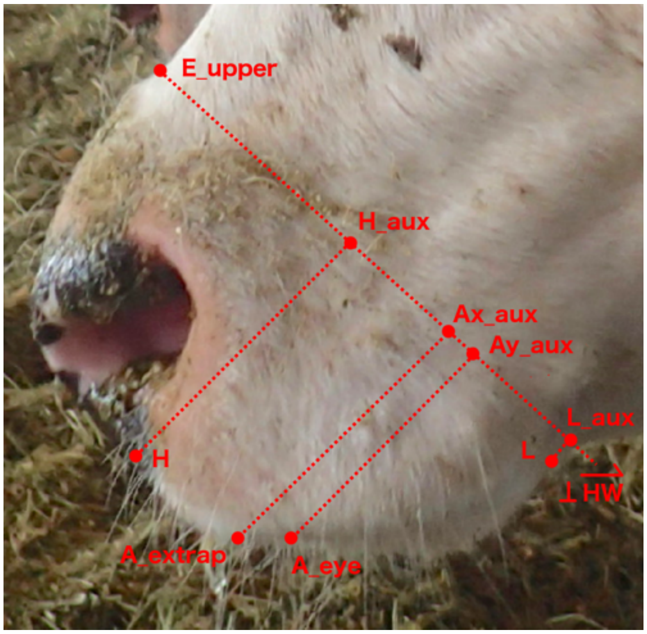

Mouth Eye-to-Extrap Offset _Length

$$MEEOL = CF * \frac{\|H, Ay_{aux2}\|}{\|H, L\_aux2\|}$$

$$CF = \frac{\|Ay_{aux2}, L_{aux2}\| - \|H, L_{aux2}\|}{\mathrm{abs}(|\|Ay_{aux2}, L_{aux2}\| - \|H, L_{aux2}\||)}$$

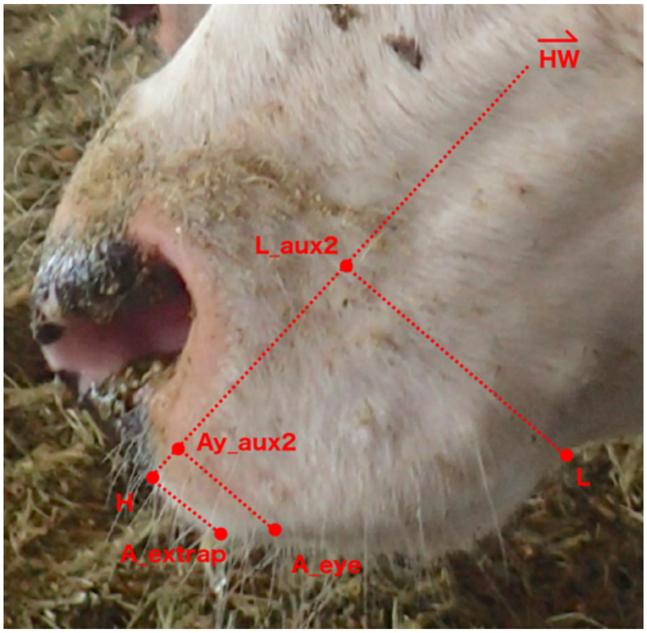

Mouth Thickness Proportion

$$MTP = \frac{\|H_{aux}, Ax_{aux}\|}{\|Ax_{aux}, E\_upper\|}$$

$$V1 = \{A_{extrap}, L\} \rightarrow \{A, L\}$$
$$V2 = \{A_{eye}, L_{full}\} \rightarrow \{A, L\}$$

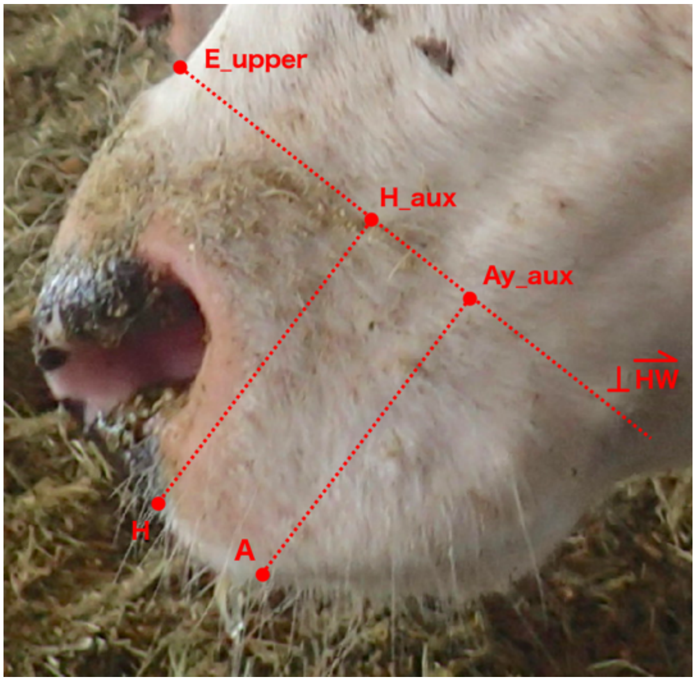

Nostril Depth Point Proportion

$$NDPP = \frac{\|I, j\_int\|}{\overline{HI}}$$

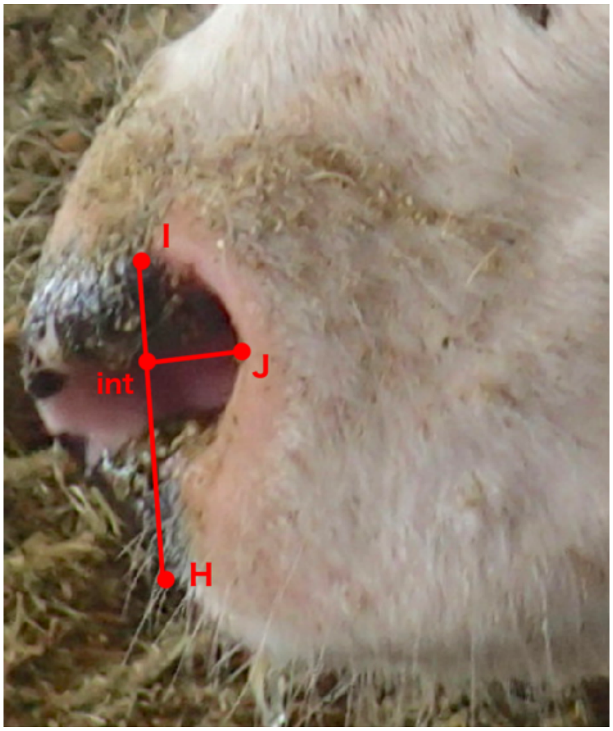

Nostril Depth Proportion_Linear

$$NDP = \frac{\|J, J\_int\|}{\|H, I\|}$$

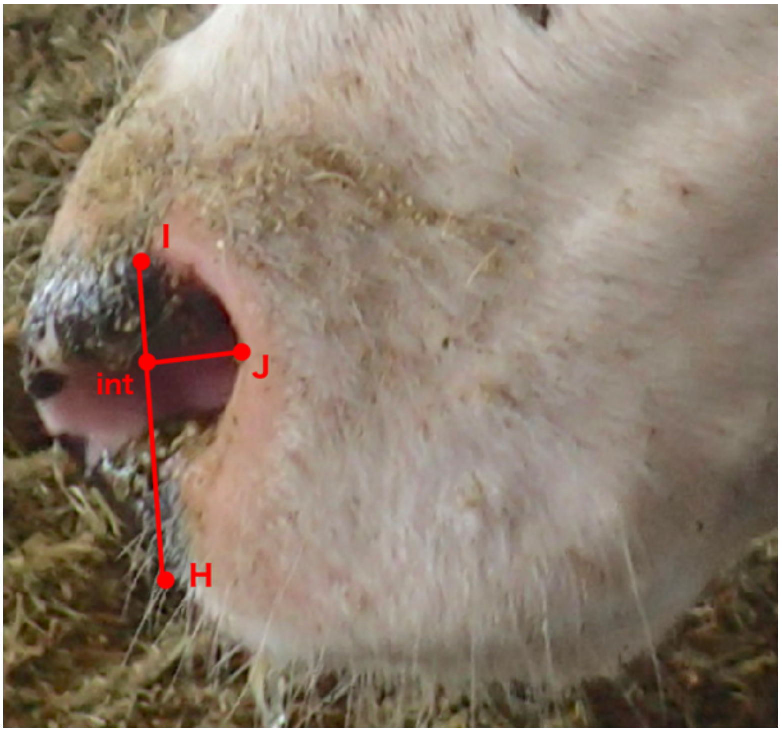

Nostril Flare Point Proportion – Lower Back

$$NFPP\_LB = \frac{\|jh\_int, H\|}{\|J, H\|}$$

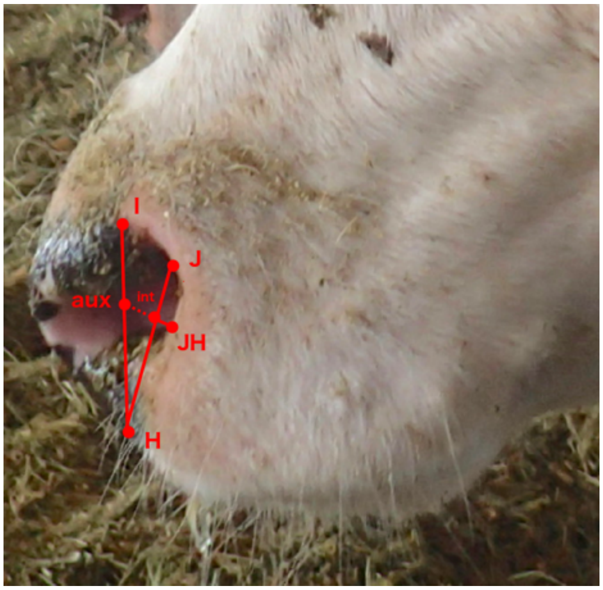

Nostril Flare Proportion – Upper Back

$$NFP\_UB = CF * \frac{\|ji, ji\_int\|}{\|J, I\|}$$

$$CF = \frac{\|ji\_aux, ji\| - \|ji\_aux, ji\_int\|}{\left\| \|ji\_aux, ji\| - \|ji\_aux, ji\_int\| \right\|}$$

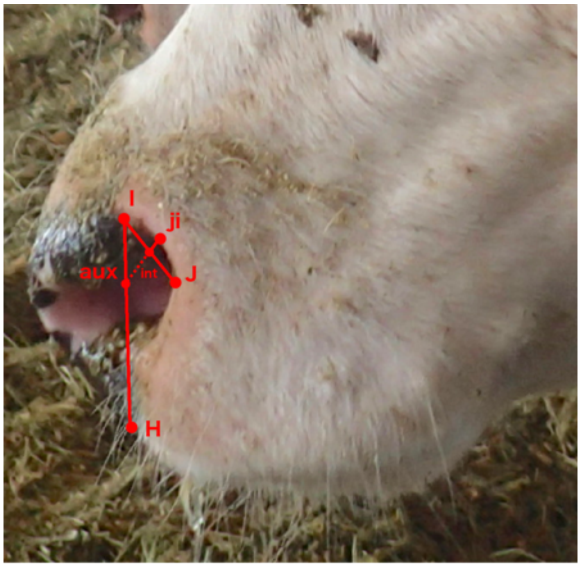

Nostril Flare Point Proportion – Upper Front

$$NFPP\_UF = \frac{\|I, ki\_int\|}{\|K, I\|}$$

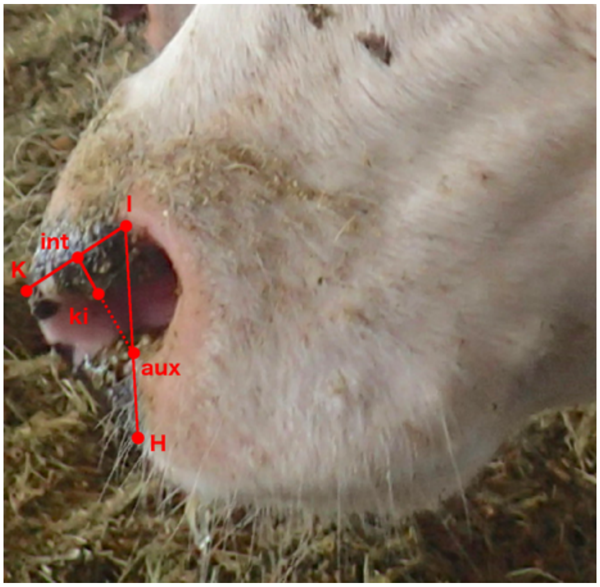

Nostril Flare Point Proportion – Lower Front

$$NFPP\_LF = \frac{\|H, kh\_int\|}{\|K, H\|}$$

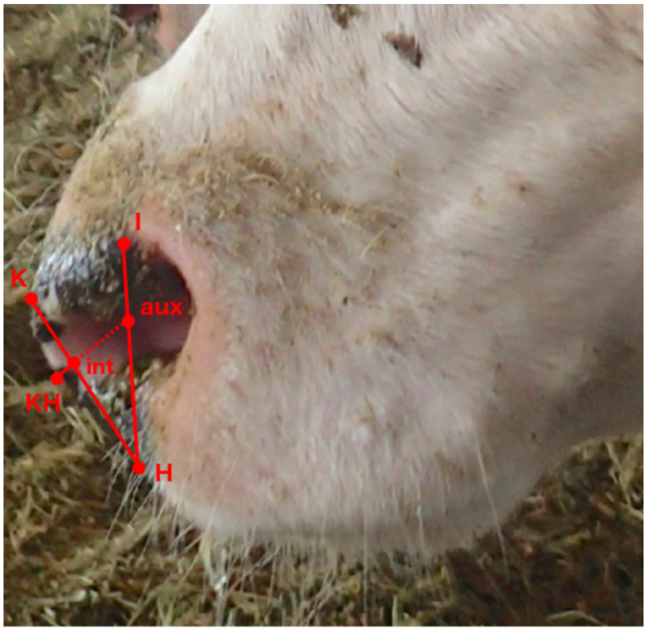

Nostril Flare Proportion – Lower Back

$$NFP\_LB = CF * \frac{\|jh, jh\_int\|}{\|J, H\|}$$

$$CF = \frac{\|jh\_aux, jh\| - \|jh\_aux, jh\_int\|}{\big|\|jh\_aux, jh\| - \|jh\_aux, jh\_int\|\big|}$$

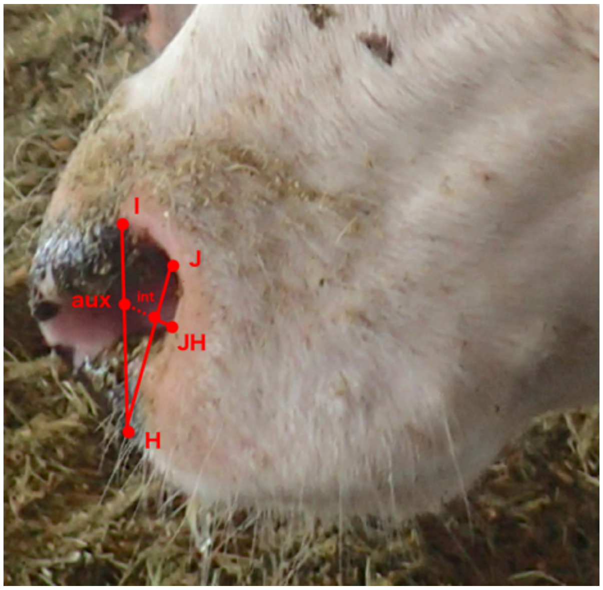

Nostril Flare Proportion – Lower Front

$$NFP\_LF = CF * \frac{\|kh, kh\_int\|}{\|K, H\|}$$

$$CF = \frac{\|kh\_aux, kh\_int\| - \|kh\_aux, kh\|}{\left\| \|kh\_aux, kh\_int\| - \|kh\_aux, kh\| \right\|}$$

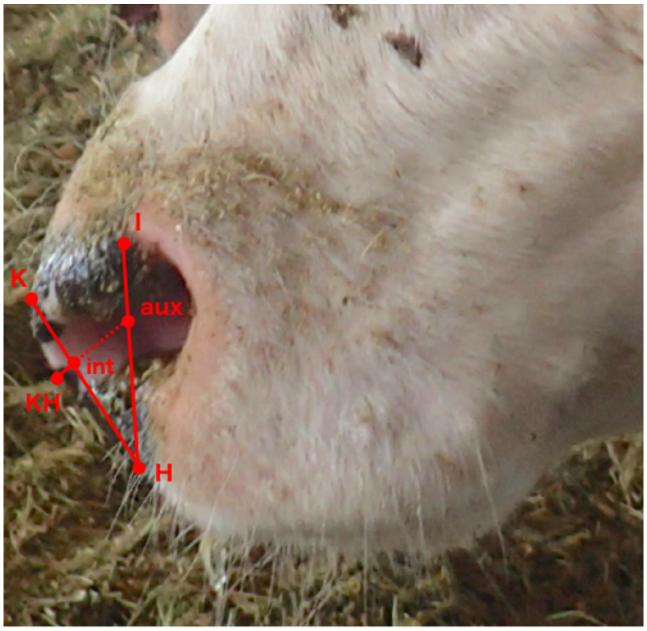

Nostril Flare Proportion – Upper Back

$$NFP\_UB = CF * \frac{\|ji, ji\_int\|}{\|J, I\|}$$

$$CF = \frac{\|ji\_aux, ji\| - \|ji\_aux, ji\_int\|}{\left\|\|ji\_aux, ji\| - \|ji\_aux, ji\_int\|\right\|}$$

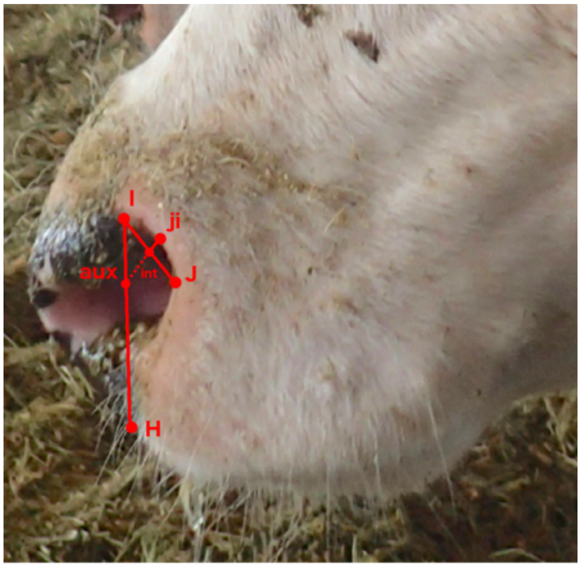

Nostril Flare Proportion – Upper Front

$$NFP\_UF = CF * \frac{\|ki, ki\_int\|}{\|K, I\|}$$

$$CF = \frac{\|ki\_aux, ki\_int\| - \|ki, ki\_aux\|}{\left\| \|ki\_aux, ki\_int\| - \|ki, ki\_aux\| \right\|}$$

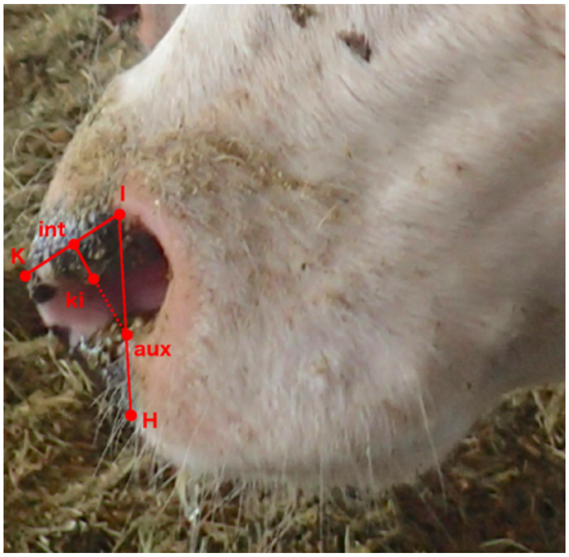

__Nostril Depth Point Proportion__

$$NDPP = \frac{\|I, j\_int\|}{\overline{HI}}$$

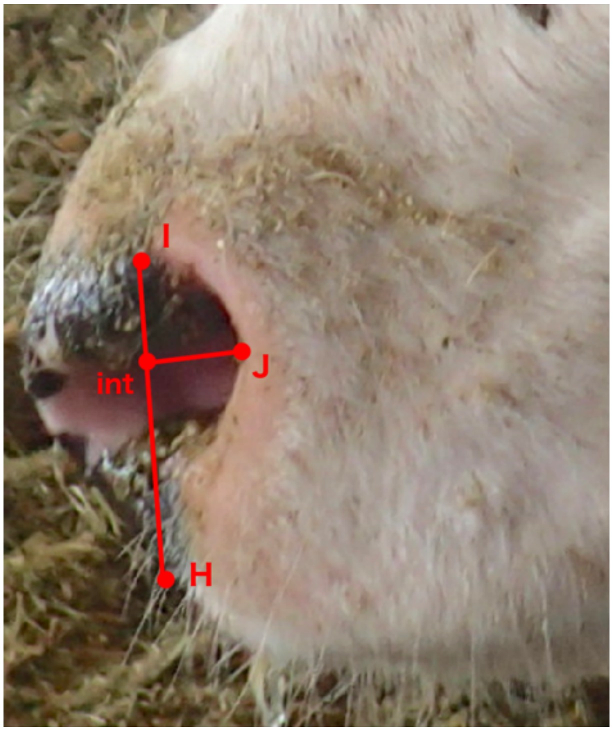

Nostril Height Proportion_Linear

$$NHP = \frac{\|k, k\_int\|}{\overline{HI}}$$

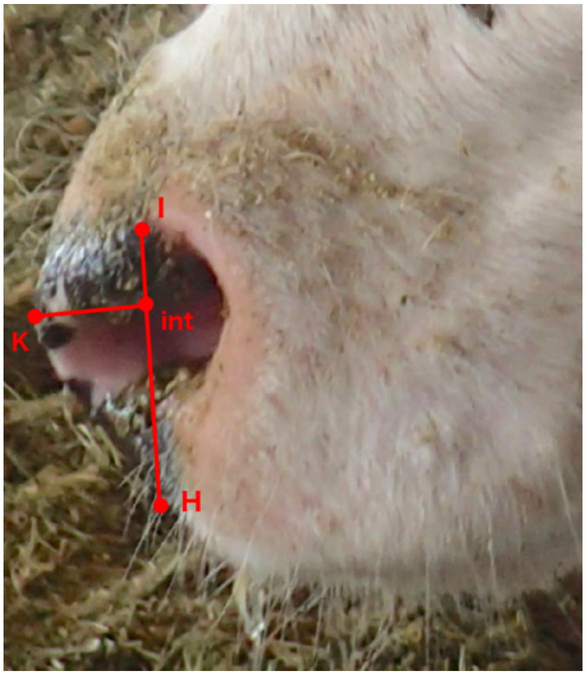

Nostril Position Angle

$$NPA = \angle IHW$$

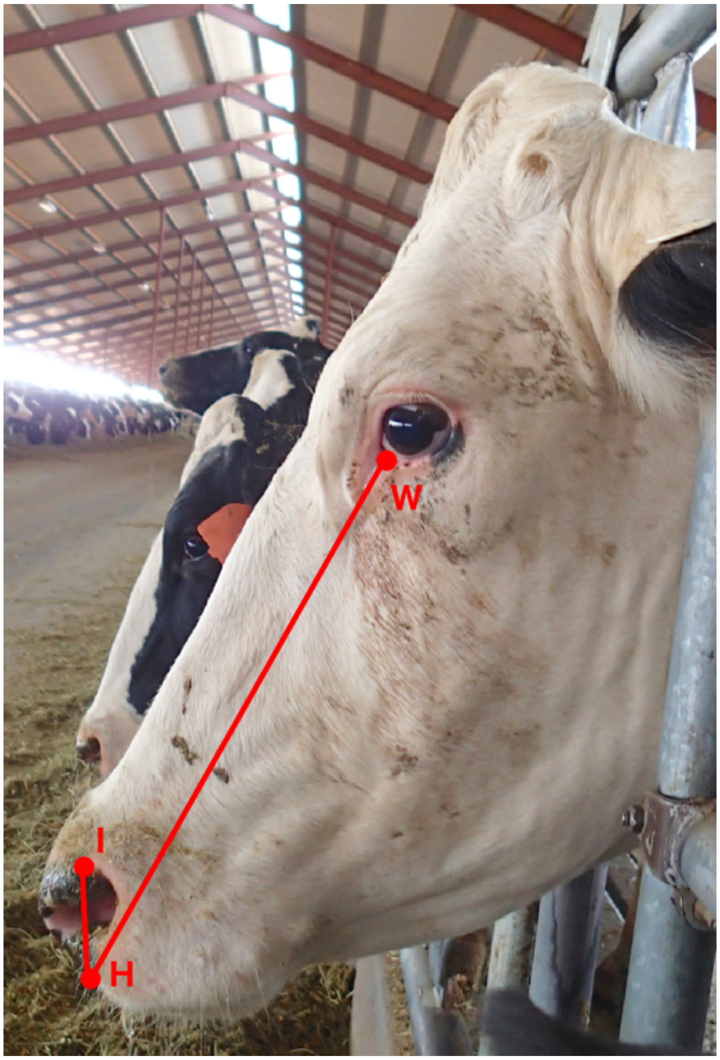

Nostril-Muzzle Ratio_Height_V1

$$NMRH = \frac{\|I_{aux}, H\_aux\|}{\|E_{upper}, A\_aux\|}$$

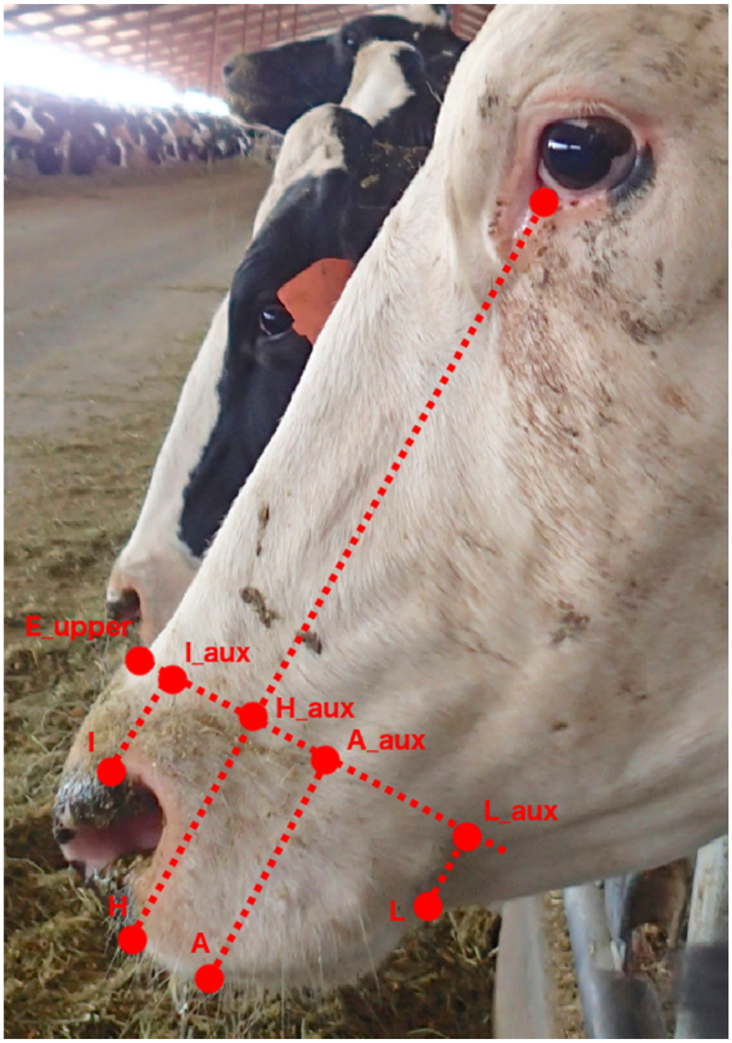

Nostril-Muzzle Ratio_Height_V2

$$NMRH = \frac{\|I_{aux}, H\_aux\|}{\|E_{upper}, L\_aux\|}$$

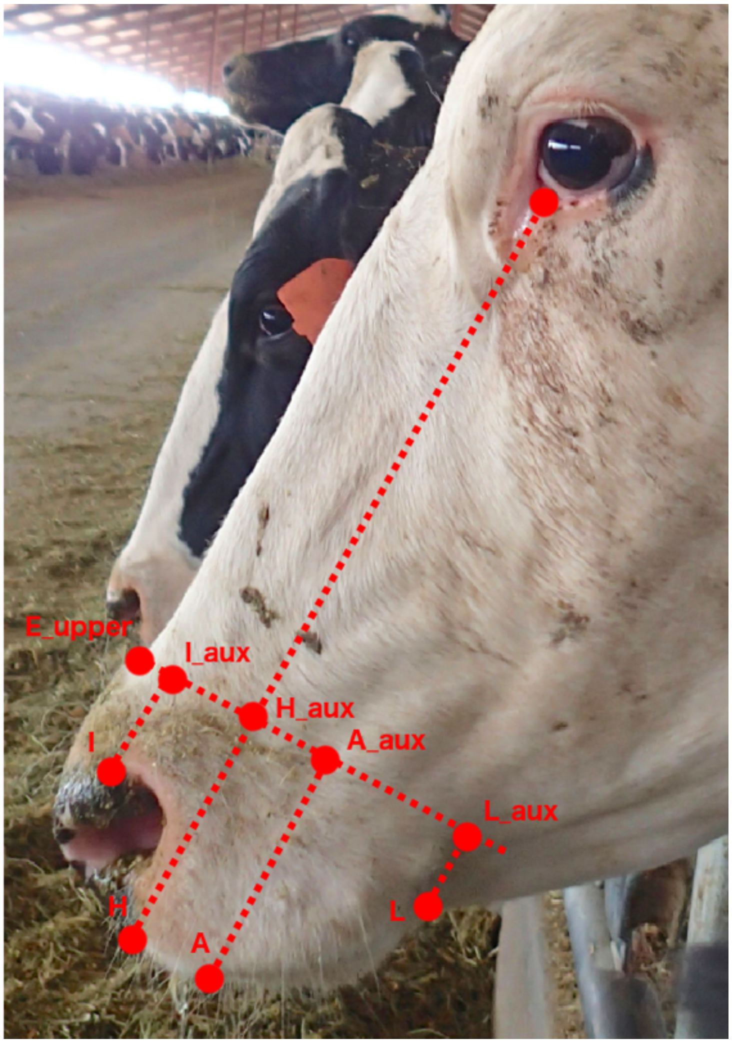

Nostril-Muzzle Ratio_Length

$$NMRL = \frac{\|H, I\_aux2\|}{\|H, E\_aux\|}$$

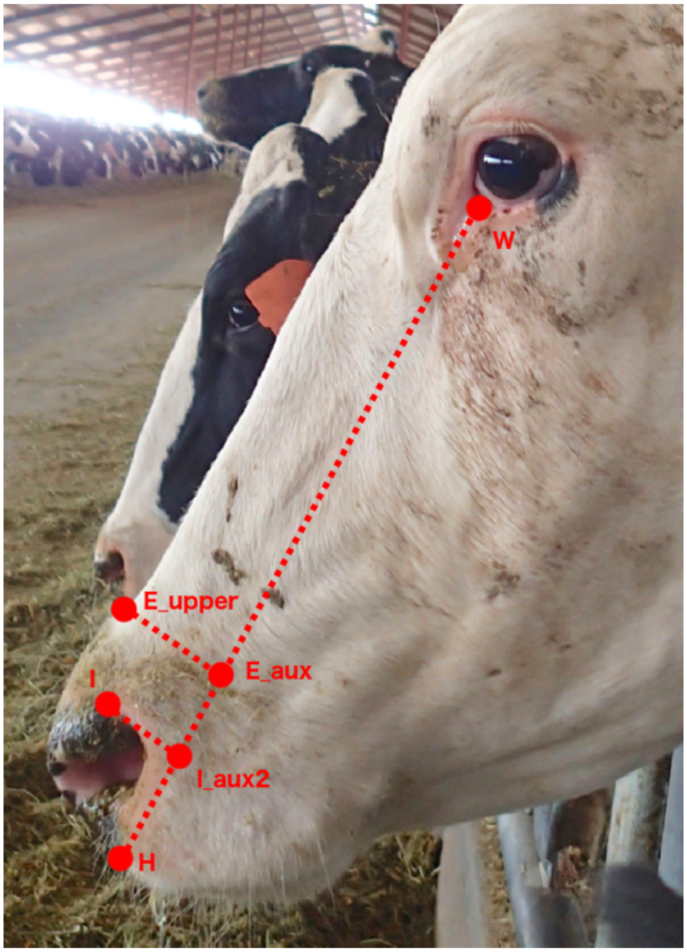

Upper Lip Roundness Point Proportion

$$ULRPP = \frac{\|A, B_{aux}\|}{\|H, A\|}$$

$V1 = \{A_{extrap}\} \rightarrow \{A\}$
$V2 = \{A_{eye}\} \rightarrow \{A\}$

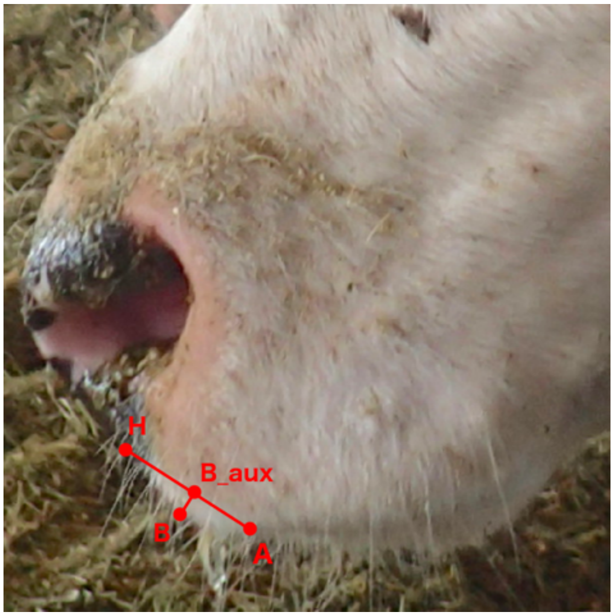

Upper Lip Roundness Proportion

$$ULRP = \frac{\|B, B_{aux}\|}{\|H, A\|}$$

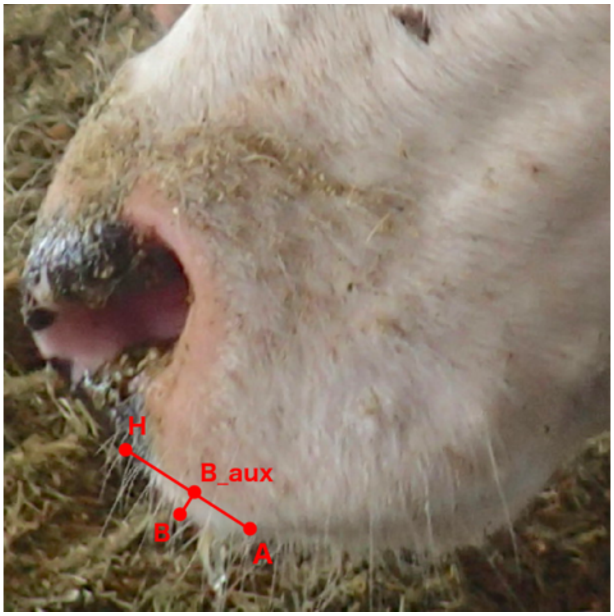

$V1 = \{A_{extrap}\} \rightarrow \{A\}$
$V2 = \{A_{eye}\} \rightarrow \{A\}$

Midface Divergence Proportion

$$MDP = CF * \frac{\|F_{int2}, F\|}{\|C, F\|}$$

$$CF = \frac{\|F, F_{aux2}\| - \|F_{int2}, F_{aux2}\|}{|\|F, F_{aux2}\| - \|F_{int2}, F_{aux2}\||}$$

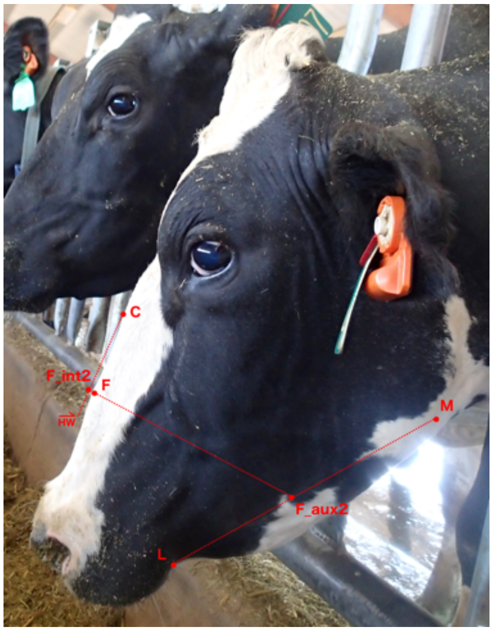

$$V1 = \{C_{extrap}, F_{extrap}\} \rightarrow \{C, F\}$$
$$V2 = \{C_{eye}, F_{extrap}\} \rightarrow \{C, F\}$$
$$V3 = \{C_{extrap}, F_{eye}\} \rightarrow \{C, F\}$$
$$V4 = \{C_{eye}, F_{eye}\} \rightarrow \{C, F\}$$

Midface Inflection Point Proportion

$$MIPP = \frac{\|C, GU_{int}\|}{\|F, C\|}$$

$$V1 = \left\{C_{extrap}, F_{extrap}\right\} \rightarrow \{C, F\}$$
$$V2 = \left\{C_{eye}, F_{extrap}\right\} \rightarrow \{C, F\}$$
$$V3 = \left\{C_{extrap}, F_{eye}\right\} \rightarrow \{C, F\}$$
$$V4 = \left\{C_{eye}, F_{eye}\right\} \rightarrow \{C, F\}$$

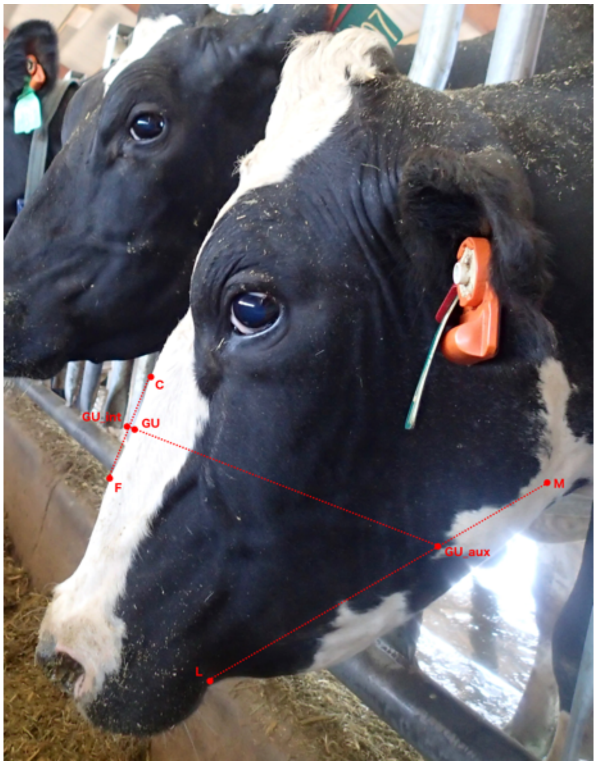

Midface Inflection Proportion

$$MIP = CF * \frac{\|GU, GU_{int}\|}{\|F, C\|}$$

$$CF = \frac{\|GU, GU_{aux}\| - \|GU_{int}, GU_{aux}\|}{|\|GU, GU_{aux}\| - \|GU_{int}, GU_{aux}\||}$$

$$V1 = \{C_{extrap}, F_{extrap}\} \rightarrow \{C, F\}$$
$$V2 = \{C_{eye}, F_{extrap}\} \rightarrow \{C, F\}$$
$$V3 = \{C_{extrap}, F_{eye}\} \rightarrow \{C, F\}$$
$$V4 = \{C_{eye}, F_{eye}\} \rightarrow \{C, F\}$$

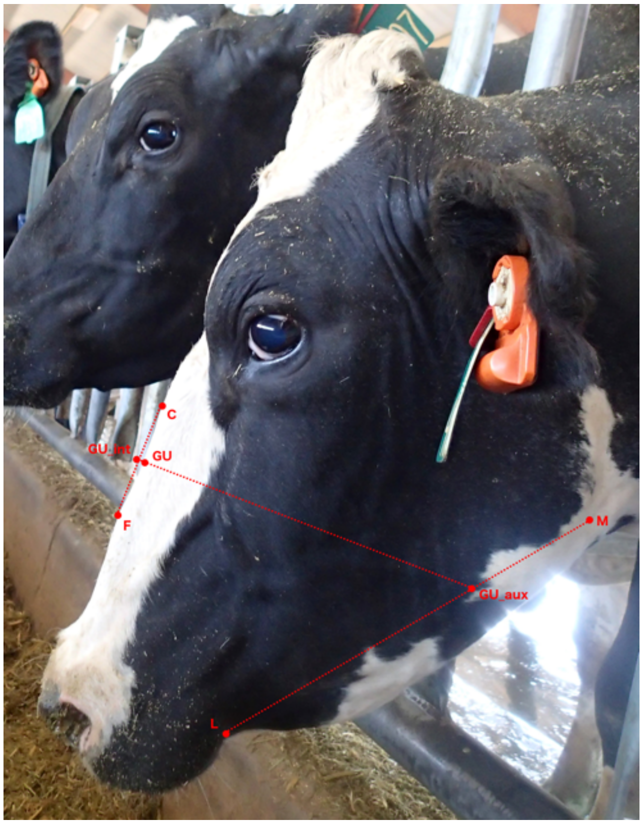

Midface-Nose Length Proportion

$$MNLP = \frac{\|C_{len}, F_{len}\|}{\|F_{len}, E_{len}\|}$$

$$V1 = \{C_{extrap}, F_{extrap}\} \rightarrow \{C, F\}$$
$$V2 = \{C_{eye}, F_{extrap}\} \rightarrow \{C, F\}$$
$$V3 = \{C_{extrap}, F_{eye}\} \rightarrow \{C, F\}$$
$$V4 = \{C_{eye}, F_{eye}\} \rightarrow \{C, F\}$$

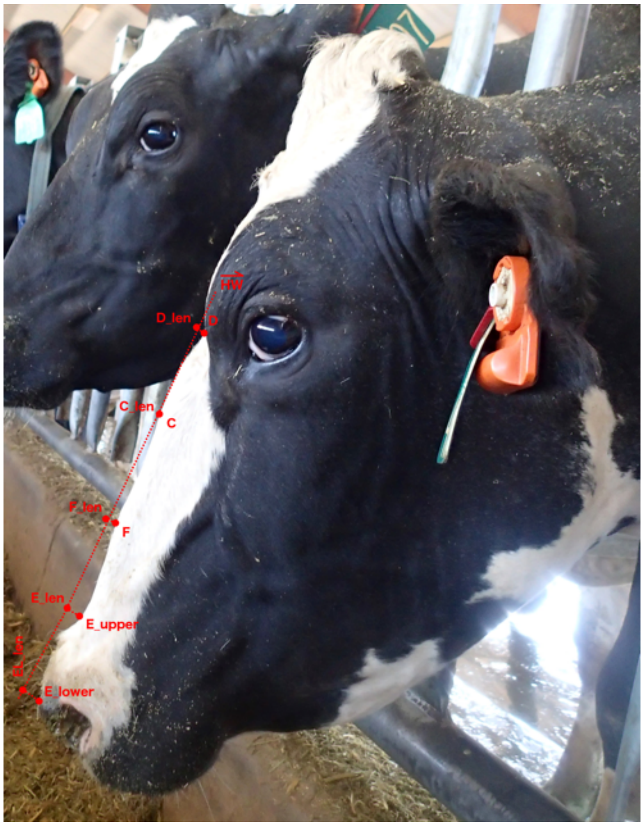

Midface-Nose Roundness Proportion

$$MNRP = CF * \frac{\|E_{int2}, E_{upper}\|}{\|E_{upper}, F\|}$$

$$CF = \frac{\|E_{int2}, E_{aux2}\| - \|E_{upper}, E_{aux2}\|}{\left|\|E_{int2}, E_{aux2}\| - \|E_{upper}, E_{aux2}\|\right|}$$

$$V1 = \{C_{extrap}, F_{extrap}\} \rightarrow \{C, F\}$$
$$V2 = \{C_{eye}, F_{extrap}\} \rightarrow \{C, F\}$$
$$V3 = \{C_{extrap}, F_{eye}\} \rightarrow \{C, F\}$$
$$V4 = \{C_{eye}, F_{eye}\} \rightarrow \{C, F\}$$

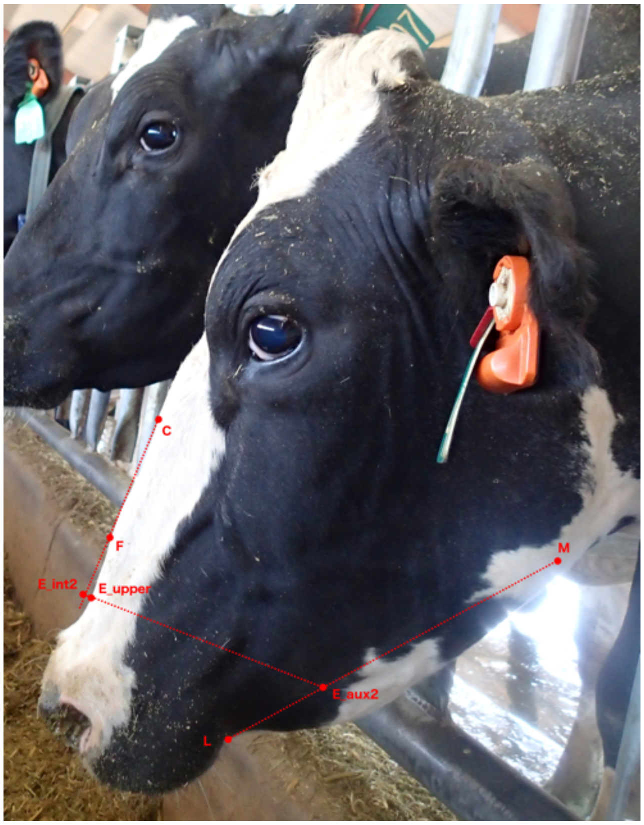

Midface-Topline Length Proportion

$$MTLP = \frac{\|C_{len}, F\_len\|}{\|EL_{len}, D_{len}\|}$$

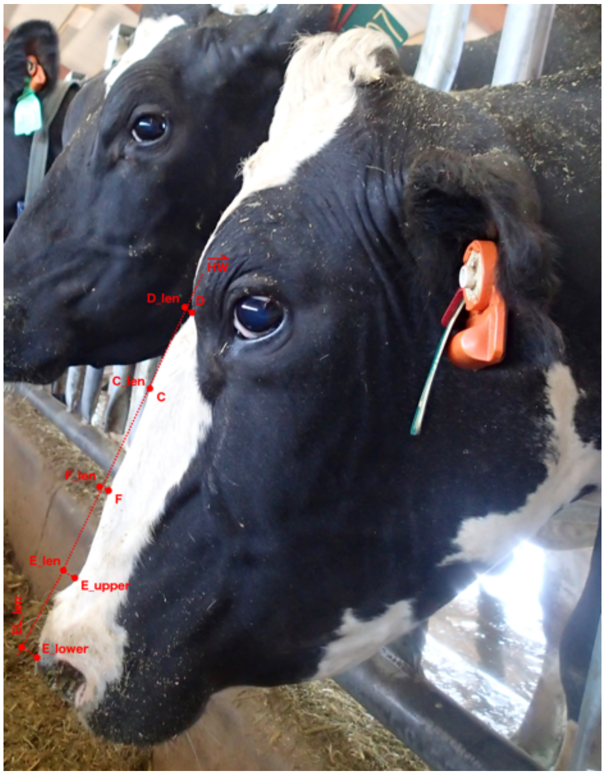

$$V1 = \left\{C_{extrap}, F_{extrap}\right\} \rightarrow \{C, F\}$$
$$V2 = \left\{C_{eye}, F_{extrap}\right\} \rightarrow \{C, F\}$$
$$V3 = \left\{C_{extrap}, F_{eye}\right\} \rightarrow \{C, F\}$$
$$V4 = \left\{C_{eye}, F_{eye}\right\} \rightarrow \{C, F\}$$

Nares Divergence Proportion_V1

$$NaDP = CF * \frac{\|E_{lower}, E_{int4}\|}{\left\|E_{upper}, E_{lower}\right\|}$$

$$CF = \frac{\|E_{lower}, E_{aux4}\| - \|E_{int4}, E_{aux4}\|}{|\|E_{lower}, E_{aux4}\| - \|E_{int4}, E_{aux4}\||}$$

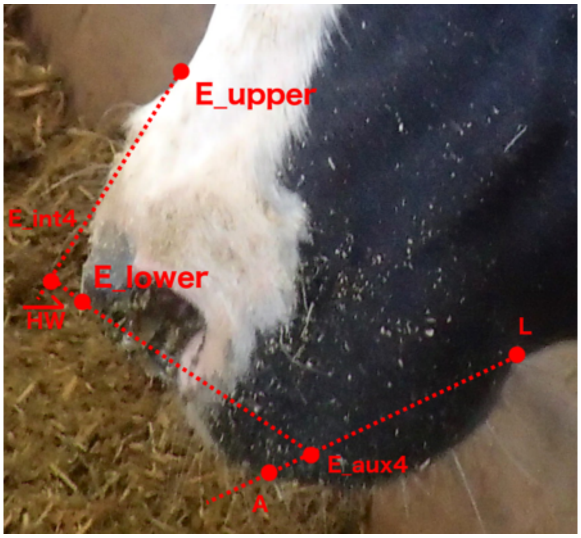

Nares Divergence Proportion_V2

$$NaDP = CF * \frac{\|E_{lower}, E_{int4}\|}{\|E_{mid}, E_{int4}\|}$$

$$CF = \frac{\|E_{lower}, E_{aux4}\| - \|E_{int4}, E_{aux4}\|}{|\|E_{lower}, E_{aux4}\| - \|E_{int4}, E_{aux4}\||}$$

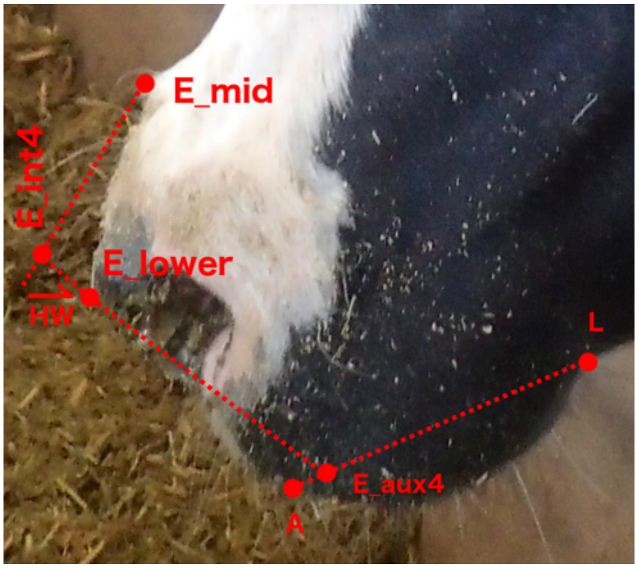

Nares Divergence Proportion_V3

$$NaDP = CF * \frac{\|E_{lower}, E_{int4}\|}{\|E_{upper}, E_{int4}\|}$$

$$CF = \frac{\|E_{lower}, E_{aux4}\| - \|E_{int4}, E_{aux4}\|}{|\|E_{lower}, E_{aux4}\| - \|E_{int4}, E_{aux4}\||}$$

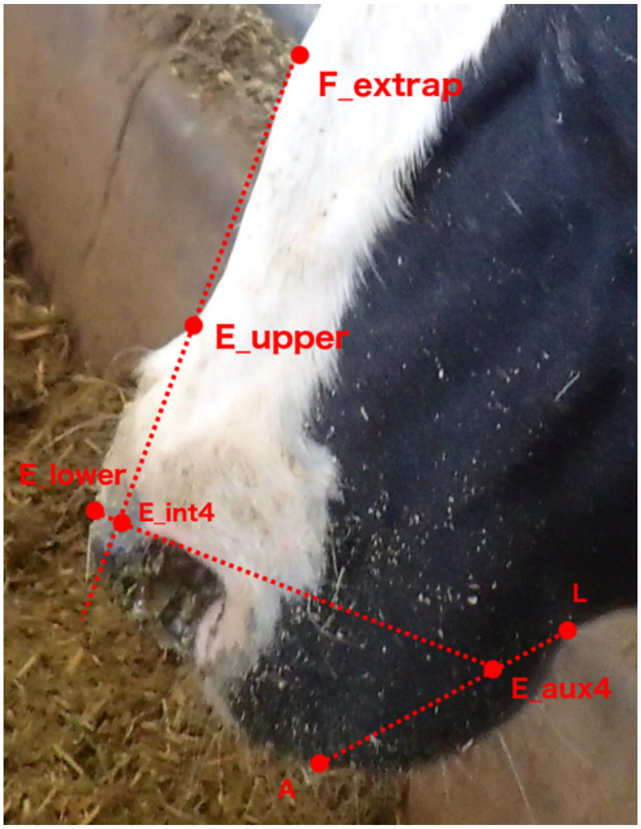

Nares Divergence Proportion_V4

$$NaDP = CF * \frac{\|E_{lower}, E_{int4}\|}{\|E_{upper}, E_{int4}\|}$$

$$CF = \frac{\|E_{lower}, E_{aux4}\| - \|E_{int4}, E_{aux4}\|}{|\|E_{lower}, E_{aux4}\| - \|E_{int4}, E_{aux4}\||}$$

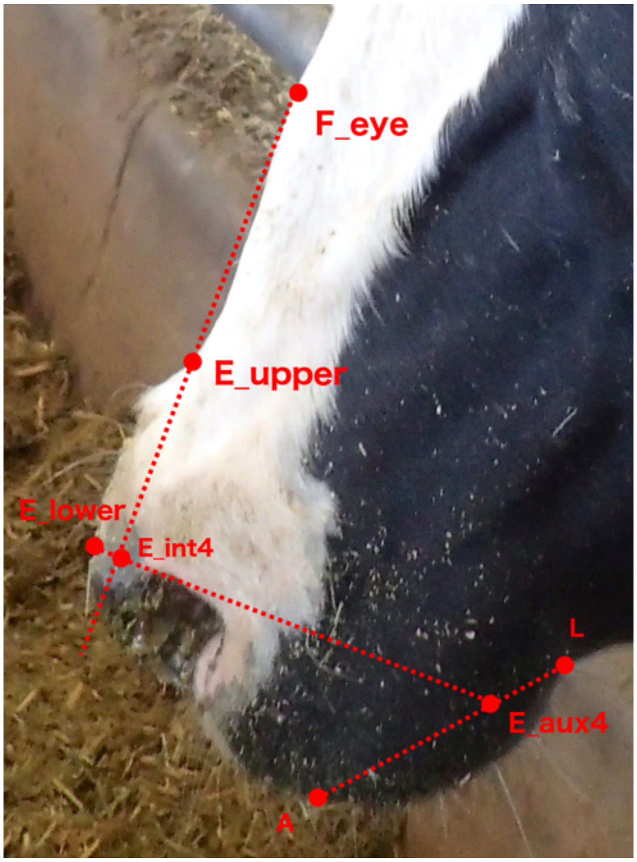

Nares Roundness Point Proportion

$$NRPP = \frac{\left\| E_{upper}, E_{int} \right\|}{\left\| E_{upper}, E_{lower} \right\|}$$

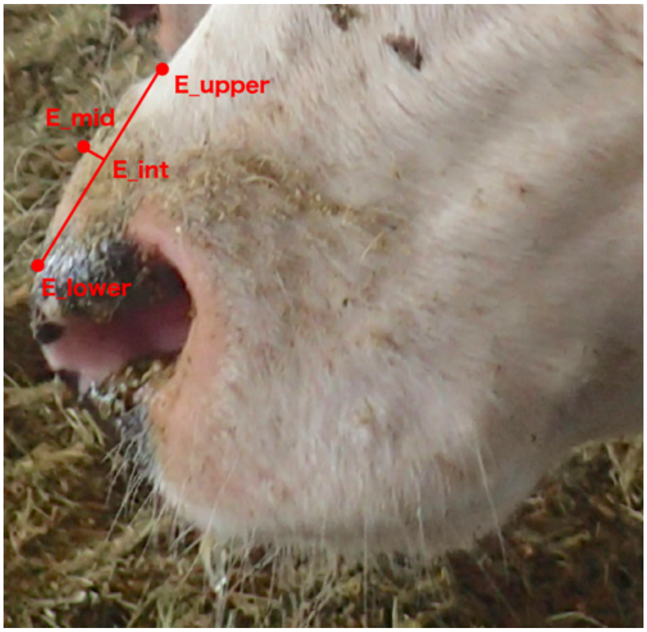

Nares Roundness Proportion

$$NRP = \frac{\|E_{mid}, E_{int}\|}{\|E_{upper}, E_{lower}\|}$$

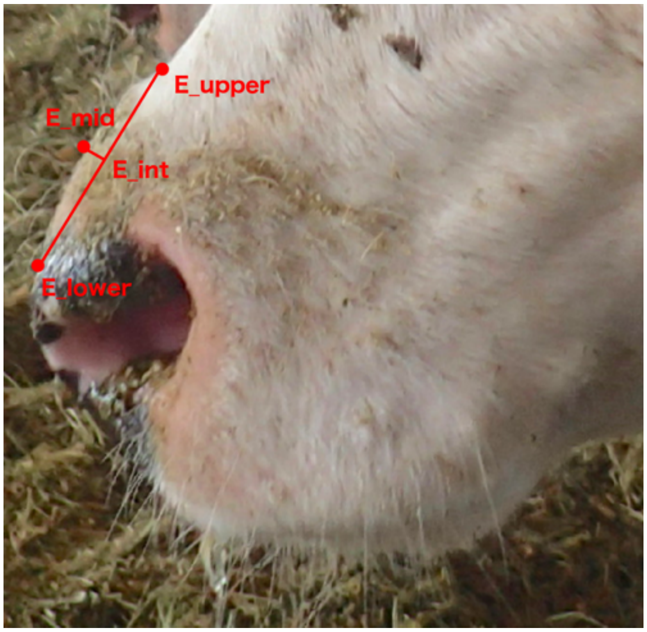

Nares Thickness Proportion_V1

$$NTP = \frac{\|I, IE_{int}\|}{\|E_{lower}, E_{upper}\|}$$

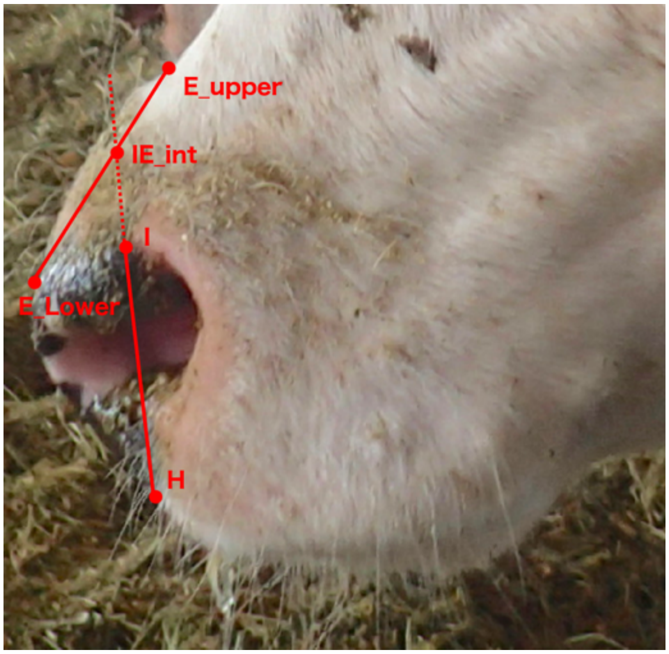

Nares Thickness Proportion_V1

$$NTP = \frac{\|I, IE_{int}\|}{\|I, H\|}$$

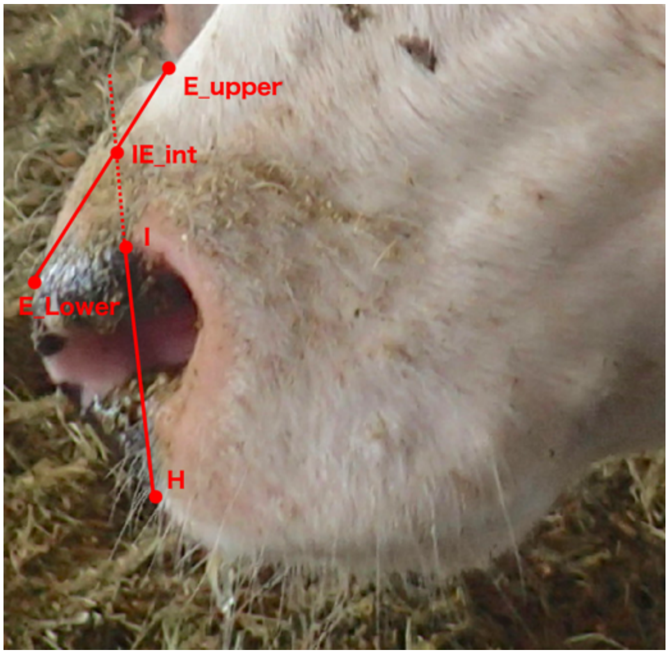

Nares Thickness Proportion_V3

$$NTP = \frac{\|I_{aux}, IE_{aux}\|}{\|IE_{aux}, H_{aux}\|}$$

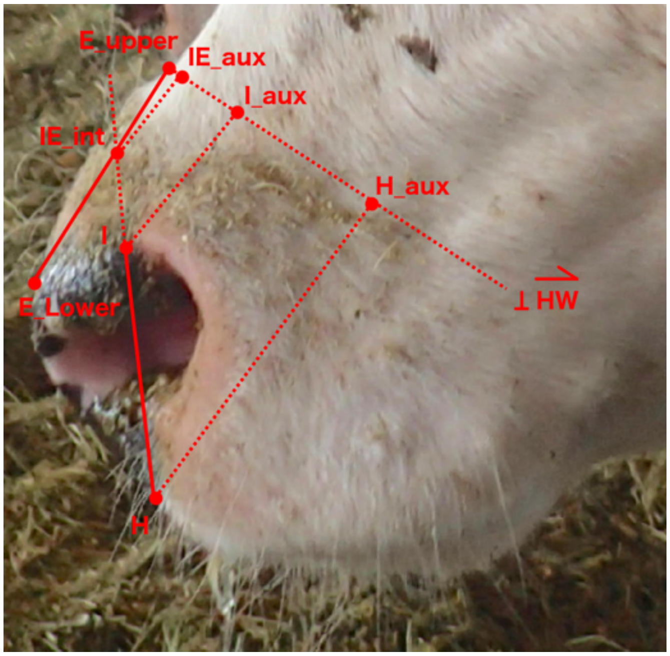

Nares-Nose Length Proportion

$$NNLP = \frac{\|EL_{len}, E_{len}\|}{\|E_{len}, F_{len}\|}$$

$$V1 = \{F_{extrap}\} \rightarrow \{F\}$$
$$V2 = \{F_{eye}\} \rightarrow \{F\}$$

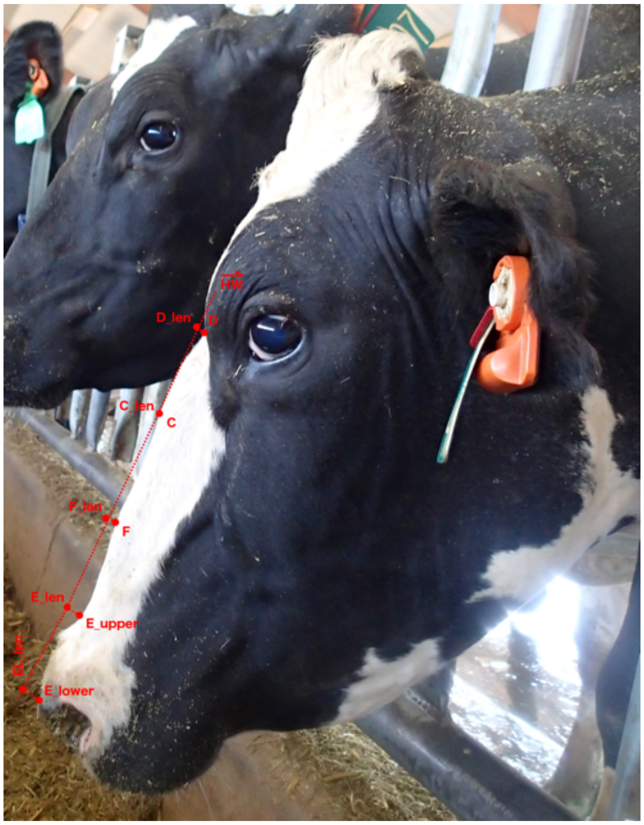

Nares-Topline Length Proportion

$$NaTLP = \frac{\|F_{eye}, GL_{int}\|}{\|F_{eye}, E_{upper}\|}$$

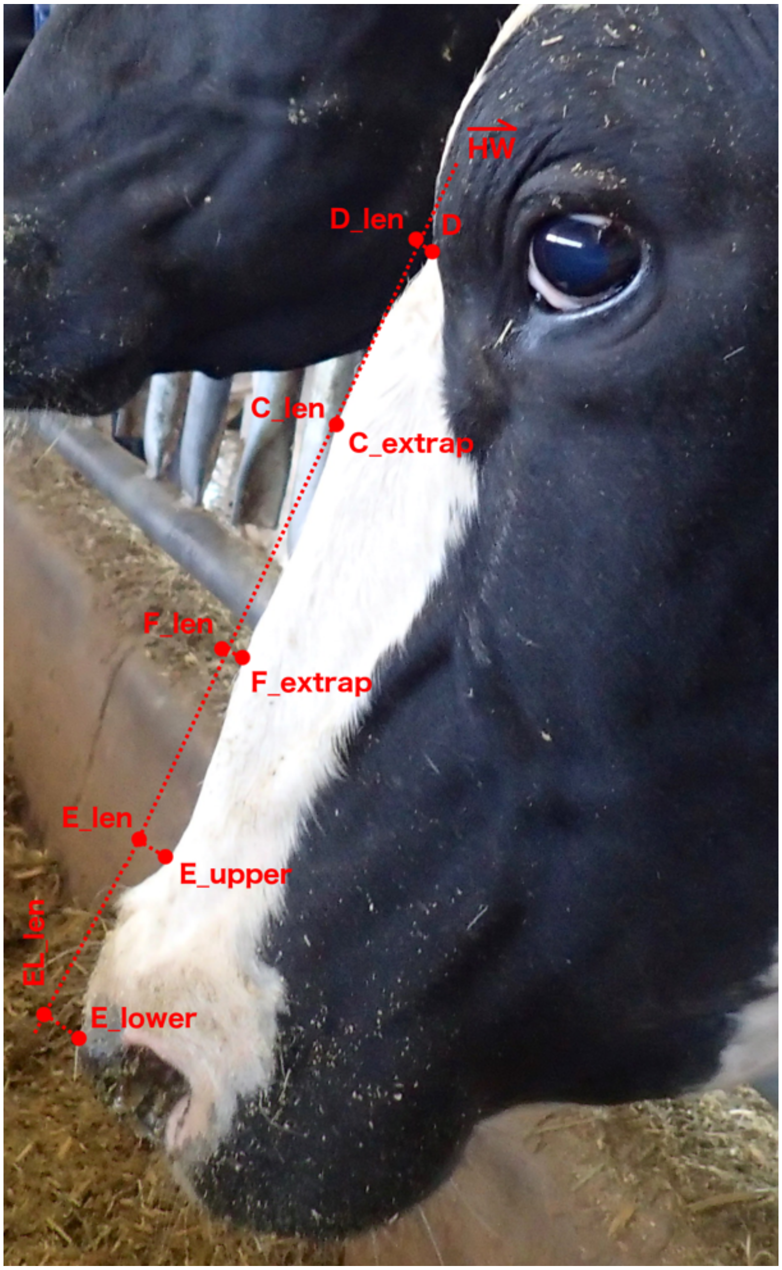

Nose Divergence Proportion

$$NDP = CF * \frac{\left\|E_{upper}, E_{int3}\right\|}{\left\|E_{upper}, F_{extrap}\right\|}$$

$$CF = \frac{\left\|E_{upper}, E_{aux3}\right\| - \left\|E_{int3}, E_{aux3}\right\|}{\left|\left\|E_{upper}, E_{aux3}\right\| - \left\|E_{int3}, E_{aux3}\right\|\right|}$$

$$V1 = \left\{F_{extrap}\right\} \rightarrow \{F\}$$
$$V2 = \left\{F_{eye}\right\} \rightarrow \{F\}$$

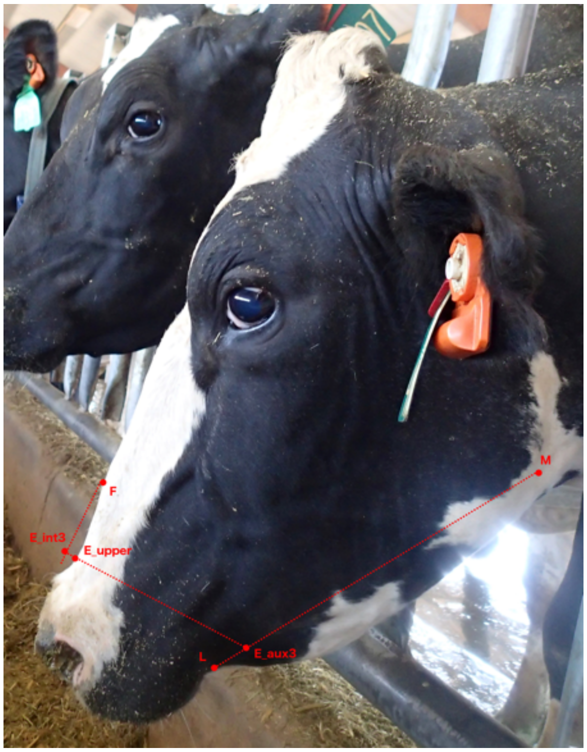

Nose Inflection Point Proportion

$$NIPP = \frac{\|F, GL_{int}\|}{\|F, E_{upper}\|}$$

$$V1 = \{F_{extrap}\} \rightarrow \{F\}$$
$$V2 = \{F_{eye}\} \rightarrow \{F\}$$

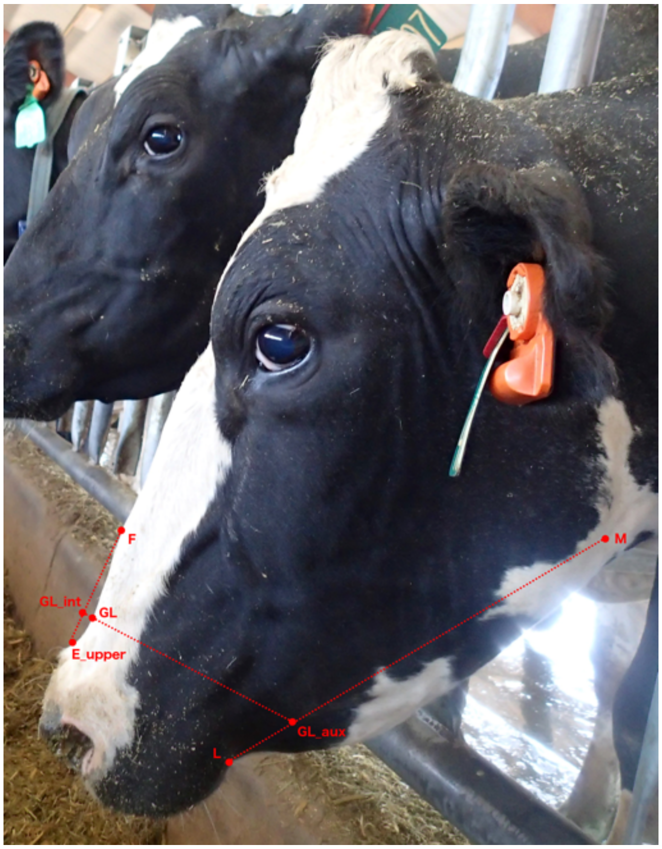

Nose Inflection Proportion

$$NIP = CF * \frac{\|GL, GL_{int}\|}{\|F, E_{upper}\|}$$

$$CF = \frac{\|GL, GL_{aux}\| - \|GL_{int}, GL_{aux}\|}{|\|GL, GL_{aux}\| - \|GL_{int}, GL_{aux}\||}$$

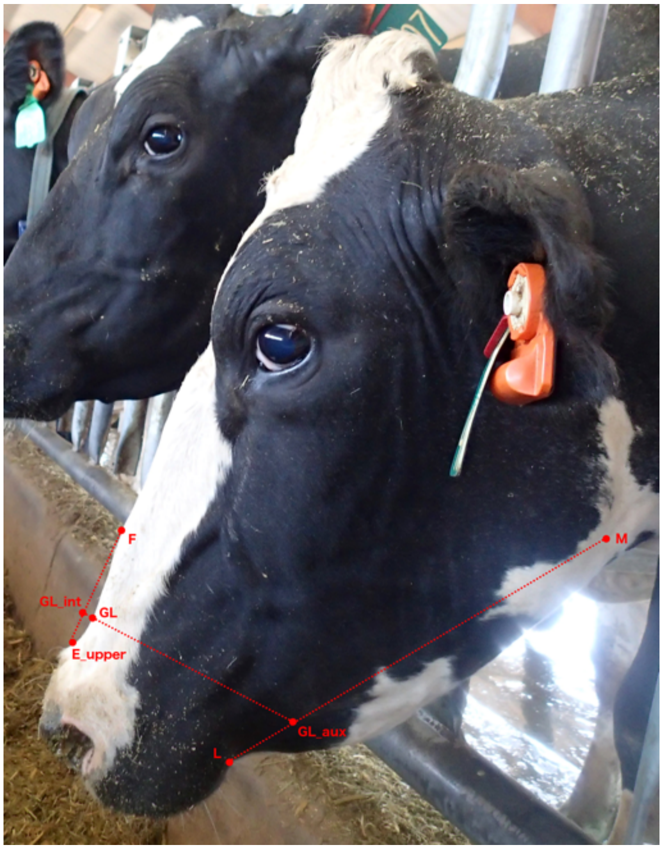

$$V1 = \{F_{extrap}\} \rightarrow \{F\}$$
$$V2 = \{F_{eye}\} \rightarrow \{F\}$$

Nose-Topline Length Proportion

$$NTLP = \frac{\|E_{len}, F\_len\|}{\|EL_{len}, D_{len}\|}$$

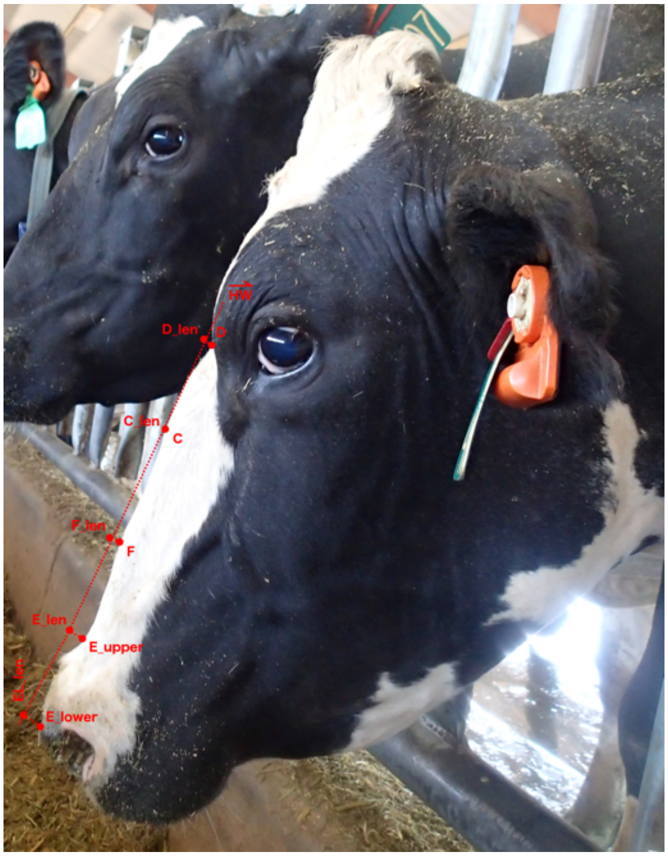

$$V1 = \{F_{extrap}\} \rightarrow \{F\}$$
$$V2 = \{F_{eye}\} \rightarrow \{F\}$$

Sinus Projection Proportion

$$SPP = \frac{\|C_{extrap}, W\|}{\|W, X\|}$$

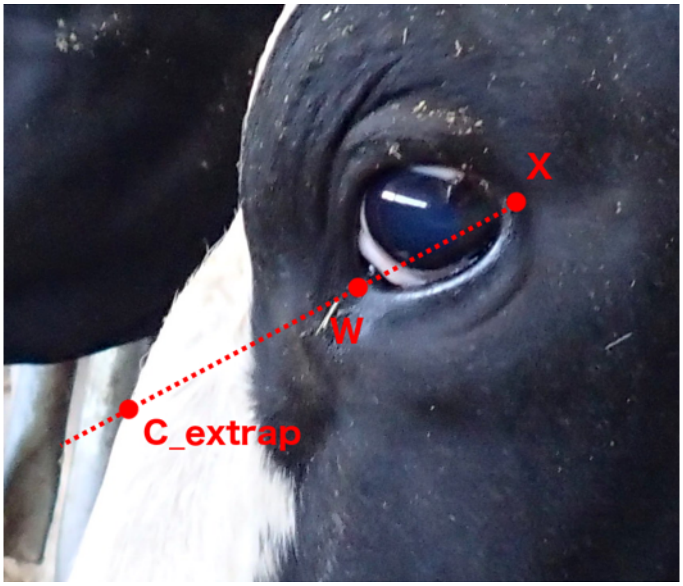

<u>**Sinus-Midface Length Proportion**</u>

$$SMLP = \frac{\|C_{len}, F_{len}\|}{\|C_{len}, D_{len}\|}$$

$$V1 = \left\{C_{extrap}, F_{extrap}\right\} \to \{C, F\}$$
$$V2 = \left\{C_{eye}, F_{extrap}\right\} \to \{C, F\}$$
$$V3 = \left\{C_{extrap}, F_{eye}\right\} \to \{C, F\}$$
$$V4 = \left\{C_{eye}, F_{eye}\right\} \to \{C, F\}$$

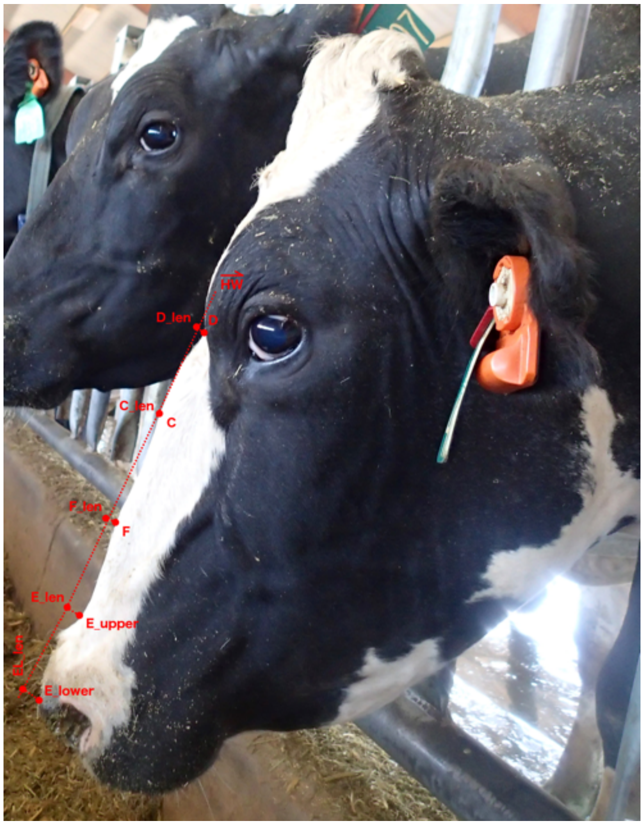

Sinus-Midface Rounding Proportion

$$SMRP = CF * \frac{\|D_{int}, D\|}{\|C_{extrap}, D_{int}\|}$$

$$CF = \frac{\|D_{int}, D_{aux}\| - \|D, D_{aux}\|}{|\|D_{int}, D_{aux}\| - \|D, D_{aux}\||}$$

$$V1 = \{C_{extrap}, F_{extrap}\} \rightarrow \{C, F\}$$
$$V2 = \{C_{eye}, F_{extrap}\} \rightarrow \{C, F\}$$
$$V3 = \{C_{extrap}, F_{eye}\} \rightarrow \{C, F\}$$
$$V4 = \{C_{eye}, F_{eye}\} \rightarrow \{C, F\}$$

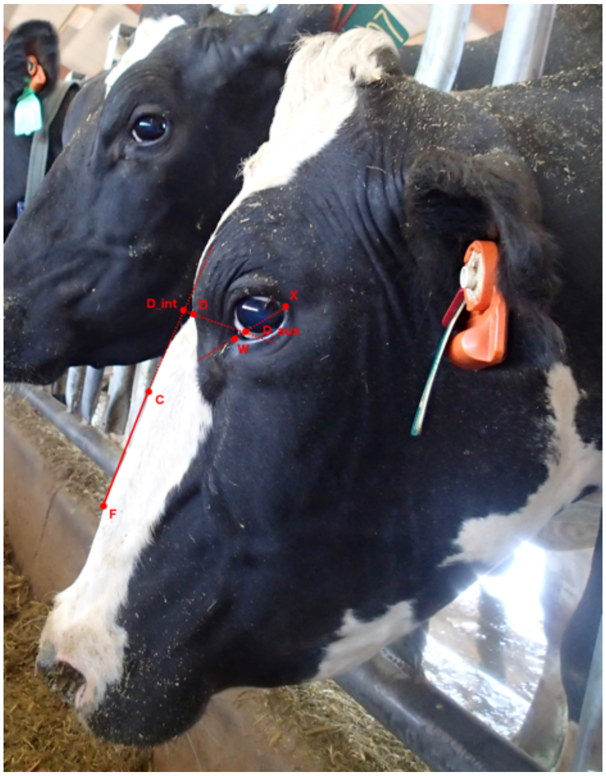

<u>Sinus-Topline Length Proportion</u>

$$STLP = \frac{\|C_{len}, D\_len\|}{\|EL_{len}, D_{len}\|}$$

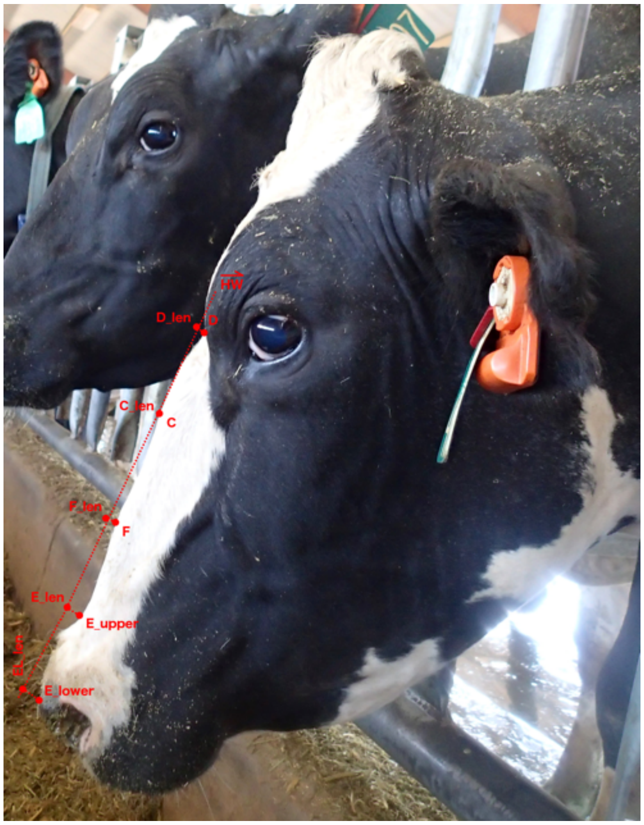

$$V1 = \{C_{extrap}, F_{extrap}\} \rightarrow \{C, F\}$$
$$V2 = \{C_{eye}, F_{eye}\} \rightarrow \{C, F\}$$

Upper-Lower Topline Length Proportion

$$ULTLP = \frac{\|EL_{len}, F_{len}\|}{\|F_{len}, D_{len}\|}$$

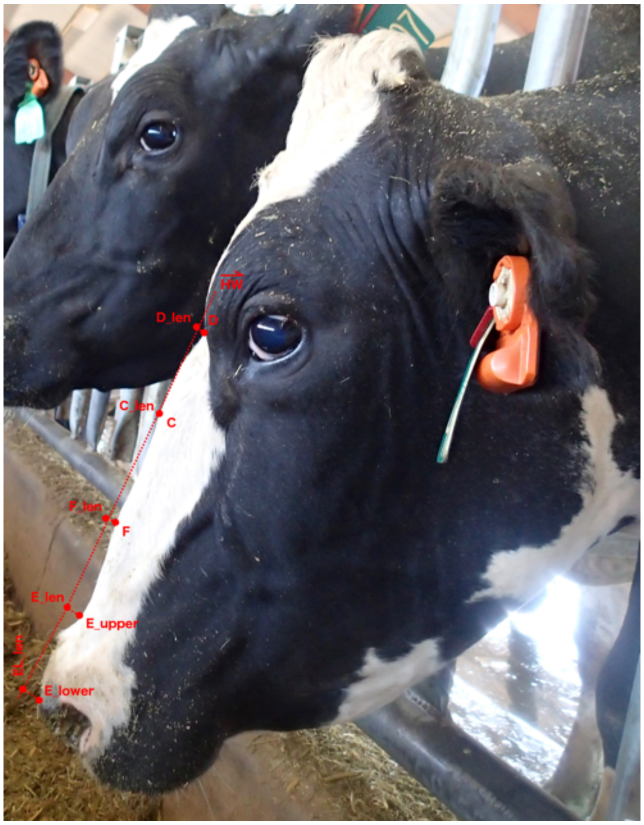

$V1 = \{F_{extrap}\} \rightarrow \{F\}$
$V2 = \{F_{eye}\} \rightarrow \{F\}$
