# Supplementary material for: Improving the Reliability of Scale-Free Image Morphometrics in Applications with Minimally Restrained Livestock Using Projective Geometry and Unsupervised Machine Learning
Source: Sensors (Basel). 2022 Oct 31;22(21):8347. doi: 10.3390/s22218347 (PMC9653925; doi:10.3390/s22218347)
Supplement: Supplementary file 1 [file sensors-22-08347-s001.zip › SupplementalMaterials/Visualizations/AtribPlotAll.pdf]

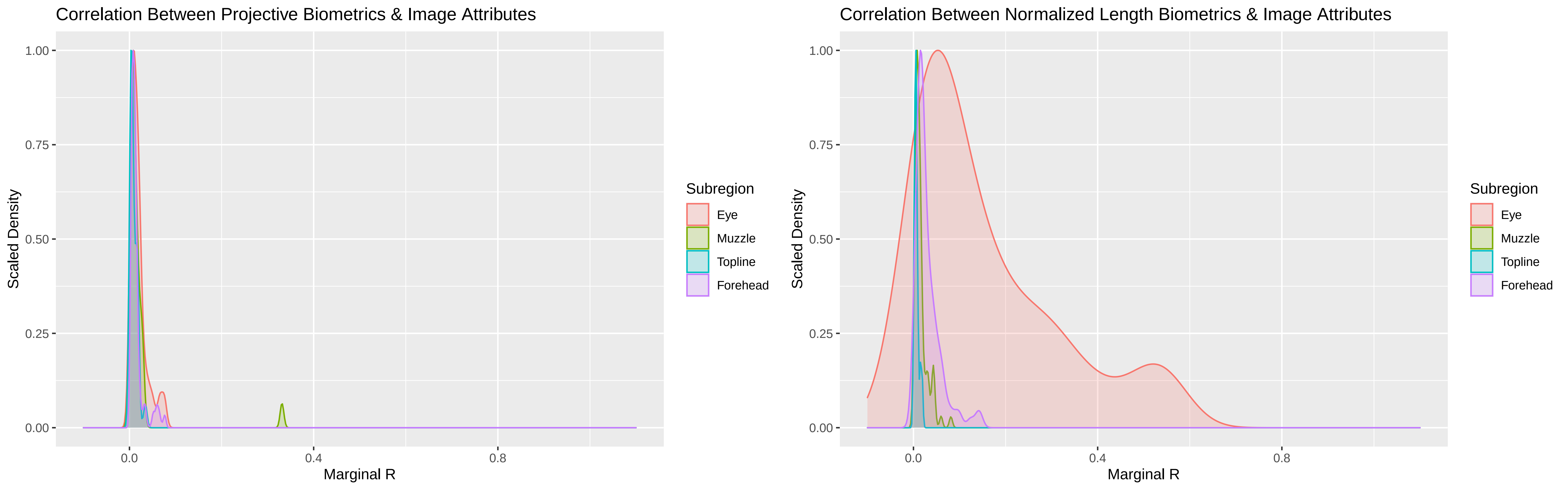

Correlation Between Projective Biometrics & Image Attributes
Scaled Density
1.00
0.75
0.50
0.25
0.00
0.0
0.4
0.8
Marginal R
Subregion
Eye
Muzzle
Topline
Forehead
Correlation Between Normalized Length Biometrics & Image Attributes
Scaled Density
1.00
0.75
0.50
0.25
0.00
0.0
0.4
0.8
Marginal R
Subregion
Eye
Muzzle
Topline
Forehead
