# Supplementary material for: Improving the Reliability of Scale-Free Image Morphometrics in Applications with Minimally Restrained Livestock Using Projective Geometry and Unsupervised Machine Learning
Source: Sensors (Basel). 2022 Oct 31;22(21):8347. doi: 10.3390/s22218347 (PMC9653925; doi:10.3390/s22218347)
Supplement: Supplementary file 1 [file sensors-22-08347-s001.zip › SupplementalMaterials/Visualizations/BiasPlot.pdf]

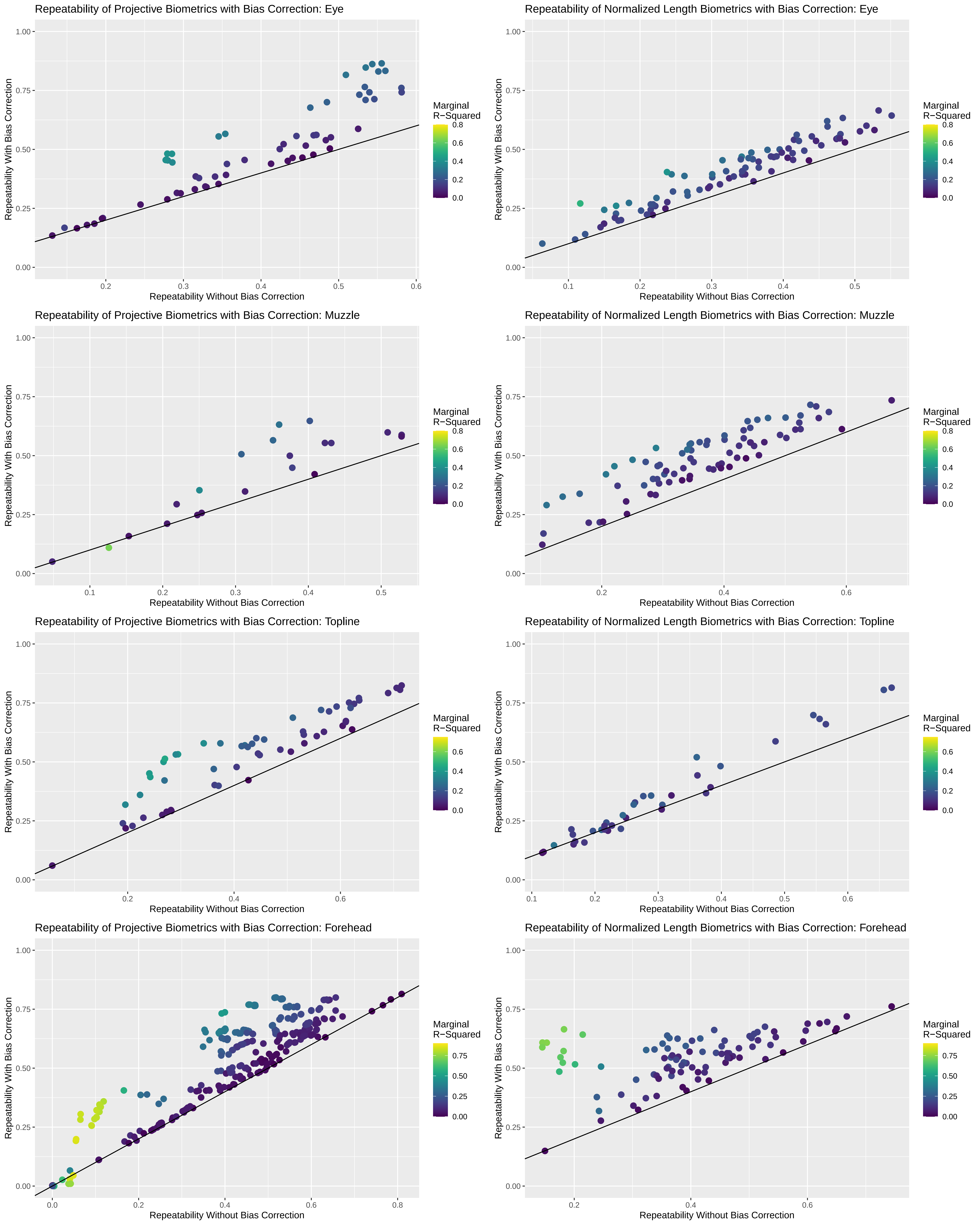

Repeatability of Projective Biometrics with Bias Correction: Eye
Repeatability of Normalized Length Biometrics with Bias Correction: Eye
Repeatability of Projective Biometrics with Bias Correction: Muzzle
Repeatability of Normalized Length Biometrics with Bias Correction: Muzzle
Repeatability of Projective Biometrics with Bias Correction: Topline
Repeatability of Normalized Length Biometrics with Bias Correction: Topline
Repeatability of Projective Biometrics with Bias Correction: Forehead
Repeatability of Normalized Length Biometrics with Bias Correction: Forehead
Repeatability With Bias Correction
Repeatability Without Bias Correction
Marginal R−Squared
