# Supplementary figures and images for: Improving the Reliability of Scale-Free Image Morphometrics in Applications with Minimally Restrained Livestock Using Projective Geometry and Unsupervised Machine Learning (part 2 of 2)
Source: Sensors (Basel). 2022 Oct 31;22(21):8347. doi: 10.3390/s22218347 (PMC9653925; doi:10.3390/s22218347)

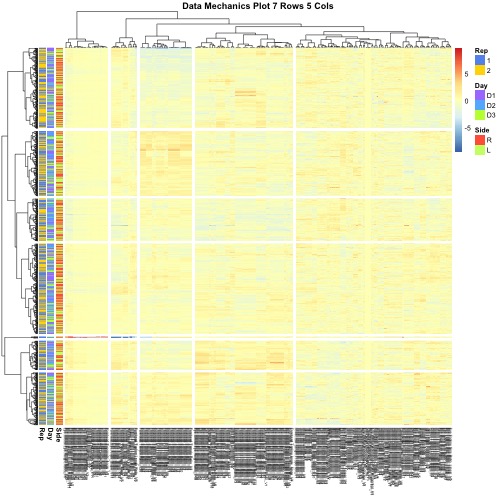

Supplement: Supplementary file 1 [file sensors-22-08347-s001.zip › SupplementalMaterials/Visualizations/DataMechanics/Forehead/Geom/Image/ErrorEncoding_Image_R7C5.jpeg]

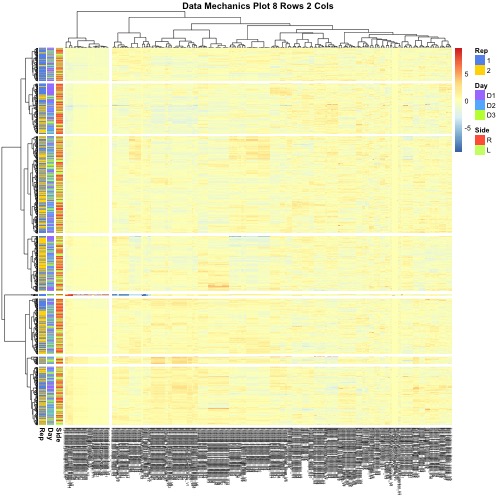

Supplement: Supplementary file 1 [file sensors-22-08347-s001.zip › SupplementalMaterials/Visualizations/DataMechanics/Forehead/Geom/Image/ErrorEncoding_Image_R8C2.jpeg]

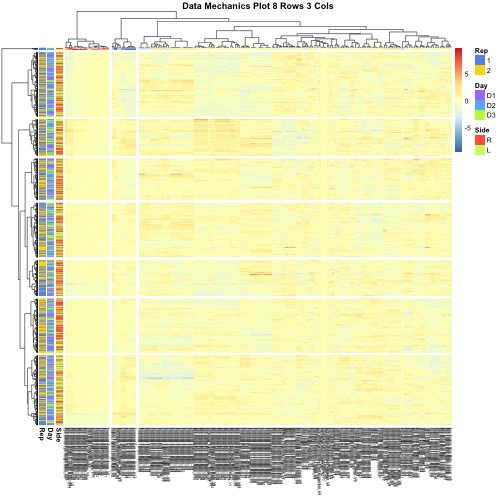

Supplement: Supplementary file 1 [file sensors-22-08347-s001.zip › SupplementalMaterials/Visualizations/DataMechanics/Forehead/Geom/Image/ErrorEncoding_Image_R8C3.jpeg]

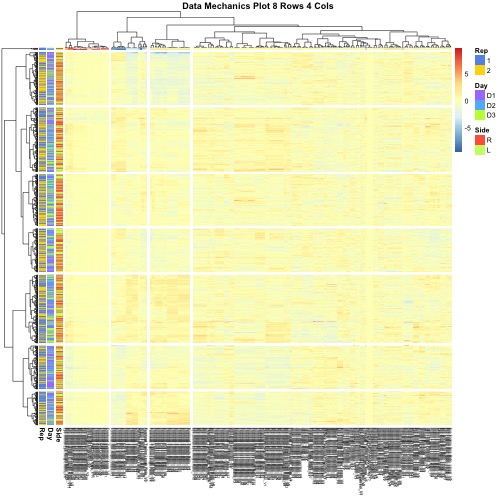

Supplement: Supplementary file 1 [file sensors-22-08347-s001.zip › SupplementalMaterials/Visualizations/DataMechanics/Forehead/Geom/Image/ErrorEncoding_Image_R8C4.jpeg]

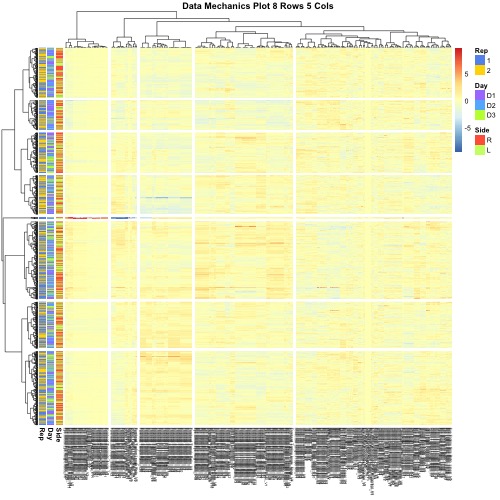

Supplement: Supplementary file 1 [file sensors-22-08347-s001.zip › SupplementalMaterials/Visualizations/DataMechanics/Forehead/Geom/Image/ErrorEncoding_Image_R8C5.jpeg]

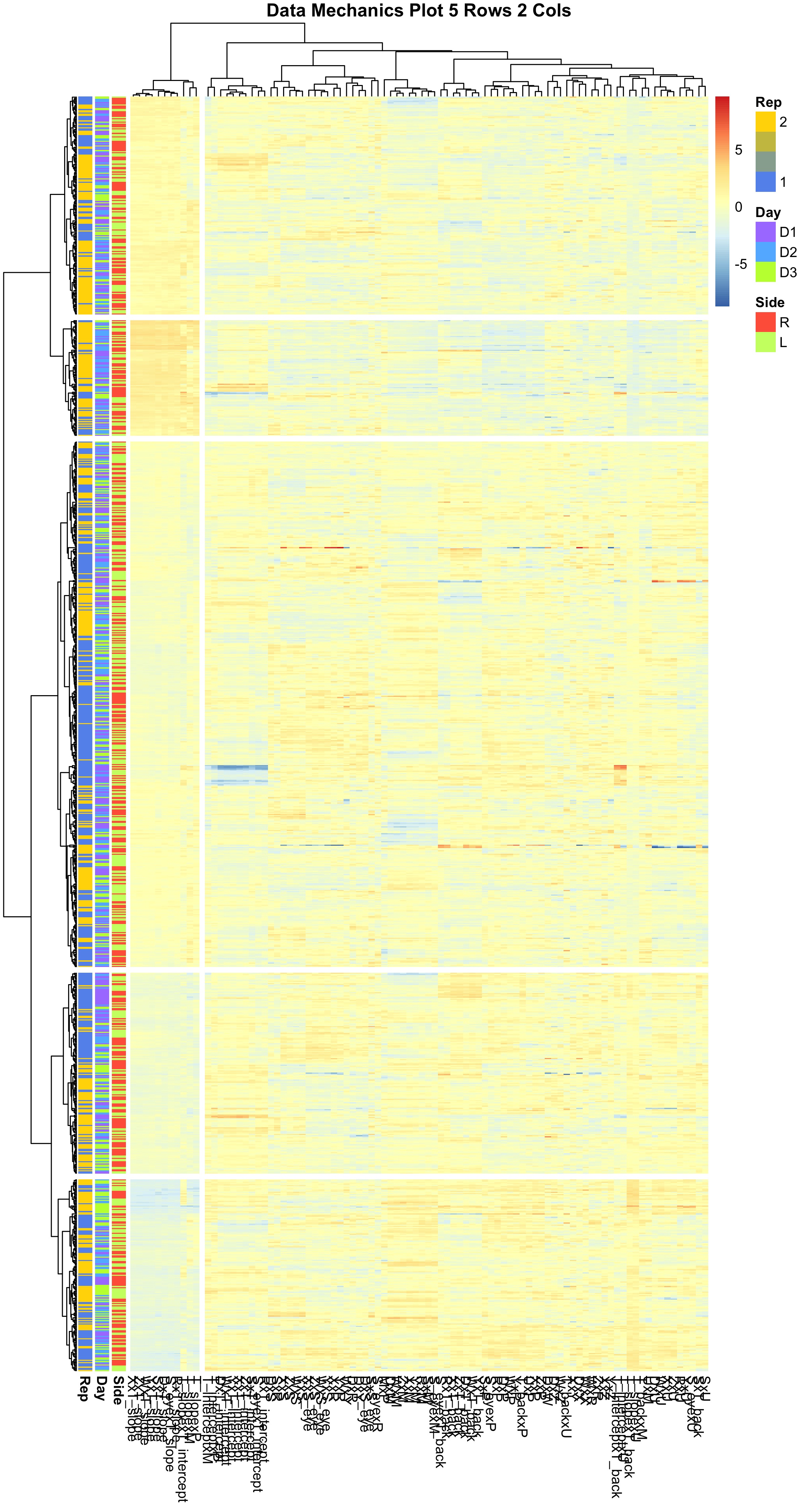

Supplement: Supplementary file 1 [file sensors-22-08347-s001.zip › SupplementalMaterials/Visualizations/DataMechanics/Forehead/NormLength/Annotation/ErrorEncoding_Annotation_R5C2.jpeg]

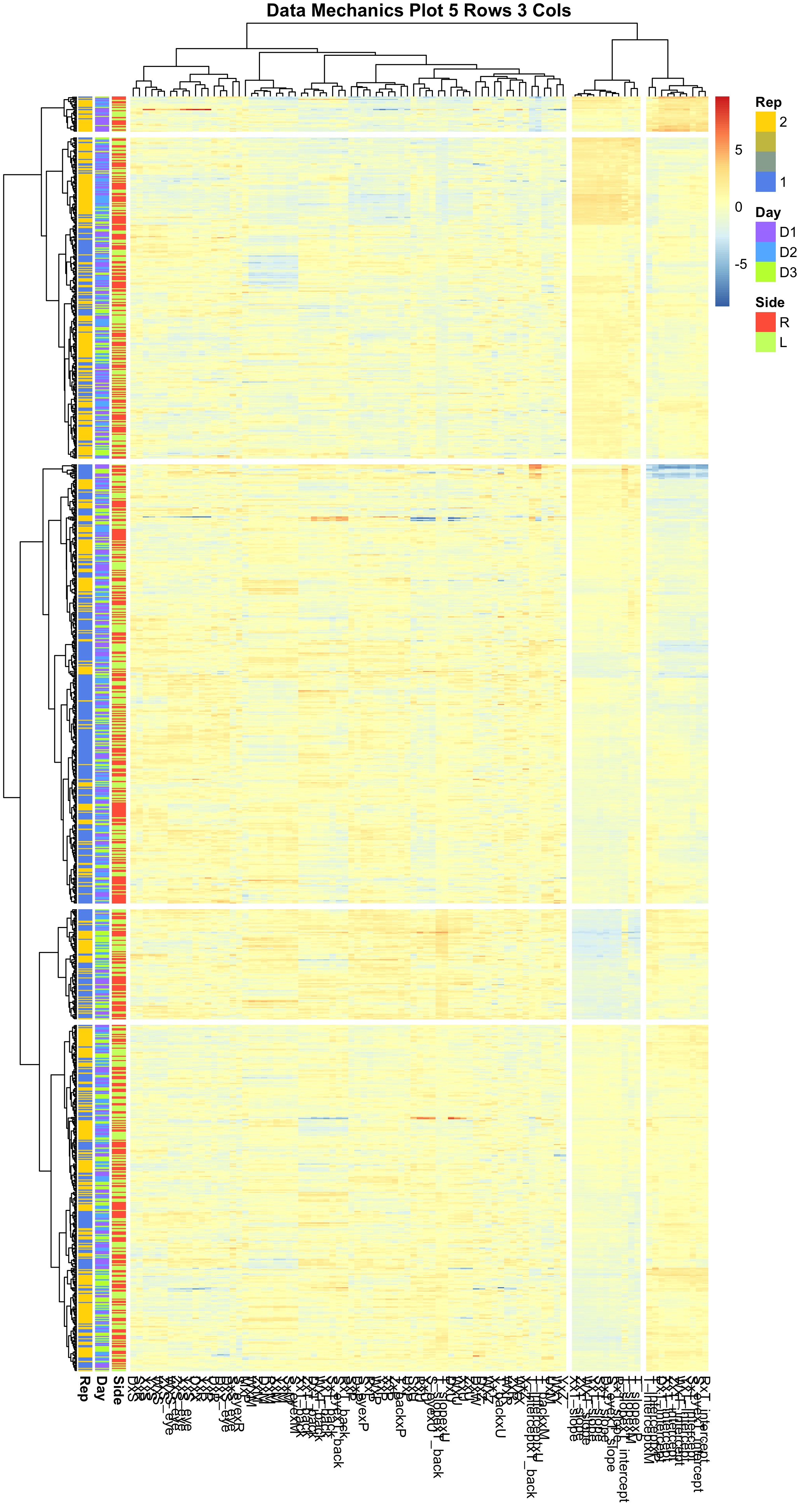

Supplement: Supplementary file 1 [file sensors-22-08347-s001.zip › SupplementalMaterials/Visualizations/DataMechanics/Forehead/NormLength/Annotation/ErrorEncoding_Annotation_R5C3.jpeg]

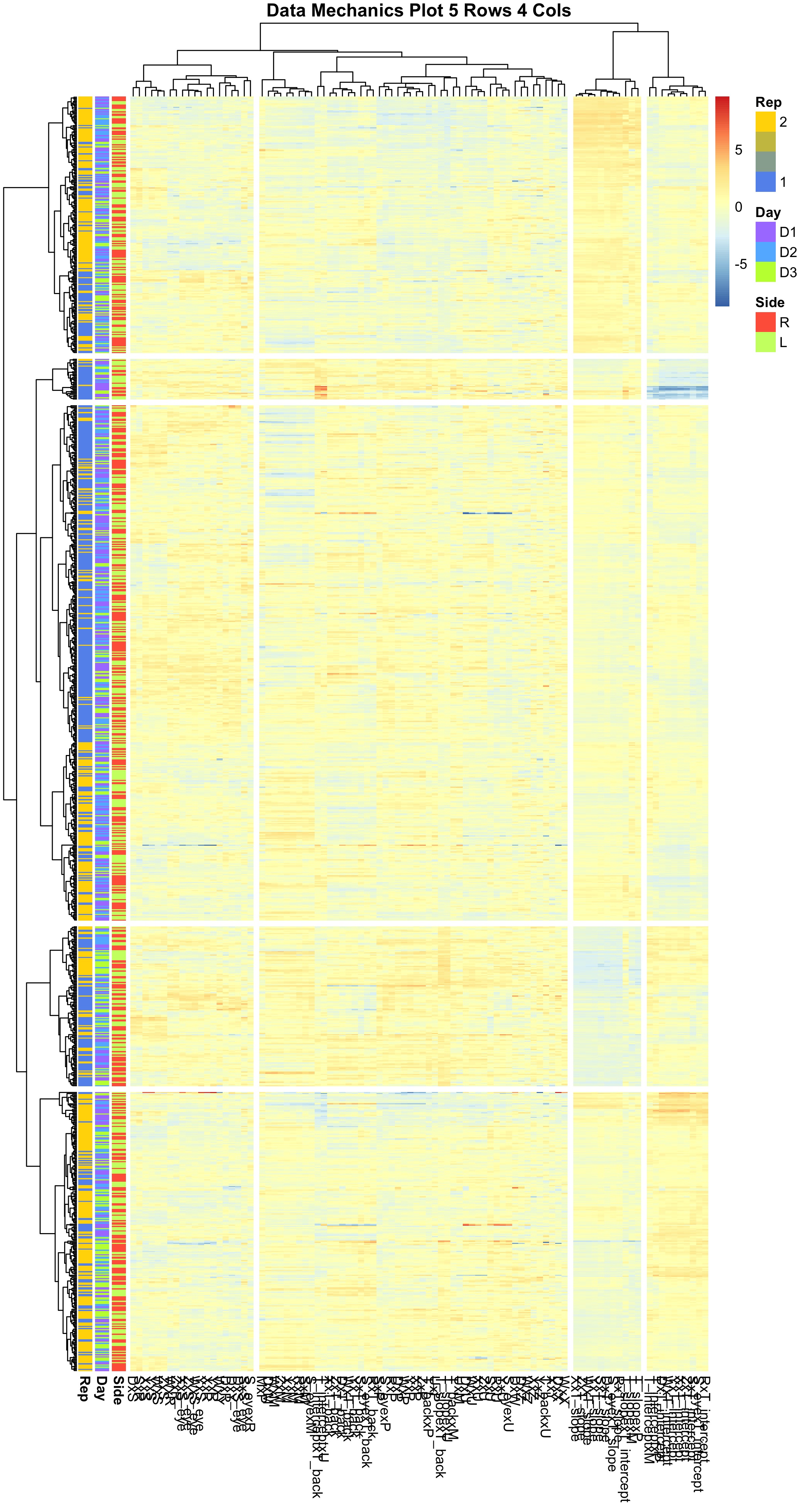

Supplement: Supplementary file 1 [file sensors-22-08347-s001.zip › SupplementalMaterials/Visualizations/DataMechanics/Forehead/NormLength/Annotation/ErrorEncoding_Annotation_R5C4.jpeg]

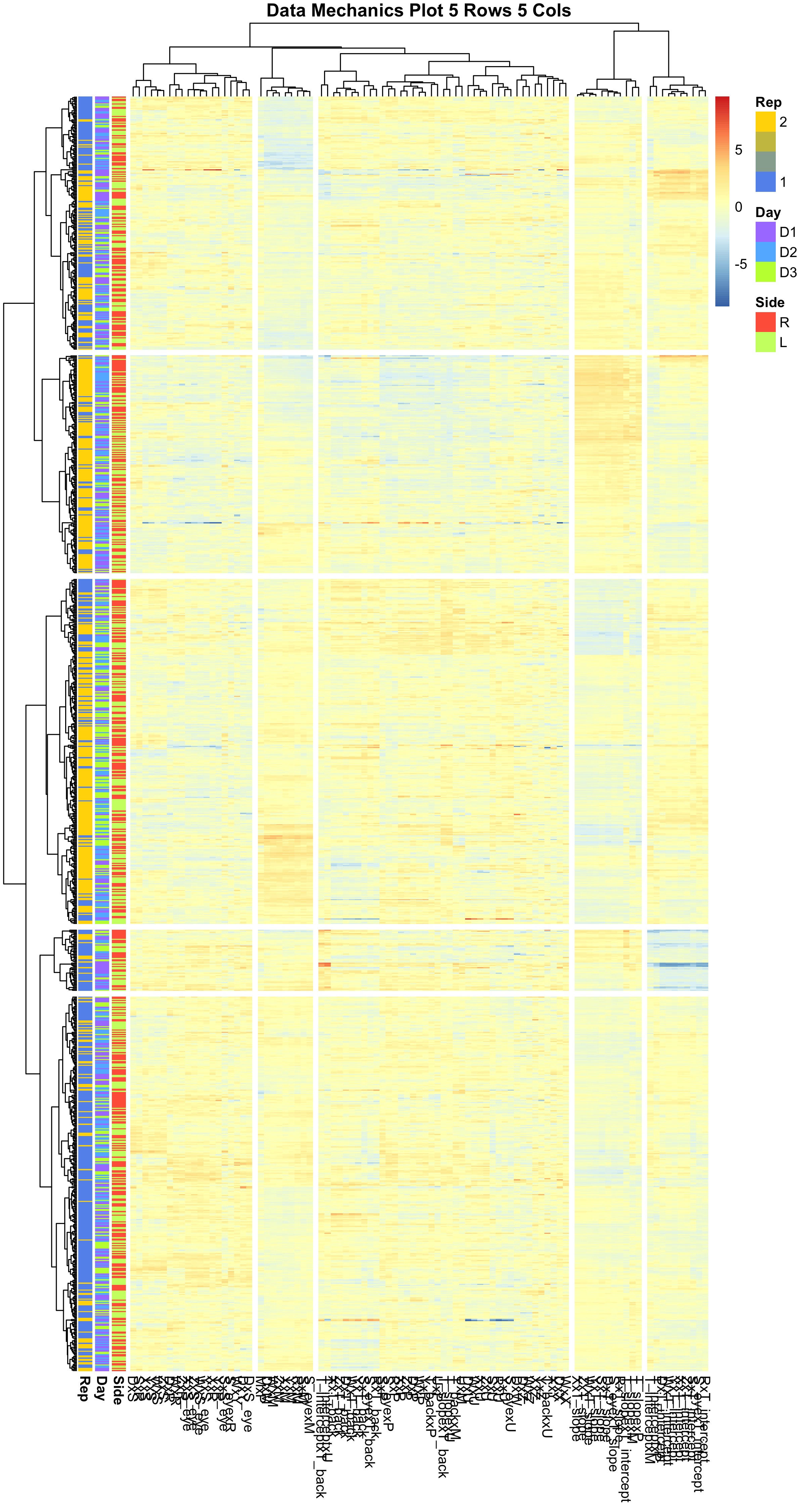

Supplement: Supplementary file 1 [file sensors-22-08347-s001.zip › SupplementalMaterials/Visualizations/DataMechanics/Forehead/NormLength/Annotation/ErrorEncoding_Annotation_R5C5.jpeg]

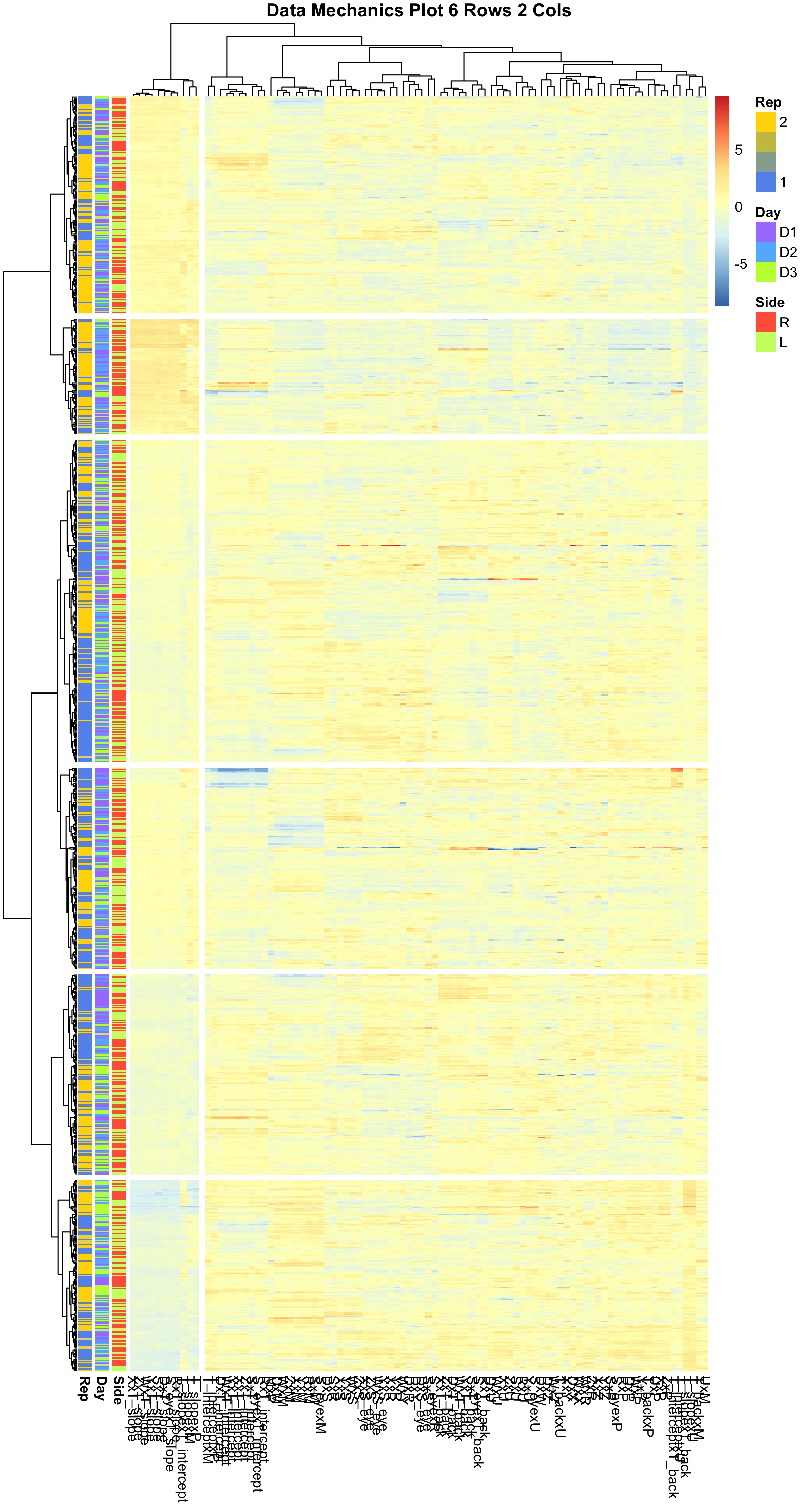

Supplement: Supplementary file 1 [file sensors-22-08347-s001.zip › SupplementalMaterials/Visualizations/DataMechanics/Forehead/NormLength/Annotation/ErrorEncoding_Annotation_R6C2.jpeg]

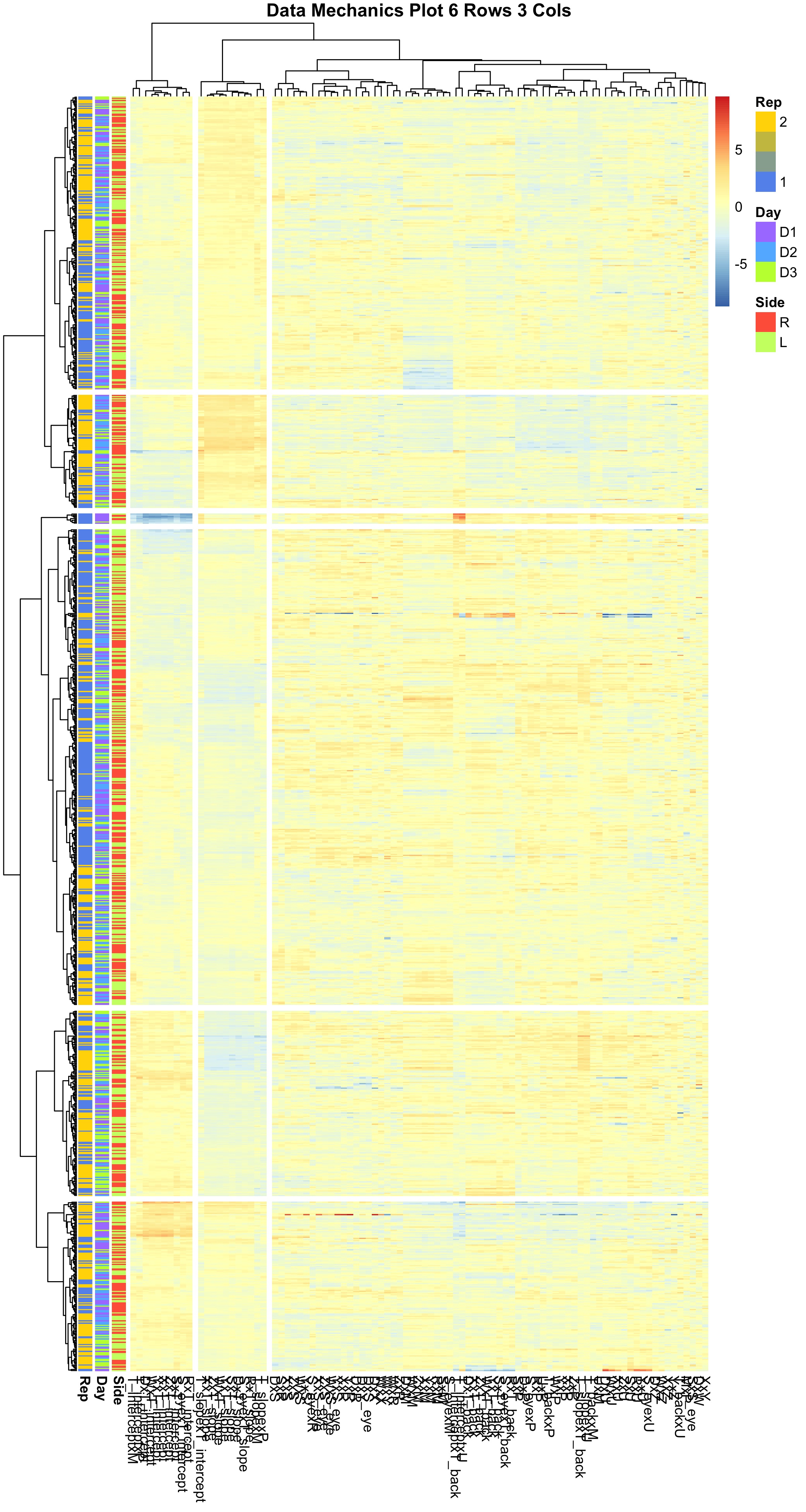

Supplement: Supplementary file 1 [file sensors-22-08347-s001.zip › SupplementalMaterials/Visualizations/DataMechanics/Forehead/NormLength/Annotation/ErrorEncoding_Annotation_R6C3.jpeg]

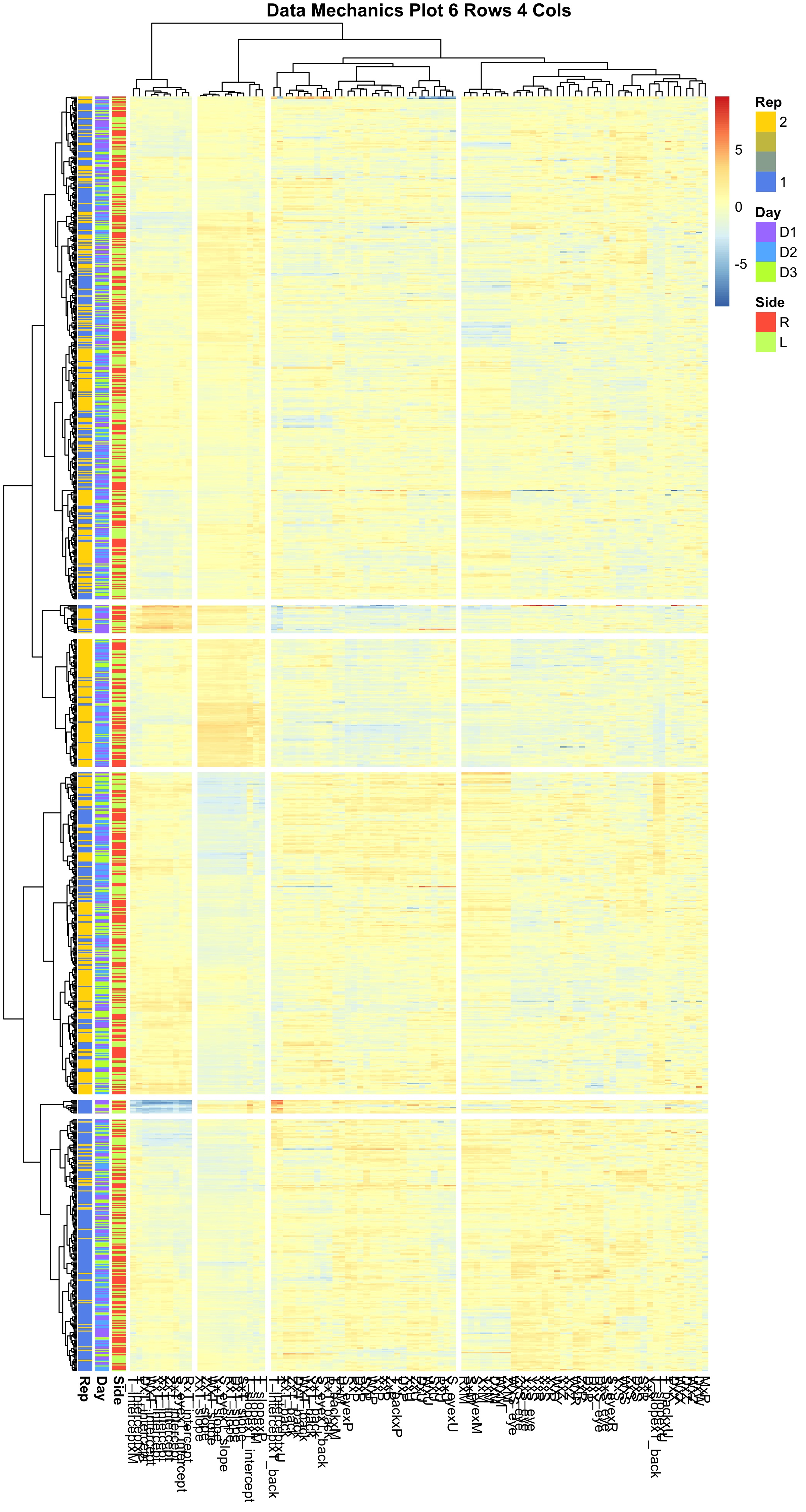

Supplement: Supplementary file 1 [file sensors-22-08347-s001.zip › SupplementalMaterials/Visualizations/DataMechanics/Forehead/NormLength/Annotation/ErrorEncoding_Annotation_R6C4.jpeg]

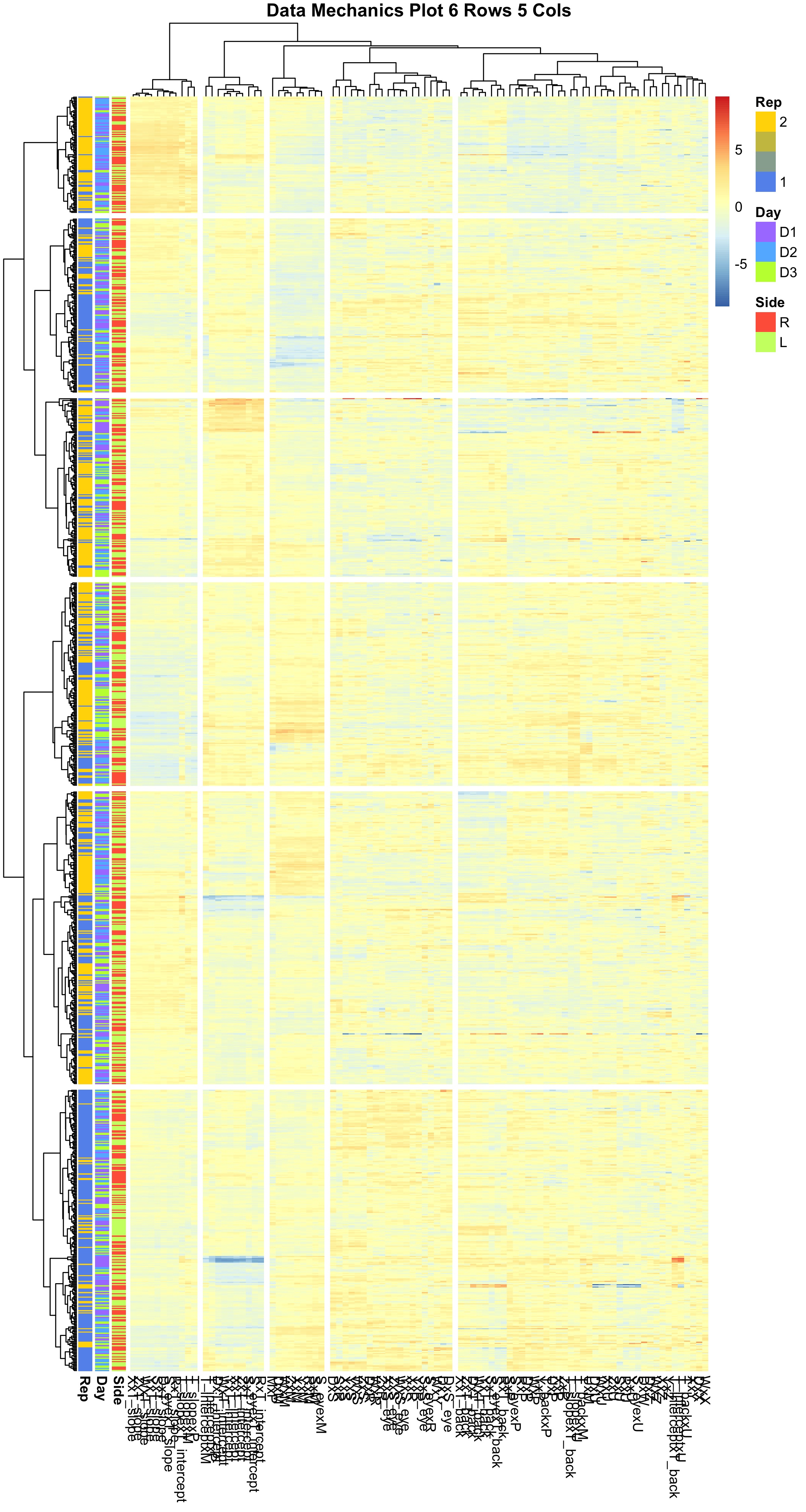

Supplement: Supplementary file 1 [file sensors-22-08347-s001.zip › SupplementalMaterials/Visualizations/DataMechanics/Forehead/NormLength/Annotation/ErrorEncoding_Annotation_R6C5.jpeg]

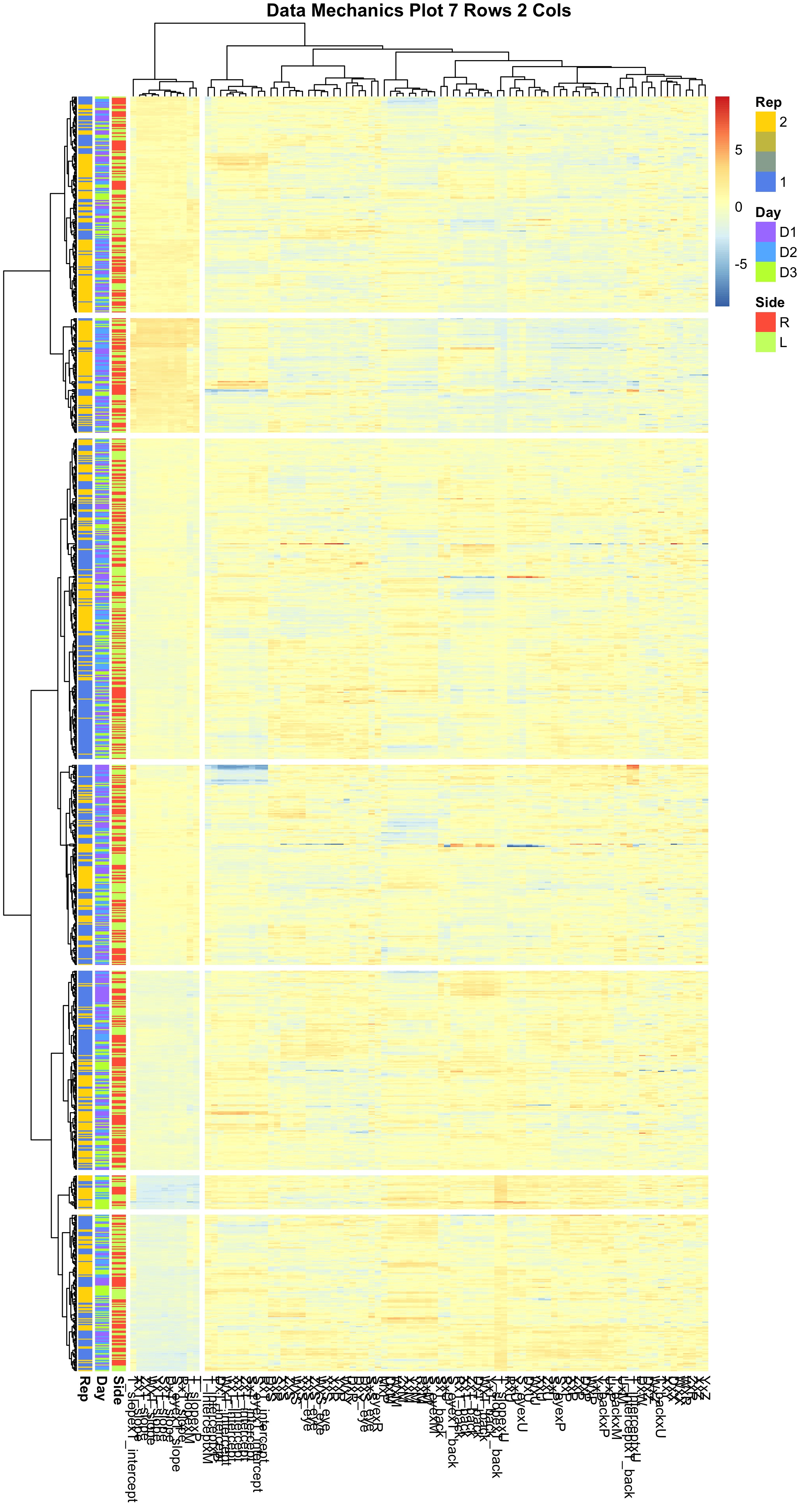

Supplement: Supplementary file 1 [file sensors-22-08347-s001.zip › SupplementalMaterials/Visualizations/DataMechanics/Forehead/NormLength/Annotation/ErrorEncoding_Annotation_R7C2.jpeg]

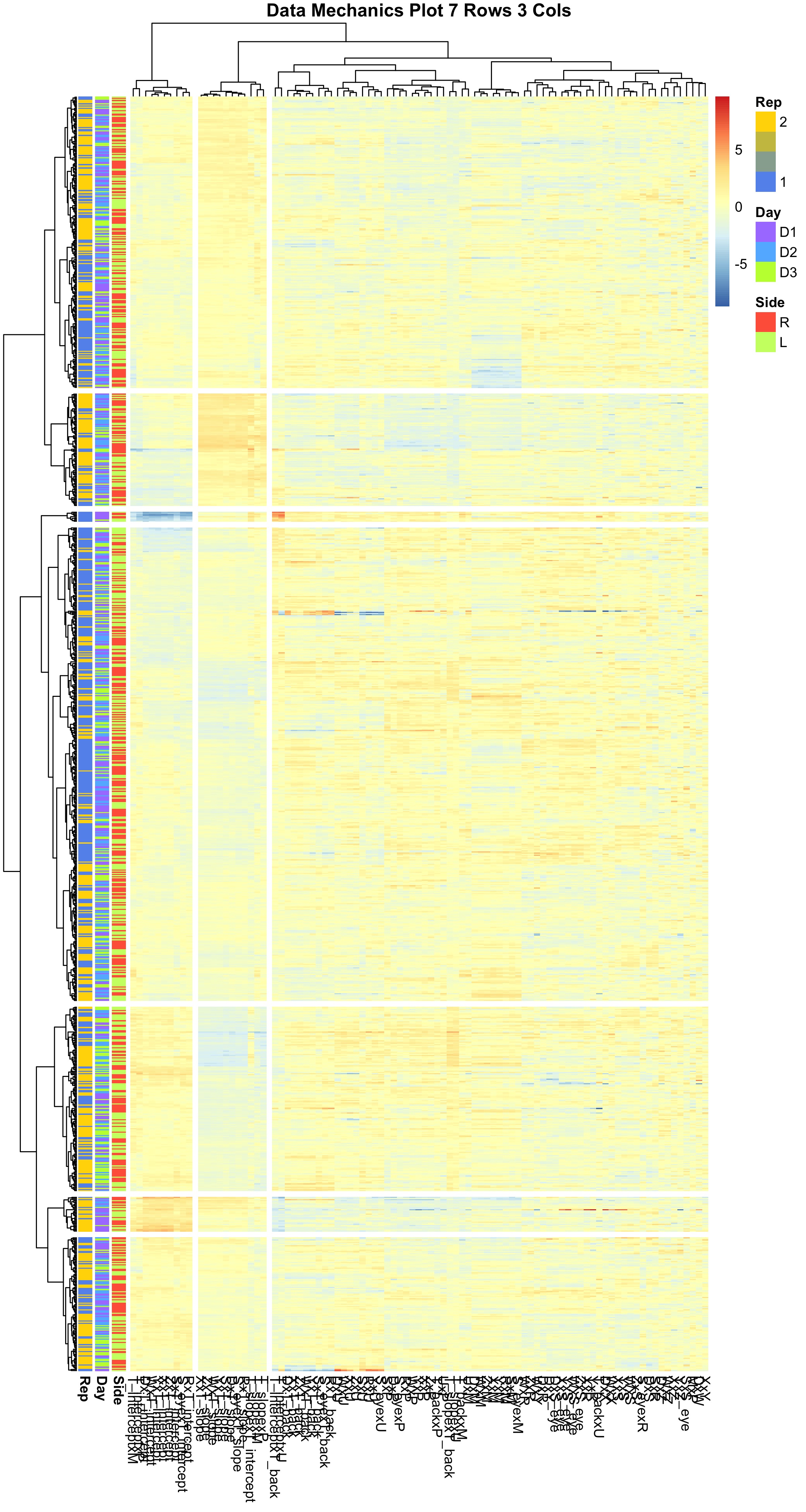

Supplement: Supplementary file 1 [file sensors-22-08347-s001.zip › SupplementalMaterials/Visualizations/DataMechanics/Forehead/NormLength/Annotation/ErrorEncoding_Annotation_R7C3.jpeg]

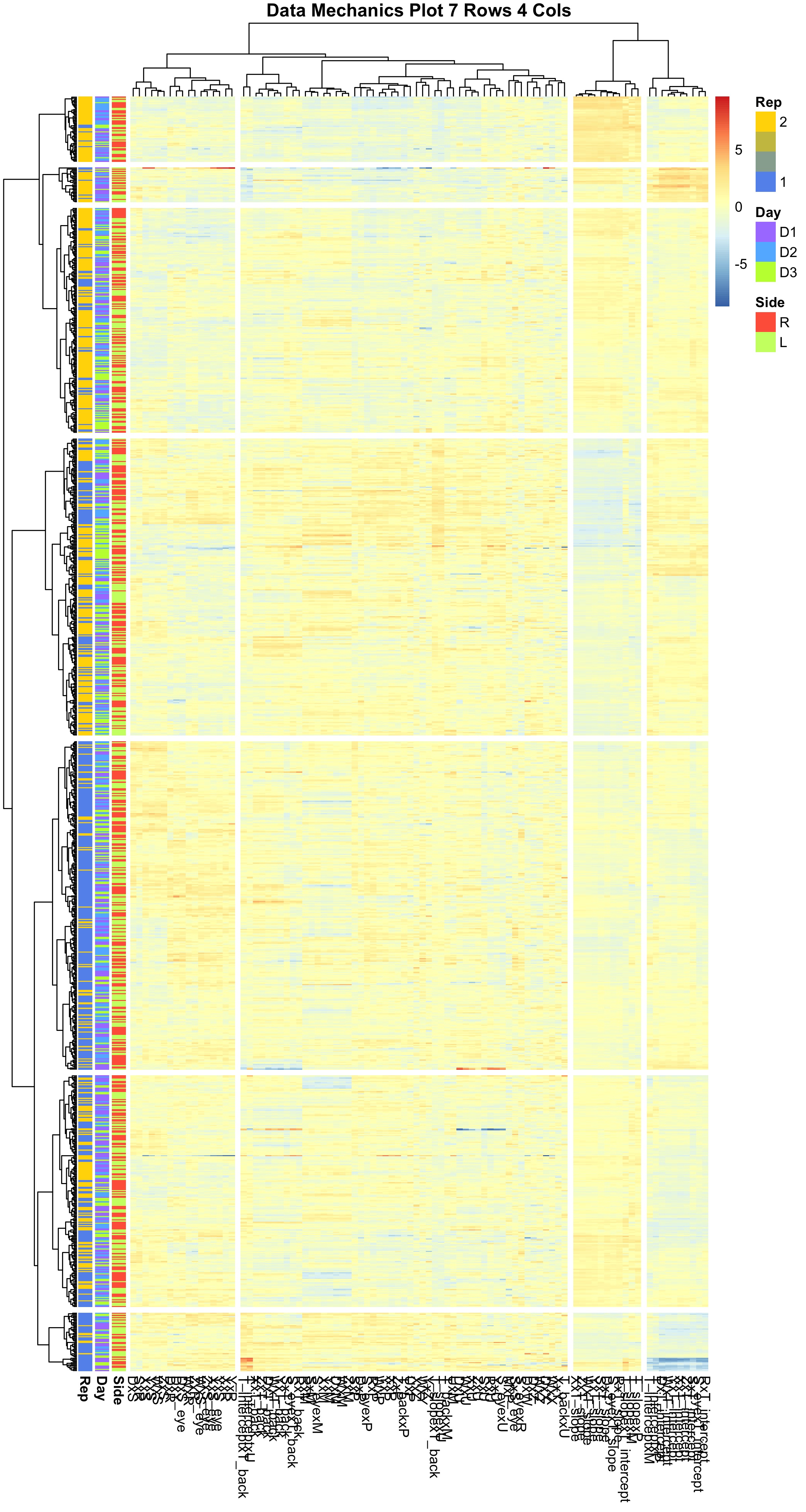

Supplement: Supplementary file 1 [file sensors-22-08347-s001.zip › SupplementalMaterials/Visualizations/DataMechanics/Forehead/NormLength/Annotation/ErrorEncoding_Annotation_R7C4.jpeg]

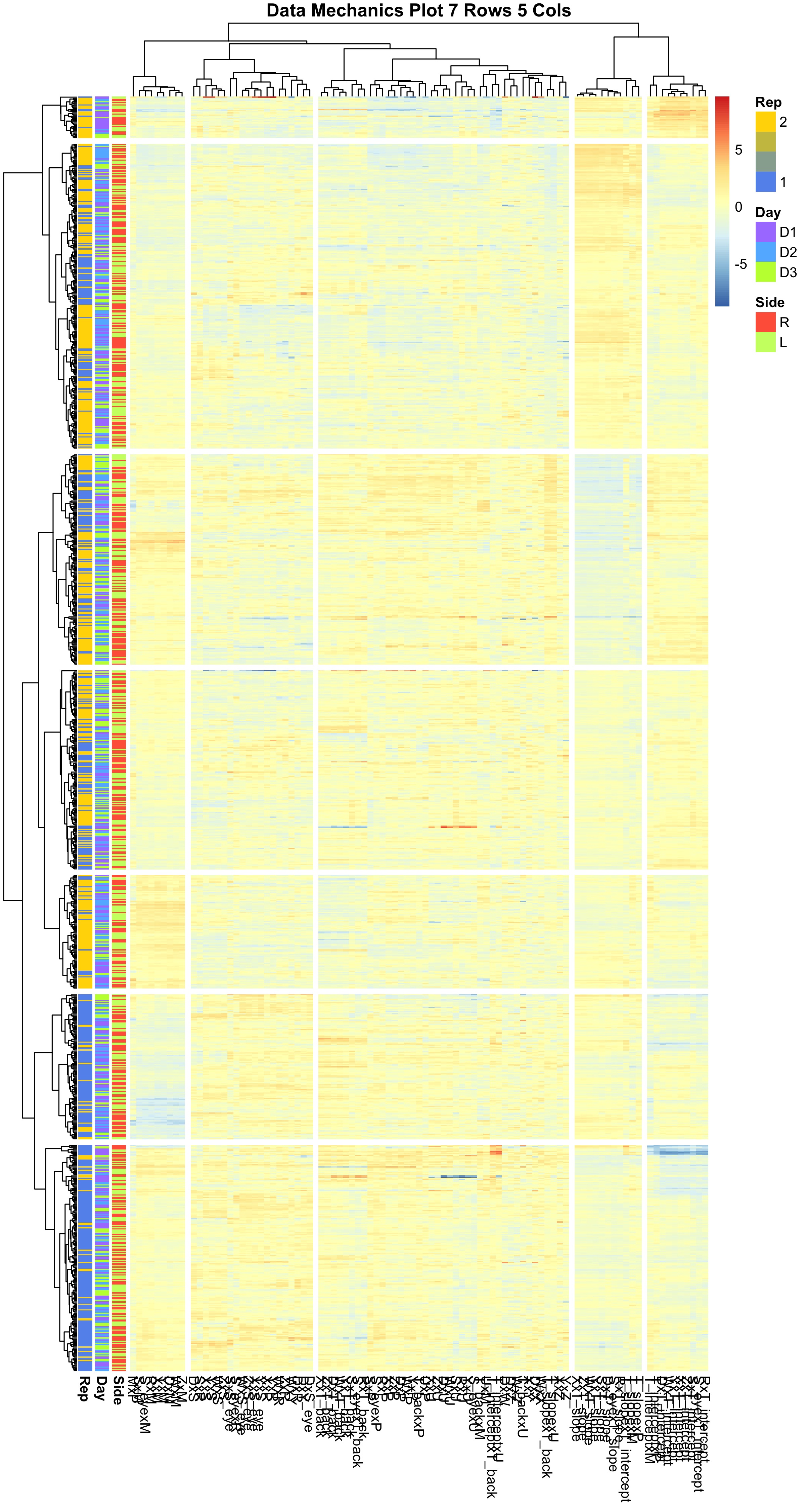

Supplement: Supplementary file 1 [file sensors-22-08347-s001.zip › SupplementalMaterials/Visualizations/DataMechanics/Forehead/NormLength/Annotation/ErrorEncoding_Annotation_R7C5.jpeg]

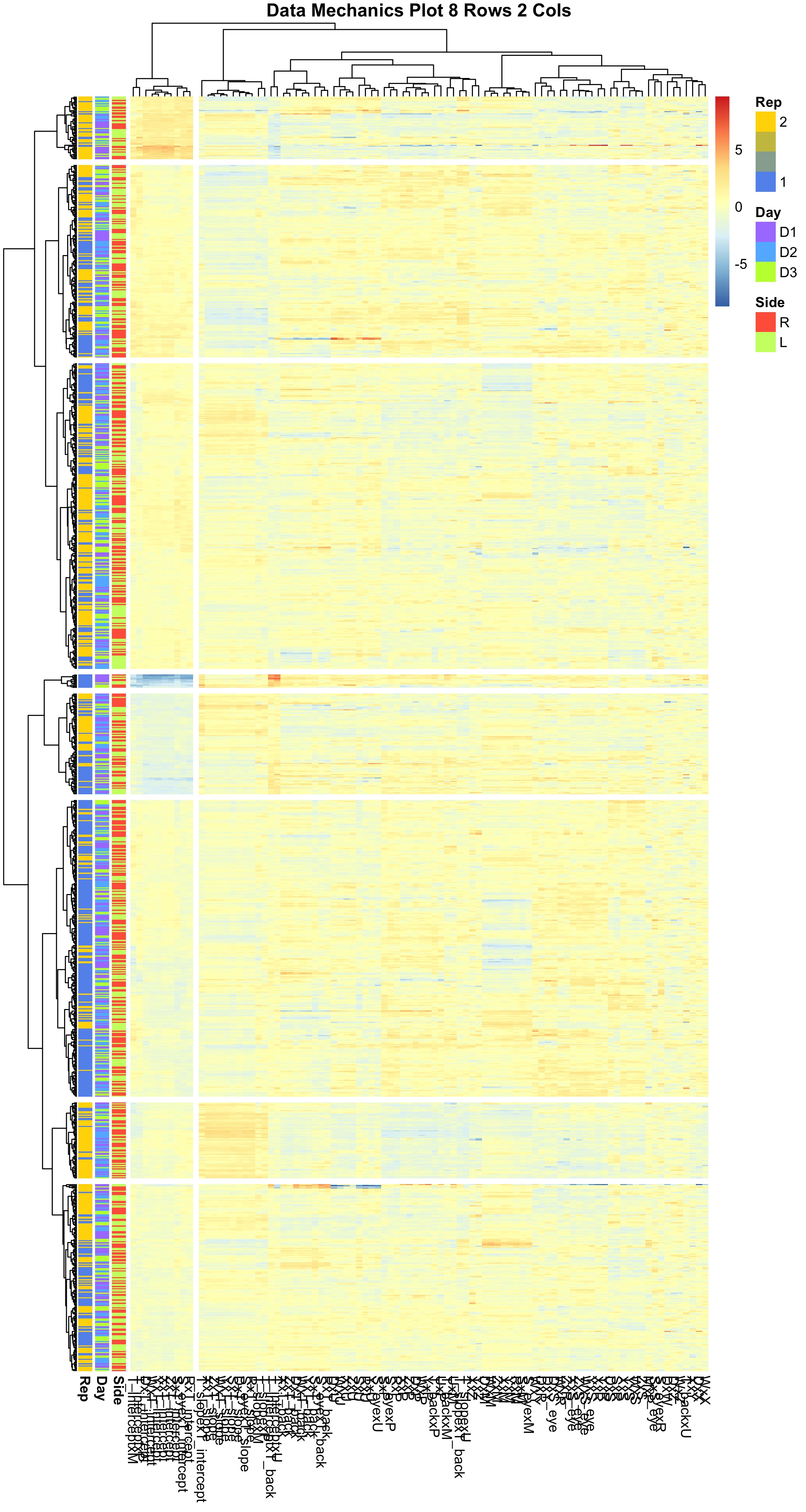

Supplement: Supplementary file 1 [file sensors-22-08347-s001.zip › SupplementalMaterials/Visualizations/DataMechanics/Forehead/NormLength/Annotation/ErrorEncoding_Annotation_R8C2.jpeg]

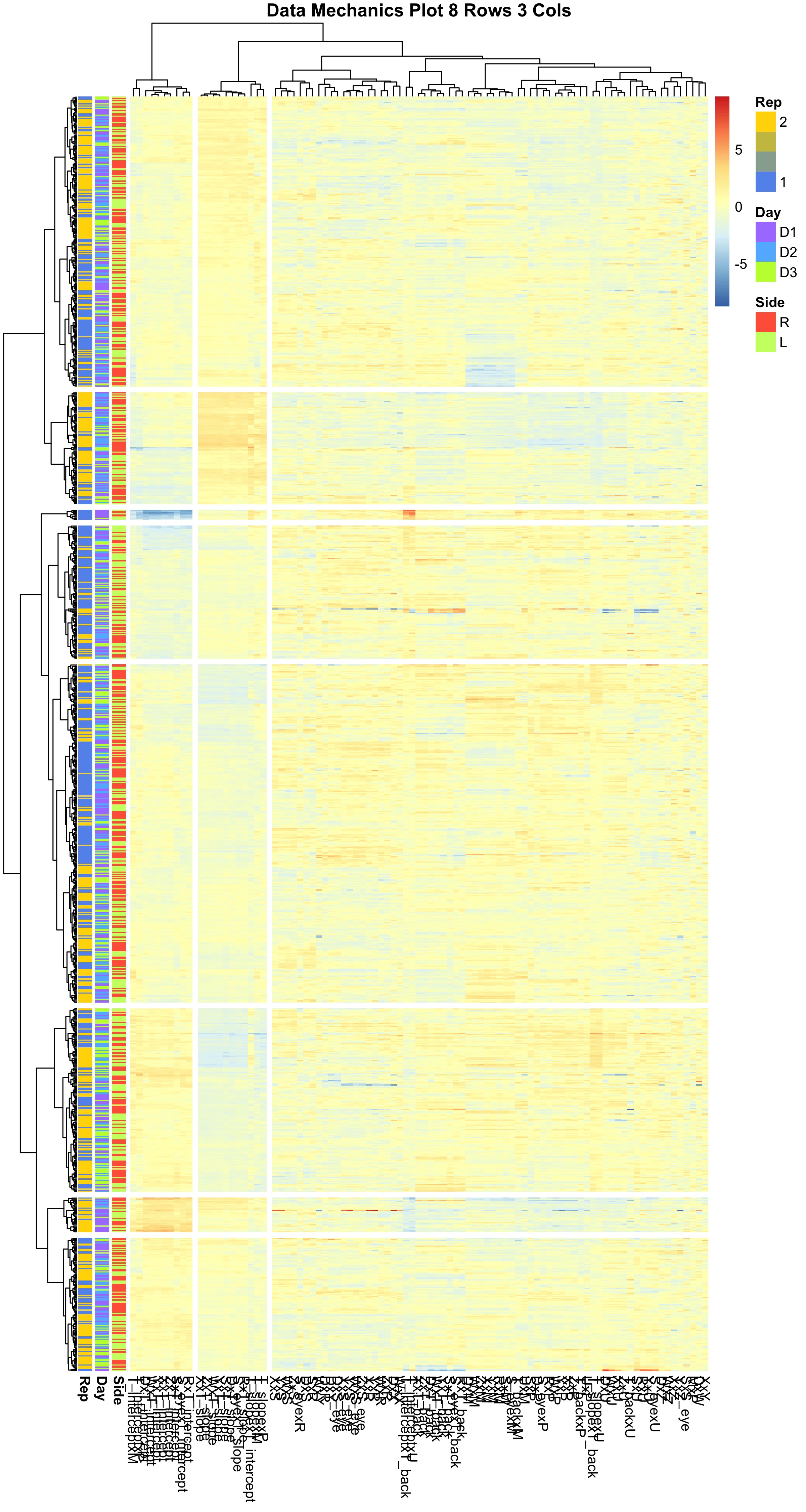

Supplement: Supplementary file 1 [file sensors-22-08347-s001.zip › SupplementalMaterials/Visualizations/DataMechanics/Forehead/NormLength/Annotation/ErrorEncoding_Annotation_R8C3.jpeg]

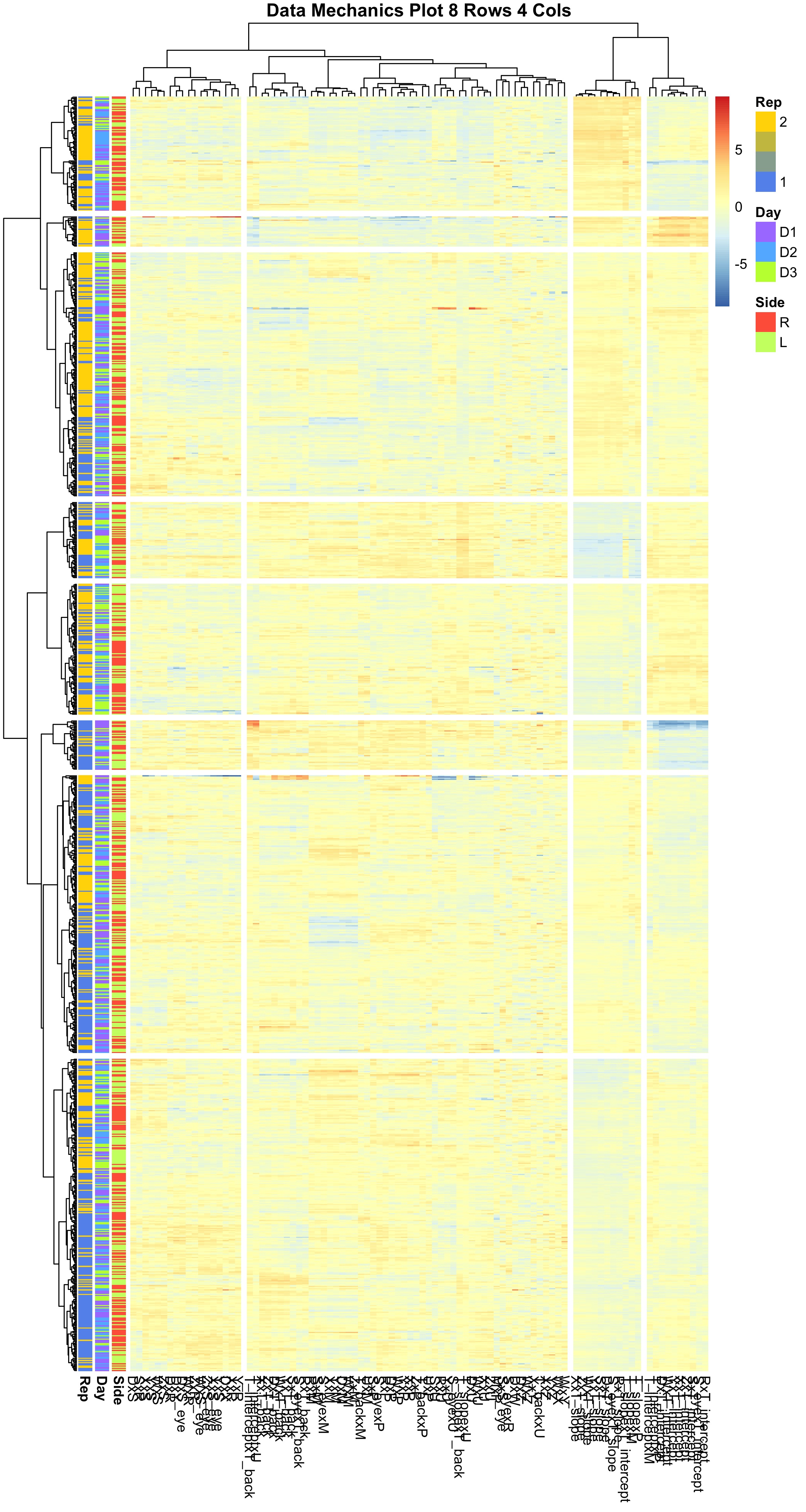

Supplement: Supplementary file 1 [file sensors-22-08347-s001.zip › SupplementalMaterials/Visualizations/DataMechanics/Forehead/NormLength/Annotation/ErrorEncoding_Annotation_R8C4.jpeg]

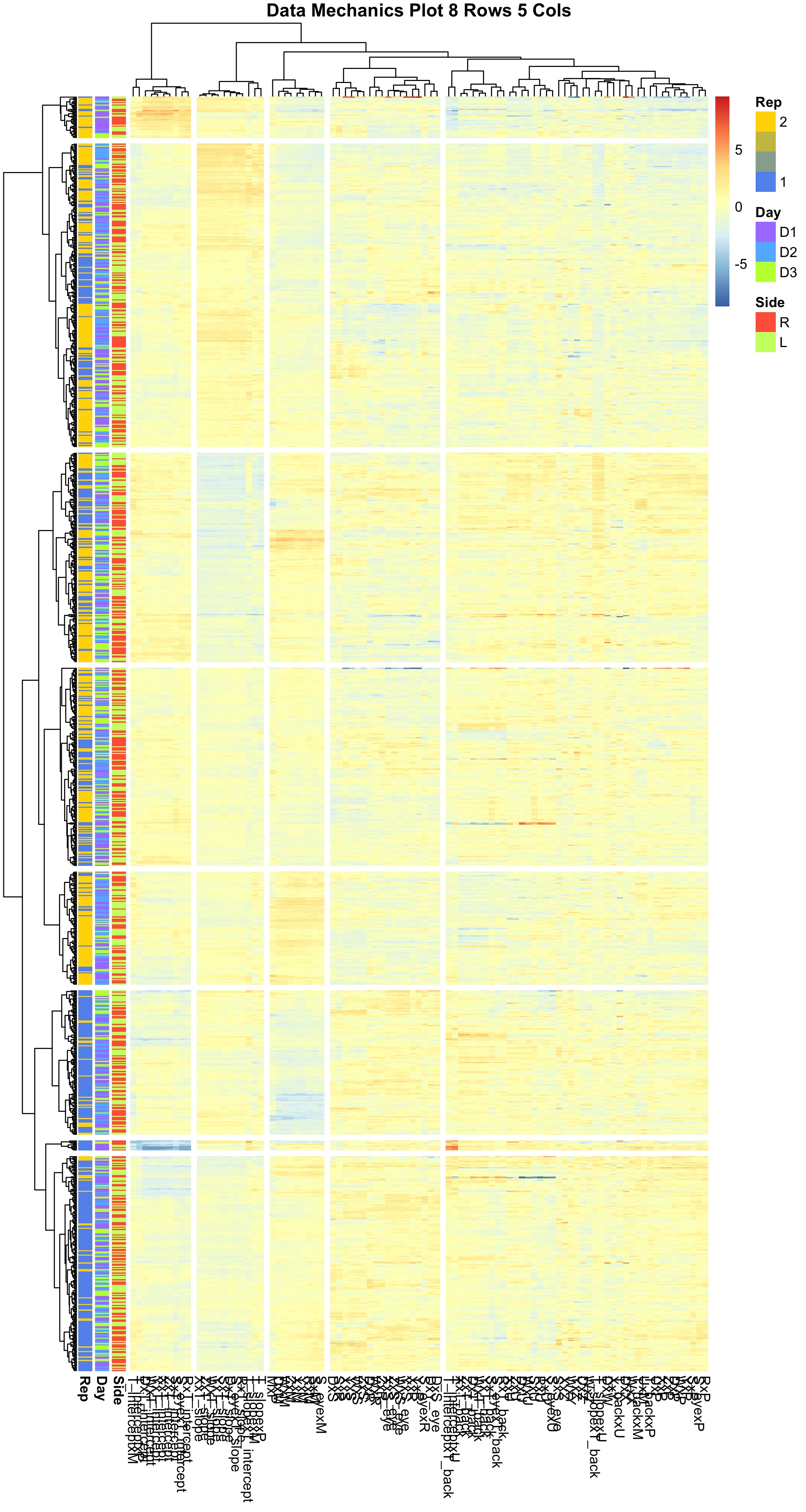

Supplement: Supplementary file 1 [file sensors-22-08347-s001.zip › SupplementalMaterials/Visualizations/DataMechanics/Forehead/NormLength/Annotation/ErrorEncoding_Annotation_R8C5.jpeg]

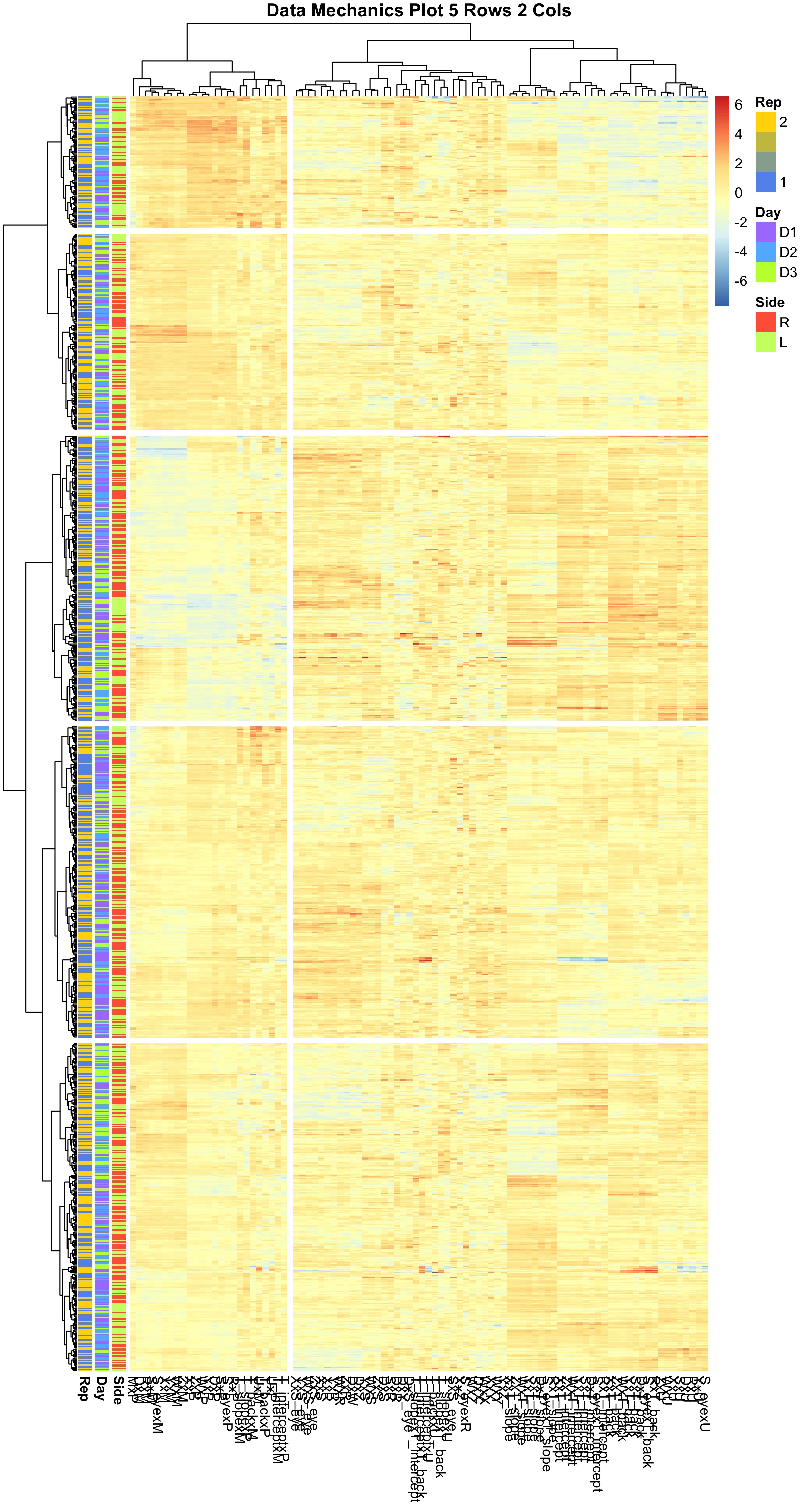

Supplement: Supplementary file 1 [file sensors-22-08347-s001.zip › SupplementalMaterials/Visualizations/DataMechanics/Forehead/NormLength/Image/ErrorEncoding_Image_R5C2.jpeg]

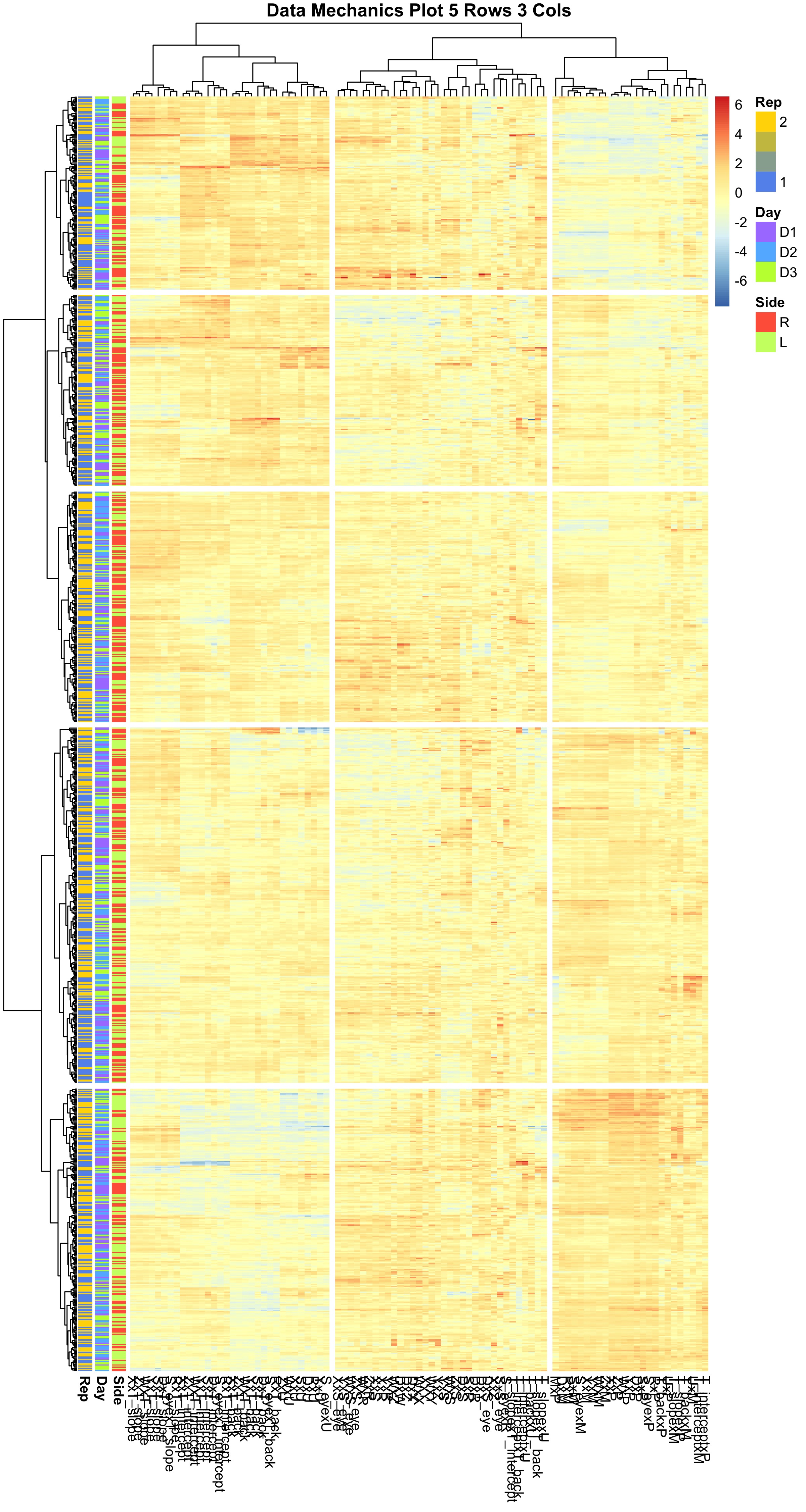

Supplement: Supplementary file 1 [file sensors-22-08347-s001.zip › SupplementalMaterials/Visualizations/DataMechanics/Forehead/NormLength/Image/ErrorEncoding_Image_R5C3.jpeg]

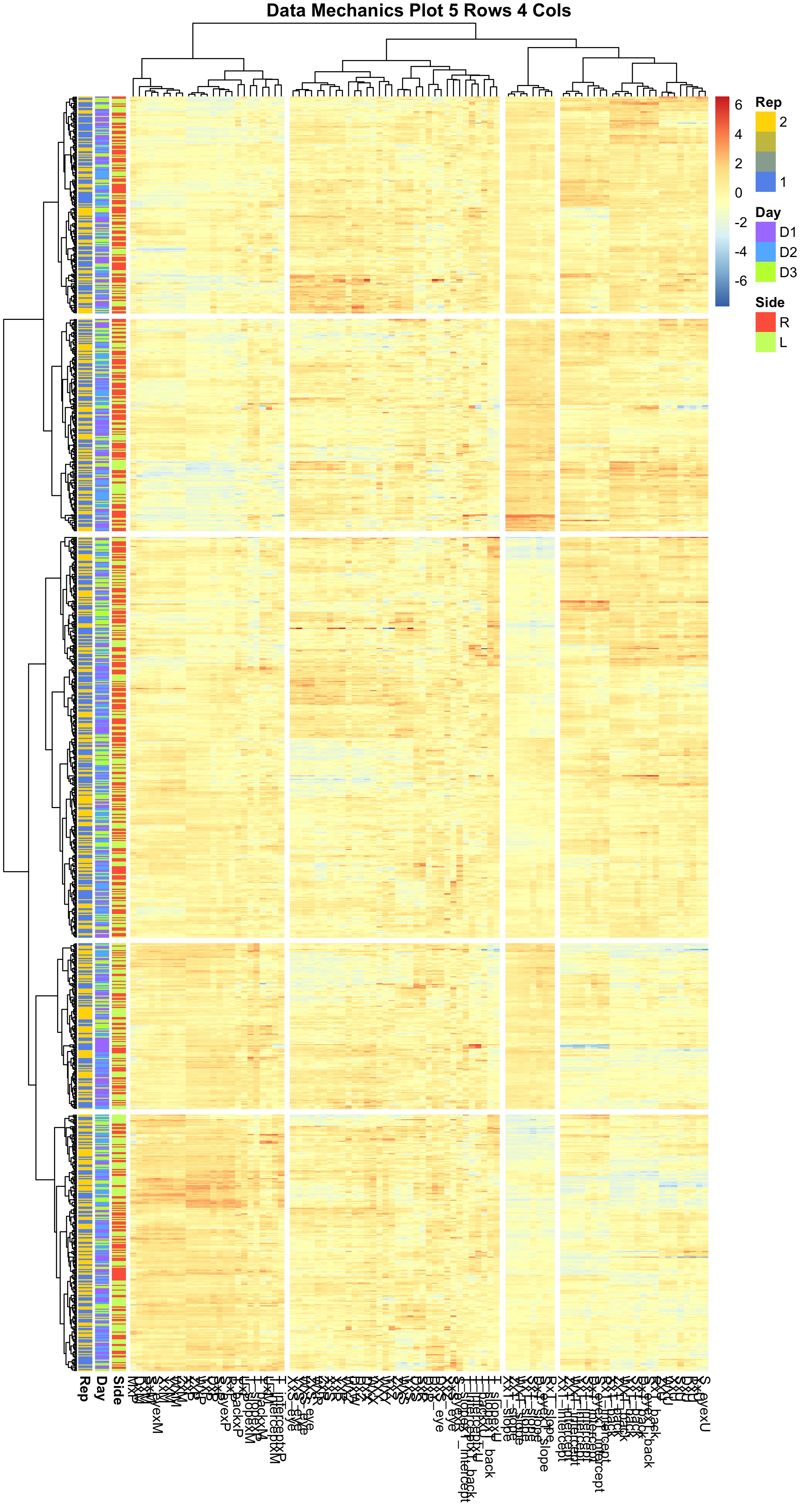

Supplement: Supplementary file 1 [file sensors-22-08347-s001.zip › SupplementalMaterials/Visualizations/DataMechanics/Forehead/NormLength/Image/ErrorEncoding_Image_R5C4.jpeg]

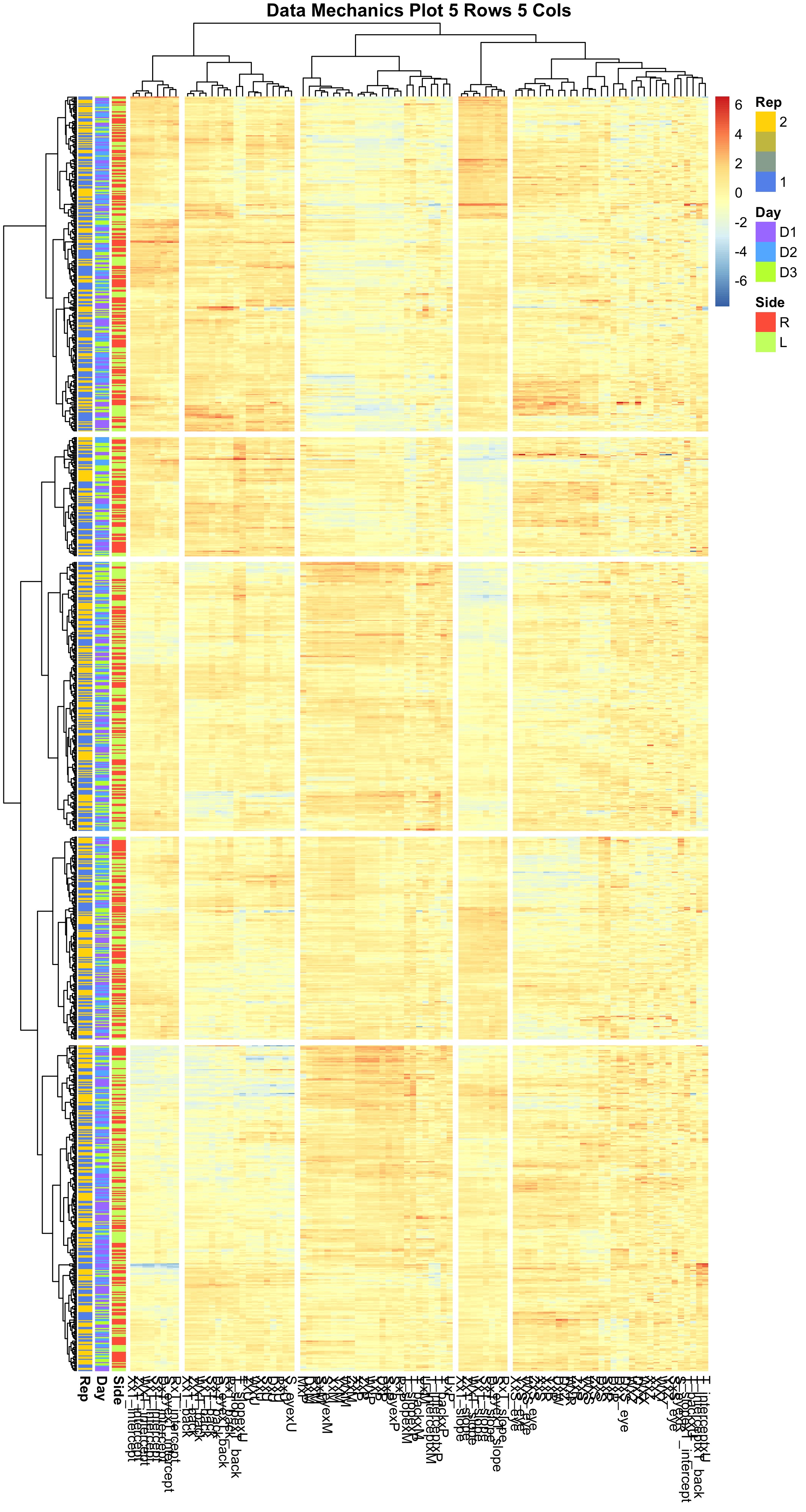

Supplement: Supplementary file 1 [file sensors-22-08347-s001.zip › SupplementalMaterials/Visualizations/DataMechanics/Forehead/NormLength/Image/ErrorEncoding_Image_R5C5.jpeg]

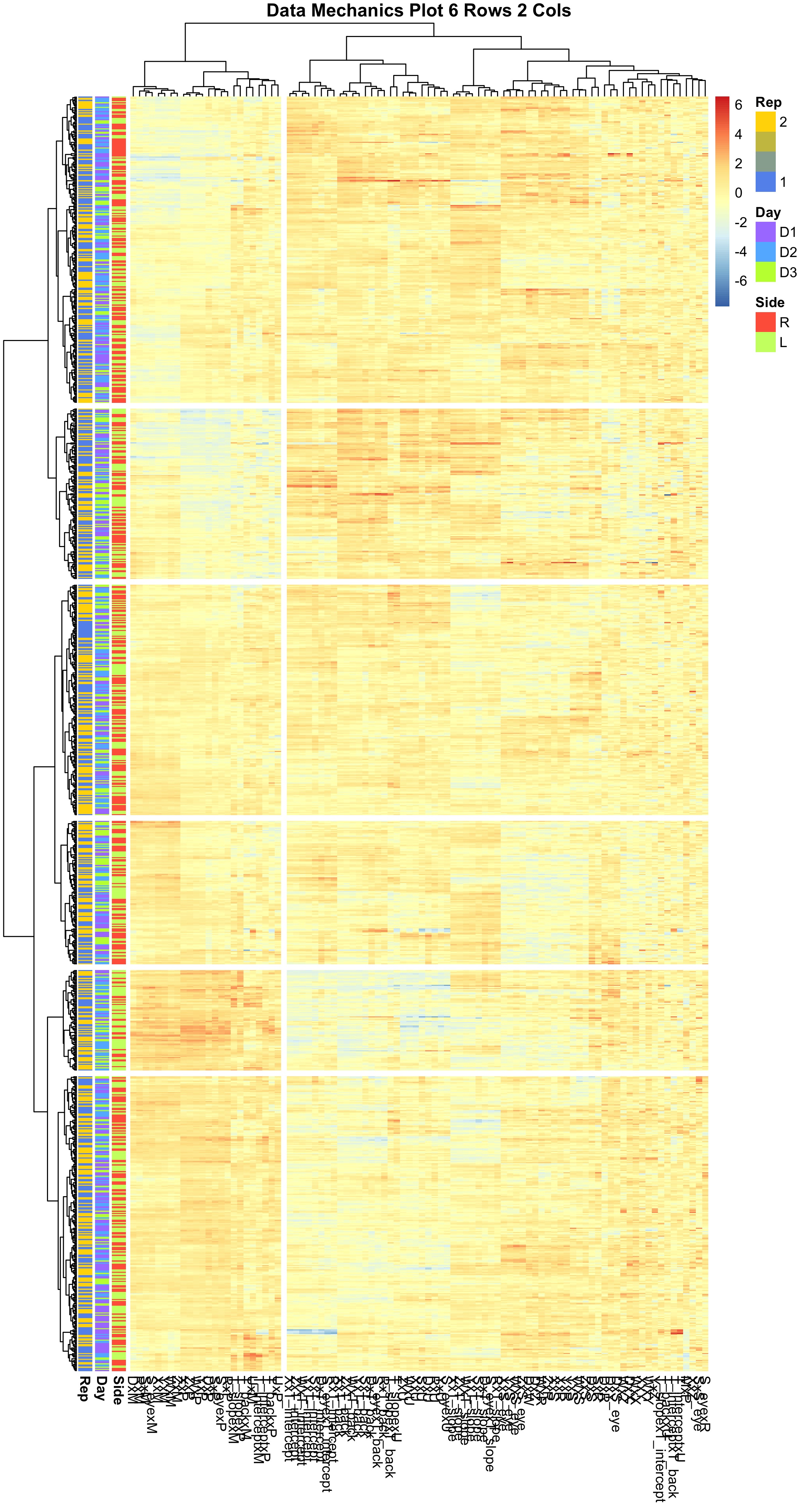

Supplement: Supplementary file 1 [file sensors-22-08347-s001.zip › SupplementalMaterials/Visualizations/DataMechanics/Forehead/NormLength/Image/ErrorEncoding_Image_R6C2.jpeg]

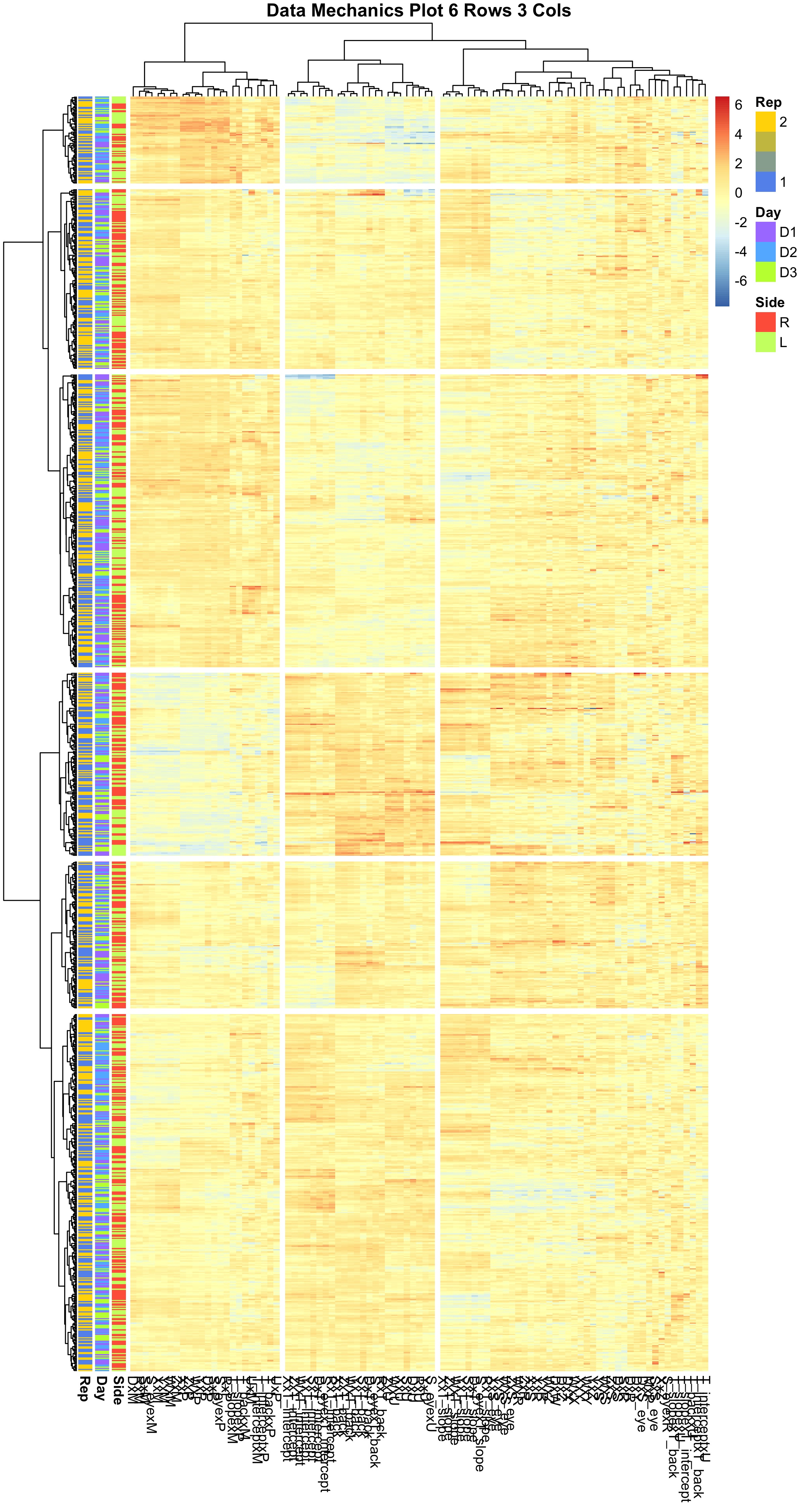

Supplement: Supplementary file 1 [file sensors-22-08347-s001.zip › SupplementalMaterials/Visualizations/DataMechanics/Forehead/NormLength/Image/ErrorEncoding_Image_R6C3.jpeg]

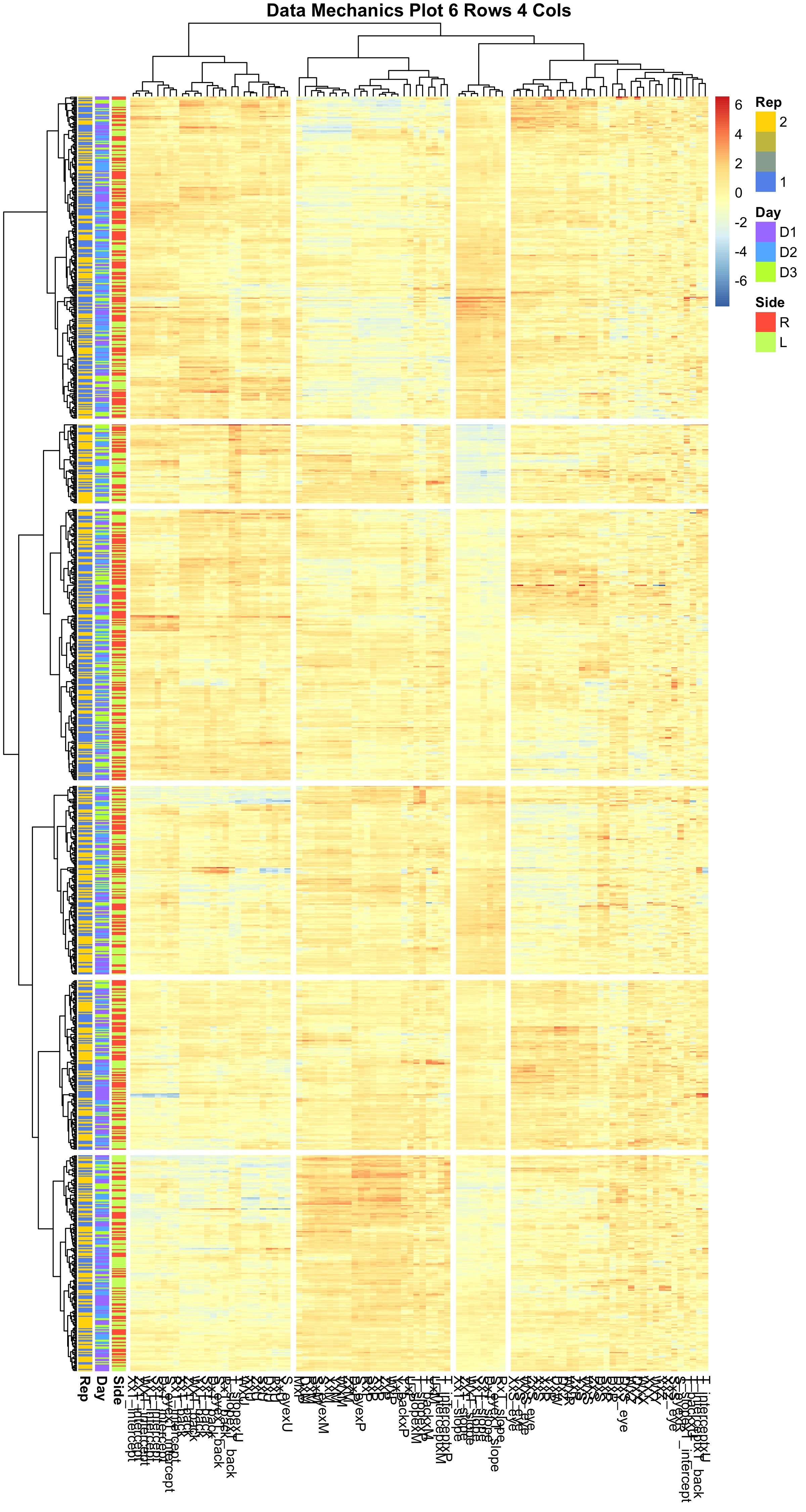

Supplement: Supplementary file 1 [file sensors-22-08347-s001.zip › SupplementalMaterials/Visualizations/DataMechanics/Forehead/NormLength/Image/ErrorEncoding_Image_R6C4.jpeg]

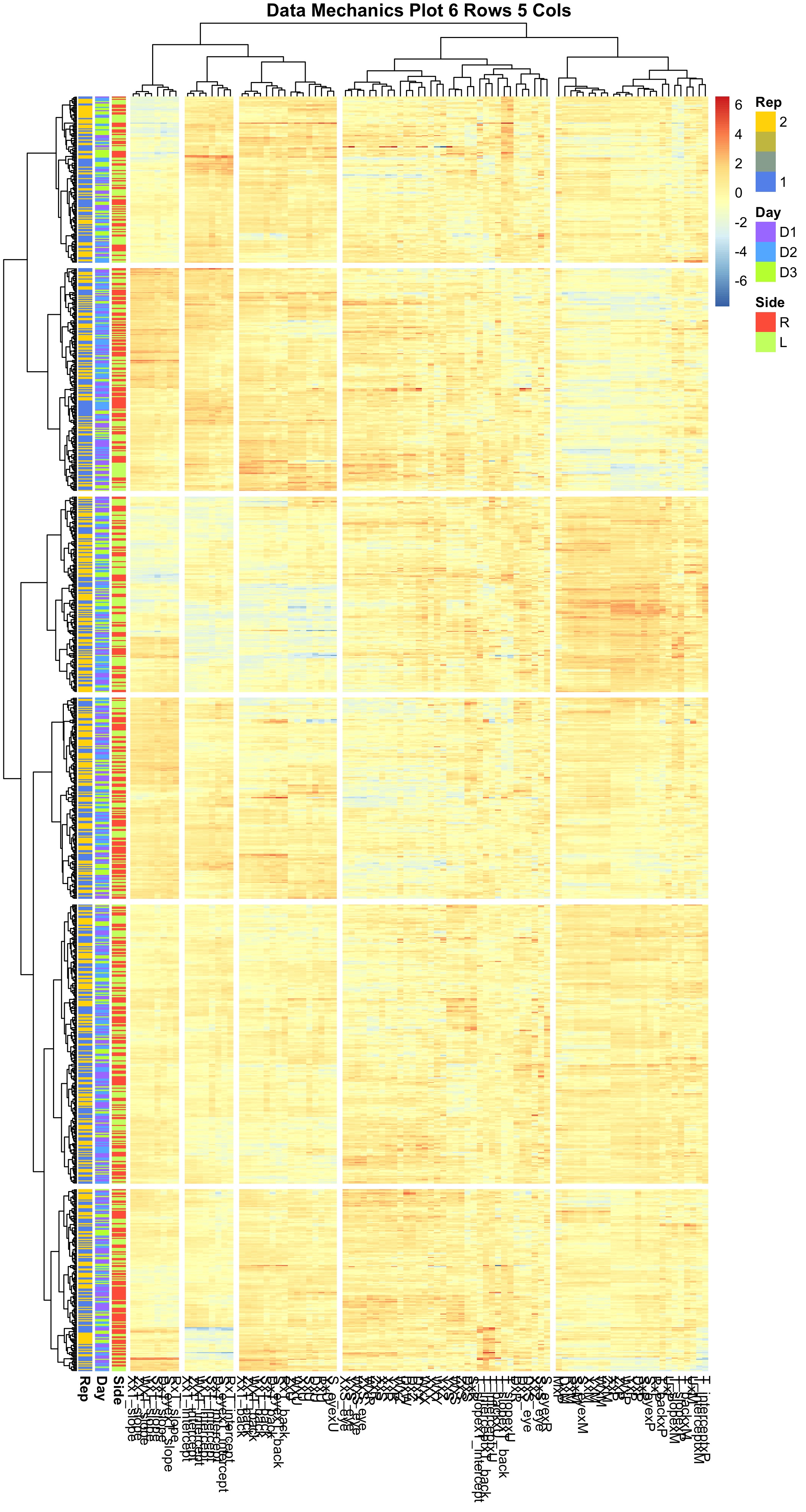

Supplement: Supplementary file 1 [file sensors-22-08347-s001.zip › SupplementalMaterials/Visualizations/DataMechanics/Forehead/NormLength/Image/ErrorEncoding_Image_R6C5.jpeg]

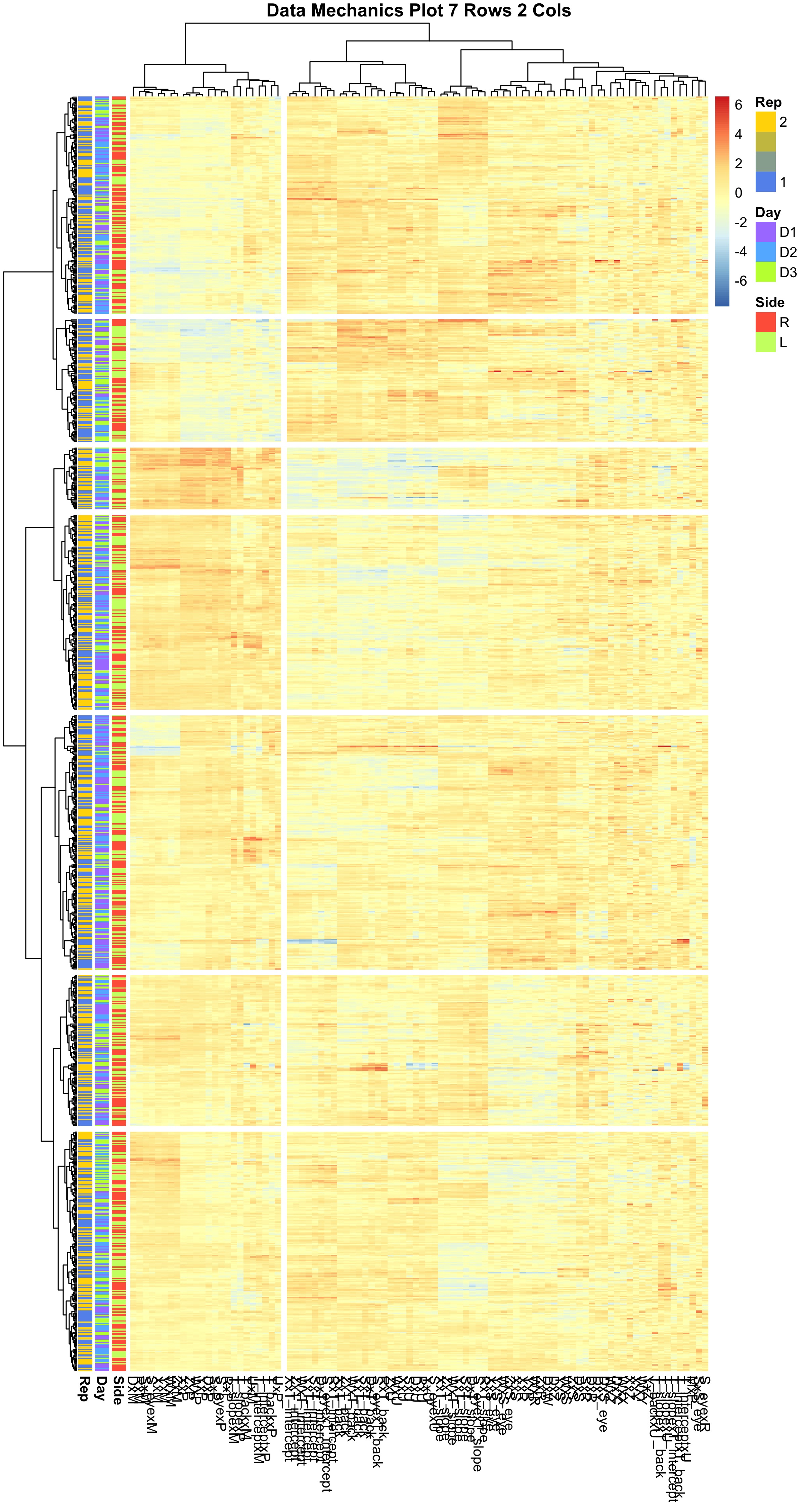

Supplement: Supplementary file 1 [file sensors-22-08347-s001.zip › SupplementalMaterials/Visualizations/DataMechanics/Forehead/NormLength/Image/ErrorEncoding_Image_R7C2.jpeg]

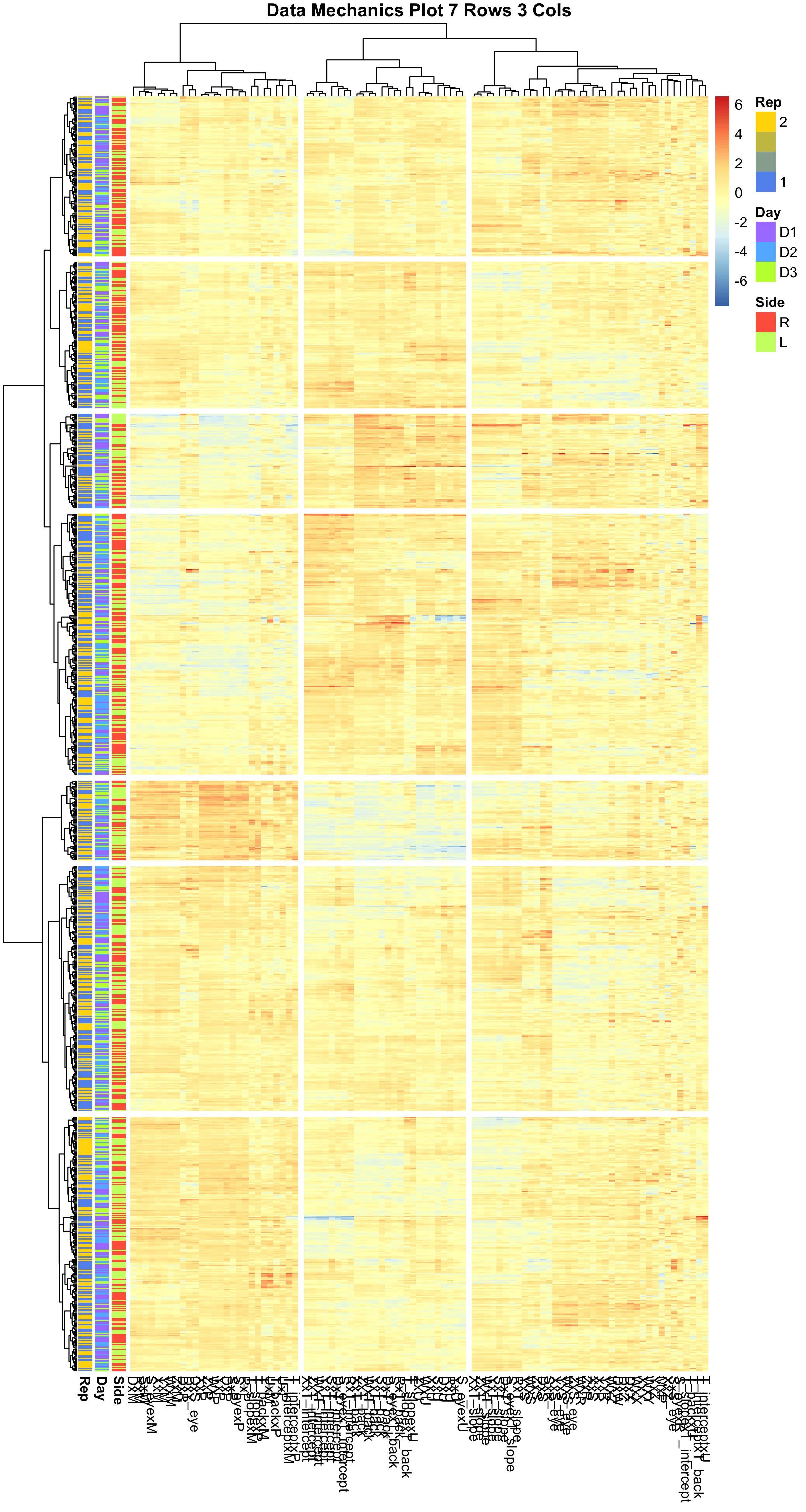

Supplement: Supplementary file 1 [file sensors-22-08347-s001.zip › SupplementalMaterials/Visualizations/DataMechanics/Forehead/NormLength/Image/ErrorEncoding_Image_R7C3.jpeg]

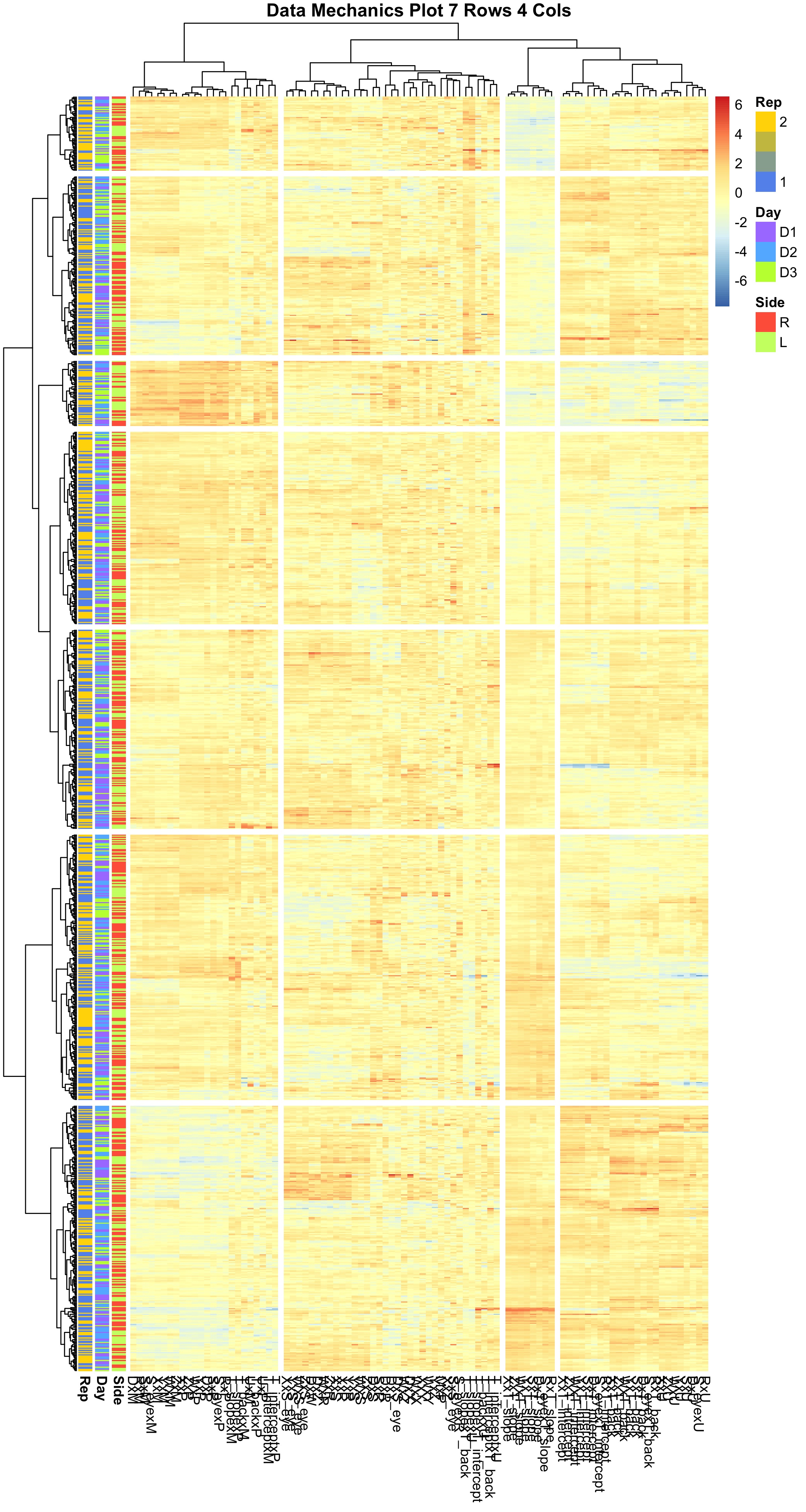

Supplement: Supplementary file 1 [file sensors-22-08347-s001.zip › SupplementalMaterials/Visualizations/DataMechanics/Forehead/NormLength/Image/ErrorEncoding_Image_R7C4.jpeg]

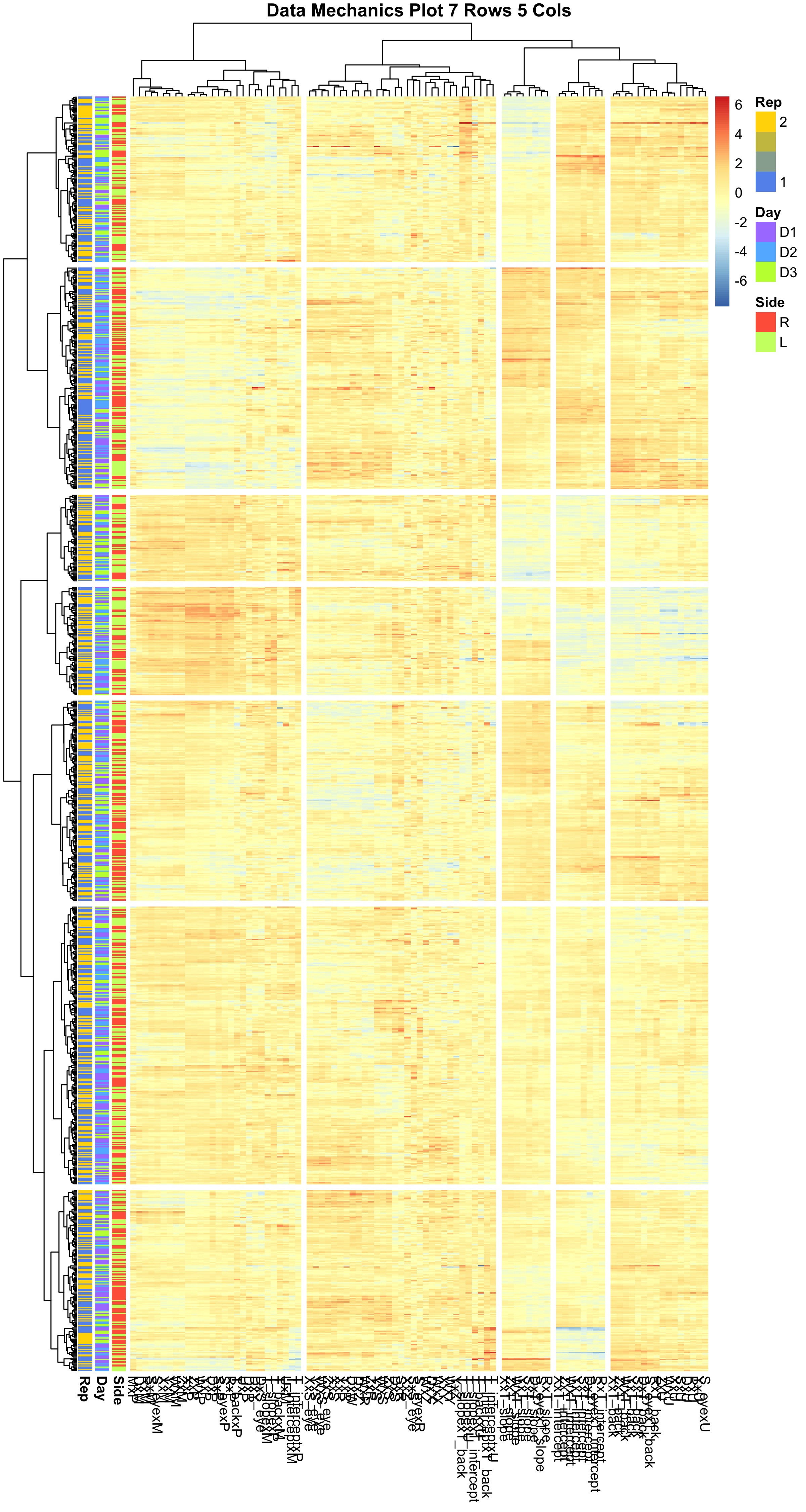

Supplement: Supplementary file 1 [file sensors-22-08347-s001.zip › SupplementalMaterials/Visualizations/DataMechanics/Forehead/NormLength/Image/ErrorEncoding_Image_R7C5.jpeg]

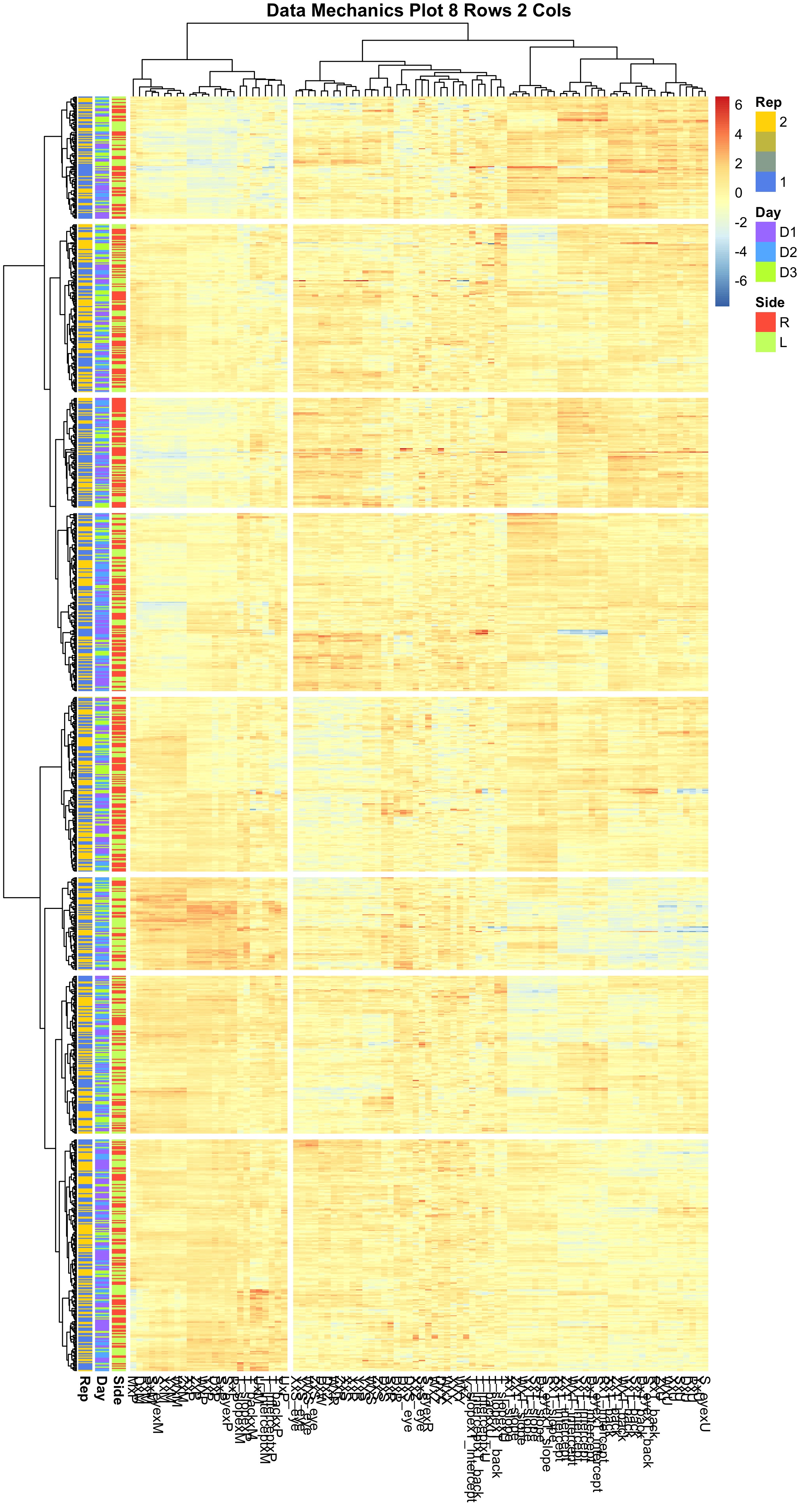

Supplement: Supplementary file 1 [file sensors-22-08347-s001.zip › SupplementalMaterials/Visualizations/DataMechanics/Forehead/NormLength/Image/ErrorEncoding_Image_R8C2.jpeg]

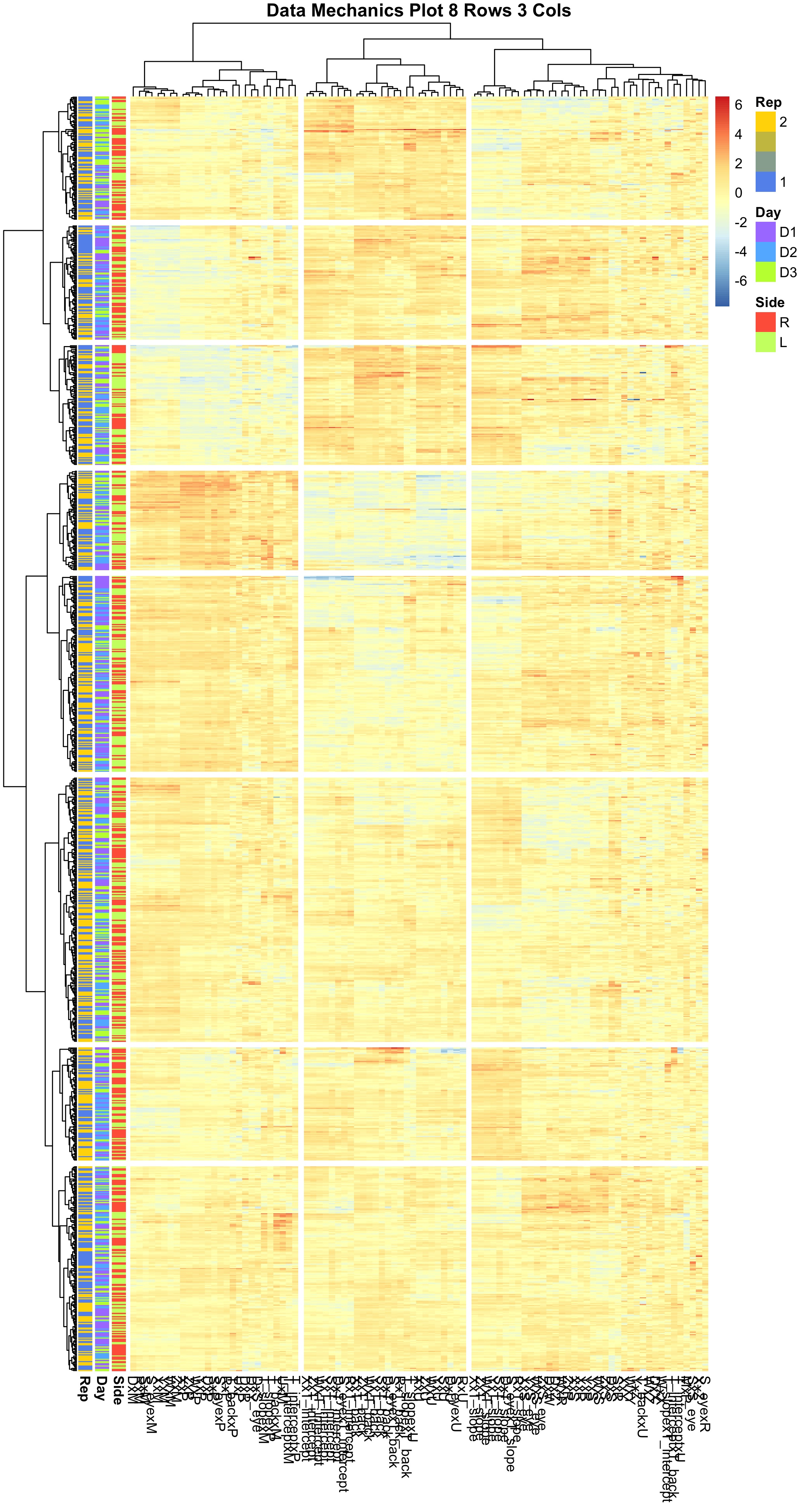

Supplement: Supplementary file 1 [file sensors-22-08347-s001.zip › SupplementalMaterials/Visualizations/DataMechanics/Forehead/NormLength/Image/ErrorEncoding_Image_R8C3.jpeg]

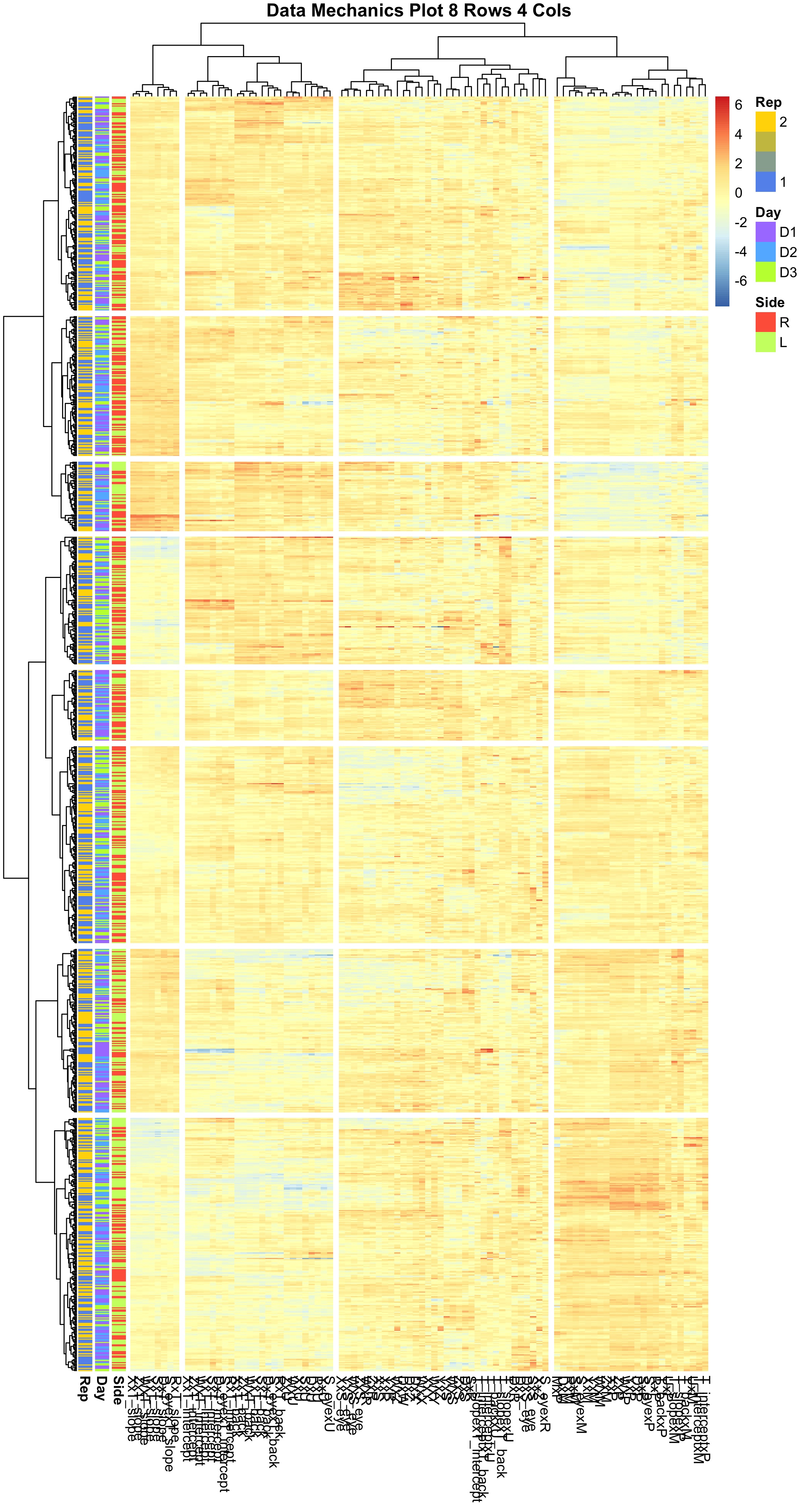

Supplement: Supplementary file 1 [file sensors-22-08347-s001.zip › SupplementalMaterials/Visualizations/DataMechanics/Forehead/NormLength/Image/ErrorEncoding_Image_R8C4.jpeg]

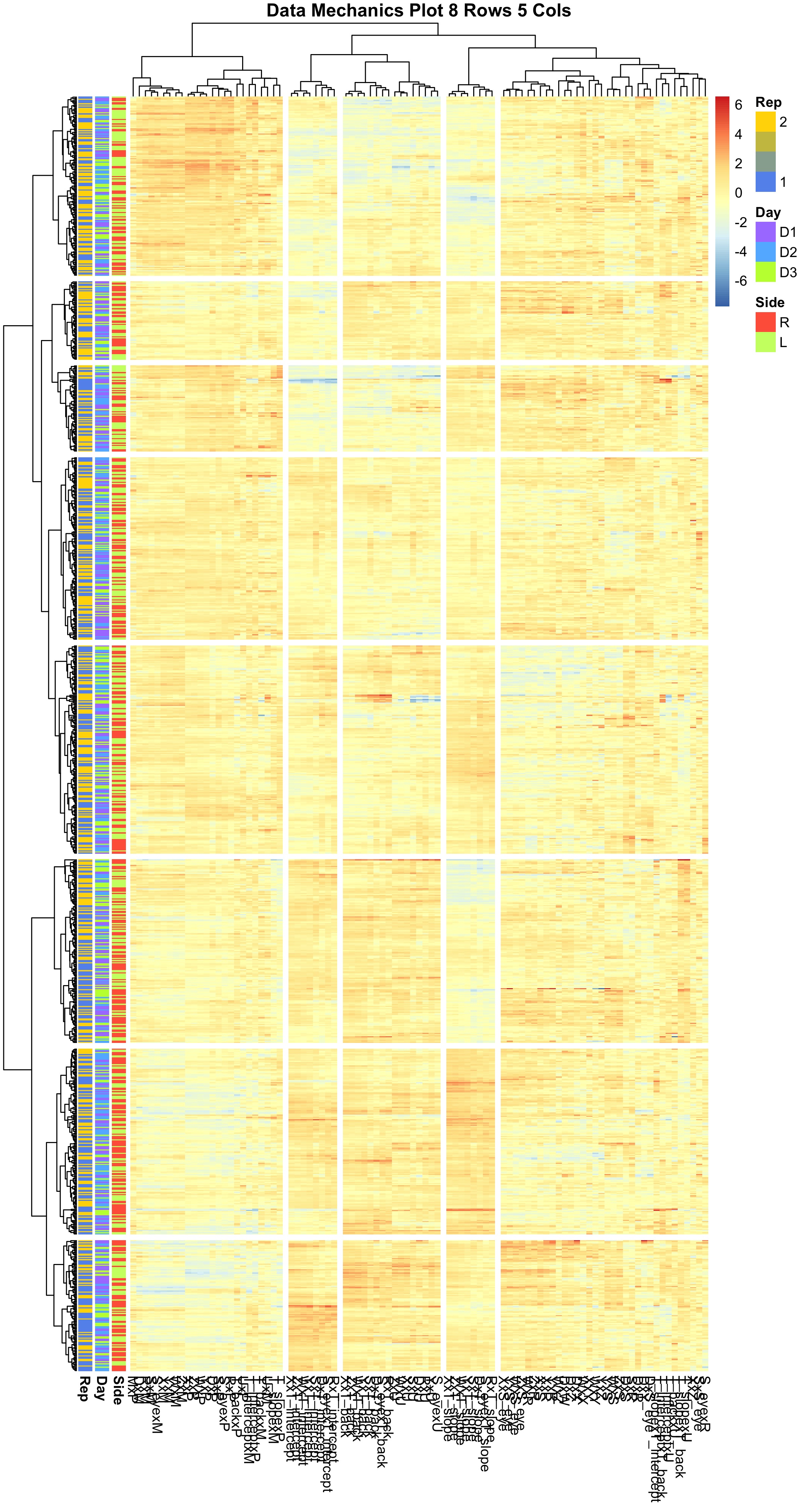

Supplement: Supplementary file 1 [file sensors-22-08347-s001.zip › SupplementalMaterials/Visualizations/DataMechanics/Forehead/NormLength/Image/ErrorEncoding_Image_R8C5.jpeg]

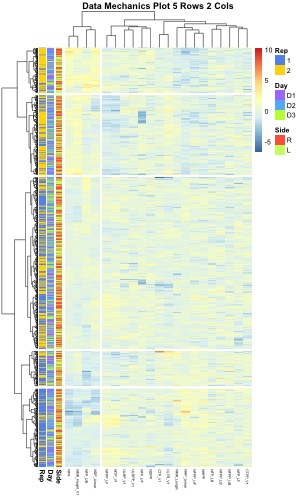

Supplement: Supplementary file 1 [file sensors-22-08347-s001.zip › SupplementalMaterials/Visualizations/DataMechanics/Muzzle/Geom/Annotation/ErrorEncoding_Annotation_R5C2.jpeg]

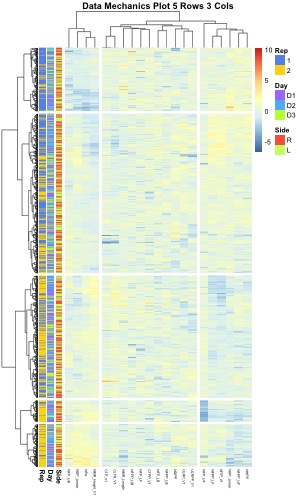

Supplement: Supplementary file 1 [file sensors-22-08347-s001.zip › SupplementalMaterials/Visualizations/DataMechanics/Muzzle/Geom/Annotation/ErrorEncoding_Annotation_R5C3.jpeg]

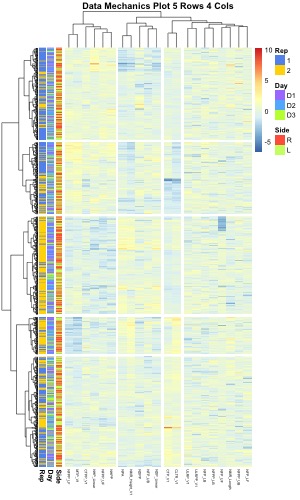

Supplement: Supplementary file 1 [file sensors-22-08347-s001.zip › SupplementalMaterials/Visualizations/DataMechanics/Muzzle/Geom/Annotation/ErrorEncoding_Annotation_R5C4.jpeg]

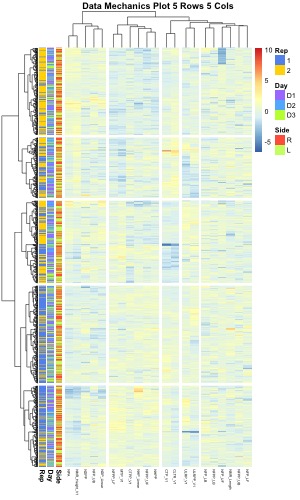

Supplement: Supplementary file 1 [file sensors-22-08347-s001.zip › SupplementalMaterials/Visualizations/DataMechanics/Muzzle/Geom/Annotation/ErrorEncoding_Annotation_R5C5.jpeg]

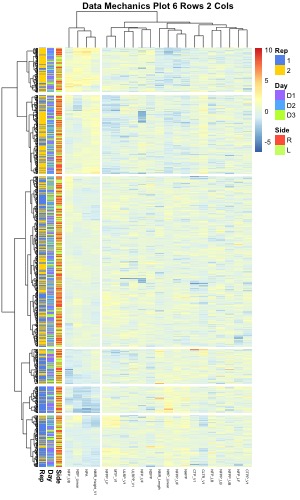

Supplement: Supplementary file 1 [file sensors-22-08347-s001.zip › SupplementalMaterials/Visualizations/DataMechanics/Muzzle/Geom/Annotation/ErrorEncoding_Annotation_R6C2.jpeg]

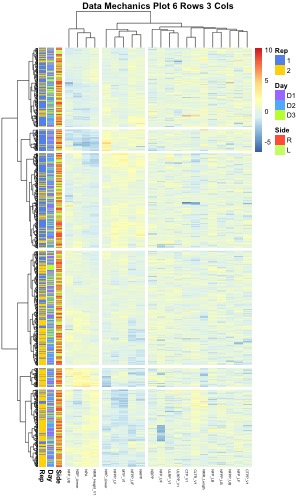

Supplement: Supplementary file 1 [file sensors-22-08347-s001.zip › SupplementalMaterials/Visualizations/DataMechanics/Muzzle/Geom/Annotation/ErrorEncoding_Annotation_R6C3.jpeg]

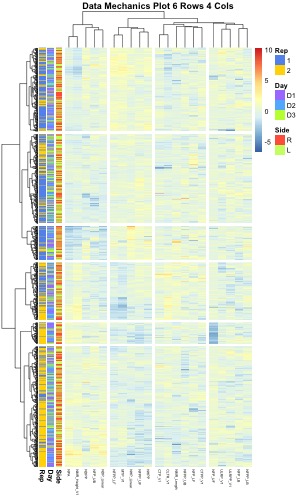

Supplement: Supplementary file 1 [file sensors-22-08347-s001.zip › SupplementalMaterials/Visualizations/DataMechanics/Muzzle/Geom/Annotation/ErrorEncoding_Annotation_R6C4.jpeg]

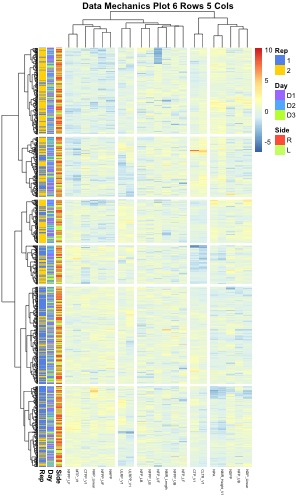

Supplement: Supplementary file 1 [file sensors-22-08347-s001.zip › SupplementalMaterials/Visualizations/DataMechanics/Muzzle/Geom/Annotation/ErrorEncoding_Annotation_R6C5.jpeg]

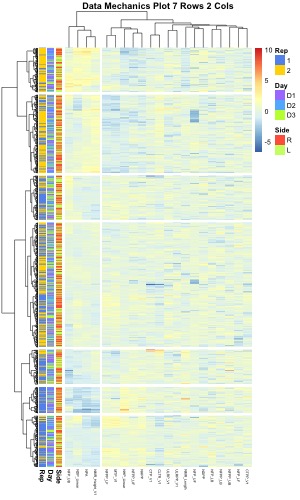

Supplement: Supplementary file 1 [file sensors-22-08347-s001.zip › SupplementalMaterials/Visualizations/DataMechanics/Muzzle/Geom/Annotation/ErrorEncoding_Annotation_R7C2.jpeg]

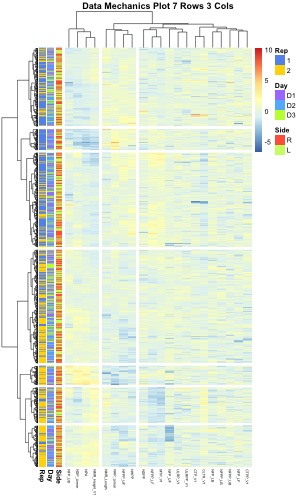

Supplement: Supplementary file 1 [file sensors-22-08347-s001.zip › SupplementalMaterials/Visualizations/DataMechanics/Muzzle/Geom/Annotation/ErrorEncoding_Annotation_R7C3.jpeg]

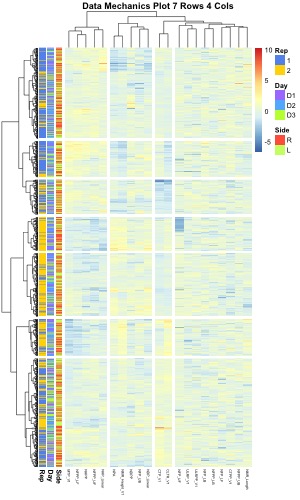

Supplement: Supplementary file 1 [file sensors-22-08347-s001.zip › SupplementalMaterials/Visualizations/DataMechanics/Muzzle/Geom/Annotation/ErrorEncoding_Annotation_R7C4.jpeg]

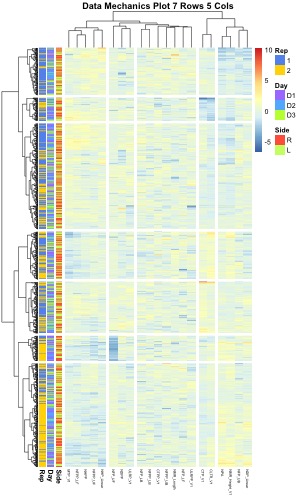

Supplement: Supplementary file 1 [file sensors-22-08347-s001.zip › SupplementalMaterials/Visualizations/DataMechanics/Muzzle/Geom/Annotation/ErrorEncoding_Annotation_R7C5.jpeg]

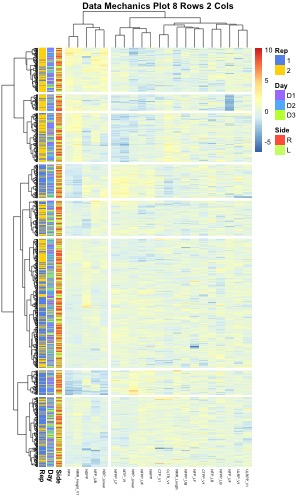

Supplement: Supplementary file 1 [file sensors-22-08347-s001.zip › SupplementalMaterials/Visualizations/DataMechanics/Muzzle/Geom/Annotation/ErrorEncoding_Annotation_R8C2.jpeg]

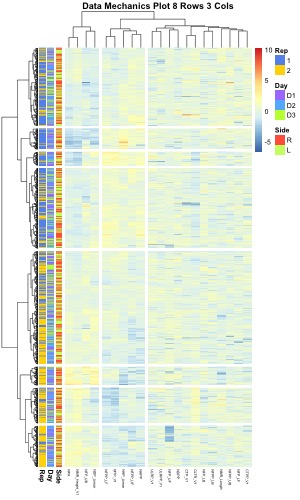

Supplement: Supplementary file 1 [file sensors-22-08347-s001.zip › SupplementalMaterials/Visualizations/DataMechanics/Muzzle/Geom/Annotation/ErrorEncoding_Annotation_R8C3.jpeg]

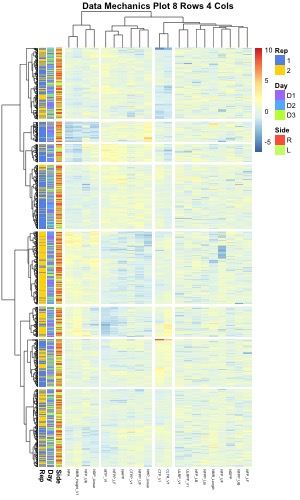

Supplement: Supplementary file 1 [file sensors-22-08347-s001.zip › SupplementalMaterials/Visualizations/DataMechanics/Muzzle/Geom/Annotation/ErrorEncoding_Annotation_R8C4.jpeg]

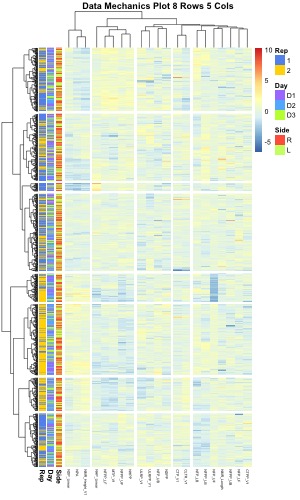

Supplement: Supplementary file 1 [file sensors-22-08347-s001.zip › SupplementalMaterials/Visualizations/DataMechanics/Muzzle/Geom/Annotation/ErrorEncoding_Annotation_R8C5.jpeg]

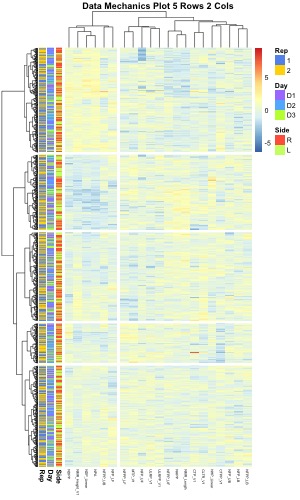

Supplement: Supplementary file 1 [file sensors-22-08347-s001.zip › SupplementalMaterials/Visualizations/DataMechanics/Muzzle/Geom/Image/ErrorEncoding_Image_R5C2.jpeg]

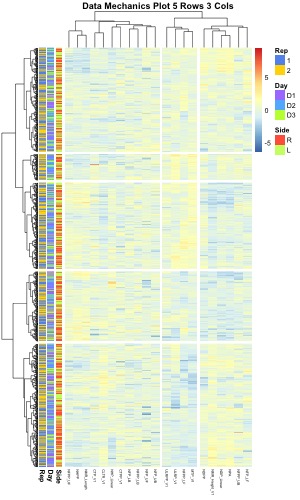

Supplement: Supplementary file 1 [file sensors-22-08347-s001.zip › SupplementalMaterials/Visualizations/DataMechanics/Muzzle/Geom/Image/ErrorEncoding_Image_R5C3.jpeg]

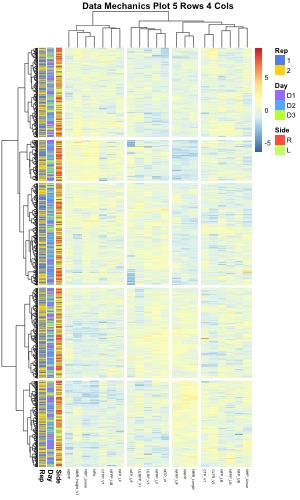

Supplement: Supplementary file 1 [file sensors-22-08347-s001.zip › SupplementalMaterials/Visualizations/DataMechanics/Muzzle/Geom/Image/ErrorEncoding_Image_R5C4.jpeg]

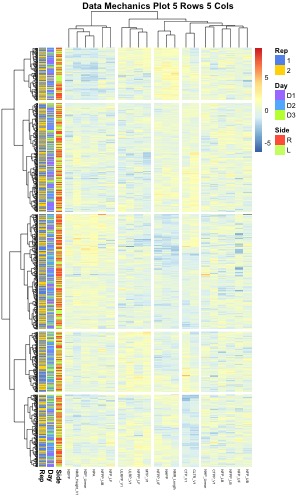

Supplement: Supplementary file 1 [file sensors-22-08347-s001.zip › SupplementalMaterials/Visualizations/DataMechanics/Muzzle/Geom/Image/ErrorEncoding_Image_R5C5.jpeg]

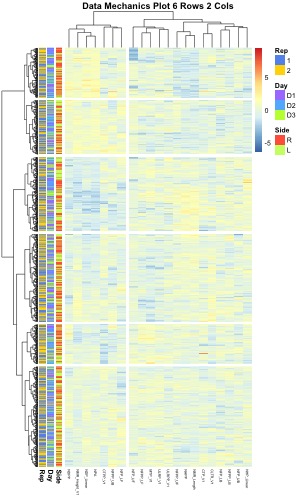

Supplement: Supplementary file 1 [file sensors-22-08347-s001.zip › SupplementalMaterials/Visualizations/DataMechanics/Muzzle/Geom/Image/ErrorEncoding_Image_R6C2.jpeg]

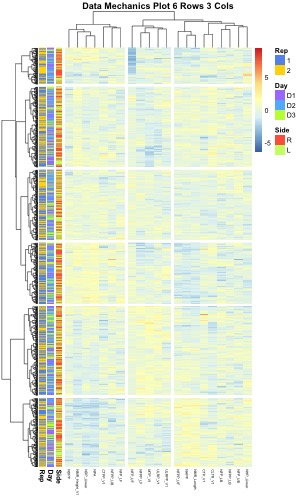

Supplement: Supplementary file 1 [file sensors-22-08347-s001.zip › SupplementalMaterials/Visualizations/DataMechanics/Muzzle/Geom/Image/ErrorEncoding_Image_R6C3.jpeg]

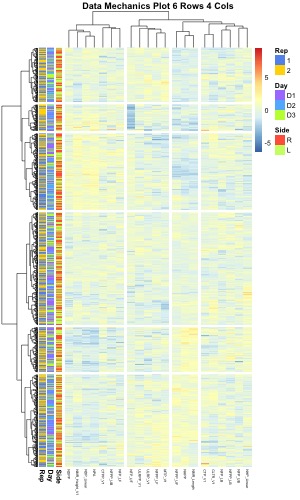

Supplement: Supplementary file 1 [file sensors-22-08347-s001.zip › SupplementalMaterials/Visualizations/DataMechanics/Muzzle/Geom/Image/ErrorEncoding_Image_R6C4.jpeg]

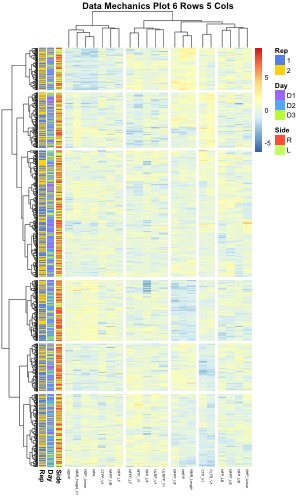

Supplement: Supplementary file 1 [file sensors-22-08347-s001.zip › SupplementalMaterials/Visualizations/DataMechanics/Muzzle/Geom/Image/ErrorEncoding_Image_R6C5.jpeg]

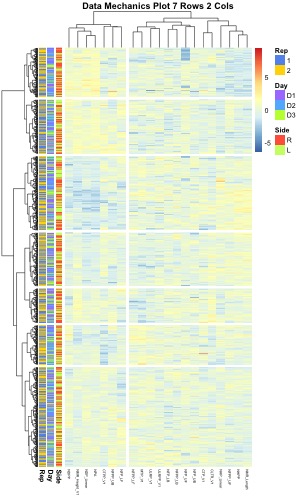

Supplement: Supplementary file 1 [file sensors-22-08347-s001.zip › SupplementalMaterials/Visualizations/DataMechanics/Muzzle/Geom/Image/ErrorEncoding_Image_R7C2.jpeg]

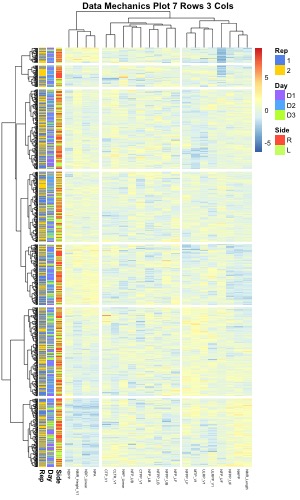

Supplement: Supplementary file 1 [file sensors-22-08347-s001.zip › SupplementalMaterials/Visualizations/DataMechanics/Muzzle/Geom/Image/ErrorEncoding_Image_R7C3.jpeg]
