# Supplementary material for: Improving the Reliability of Scale-Free Image Morphometrics in Applications with Minimally Restrained Livestock Using Projective Geometry and Unsupervised Machine Learning
Source: Sensors (Basel). 2022 Oct 31;22(21):8347. doi: 10.3390/s22218347 (PMC9653925; doi:10.3390/s22218347)
Supplement: Supplementary file 1 [file sensors-22-08347-s001.zip › SupplementalMaterials/Visualizations/ErrCorPlotAll.pdf]

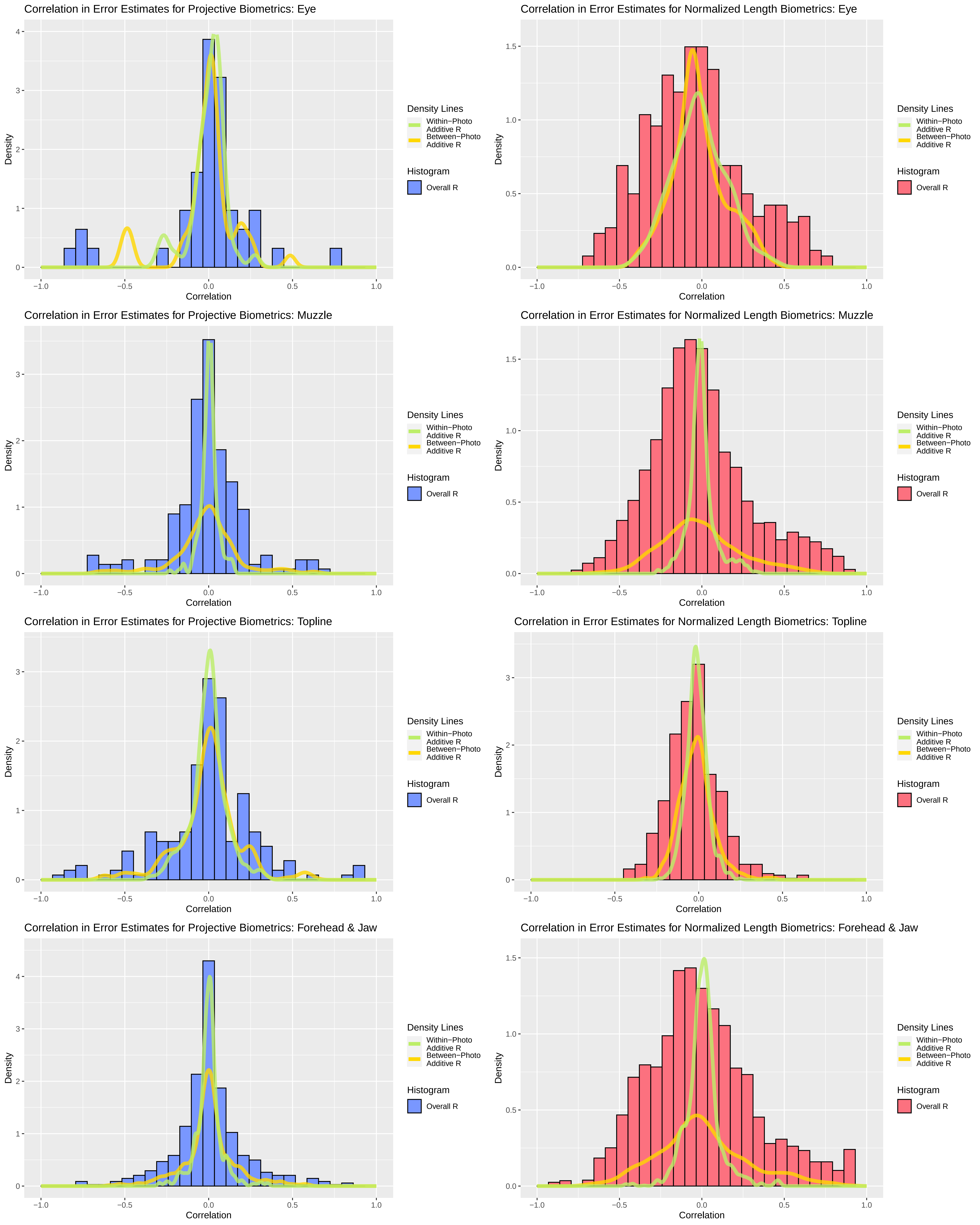
Correlation in Error Estimates for Projective Biometrics: Eye
Correlation in Error Estimates for Normalized Length Biometrics: Eye
Correlation in Error Estimates for Projective Biometrics: Muzzle
Correlation in Error Estimates for Normalized Length Biometrics: Muzzle
Correlation in Error Estimates for Projective Biometrics: Topline
Correlation in Error Estimates for Normalized Length Biometrics: Topline
Correlation in Error Estimates for Projective Biometrics: Forehead & Jaw
Correlation in Error Estimates for Normalized Length Biometrics: Forehead & Jaw
Density
Correlation
Density Lines
Within−Photo Additive R
Between−Photo Additive R
Histogram
Overall R
