# Supplementary material for: Improving the Reliability of Scale-Free Image Morphometrics in Applications with Minimally Restrained Livestock Using Projective Geometry and Unsupervised Machine Learning
Source: Sensors (Basel). 2022 Oct 31;22(21):8347. doi: 10.3390/s22218347 (PMC9653925; doi:10.3390/s22218347)
Supplement: Supplementary file 1 [file sensors-22-08347-s001.zip › SupplementalMaterials/Visualizations/HMFAPlotAll.pdf]

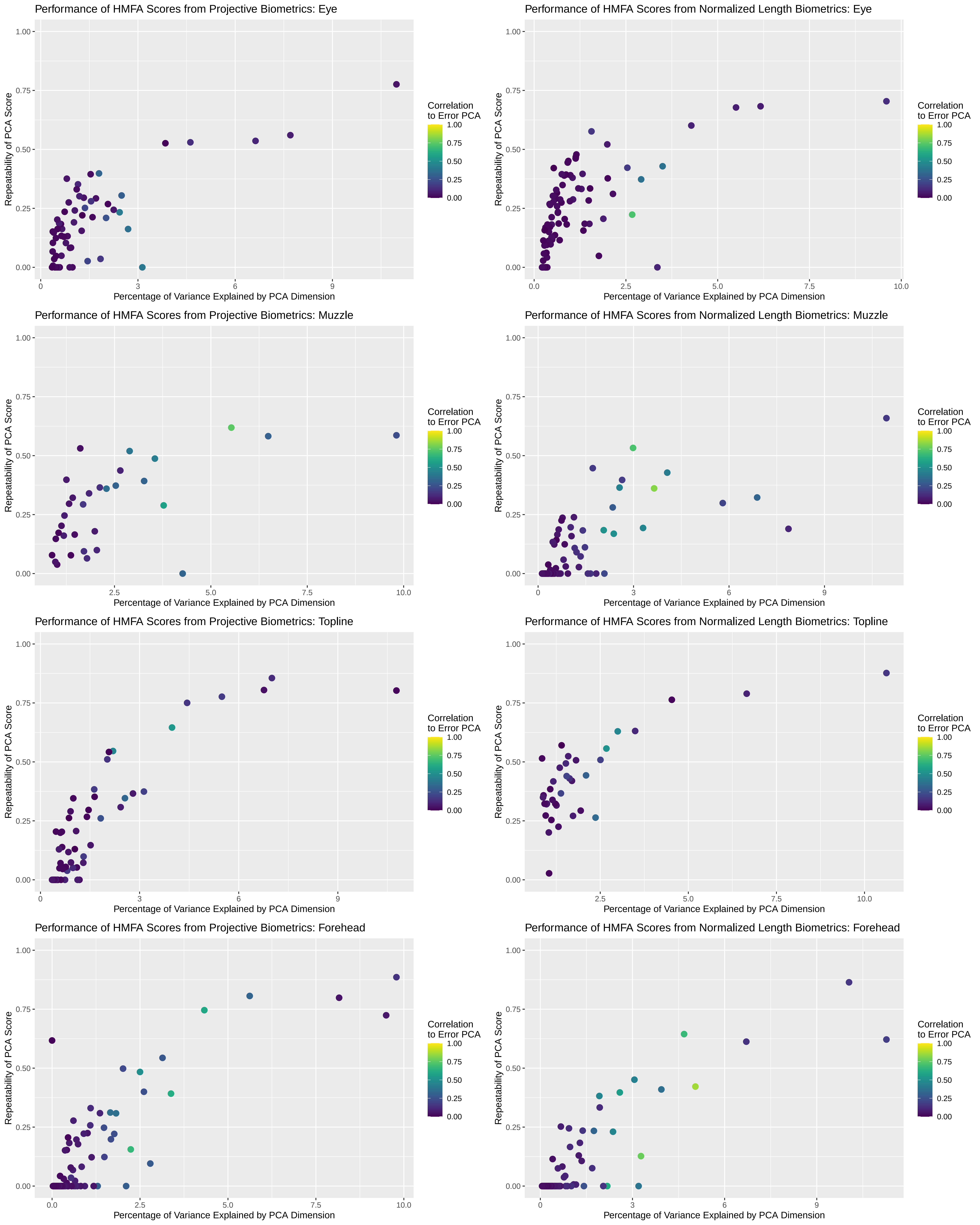

Performance of HMFA Scores from Projective Biometrics: Eye
Performance of HMFA Scores from Normalized Length Biometrics: Eye
Performance of HMFA Scores from Projective Biometrics: Muzzle
Performance of HMFA Scores from Normalized Length Biometrics: Muzzle
Performance of HMFA Scores from Projective Biometrics: Topline
Performance of HMFA Scores from Normalized Length Biometrics: Topline
Performance of HMFA Scores from Projective Biometrics: Forehead
Performance of HMFA Scores from Normalized Length Biometrics: Forehead
Repeatability of PCA Score
Percentage of Variance Explained by PCA Dimension
Correlation to Error PCA
1.00
0.75
0.50
0.25
0.00
