# Supplementary material for: Improving the Reliability of Scale-Free Image Morphometrics in Applications with Minimally Restrained Livestock Using Projective Geometry and Unsupervised Machine Learning
Source: Sensors (Basel). 2022 Oct 31;22(21):8347. doi: 10.3390/s22218347 (PMC9653925; doi:10.3390/s22218347)
Supplement: Supplementary file 1 [file sensors-22-08347-s001.zip › SupplementalMaterials/Visualizations/RepPlotAll.pdf]

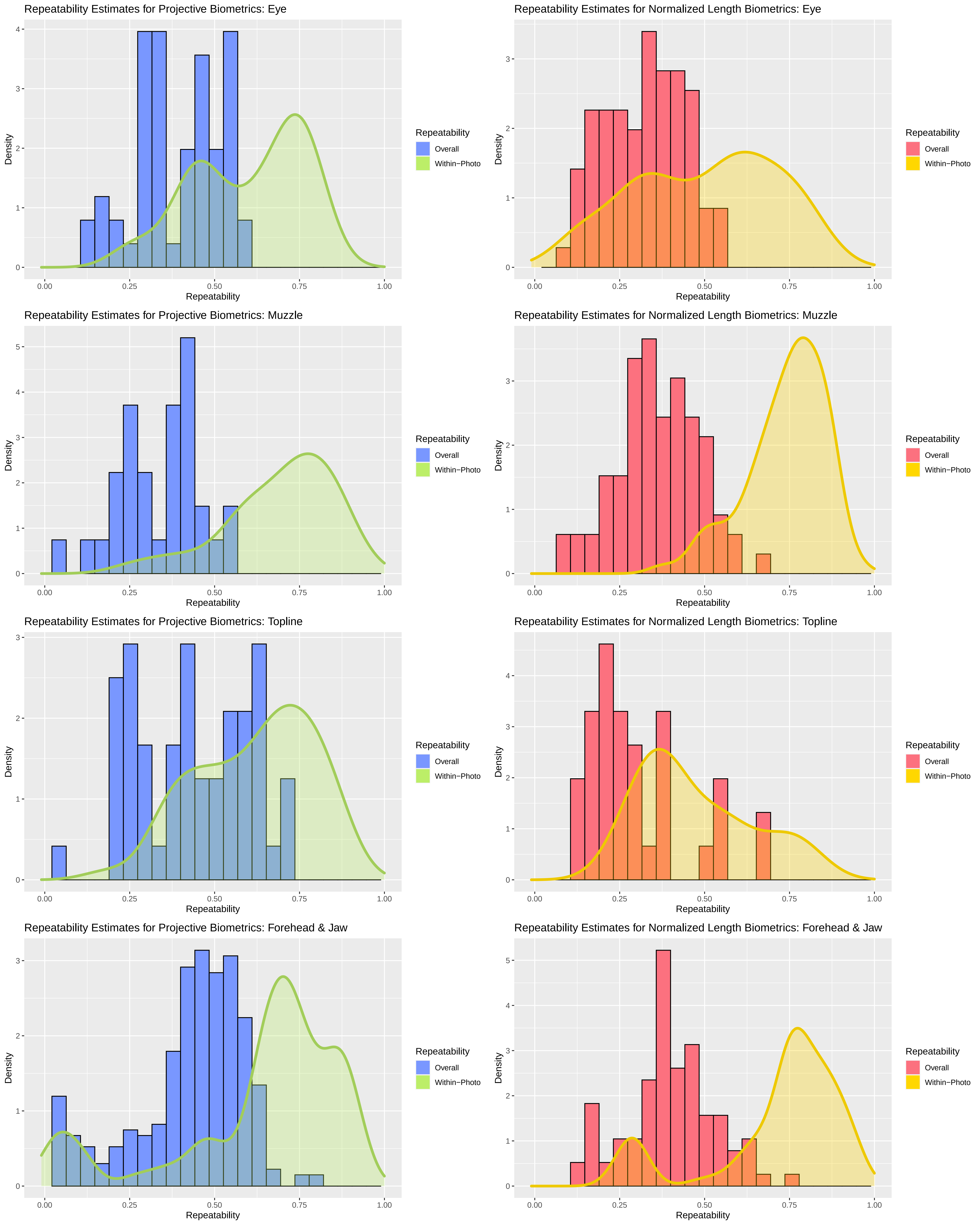

Repeatability Estimates for Projective Biometrics: Eye
Repeatability Estimates for Normalized Length Biometrics: Eye
Repeatability Estimates for Projective Biometrics: Muzzle
Repeatability Estimates for Normalized Length Biometrics: Muzzle
Repeatability Estimates for Projective Biometrics: Topline
Repeatability Estimates for Normalized Length Biometrics: Topline
Repeatability Estimates for Projective Biometrics: Forehead & Jaw
Repeatability Estimates for Normalized Length Biometrics: Forehead & Jaw
Density
Repeatability
0.00
0.25
0.50
0.75
1.00
Repeatability
Overall
Within−Photo
